# Supplementary figures and images for: DES-YOLO: a novel model for real-time detection of casting surface defects (part 2 of 2)
Source: PeerJ Comput Sci. 2024 Aug 22;10:e2224. doi: 10.7717/peerj-cs.2224 (PMC11419617; doi:10.7717/peerj-cs.2224)

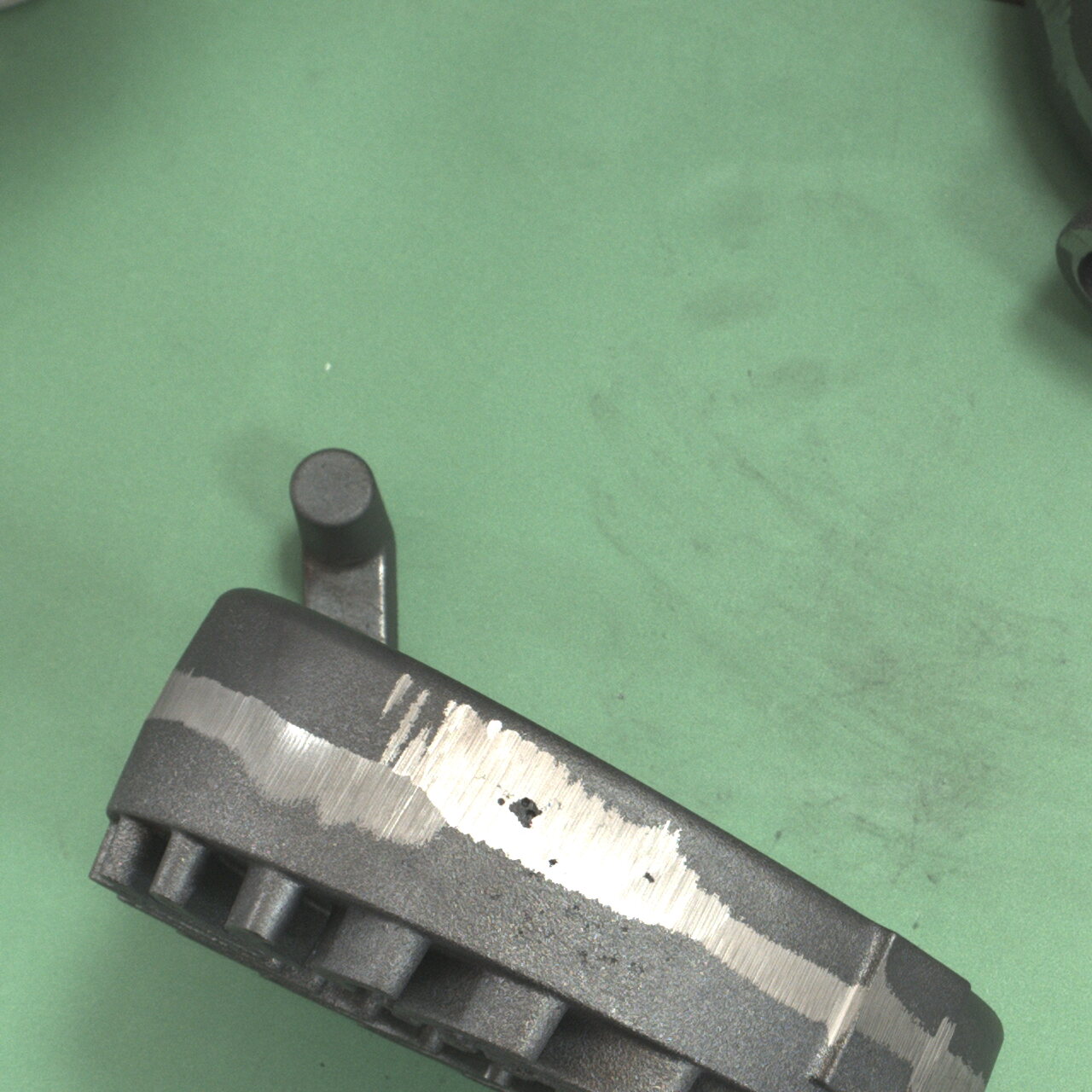

Supplement: Supplemental Information 1 — The CSD-DET dataset was collected from Guangde Hardware Casting Factory and Wuhu Automobile Casting Factory in May 2023. The CSD-DET dataset was used to train and measure the advantages of the DES-YOLO model. This is the filtered partial dataset. [file peerj-cs-10-2224-s001.zip › CastingDefectsDataSet/data/Sl_42.jpg]

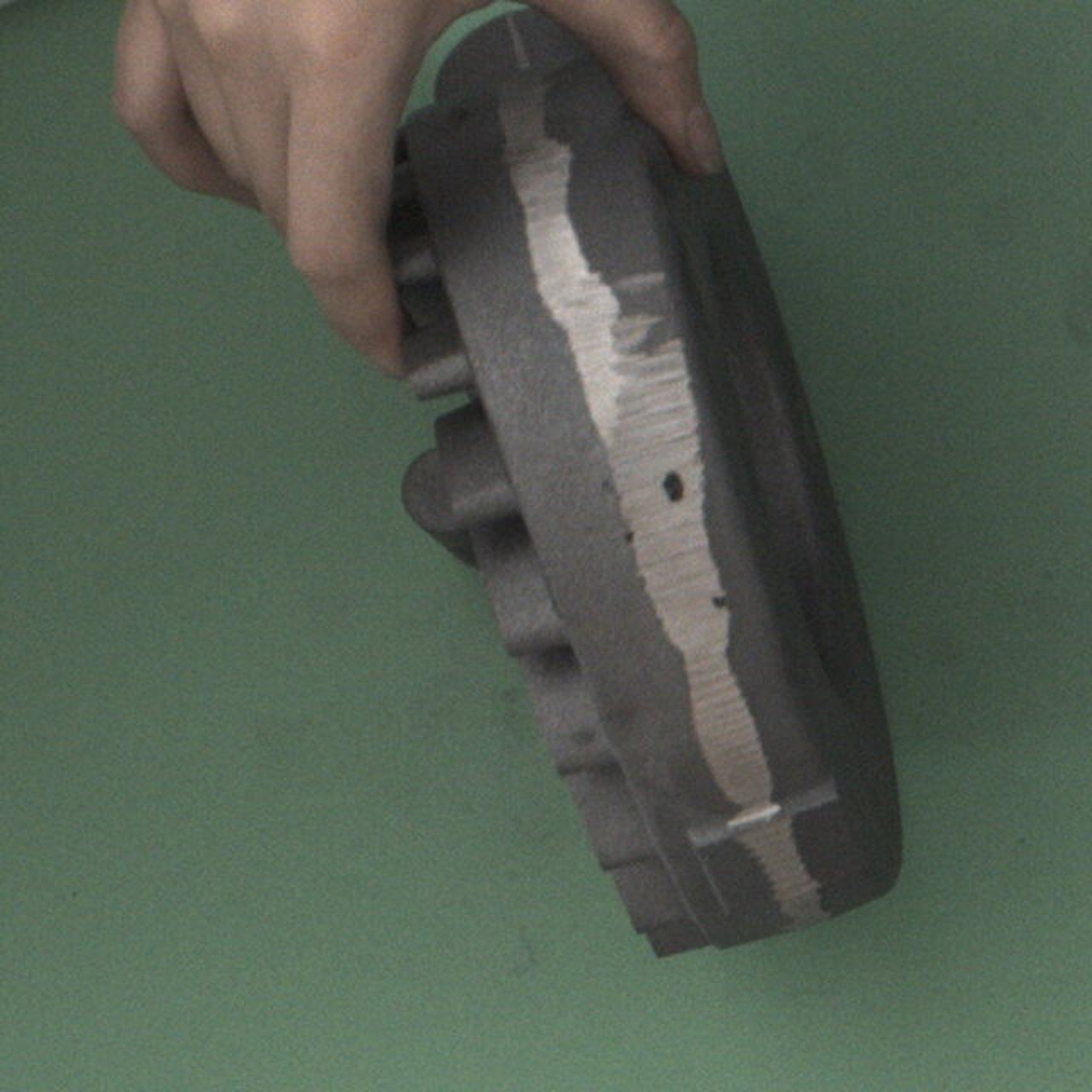

Supplement: Supplemental Information 1 — The CSD-DET dataset was collected from Guangde Hardware Casting Factory and Wuhu Automobile Casting Factory in May 2023. The CSD-DET dataset was used to train and measure the advantages of the DES-YOLO model. This is the filtered partial dataset. [file peerj-cs-10-2224-s001.zip › CastingDefectsDataSet/data/Sl_478.jpg]

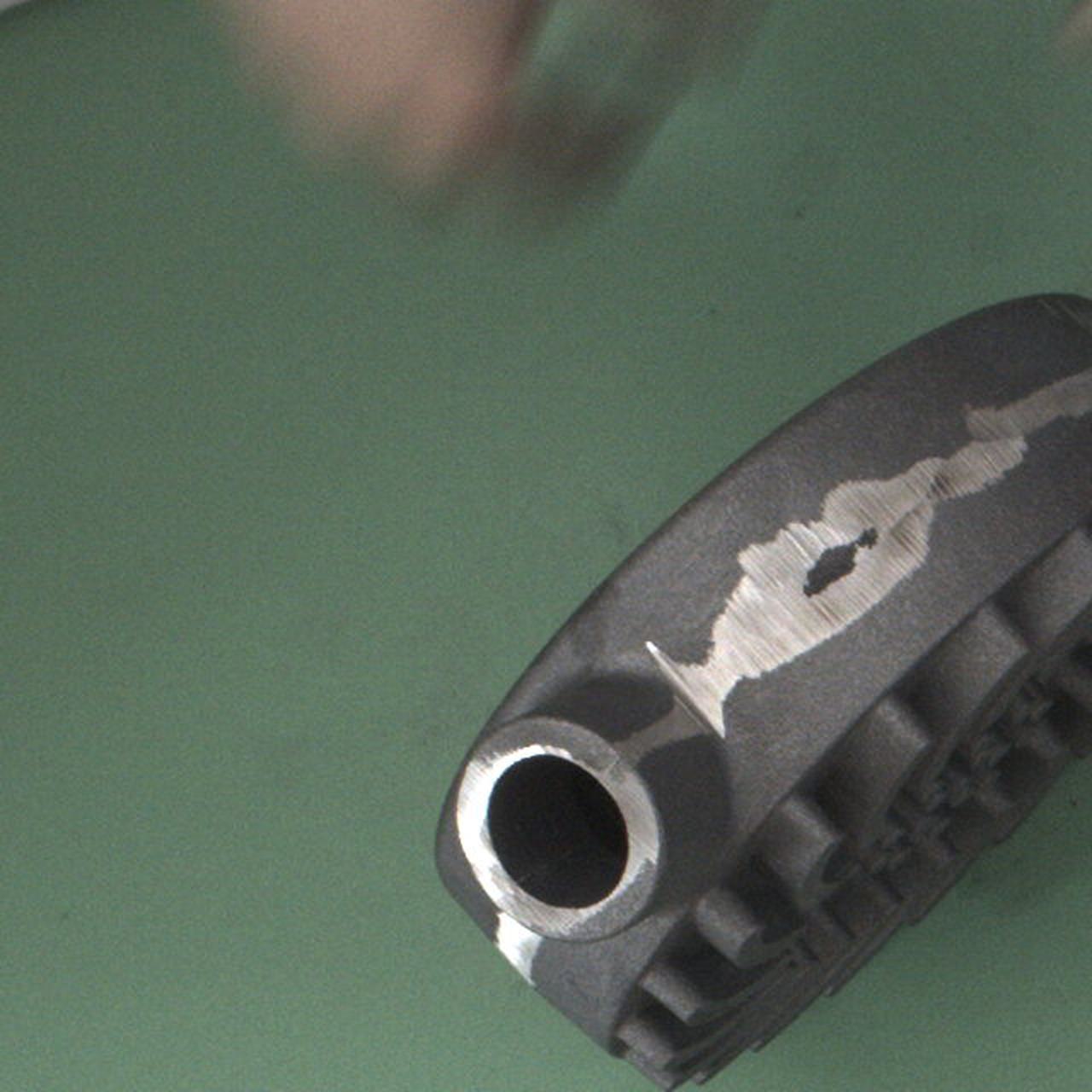

Supplement: Supplemental Information 1 — The CSD-DET dataset was collected from Guangde Hardware Casting Factory and Wuhu Automobile Casting Factory in May 2023. The CSD-DET dataset was used to train and measure the advantages of the DES-YOLO model. This is the filtered partial dataset. [file peerj-cs-10-2224-s001.zip › CastingDefectsDataSet/data/Sl_490.jpg]

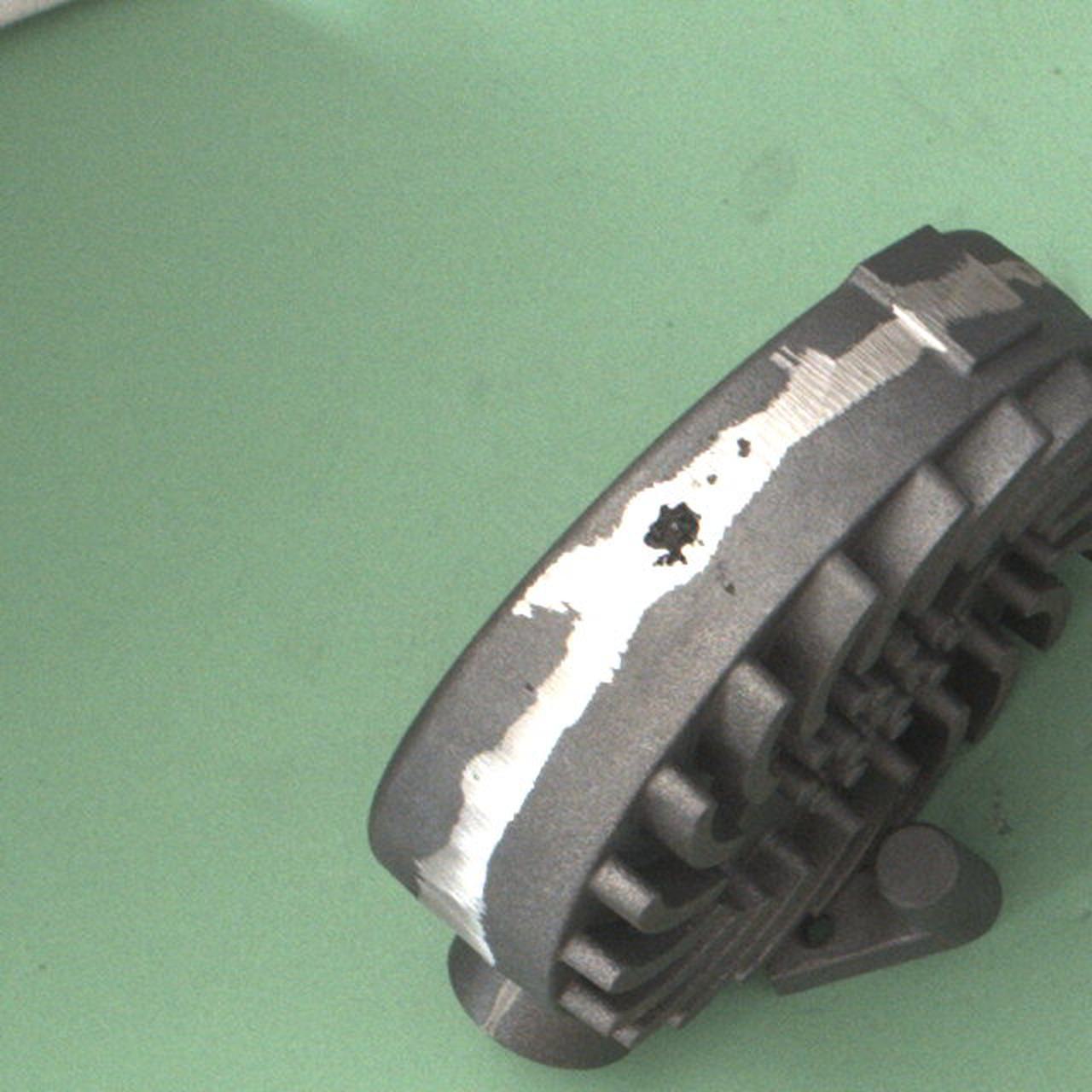

Supplement: Supplemental Information 1 — The CSD-DET dataset was collected from Guangde Hardware Casting Factory and Wuhu Automobile Casting Factory in May 2023. The CSD-DET dataset was used to train and measure the advantages of the DES-YOLO model. This is the filtered partial dataset. [file peerj-cs-10-2224-s001.zip › CastingDefectsDataSet/data/Sl_522.jpg]

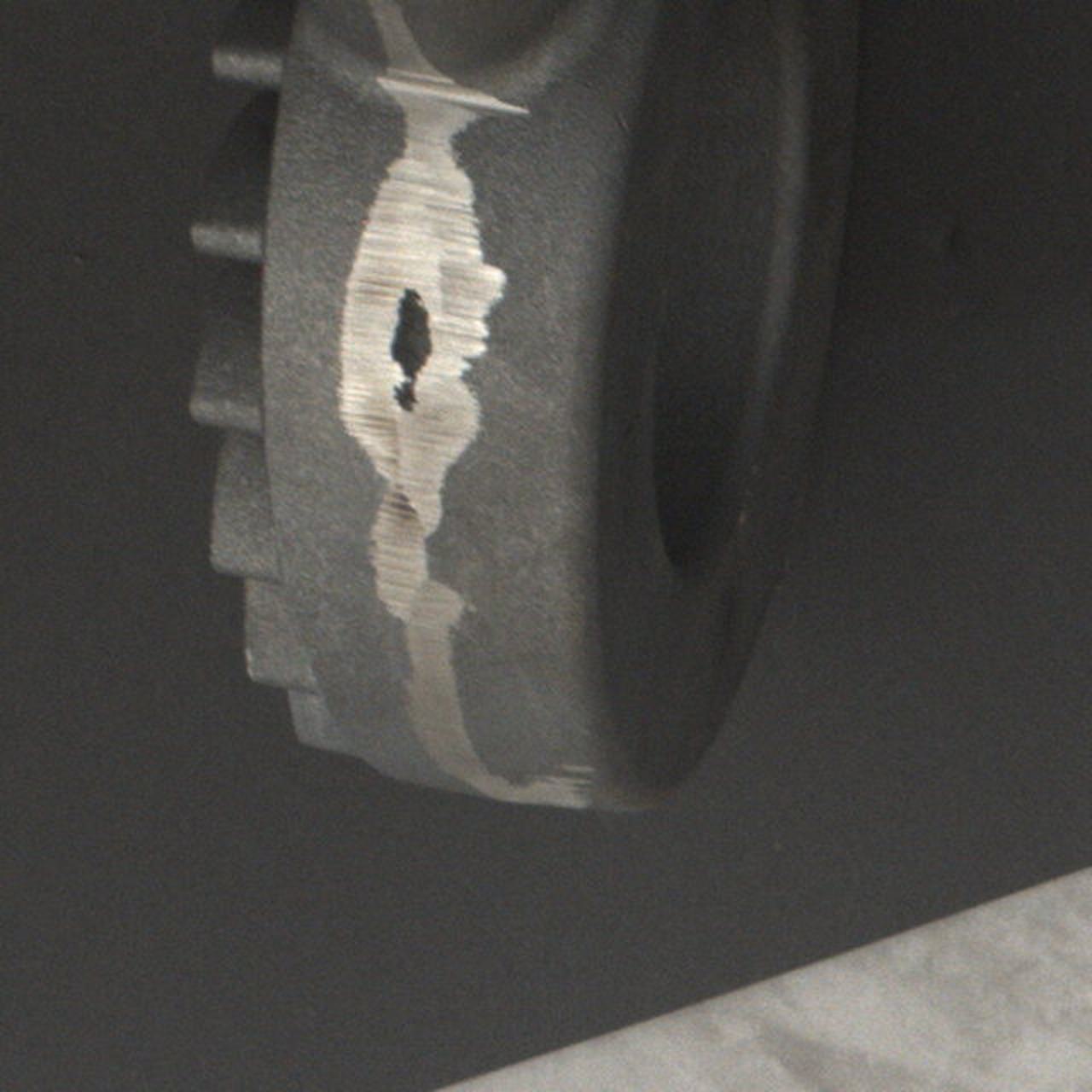

Supplement: Supplemental Information 1 — The CSD-DET dataset was collected from Guangde Hardware Casting Factory and Wuhu Automobile Casting Factory in May 2023. The CSD-DET dataset was used to train and measure the advantages of the DES-YOLO model. This is the filtered partial dataset. [file peerj-cs-10-2224-s001.zip › CastingDefectsDataSet/data/Sl_618.jpg]

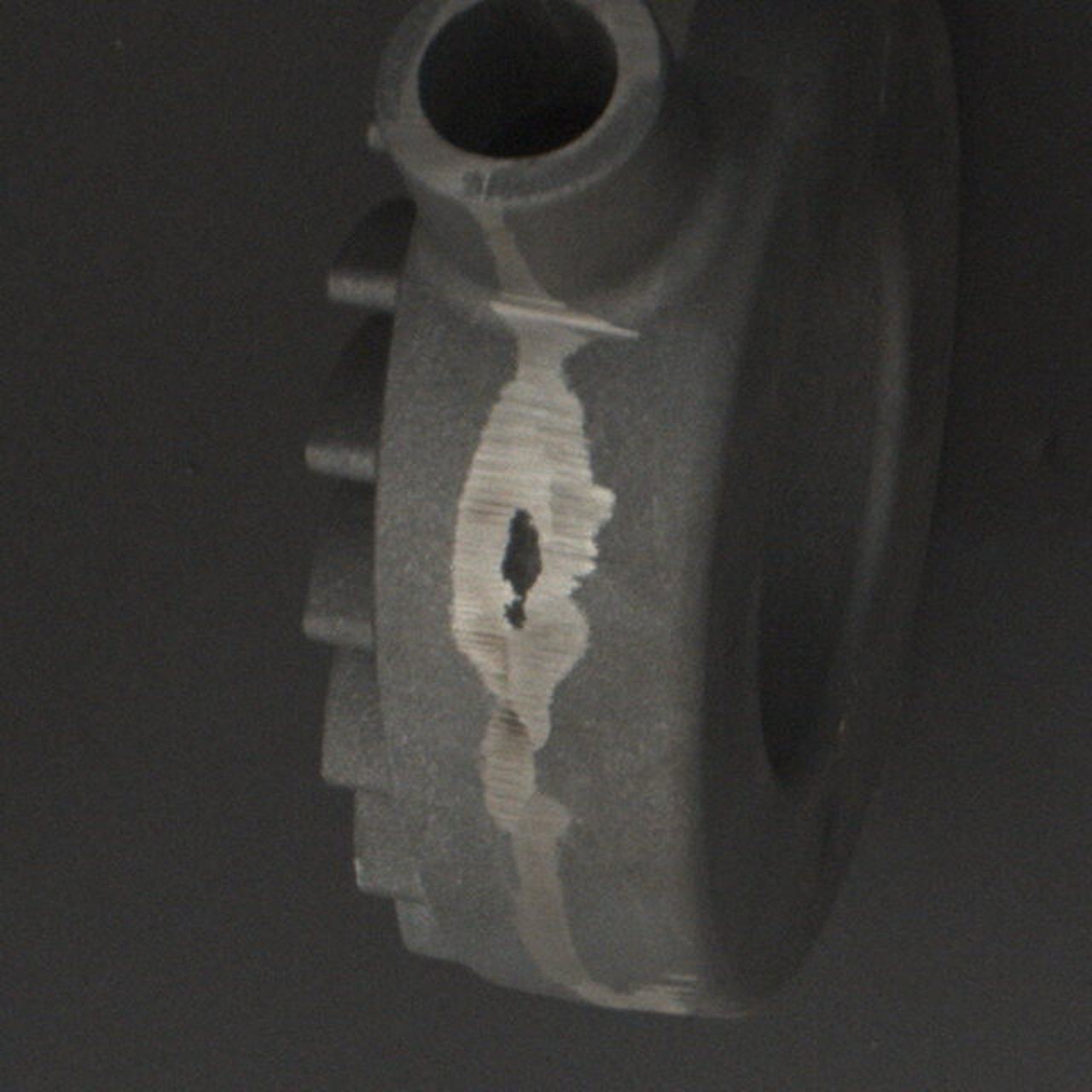

Supplement: Supplemental Information 1 — The CSD-DET dataset was collected from Guangde Hardware Casting Factory and Wuhu Automobile Casting Factory in May 2023. The CSD-DET dataset was used to train and measure the advantages of the DES-YOLO model. This is the filtered partial dataset. [file peerj-cs-10-2224-s001.zip › CastingDefectsDataSet/data/Sl_622.jpg]

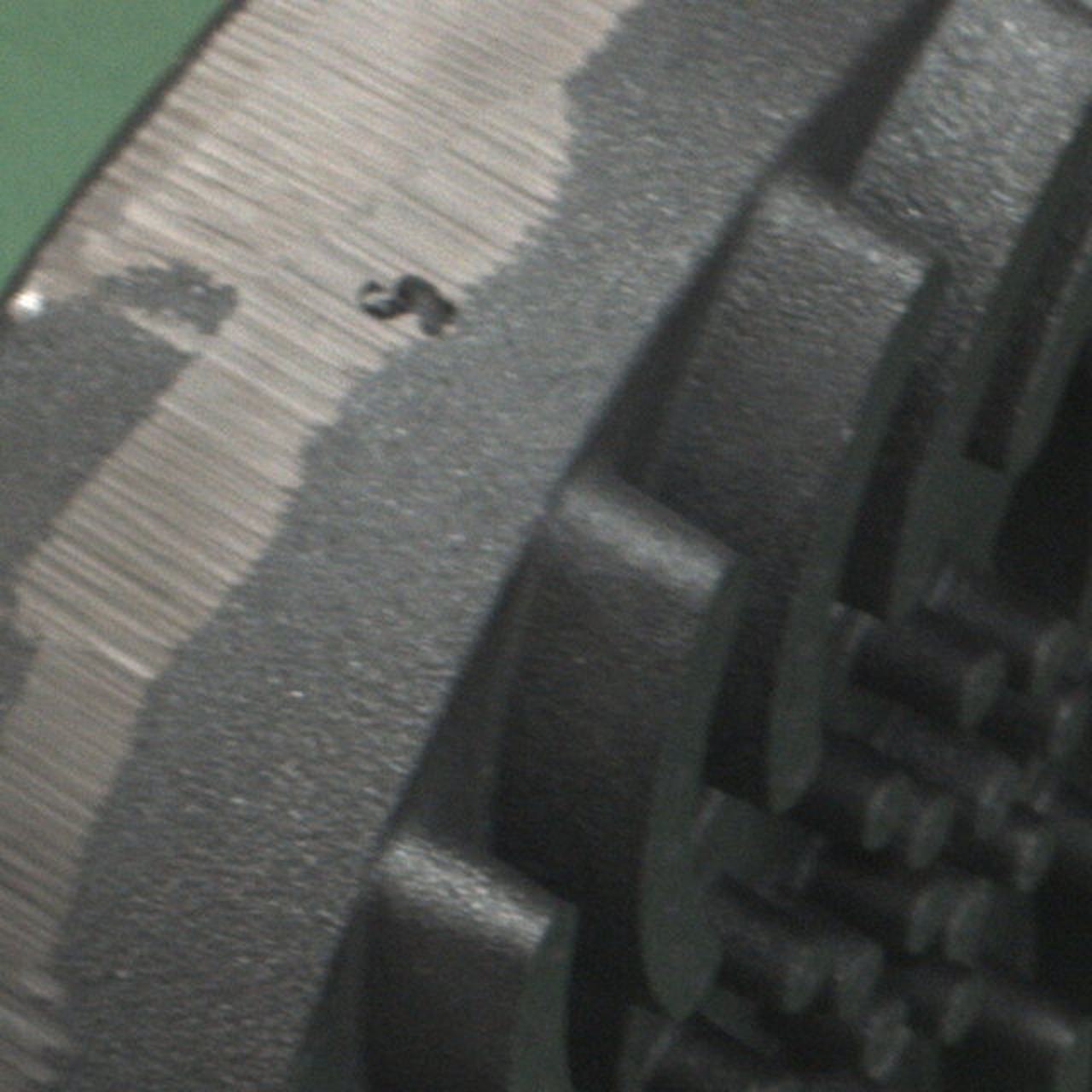

Supplement: Supplemental Information 1 — The CSD-DET dataset was collected from Guangde Hardware Casting Factory and Wuhu Automobile Casting Factory in May 2023. The CSD-DET dataset was used to train and measure the advantages of the DES-YOLO model. This is the filtered partial dataset. [file peerj-cs-10-2224-s001.zip › CastingDefectsDataSet/data/Sl_670.jpg]

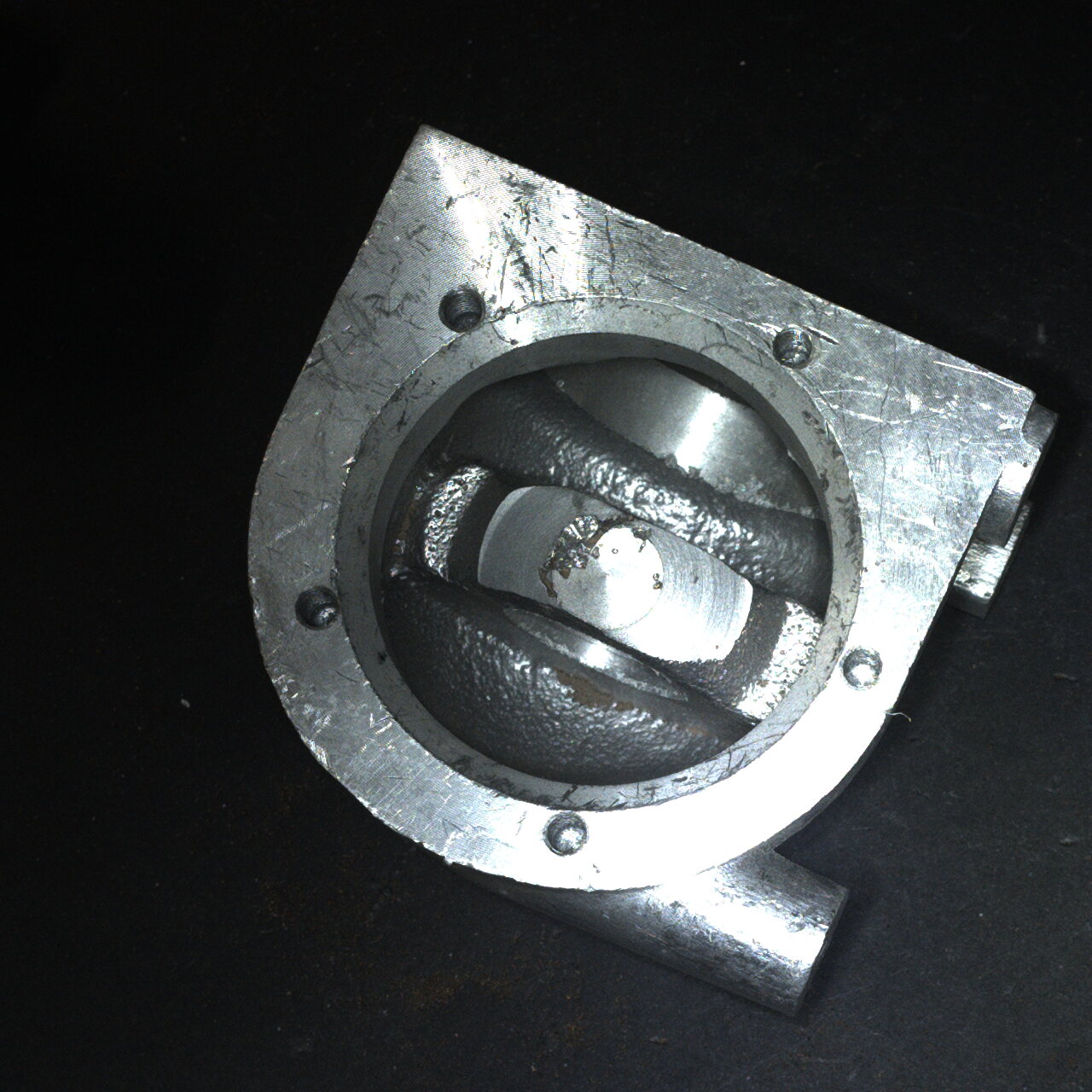

Supplement: Supplemental Information 1 — The CSD-DET dataset was collected from Guangde Hardware Casting Factory and Wuhu Automobile Casting Factory in May 2023. The CSD-DET dataset was used to train and measure the advantages of the DES-YOLO model. This is the filtered partial dataset. [file peerj-cs-10-2224-s001.zip › CastingDefectsDataSet/data/Sl_782.jpg]

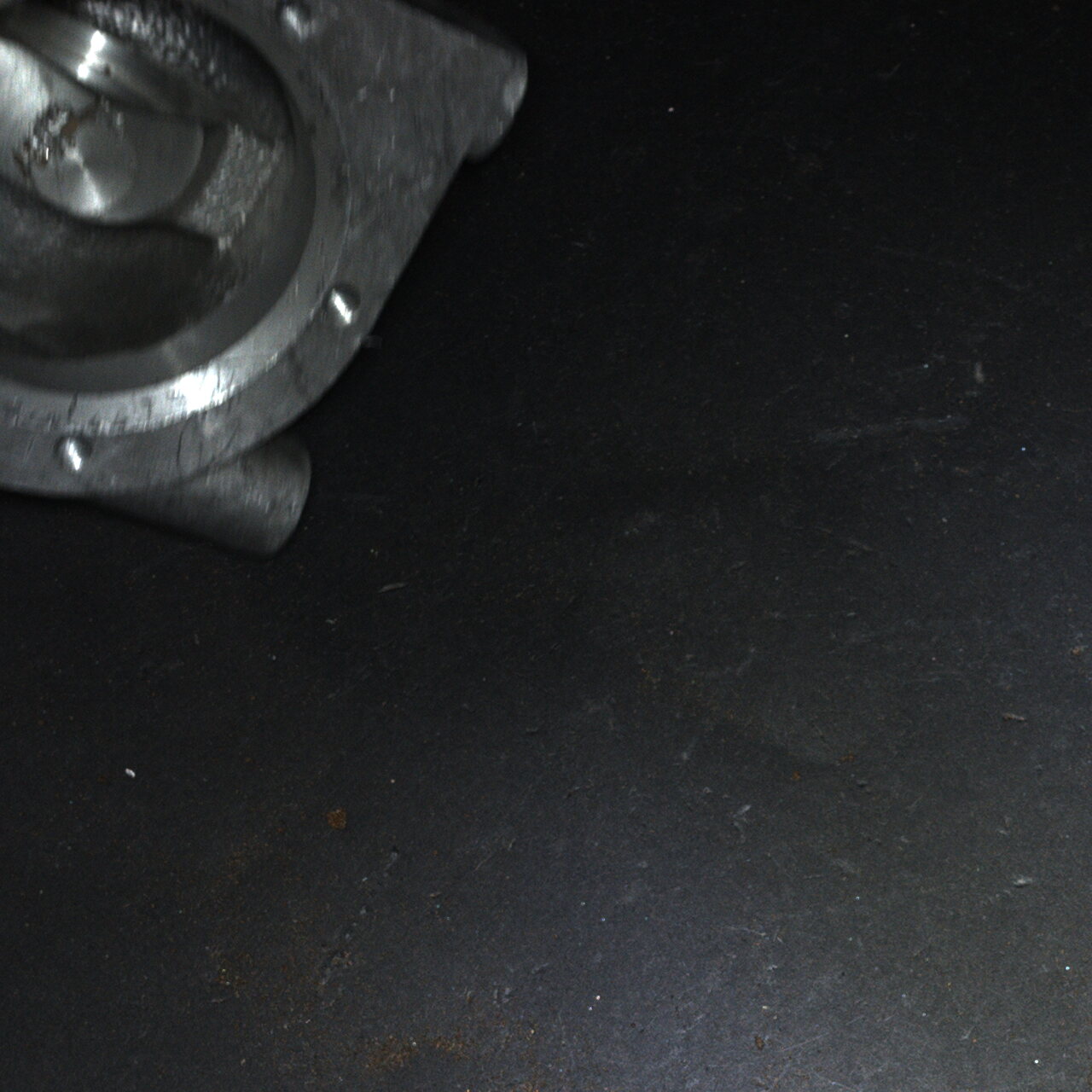

Supplement: Supplemental Information 1 — The CSD-DET dataset was collected from Guangde Hardware Casting Factory and Wuhu Automobile Casting Factory in May 2023. The CSD-DET dataset was used to train and measure the advantages of the DES-YOLO model. This is the filtered partial dataset. [file peerj-cs-10-2224-s001.zip › CastingDefectsDataSet/data/Sl_802.jpg]

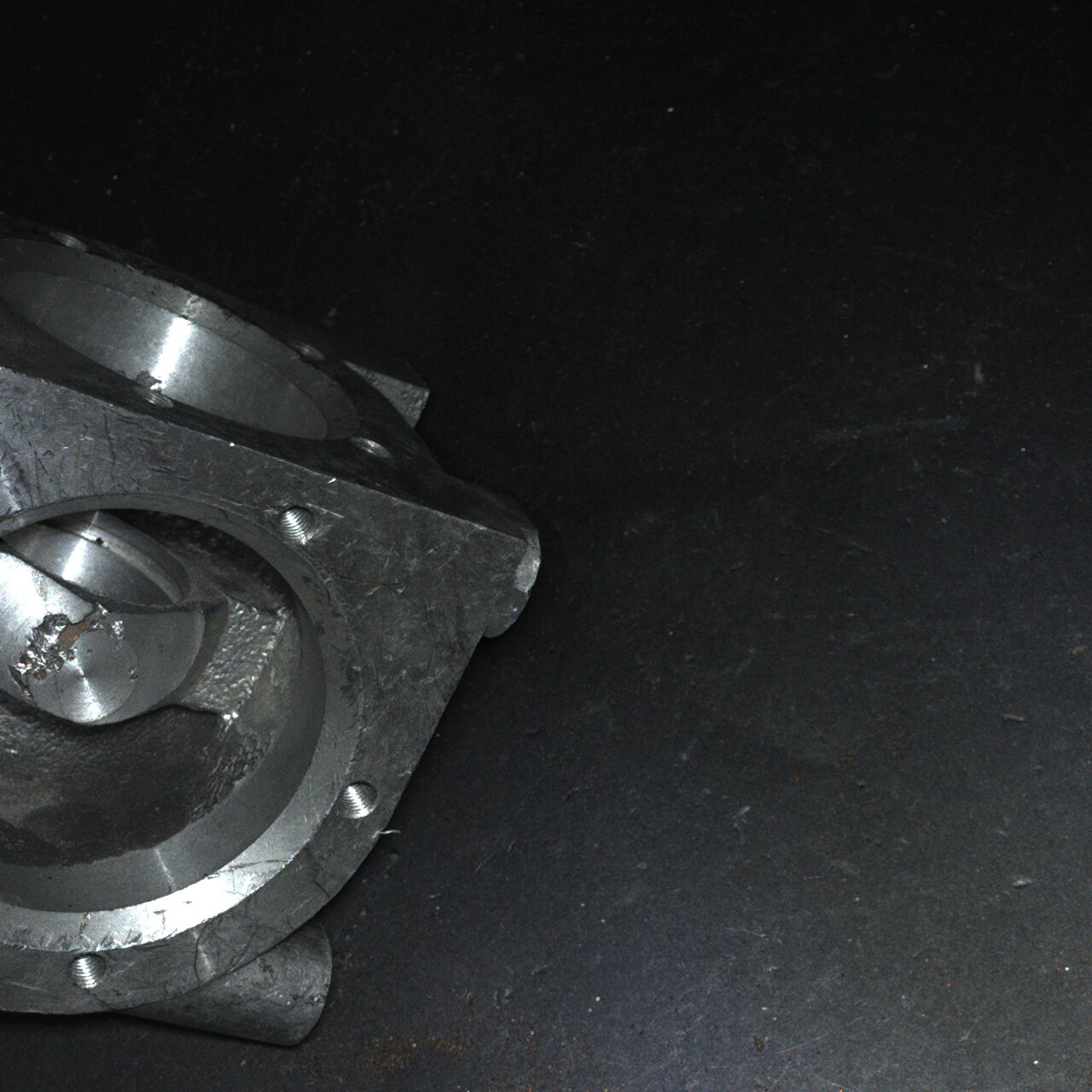

Supplement: Supplemental Information 1 — The CSD-DET dataset was collected from Guangde Hardware Casting Factory and Wuhu Automobile Casting Factory in May 2023. The CSD-DET dataset was used to train and measure the advantages of the DES-YOLO model. This is the filtered partial dataset. [file peerj-cs-10-2224-s001.zip › CastingDefectsDataSet/data/Sl_806.jpg]

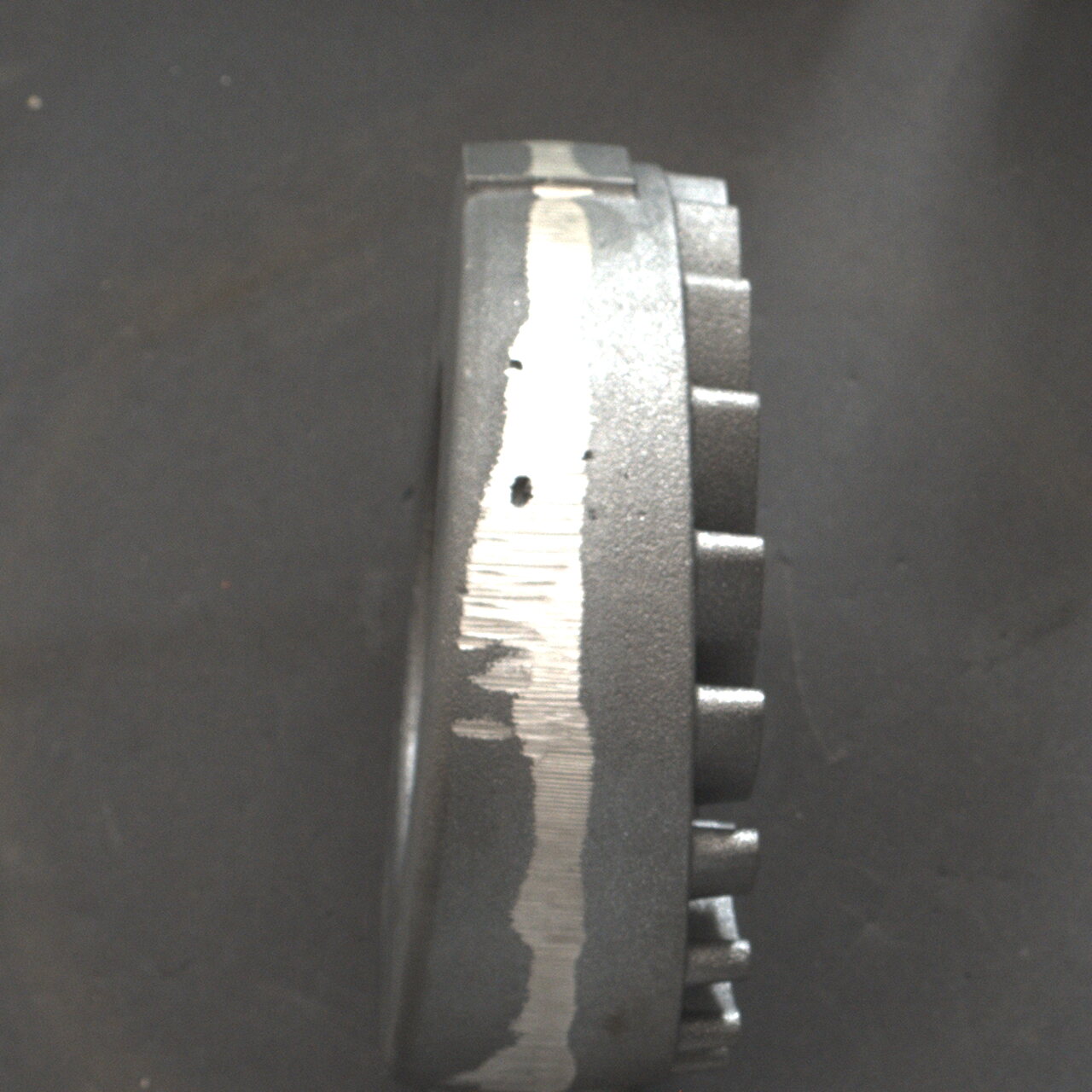

Supplement: Supplemental Information 1 — The CSD-DET dataset was collected from Guangde Hardware Casting Factory and Wuhu Automobile Casting Factory in May 2023. The CSD-DET dataset was used to train and measure the advantages of the DES-YOLO model. This is the filtered partial dataset. [file peerj-cs-10-2224-s001.zip › CastingDefectsDataSet/data/Sl_90.jpg]

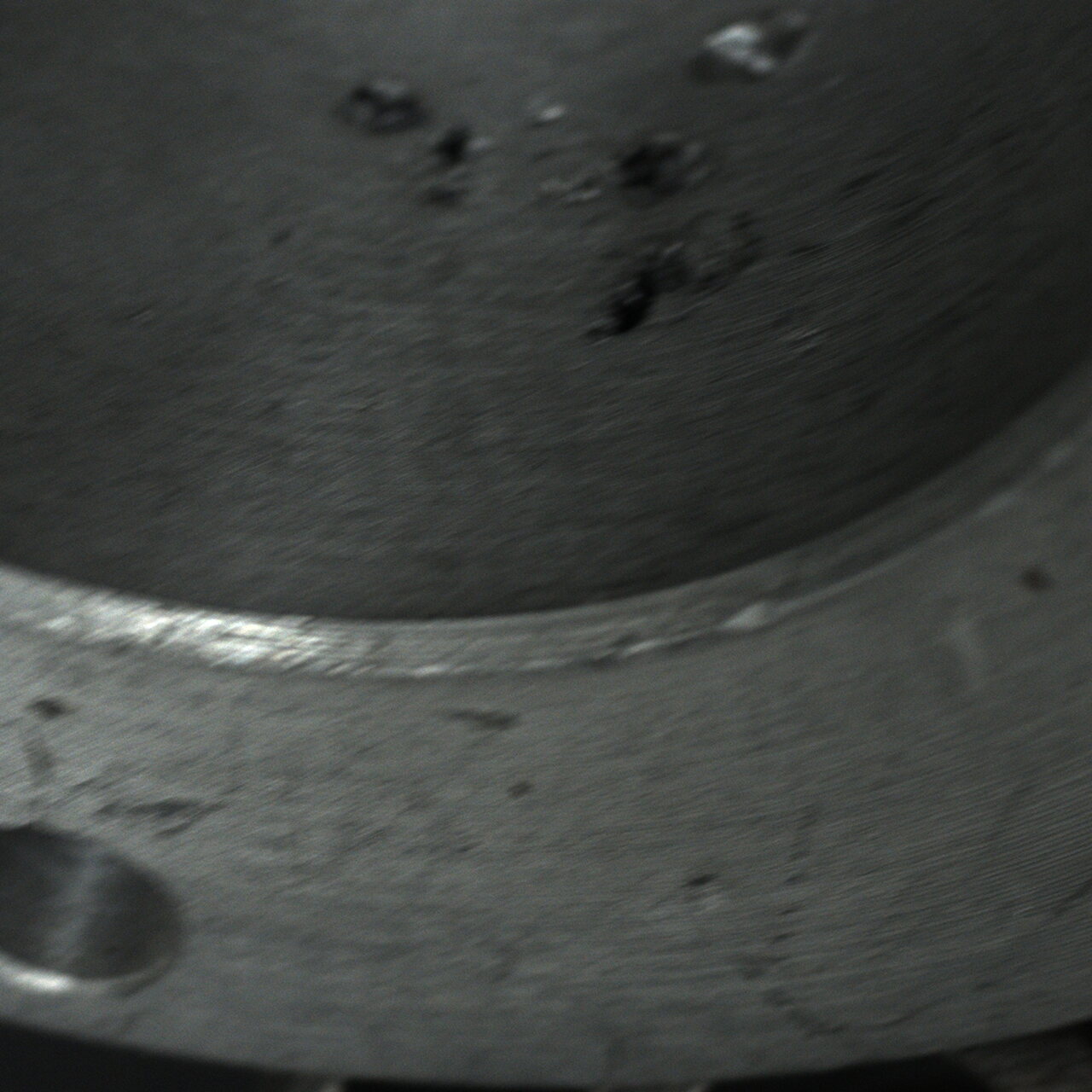

Supplement: Supplemental Information 1 — The CSD-DET dataset was collected from Guangde Hardware Casting Factory and Wuhu Automobile Casting Factory in May 2023. The CSD-DET dataset was used to train and measure the advantages of the DES-YOLO model. This is the filtered partial dataset. [file peerj-cs-10-2224-s001.zip › CastingDefectsDataSet/data/Sl_910.jpg]
