# Supplementary figures and images for: DES-YOLO: a novel model for real-time detection of casting surface defects (part 1 of 2)
Source: PeerJ Comput Sci. 2024 Aug 22;10:e2224. doi: 10.7717/peerj-cs.2224 (PMC11419617; doi:10.7717/peerj-cs.2224)

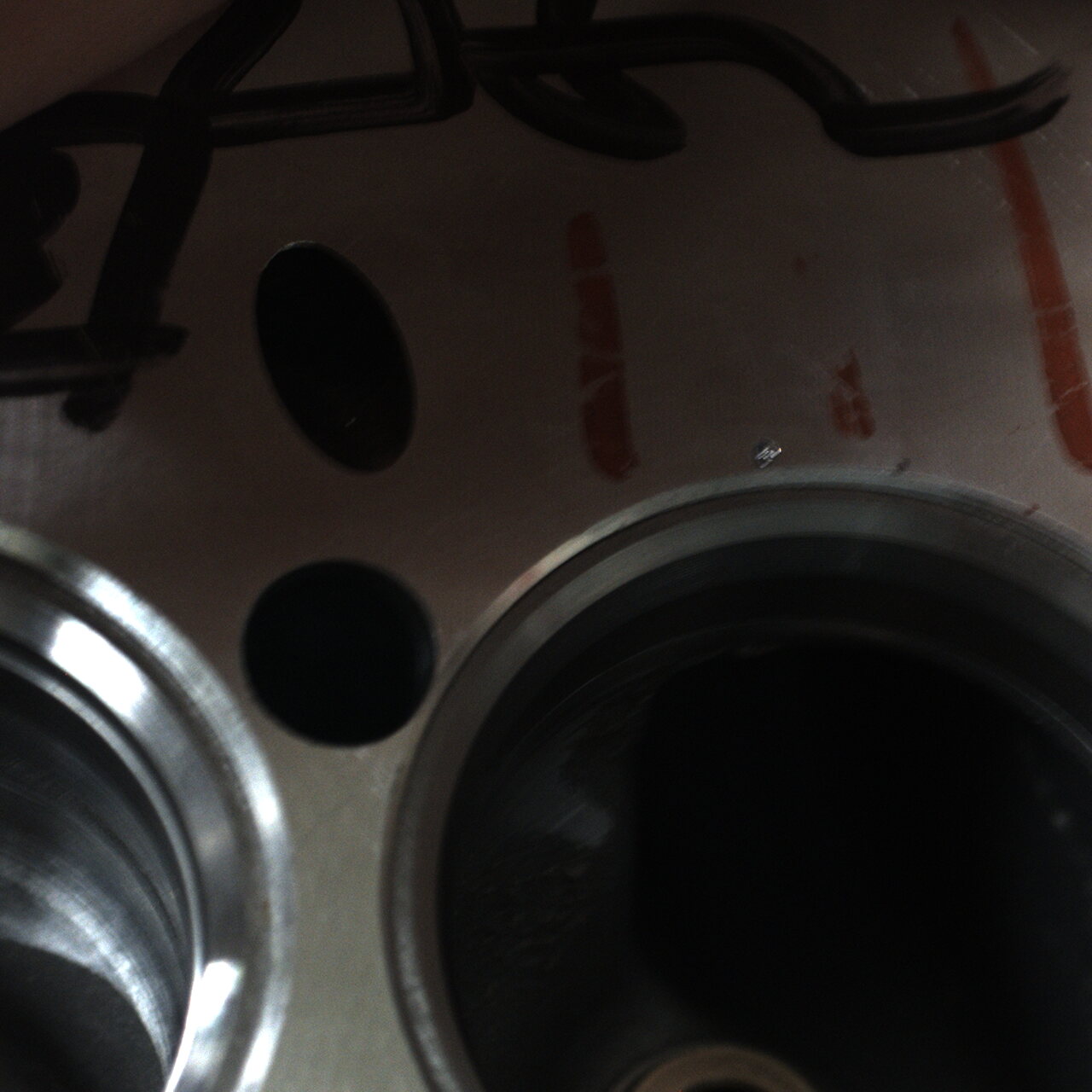

Supplement: Supplemental Information 1 — The CSD-DET dataset was collected from Guangde Hardware Casting Factory and Wuhu Automobile Casting Factory in May 2023. The CSD-DET dataset was used to train and measure the advantages of the DES-YOLO model. This is the filtered partial dataset. [file peerj-cs-10-2224-s001.zip › CastingDefectsDataSet/data/Bh_1.jpg]

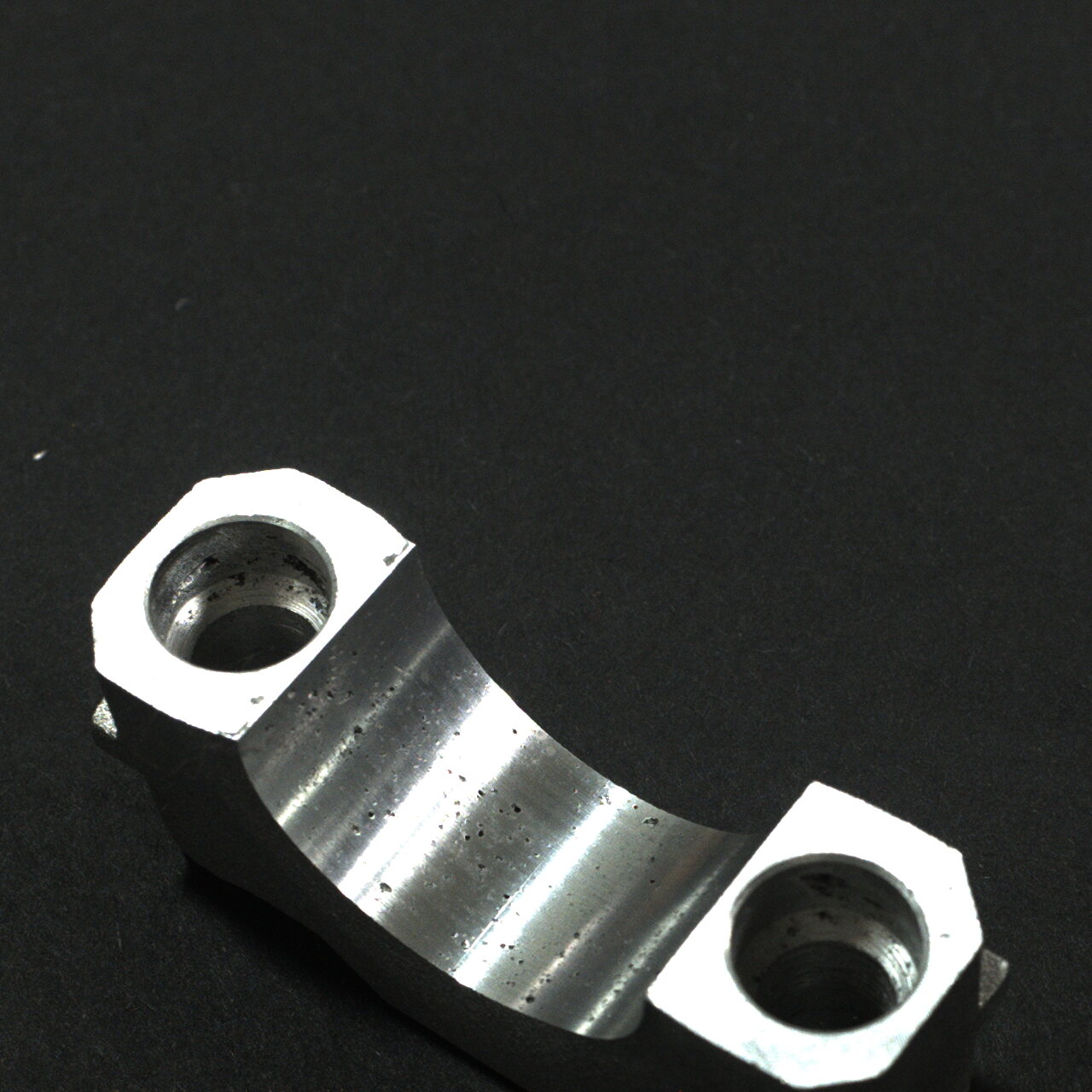

Supplement: Supplemental Information 1 — The CSD-DET dataset was collected from Guangde Hardware Casting Factory and Wuhu Automobile Casting Factory in May 2023. The CSD-DET dataset was used to train and measure the advantages of the DES-YOLO model. This is the filtered partial dataset. [file peerj-cs-10-2224-s001.zip › CastingDefectsDataSet/data/Bh_121.jpg]

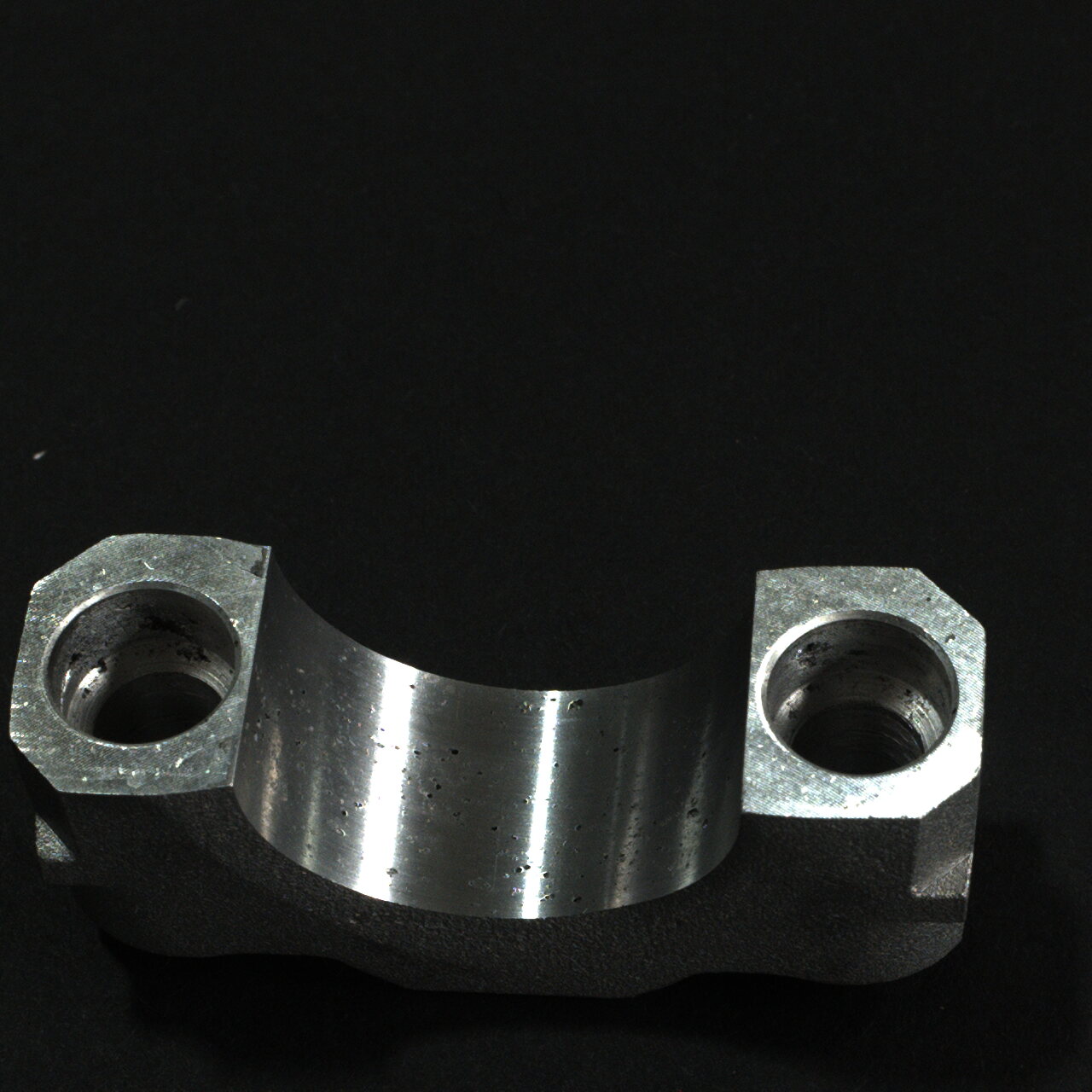

Supplement: Supplemental Information 1 — The CSD-DET dataset was collected from Guangde Hardware Casting Factory and Wuhu Automobile Casting Factory in May 2023. The CSD-DET dataset was used to train and measure the advantages of the DES-YOLO model. This is the filtered partial dataset. [file peerj-cs-10-2224-s001.zip › CastingDefectsDataSet/data/Bh_125.jpg]

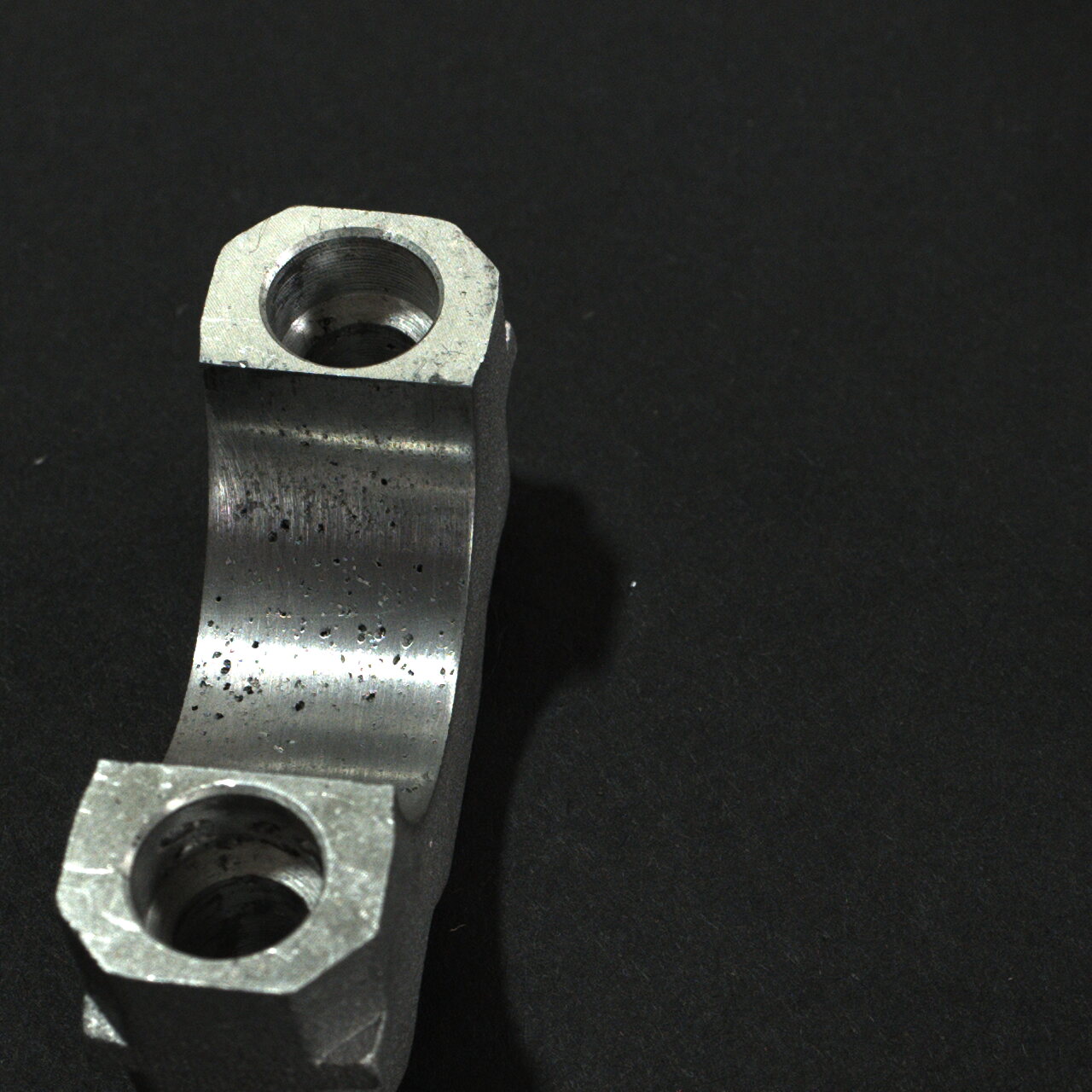

Supplement: Supplemental Information 1 — The CSD-DET dataset was collected from Guangde Hardware Casting Factory and Wuhu Automobile Casting Factory in May 2023. The CSD-DET dataset was used to train and measure the advantages of the DES-YOLO model. This is the filtered partial dataset. [file peerj-cs-10-2224-s001.zip › CastingDefectsDataSet/data/Bh_181.jpg]

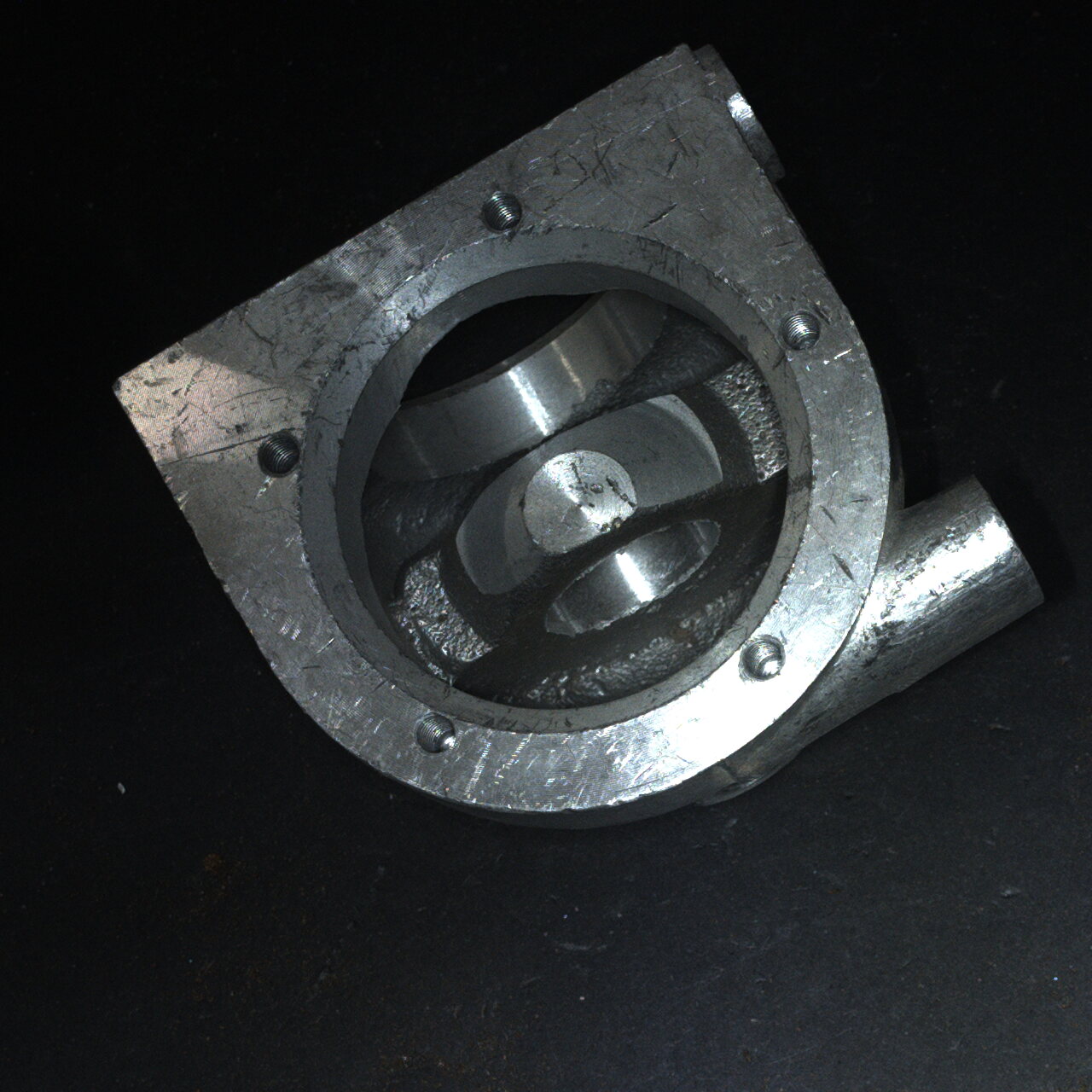

Supplement: Supplemental Information 1 — The CSD-DET dataset was collected from Guangde Hardware Casting Factory and Wuhu Automobile Casting Factory in May 2023. The CSD-DET dataset was used to train and measure the advantages of the DES-YOLO model. This is the filtered partial dataset. [file peerj-cs-10-2224-s001.zip › CastingDefectsDataSet/data/Bh_269.jpg]

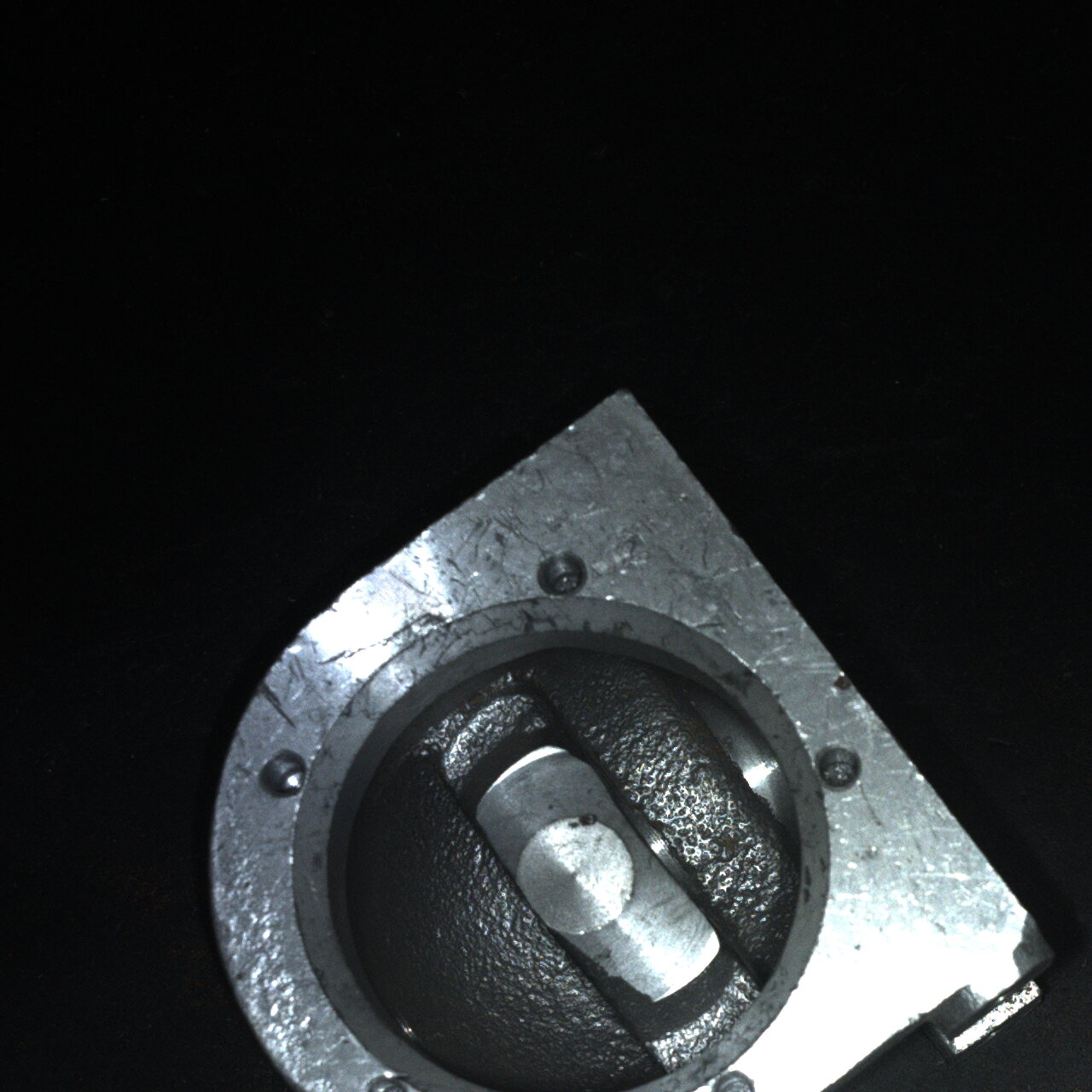

Supplement: Supplemental Information 1 — The CSD-DET dataset was collected from Guangde Hardware Casting Factory and Wuhu Automobile Casting Factory in May 2023. The CSD-DET dataset was used to train and measure the advantages of the DES-YOLO model. This is the filtered partial dataset. [file peerj-cs-10-2224-s001.zip › CastingDefectsDataSet/data/Bh_289.jpg]

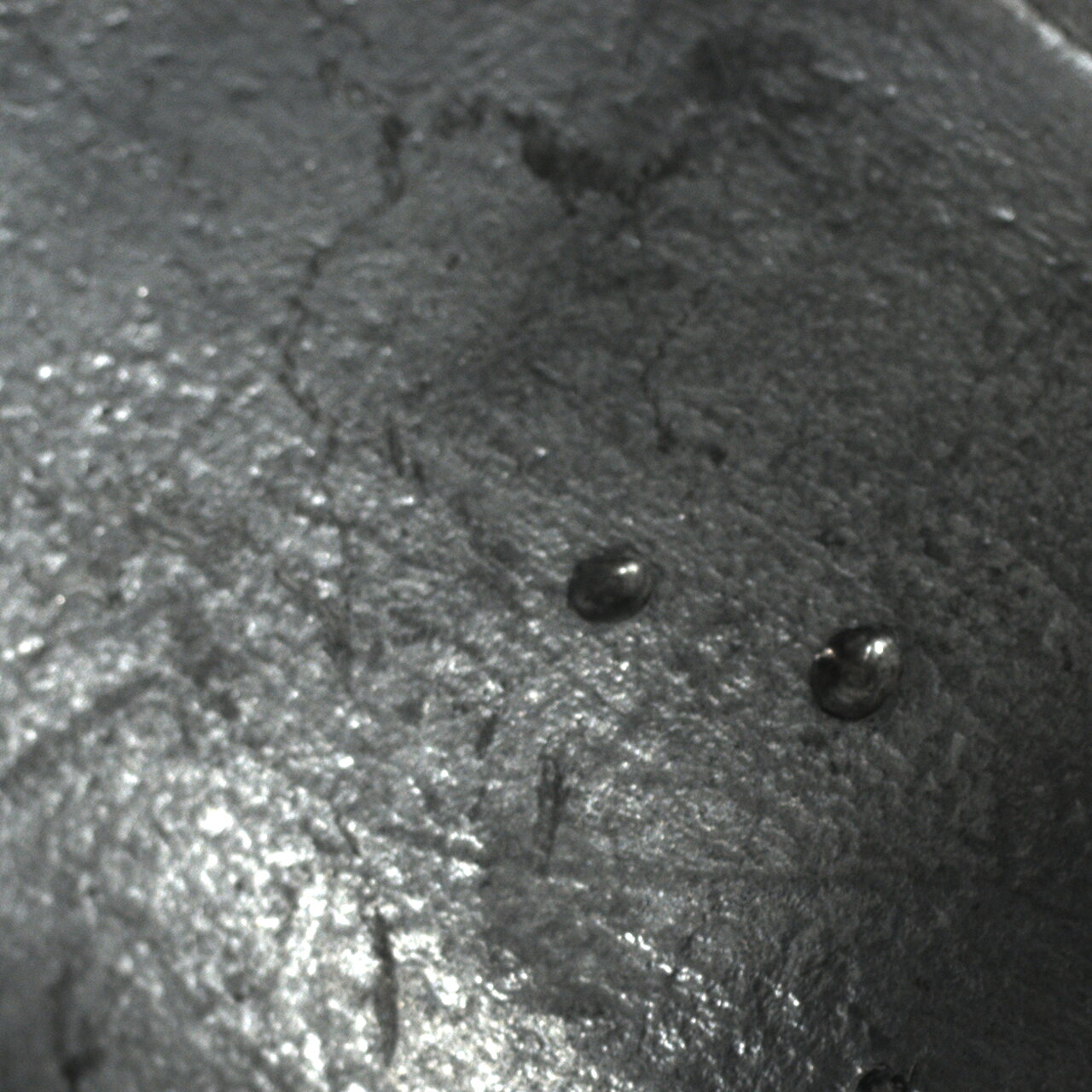

Supplement: Supplemental Information 1 — The CSD-DET dataset was collected from Guangde Hardware Casting Factory and Wuhu Automobile Casting Factory in May 2023. The CSD-DET dataset was used to train and measure the advantages of the DES-YOLO model. This is the filtered partial dataset. [file peerj-cs-10-2224-s001.zip › CastingDefectsDataSet/data/Bh_321.jpg]

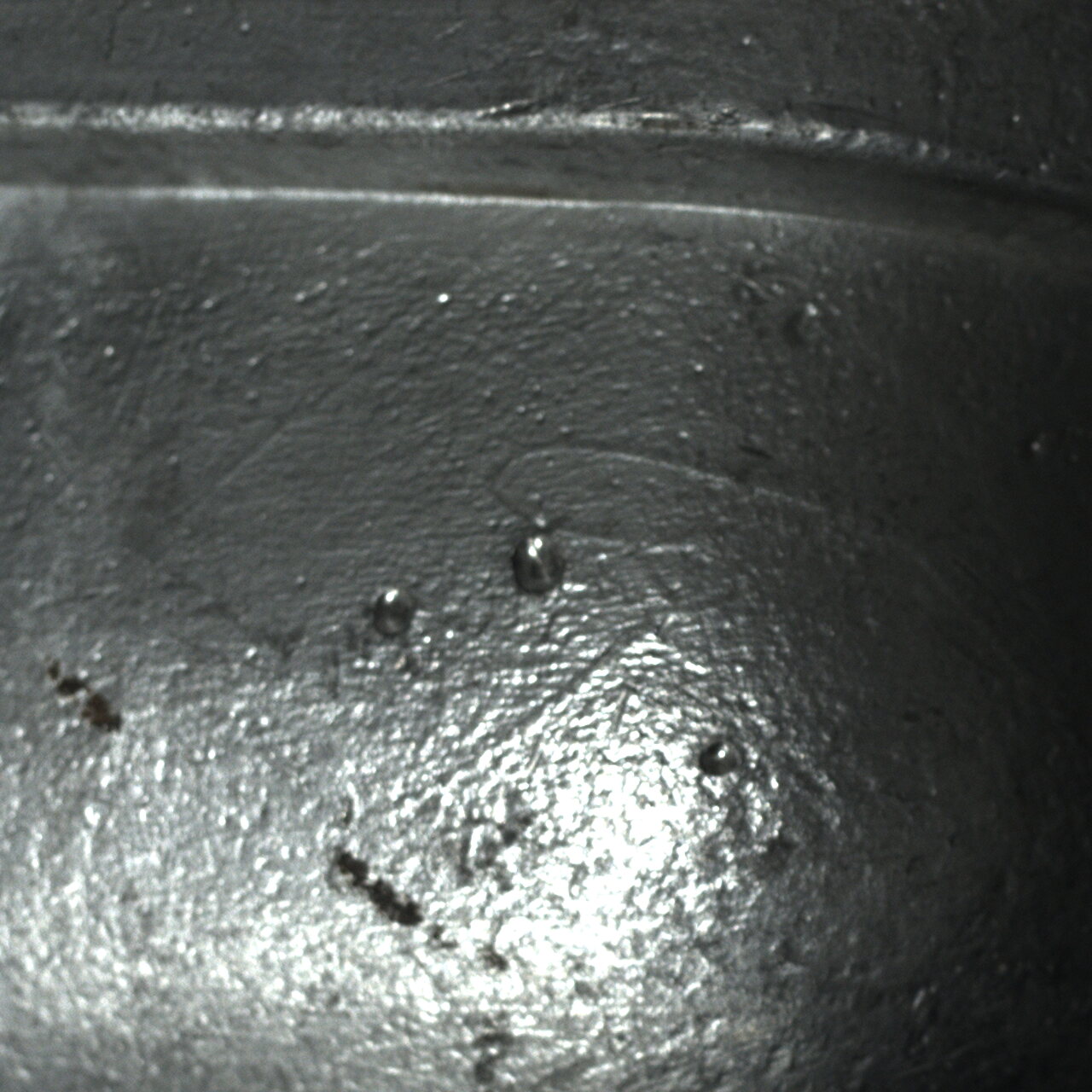

Supplement: Supplemental Information 1 — The CSD-DET dataset was collected from Guangde Hardware Casting Factory and Wuhu Automobile Casting Factory in May 2023. The CSD-DET dataset was used to train and measure the advantages of the DES-YOLO model. This is the filtered partial dataset. [file peerj-cs-10-2224-s001.zip › CastingDefectsDataSet/data/Bh_397.jpg]

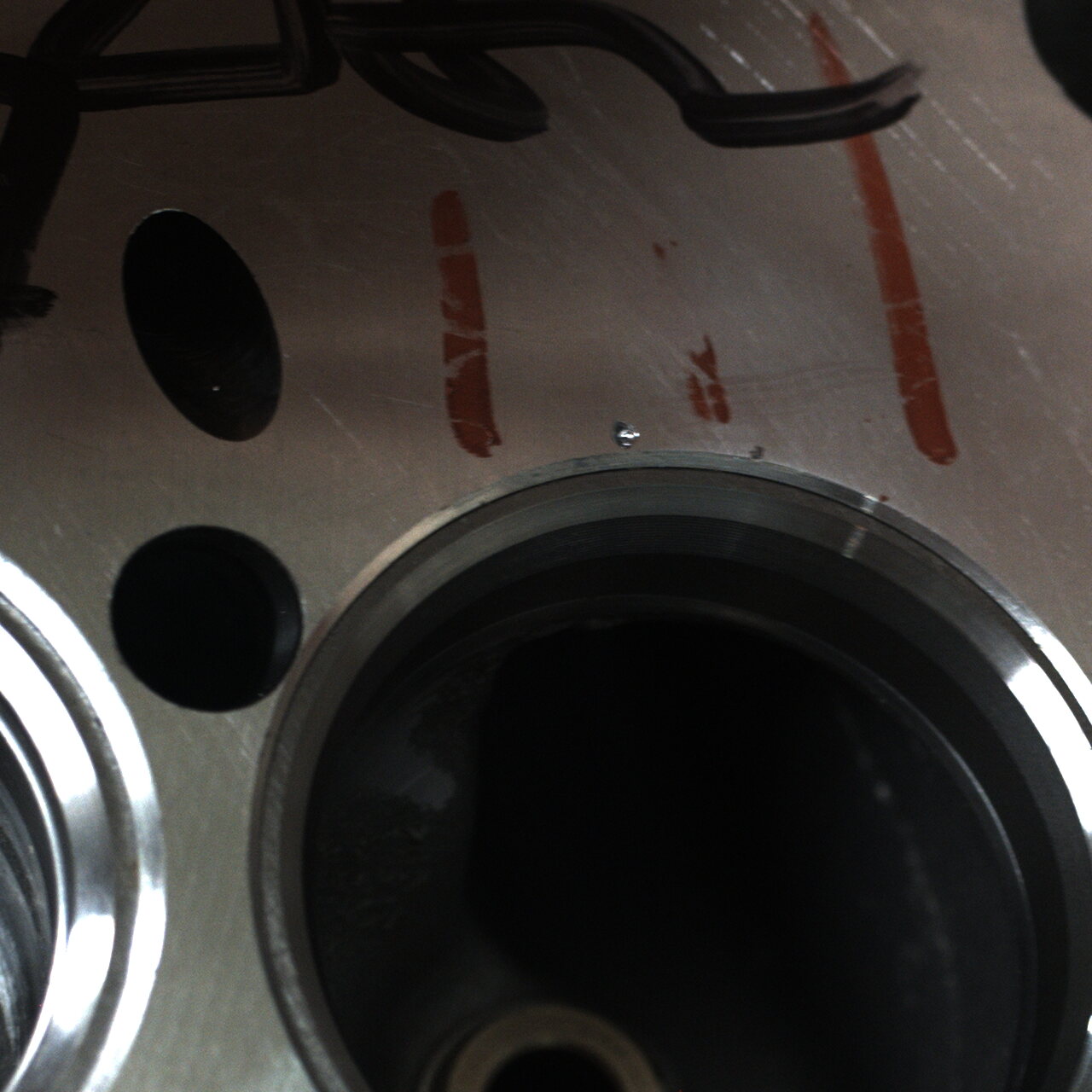

Supplement: Supplemental Information 1 — The CSD-DET dataset was collected from Guangde Hardware Casting Factory and Wuhu Automobile Casting Factory in May 2023. The CSD-DET dataset was used to train and measure the advantages of the DES-YOLO model. This is the filtered partial dataset. [file peerj-cs-10-2224-s001.zip › CastingDefectsDataSet/data/Bh_5.jpg]

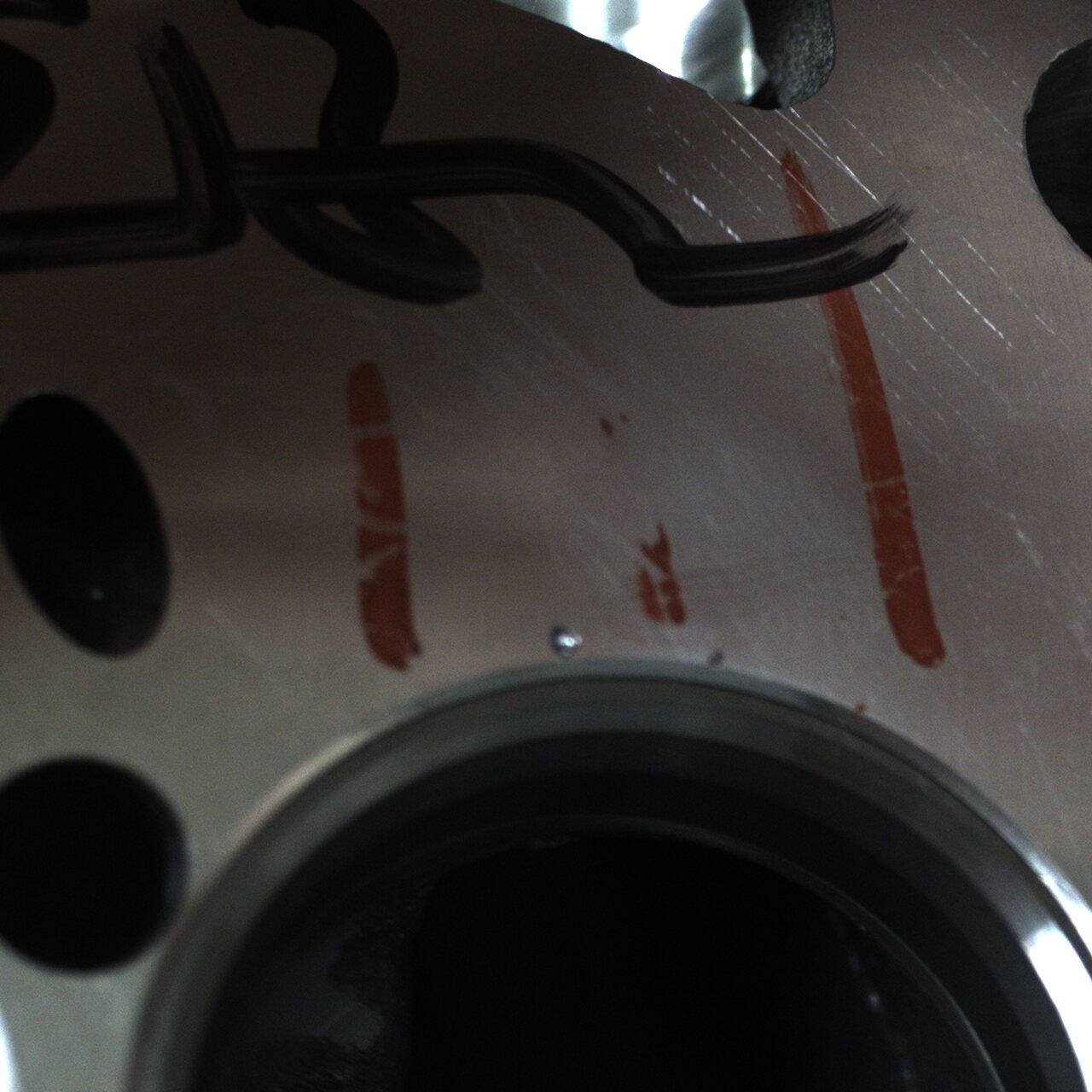

Supplement: Supplemental Information 1 — The CSD-DET dataset was collected from Guangde Hardware Casting Factory and Wuhu Automobile Casting Factory in May 2023. The CSD-DET dataset was used to train and measure the advantages of the DES-YOLO model. This is the filtered partial dataset. [file peerj-cs-10-2224-s001.zip › CastingDefectsDataSet/data/Bh_9.jpg]

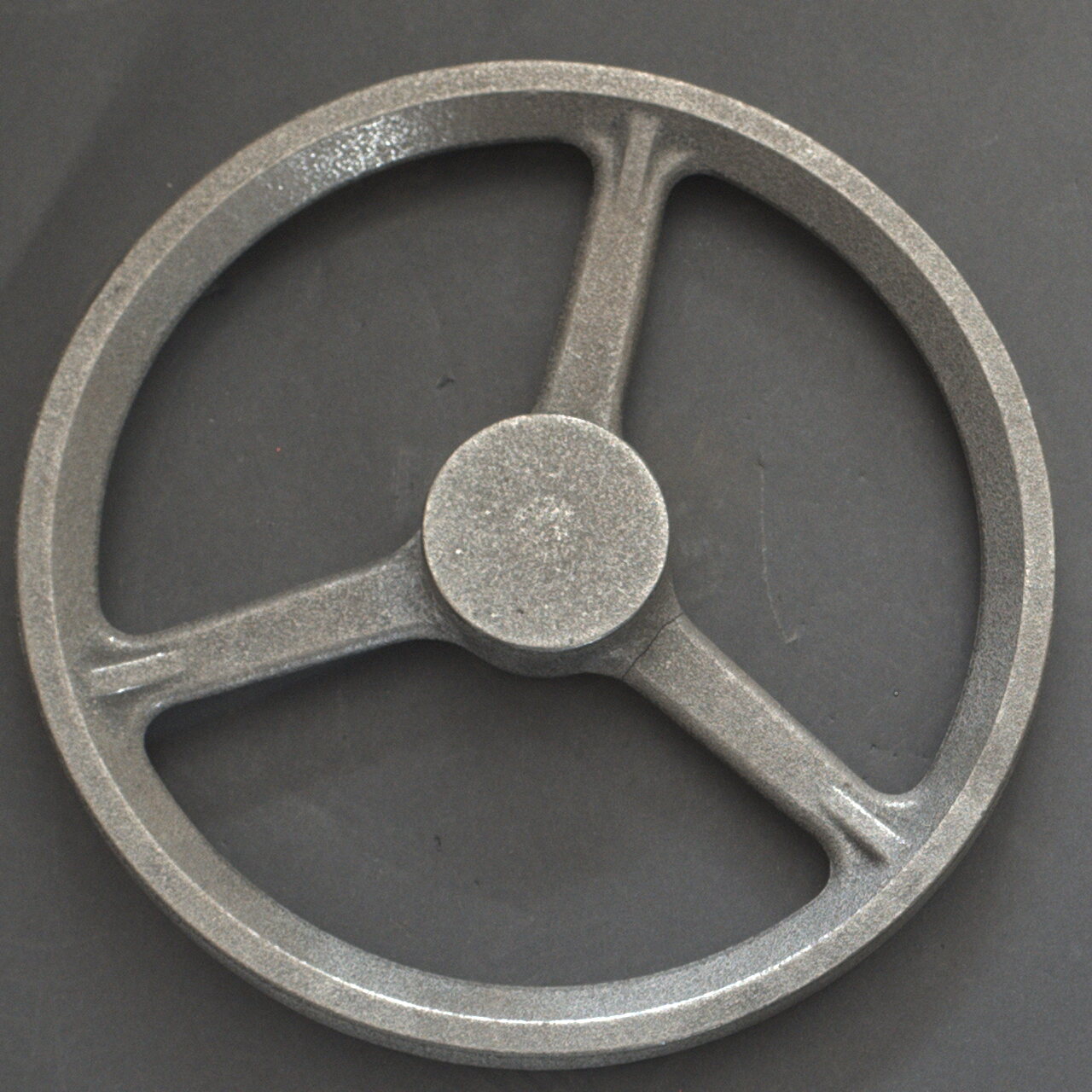

Supplement: Supplemental Information 1 — The CSD-DET dataset was collected from Guangde Hardware Casting Factory and Wuhu Automobile Casting Factory in May 2023. The CSD-DET dataset was used to train and measure the advantages of the DES-YOLO model. This is the filtered partial dataset. [file peerj-cs-10-2224-s001.zip › CastingDefectsDataSet/data/Fr_176.jpg]

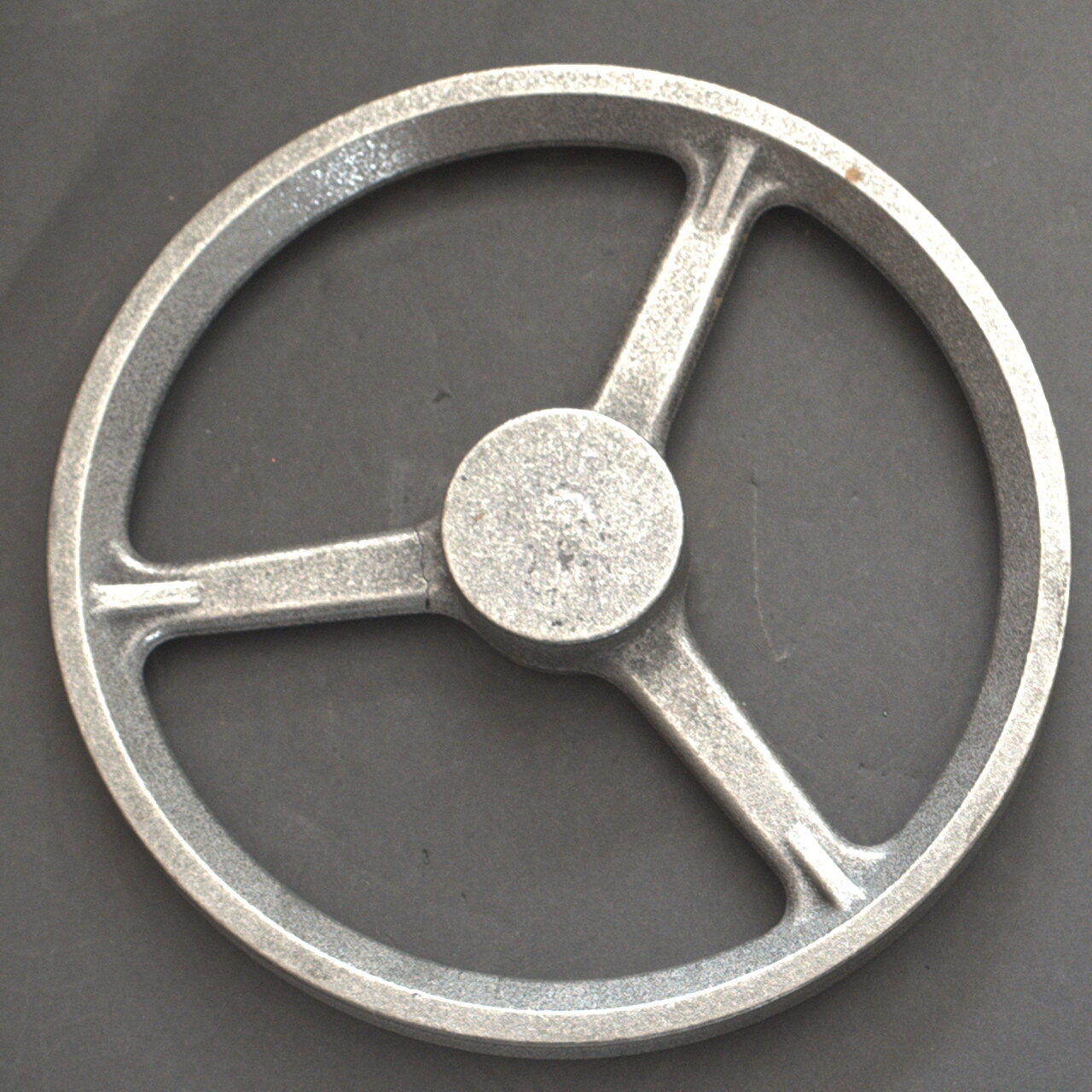

Supplement: Supplemental Information 1 — The CSD-DET dataset was collected from Guangde Hardware Casting Factory and Wuhu Automobile Casting Factory in May 2023. The CSD-DET dataset was used to train and measure the advantages of the DES-YOLO model. This is the filtered partial dataset. [file peerj-cs-10-2224-s001.zip › CastingDefectsDataSet/data/Fr_184.jpg]

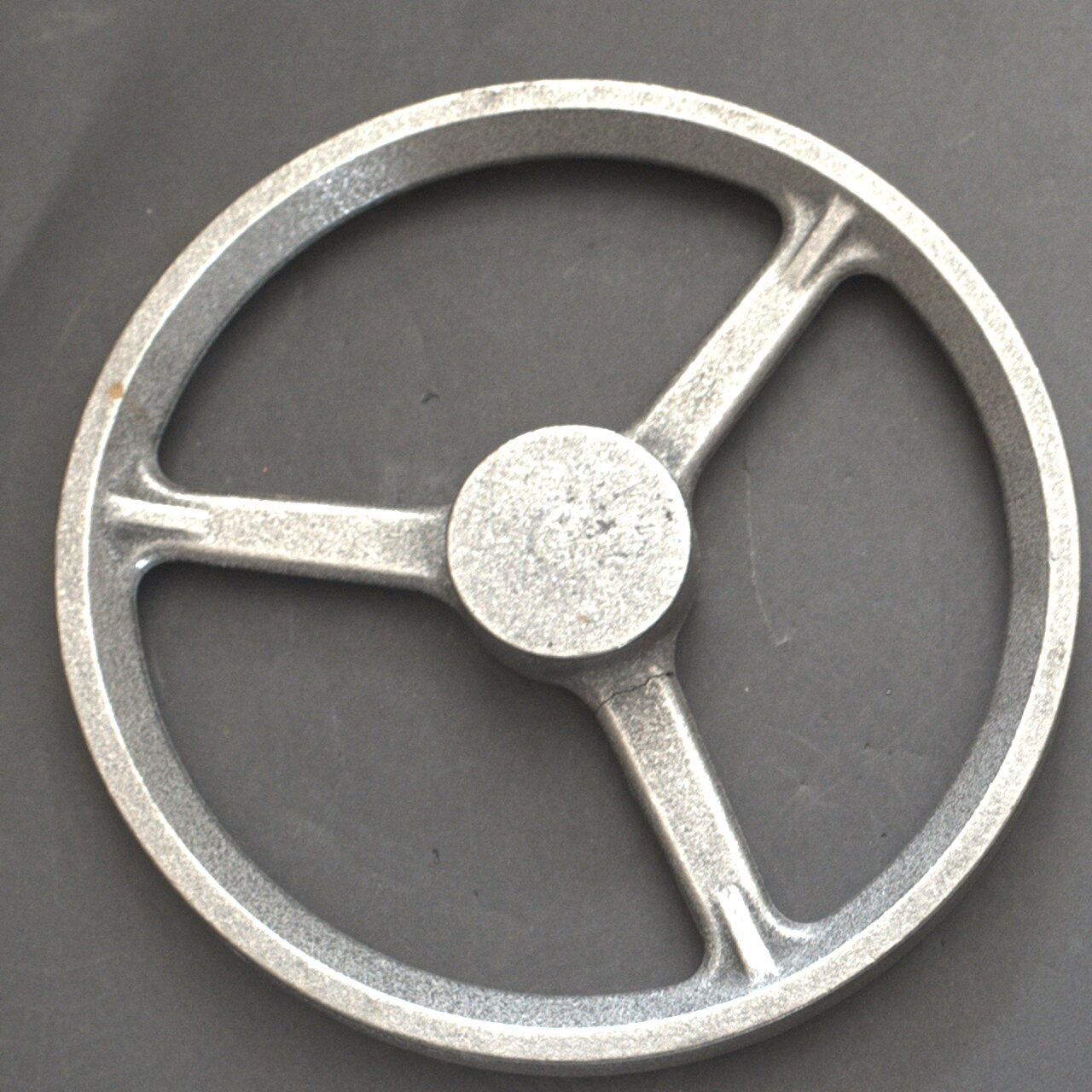

Supplement: Supplemental Information 1 — The CSD-DET dataset was collected from Guangde Hardware Casting Factory and Wuhu Automobile Casting Factory in May 2023. The CSD-DET dataset was used to train and measure the advantages of the DES-YOLO model. This is the filtered partial dataset. [file peerj-cs-10-2224-s001.zip › CastingDefectsDataSet/data/Fr_188.jpg]

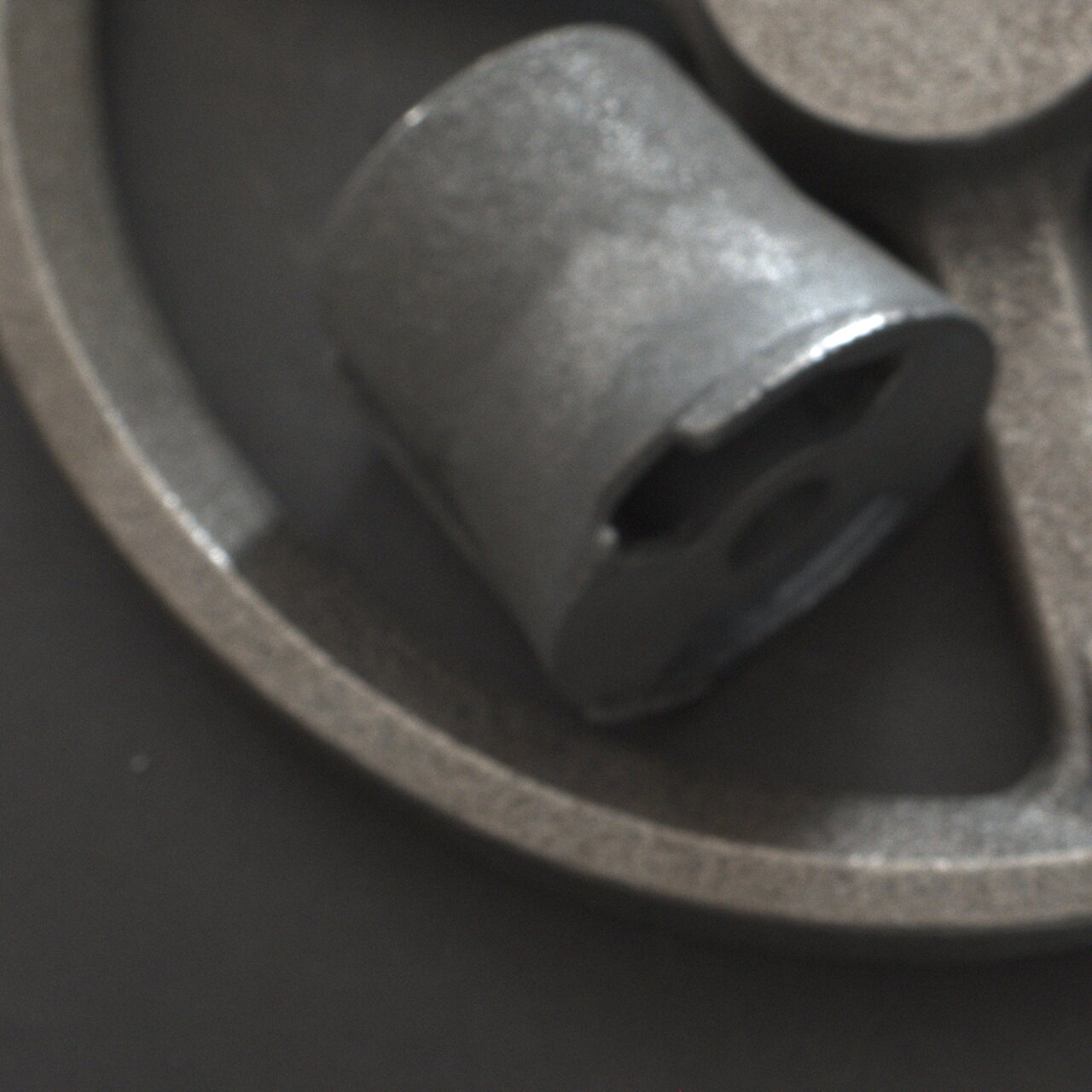

Supplement: Supplemental Information 1 — The CSD-DET dataset was collected from Guangde Hardware Casting Factory and Wuhu Automobile Casting Factory in May 2023. The CSD-DET dataset was used to train and measure the advantages of the DES-YOLO model. This is the filtered partial dataset. [file peerj-cs-10-2224-s001.zip › CastingDefectsDataSet/data/Fr_300.jpg]

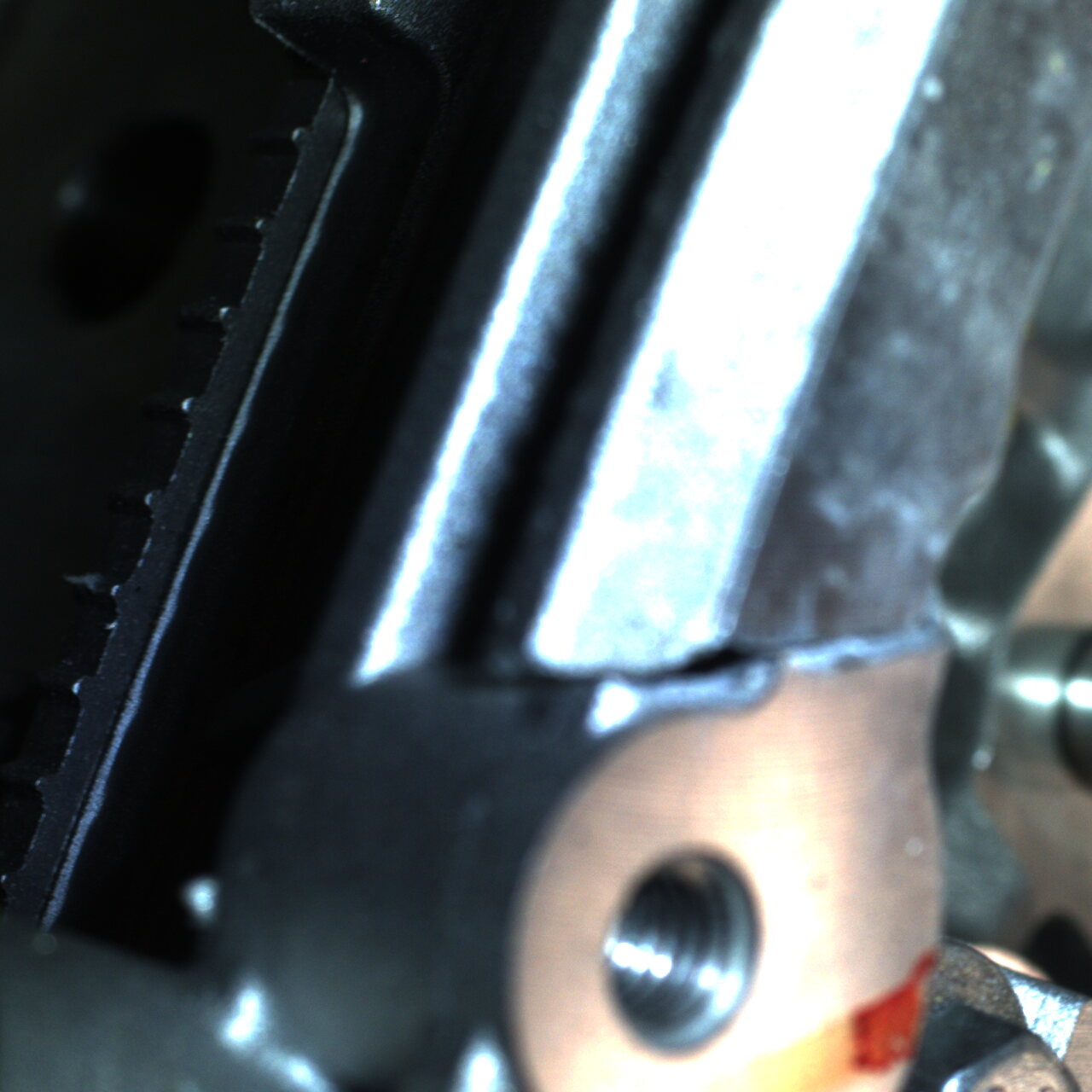

Supplement: Supplemental Information 1 — The CSD-DET dataset was collected from Guangde Hardware Casting Factory and Wuhu Automobile Casting Factory in May 2023. The CSD-DET dataset was used to train and measure the advantages of the DES-YOLO model. This is the filtered partial dataset. [file peerj-cs-10-2224-s001.zip › CastingDefectsDataSet/data/Fr_320.jpg]

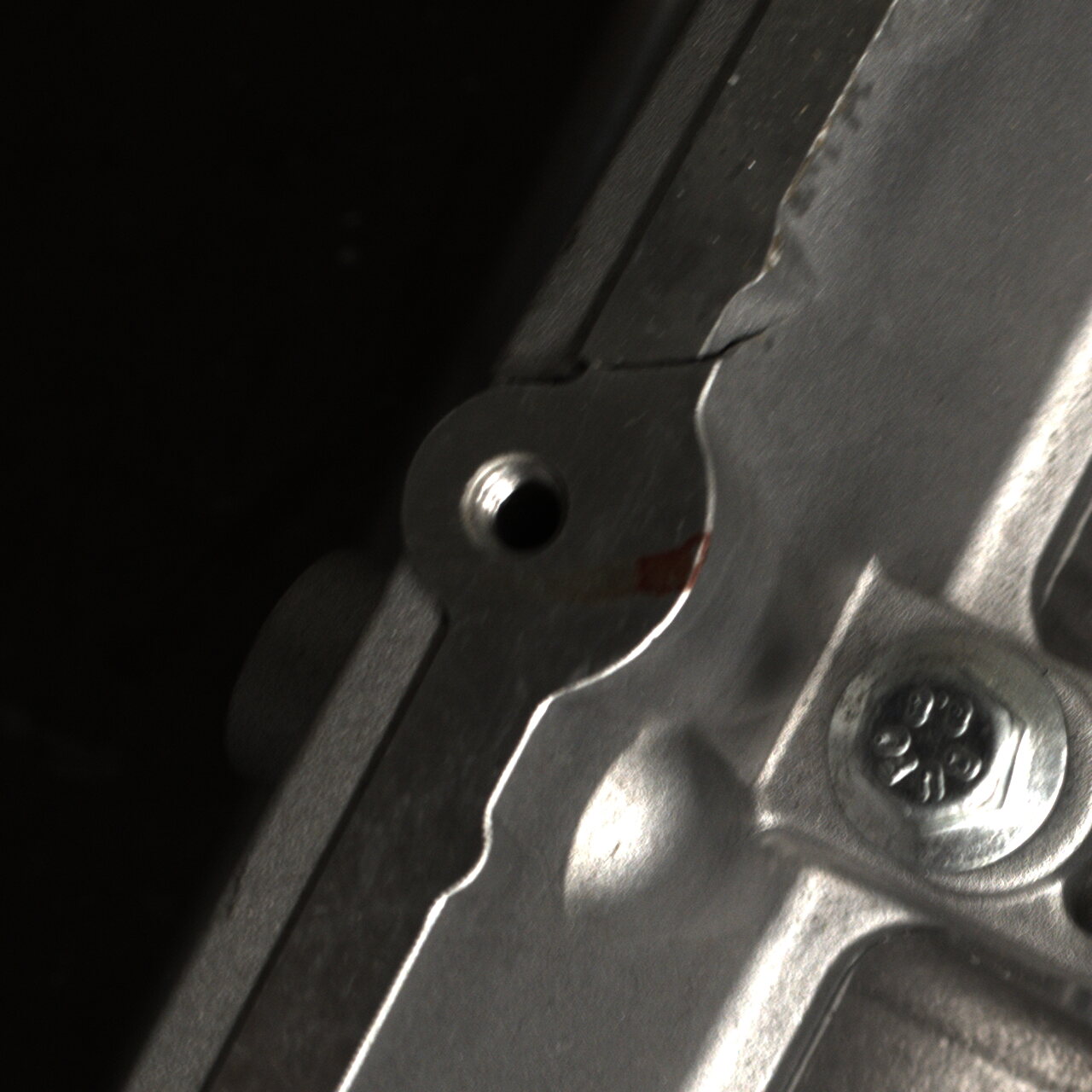

Supplement: Supplemental Information 1 — The CSD-DET dataset was collected from Guangde Hardware Casting Factory and Wuhu Automobile Casting Factory in May 2023. The CSD-DET dataset was used to train and measure the advantages of the DES-YOLO model. This is the filtered partial dataset. [file peerj-cs-10-2224-s001.zip › CastingDefectsDataSet/data/Fr_368.jpg]

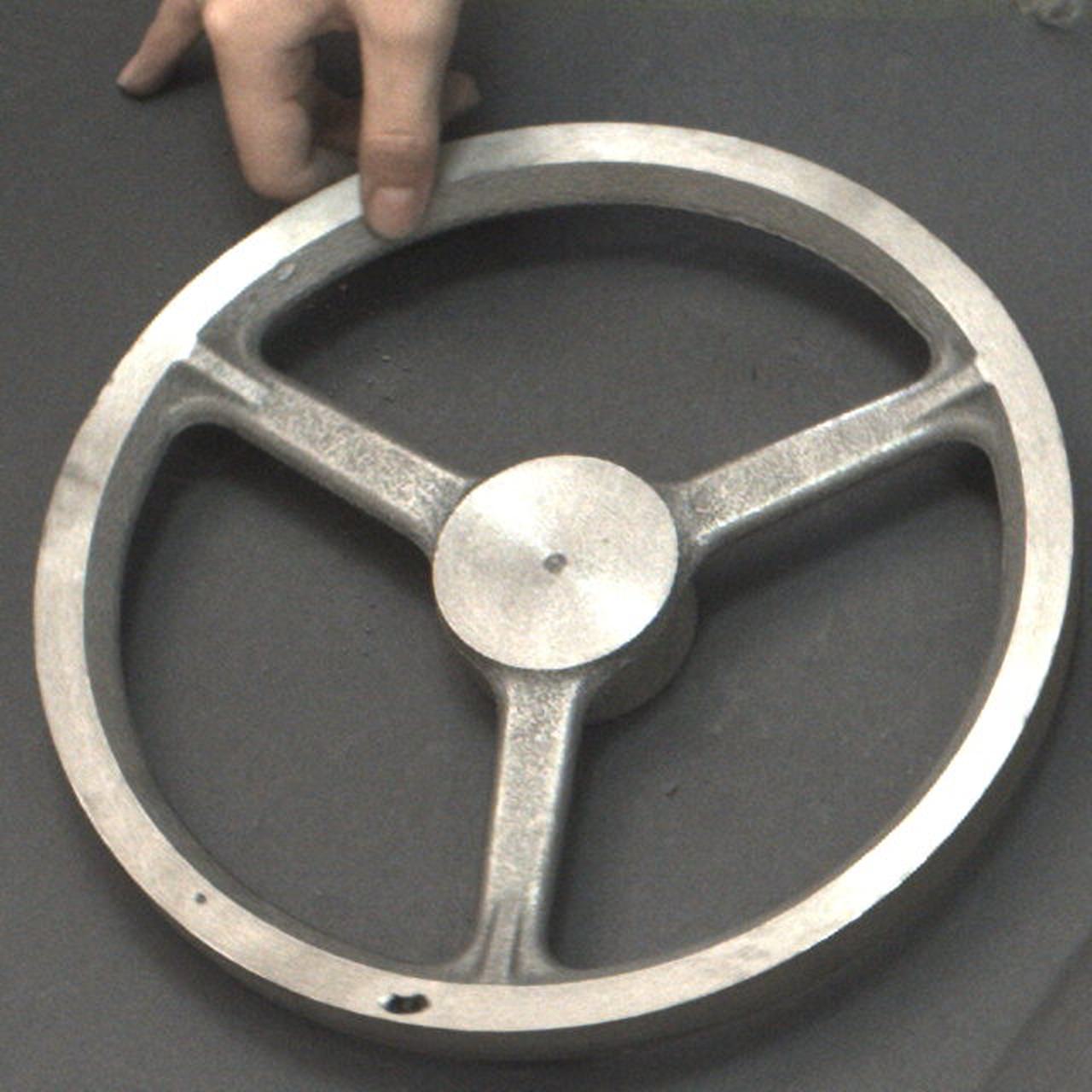

Supplement: Supplemental Information 1 — The CSD-DET dataset was collected from Guangde Hardware Casting Factory and Wuhu Automobile Casting Factory in May 2023. The CSD-DET dataset was used to train and measure the advantages of the DES-YOLO model. This is the filtered partial dataset. [file peerj-cs-10-2224-s001.zip › CastingDefectsDataSet/data/Fr_464.jpg]

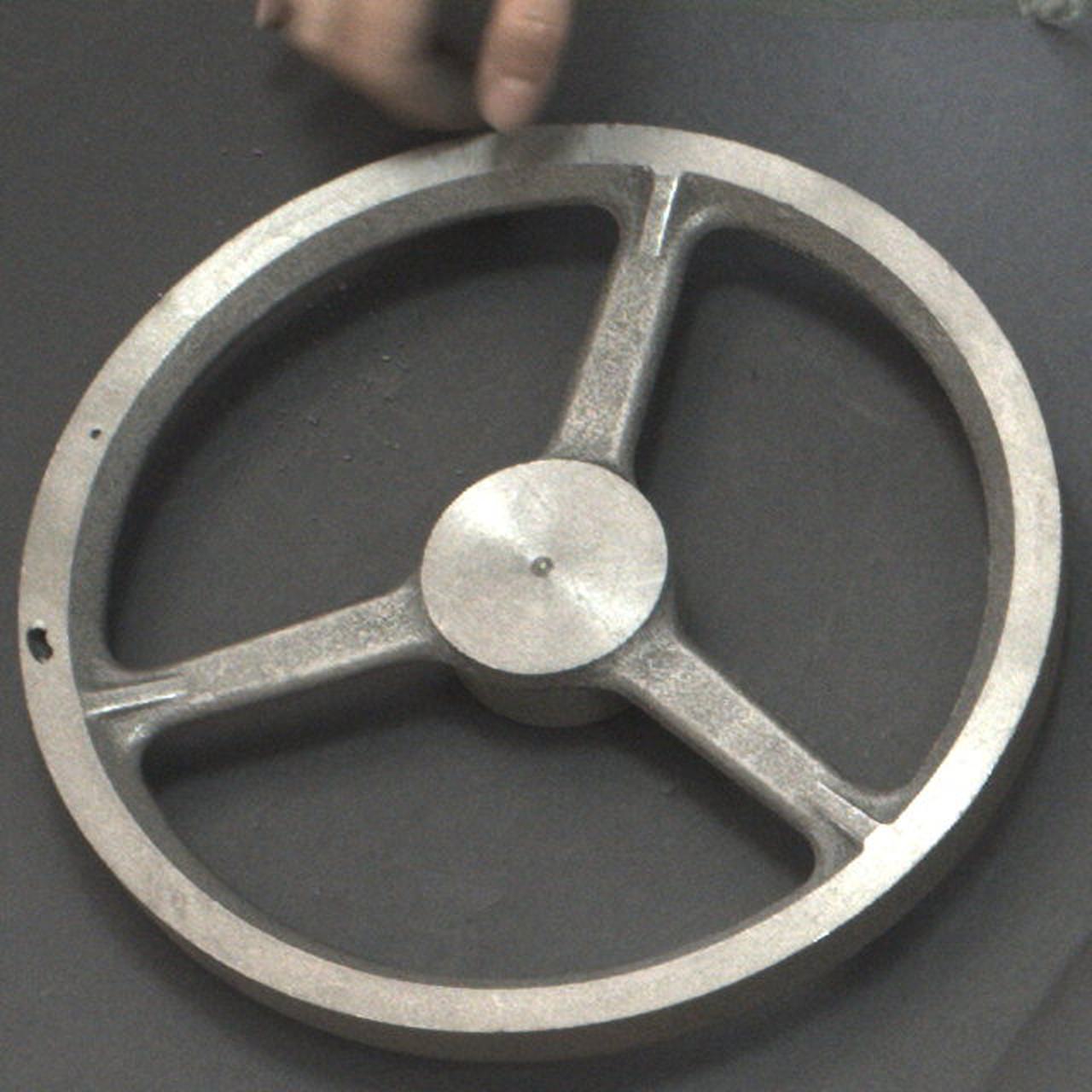

Supplement: Supplemental Information 1 — The CSD-DET dataset was collected from Guangde Hardware Casting Factory and Wuhu Automobile Casting Factory in May 2023. The CSD-DET dataset was used to train and measure the advantages of the DES-YOLO model. This is the filtered partial dataset. [file peerj-cs-10-2224-s001.zip › CastingDefectsDataSet/data/Fr_468.jpg]

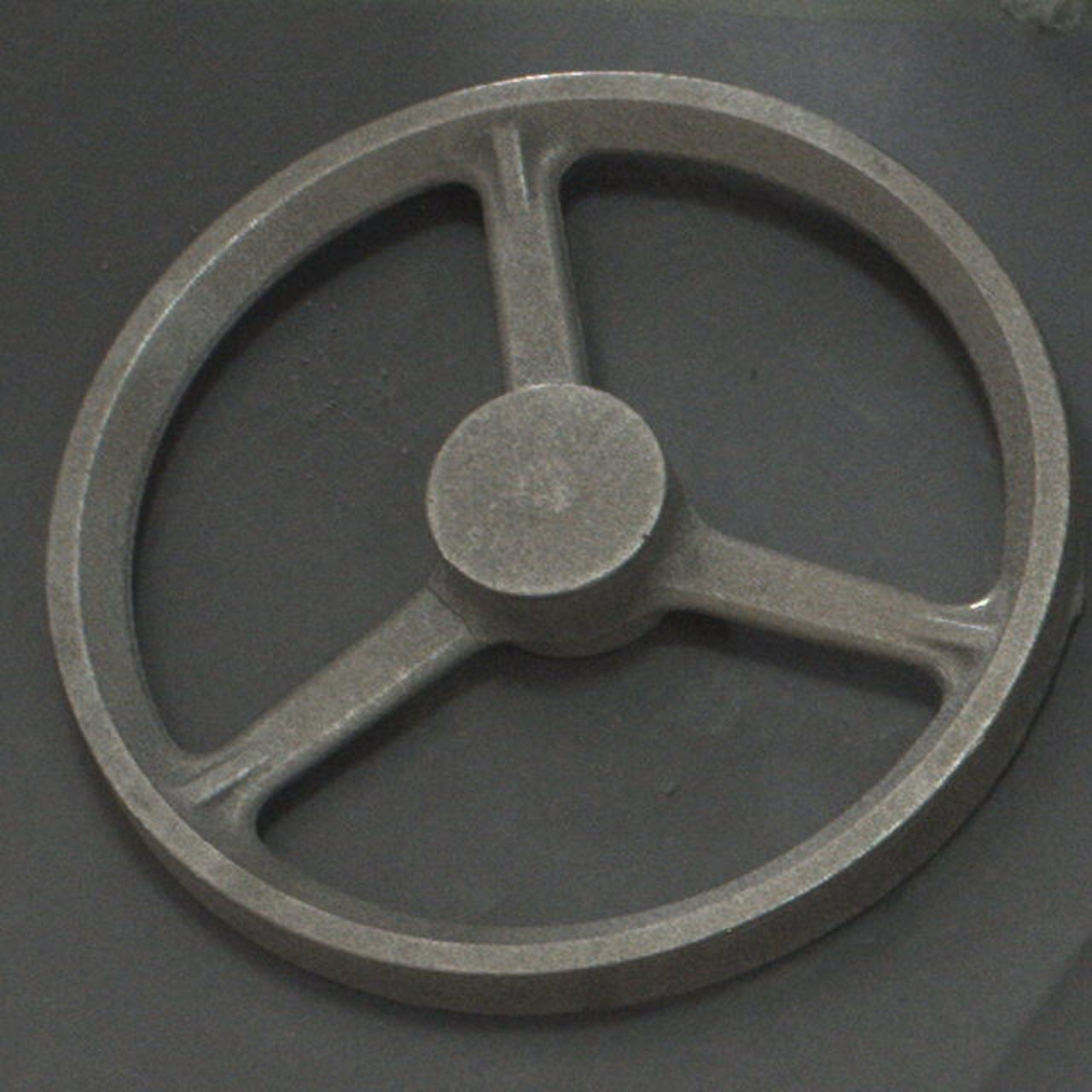

Supplement: Supplemental Information 1 — The CSD-DET dataset was collected from Guangde Hardware Casting Factory and Wuhu Automobile Casting Factory in May 2023. The CSD-DET dataset was used to train and measure the advantages of the DES-YOLO model. This is the filtered partial dataset. [file peerj-cs-10-2224-s001.zip › CastingDefectsDataSet/data/Fr_496.jpg]

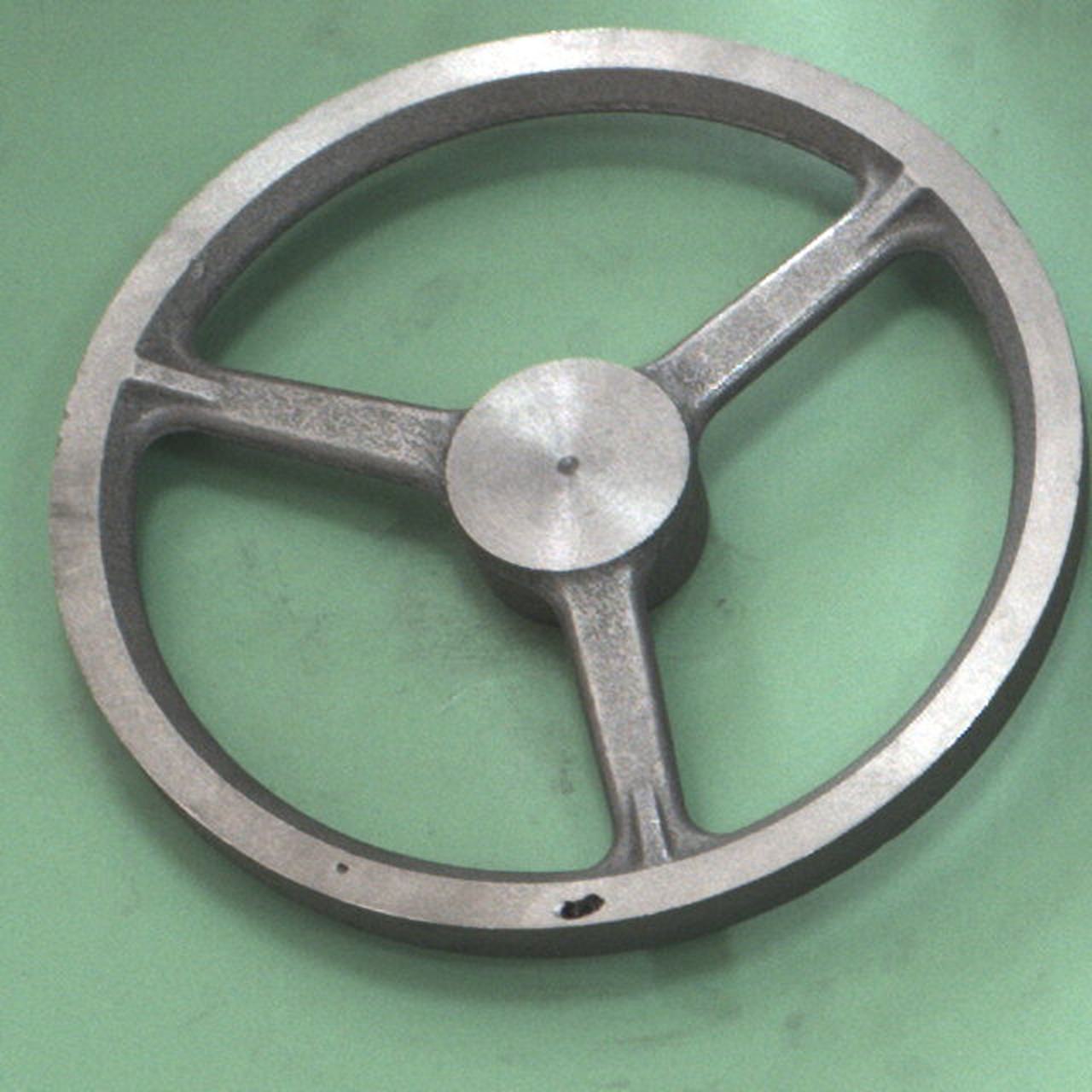

Supplement: Supplemental Information 1 — The CSD-DET dataset was collected from Guangde Hardware Casting Factory and Wuhu Automobile Casting Factory in May 2023. The CSD-DET dataset was used to train and measure the advantages of the DES-YOLO model. This is the filtered partial dataset. [file peerj-cs-10-2224-s001.zip › CastingDefectsDataSet/data/Fr_584.jpg]

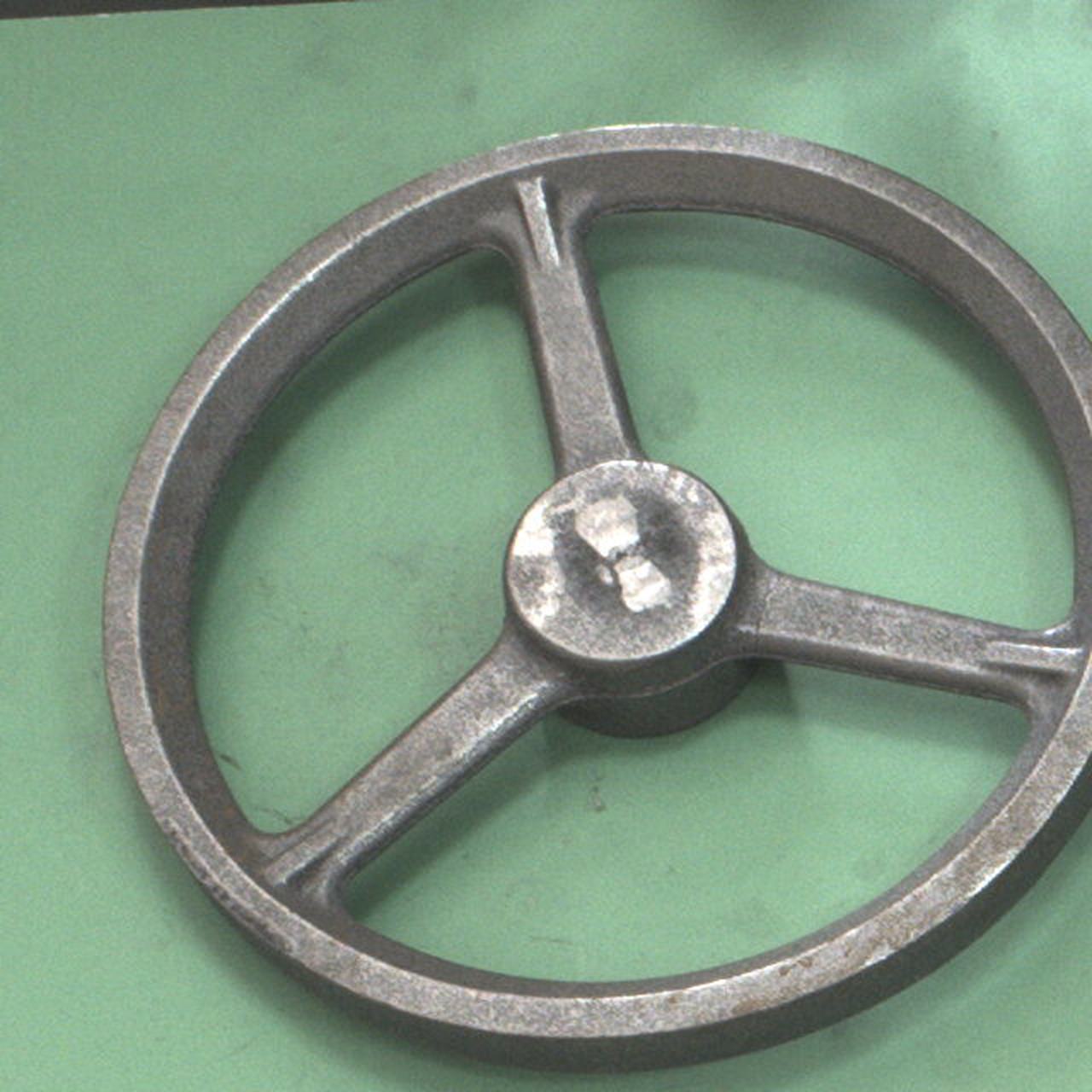

Supplement: Supplemental Information 1 — The CSD-DET dataset was collected from Guangde Hardware Casting Factory and Wuhu Automobile Casting Factory in May 2023. The CSD-DET dataset was used to train and measure the advantages of the DES-YOLO model. This is the filtered partial dataset. [file peerj-cs-10-2224-s001.zip › CastingDefectsDataSet/data/Fr_588.jpg]

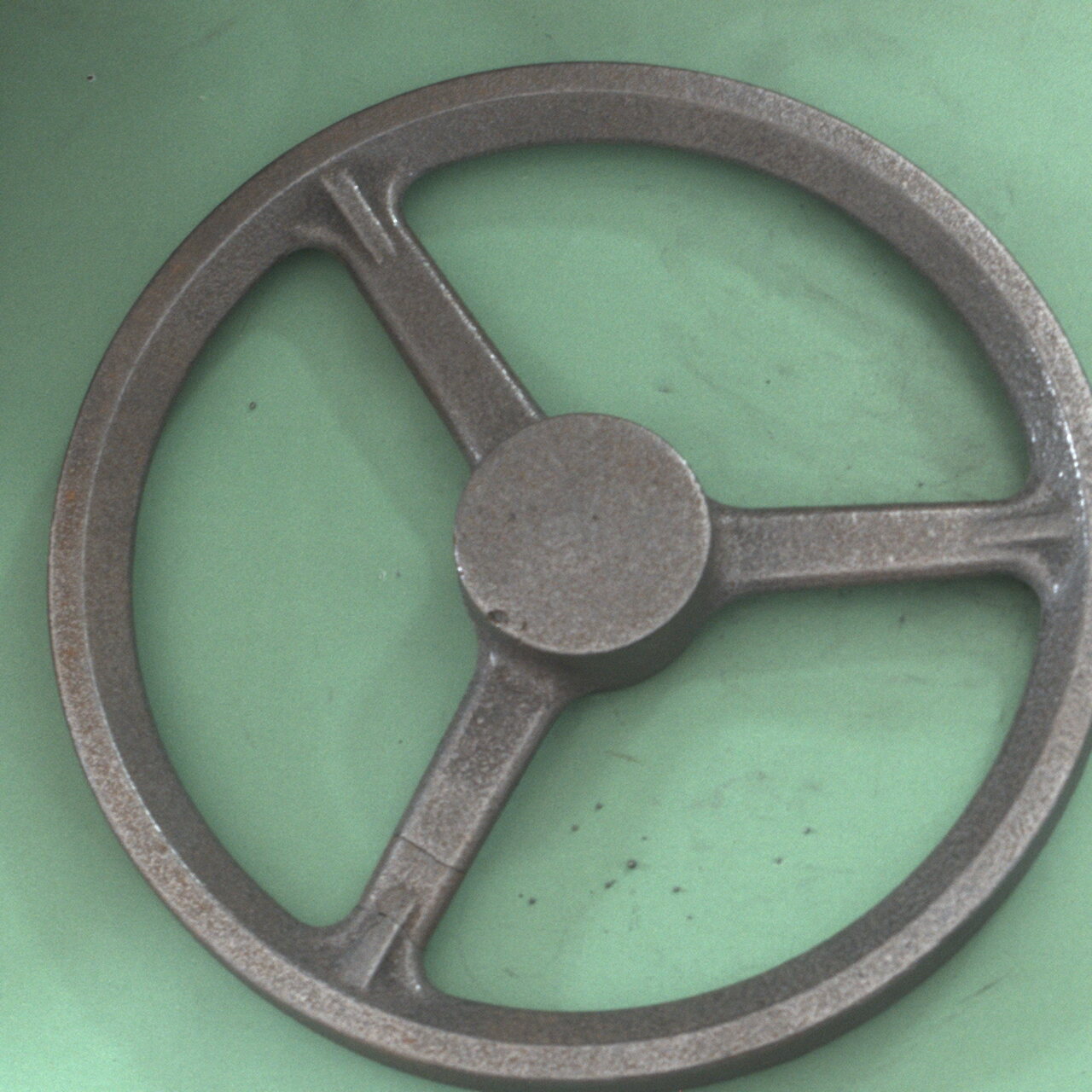

Supplement: Supplemental Information 1 — The CSD-DET dataset was collected from Guangde Hardware Casting Factory and Wuhu Automobile Casting Factory in May 2023. The CSD-DET dataset was used to train and measure the advantages of the DES-YOLO model. This is the filtered partial dataset. [file peerj-cs-10-2224-s001.zip › CastingDefectsDataSet/data/Fr_64.jpg]

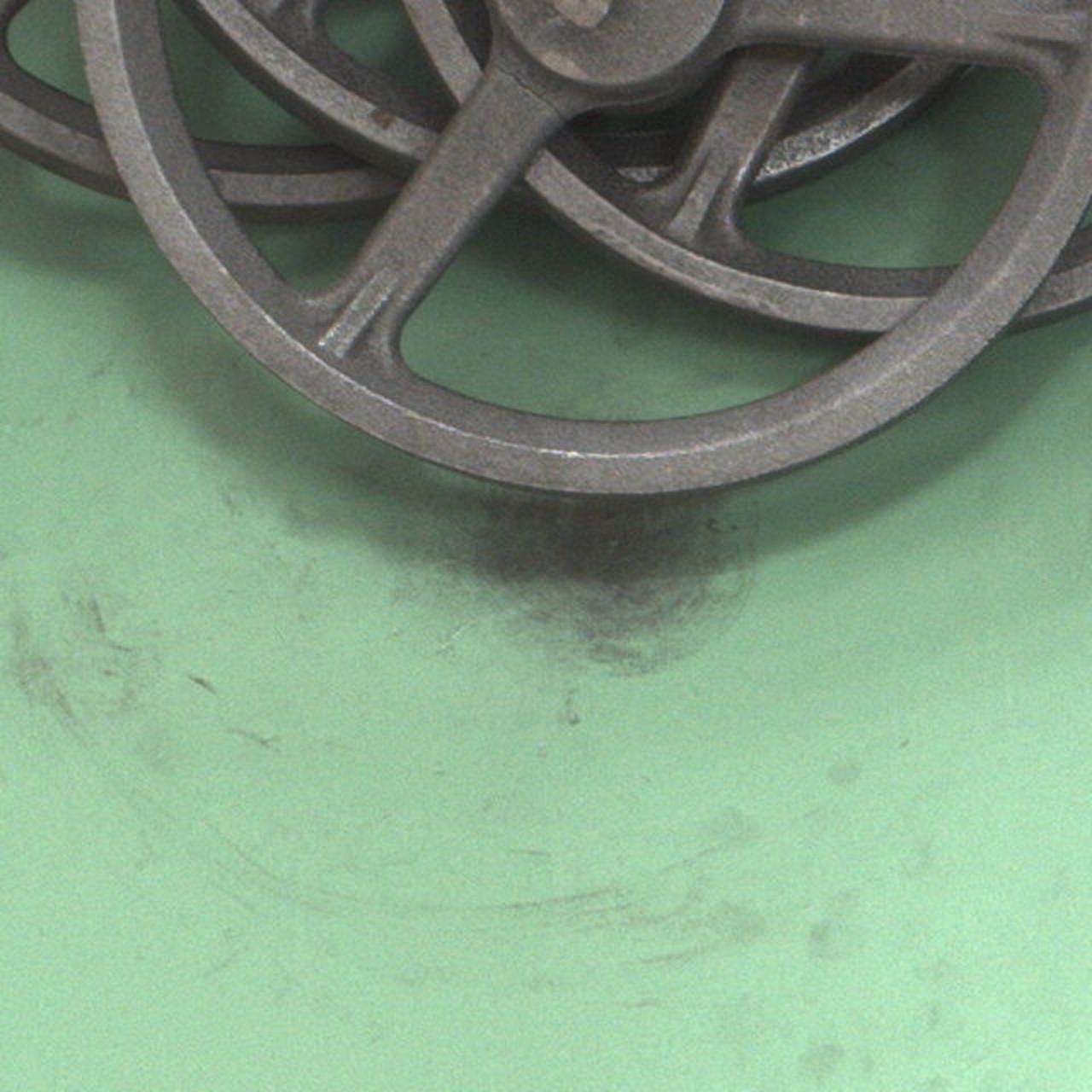

Supplement: Supplemental Information 1 — The CSD-DET dataset was collected from Guangde Hardware Casting Factory and Wuhu Automobile Casting Factory in May 2023. The CSD-DET dataset was used to train and measure the advantages of the DES-YOLO model. This is the filtered partial dataset. [file peerj-cs-10-2224-s001.zip › CastingDefectsDataSet/data/Fr_644.jpg]

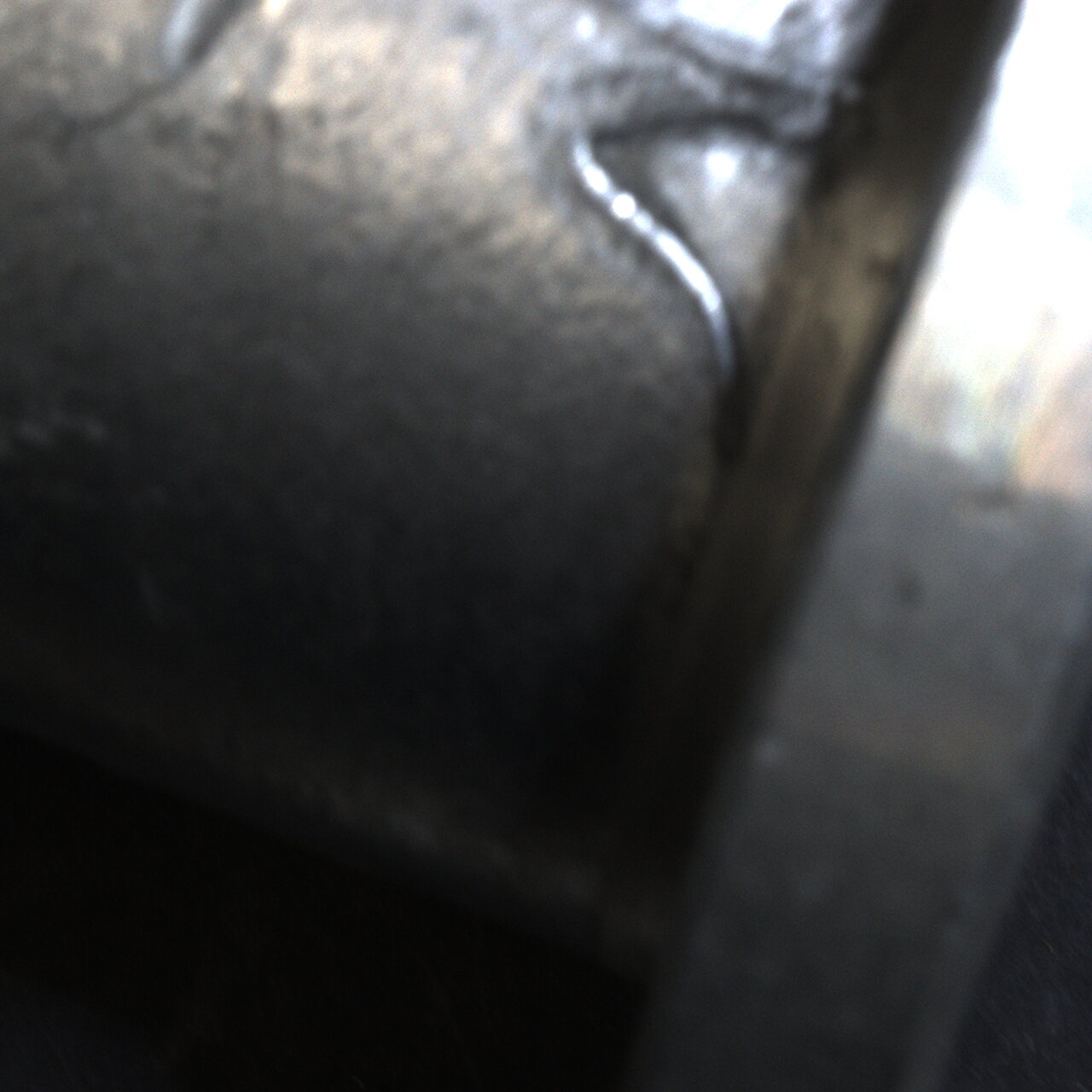

Supplement: Supplemental Information 1 — The CSD-DET dataset was collected from Guangde Hardware Casting Factory and Wuhu Automobile Casting Factory in May 2023. The CSD-DET dataset was used to train and measure the advantages of the DES-YOLO model. This is the filtered partial dataset. [file peerj-cs-10-2224-s001.zip › CastingDefectsDataSet/data/Fr_756.jpg]

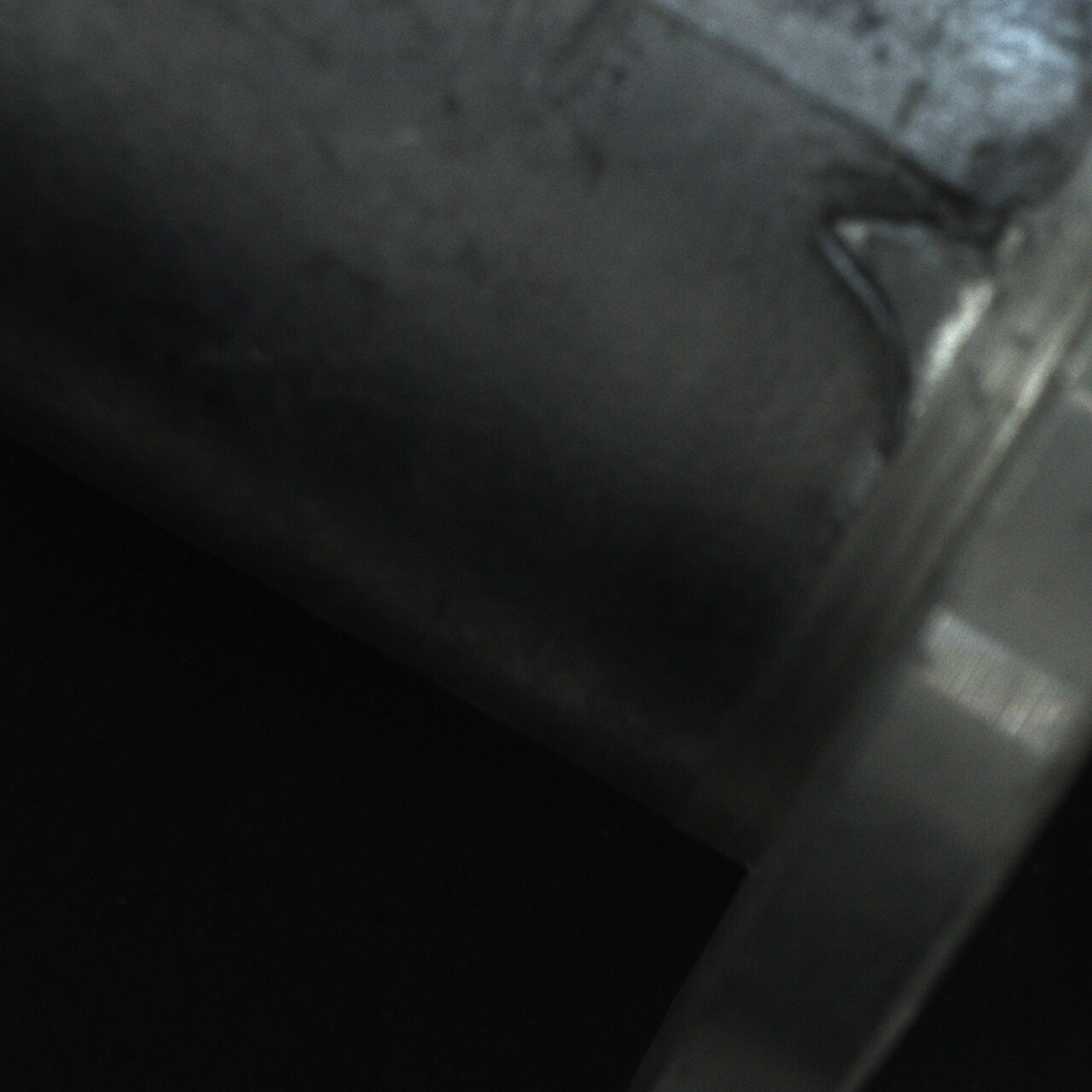

Supplement: Supplemental Information 1 — The CSD-DET dataset was collected from Guangde Hardware Casting Factory and Wuhu Automobile Casting Factory in May 2023. The CSD-DET dataset was used to train and measure the advantages of the DES-YOLO model. This is the filtered partial dataset. [file peerj-cs-10-2224-s001.zip › CastingDefectsDataSet/data/Fr_760.jpg]

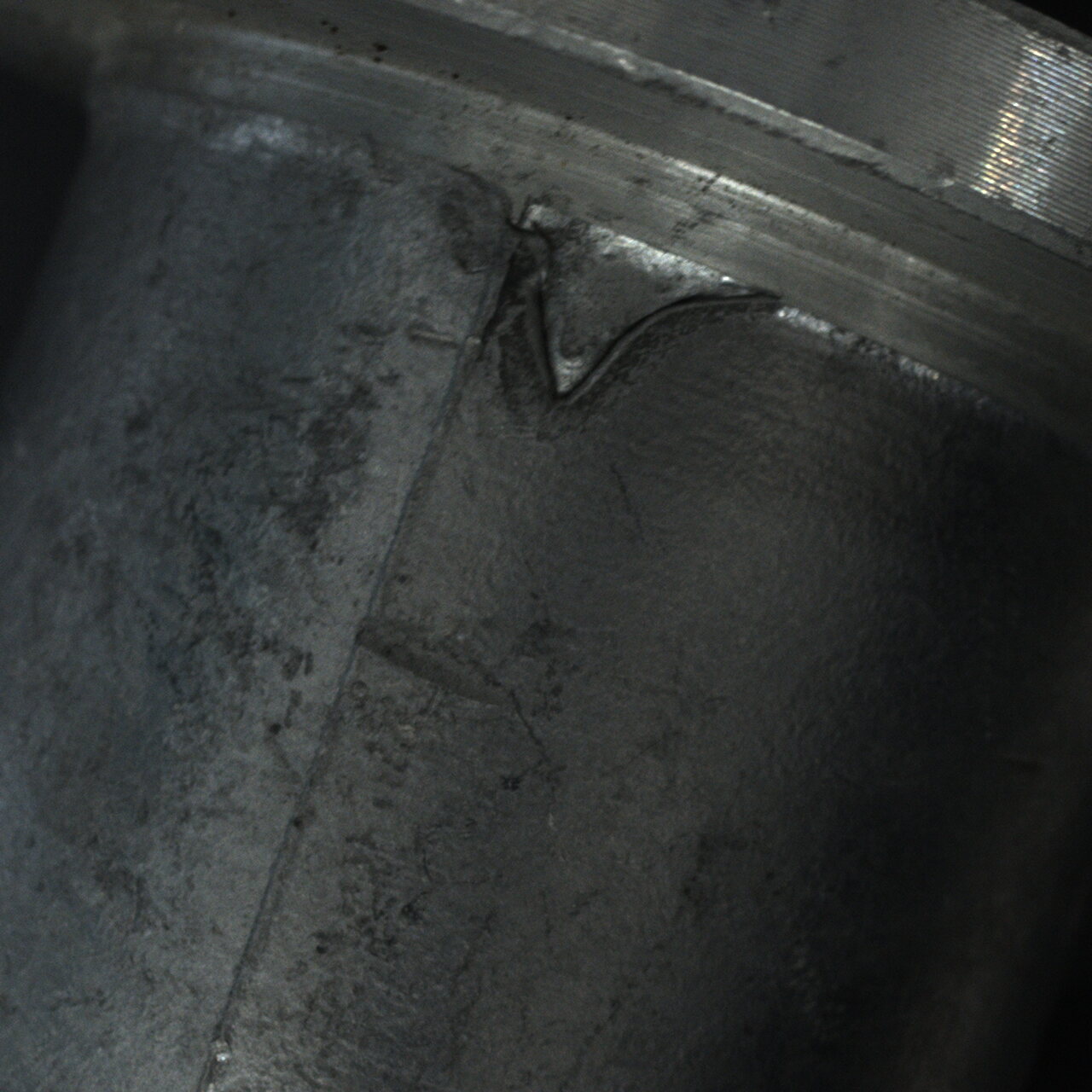

Supplement: Supplemental Information 1 — The CSD-DET dataset was collected from Guangde Hardware Casting Factory and Wuhu Automobile Casting Factory in May 2023. The CSD-DET dataset was used to train and measure the advantages of the DES-YOLO model. This is the filtered partial dataset. [file peerj-cs-10-2224-s001.zip › CastingDefectsDataSet/data/Fr_784.jpg]

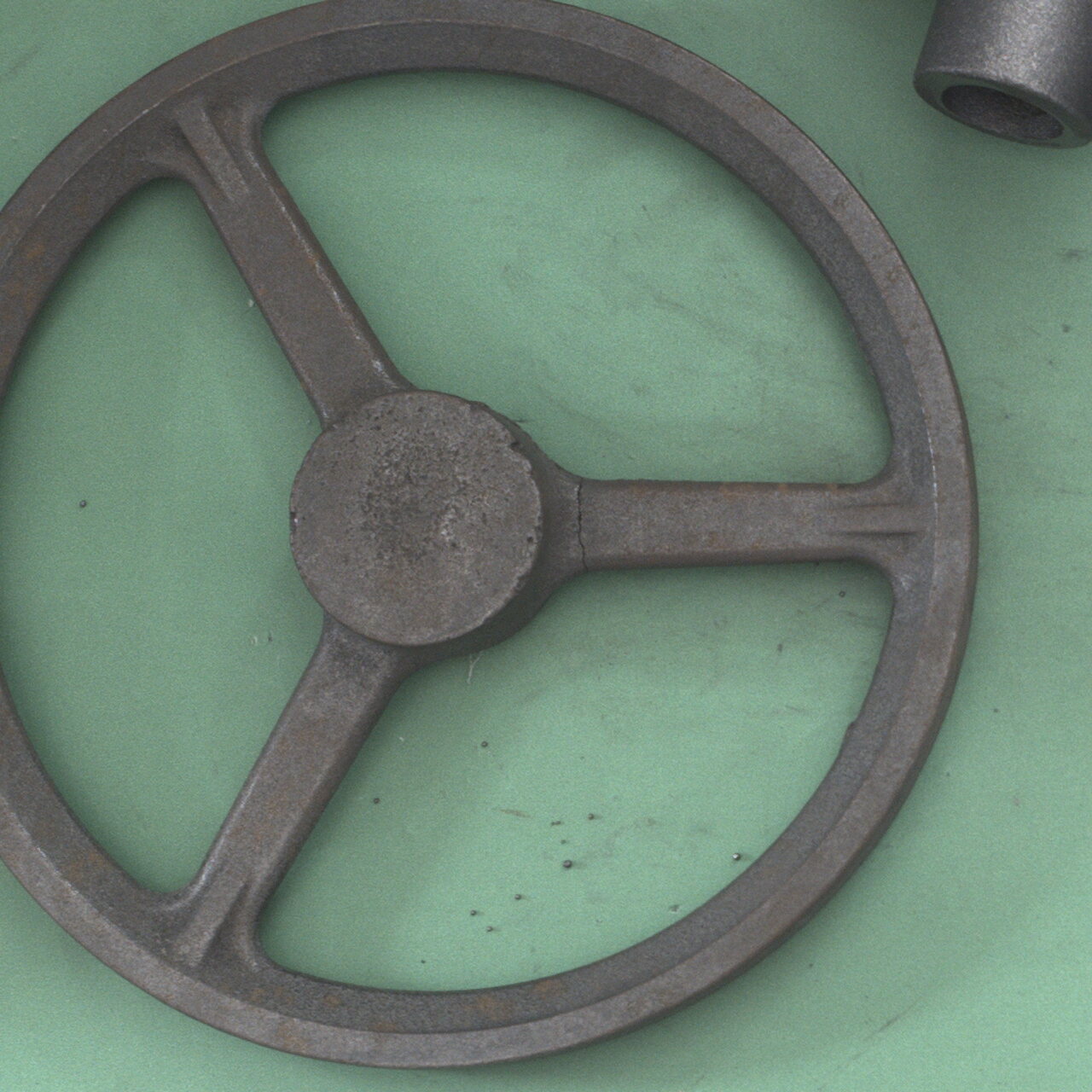

Supplement: Supplemental Information 1 — The CSD-DET dataset was collected from Guangde Hardware Casting Factory and Wuhu Automobile Casting Factory in May 2023. The CSD-DET dataset was used to train and measure the advantages of the DES-YOLO model. This is the filtered partial dataset. [file peerj-cs-10-2224-s001.zip › CastingDefectsDataSet/data/Fr_8.jpg]

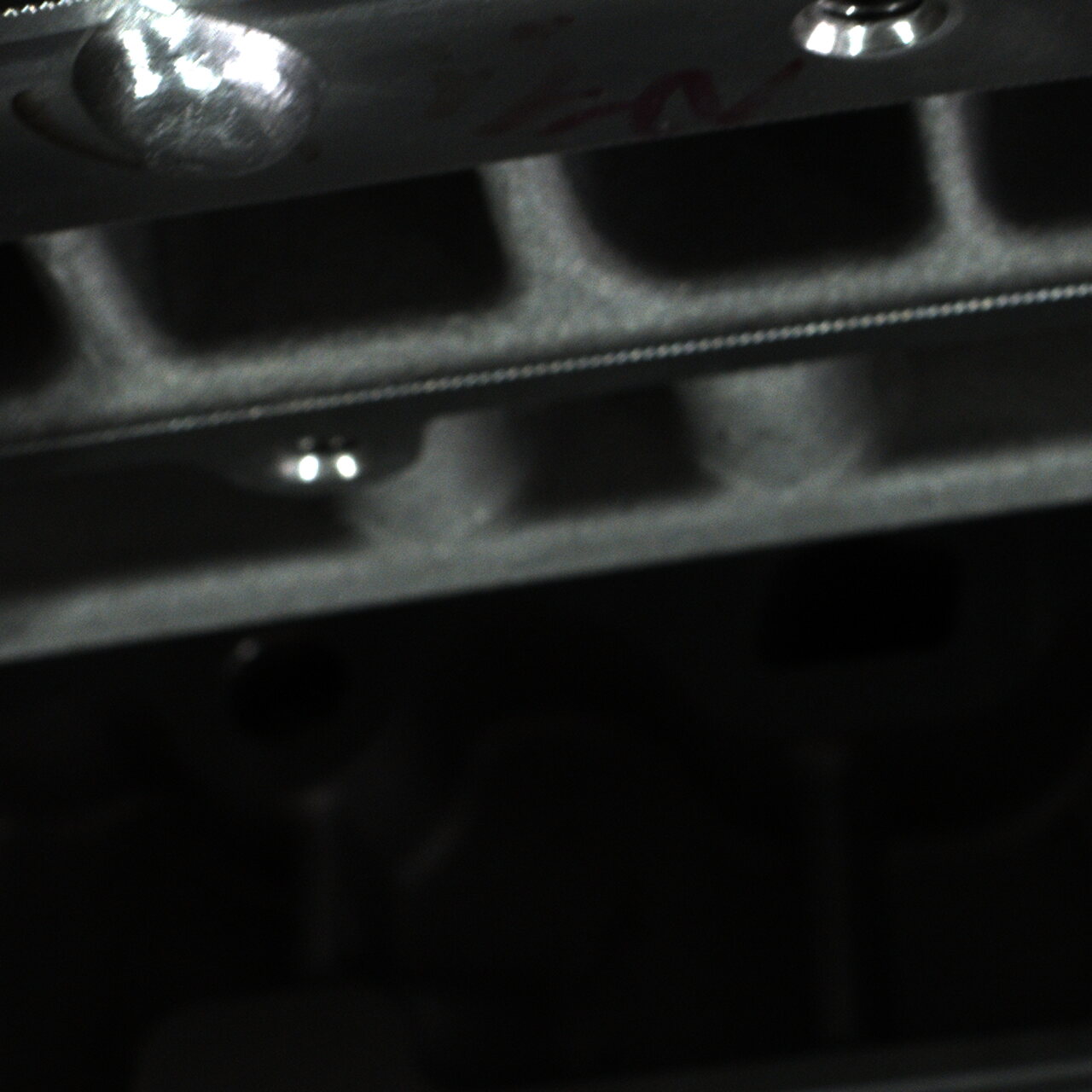

Supplement: Supplemental Information 1 — The CSD-DET dataset was collected from Guangde Hardware Casting Factory and Wuhu Automobile Casting Factory in May 2023. The CSD-DET dataset was used to train and measure the advantages of the DES-YOLO model. This is the filtered partial dataset. [file peerj-cs-10-2224-s001.zip › CastingDefectsDataSet/data/Ho_190.jpg]

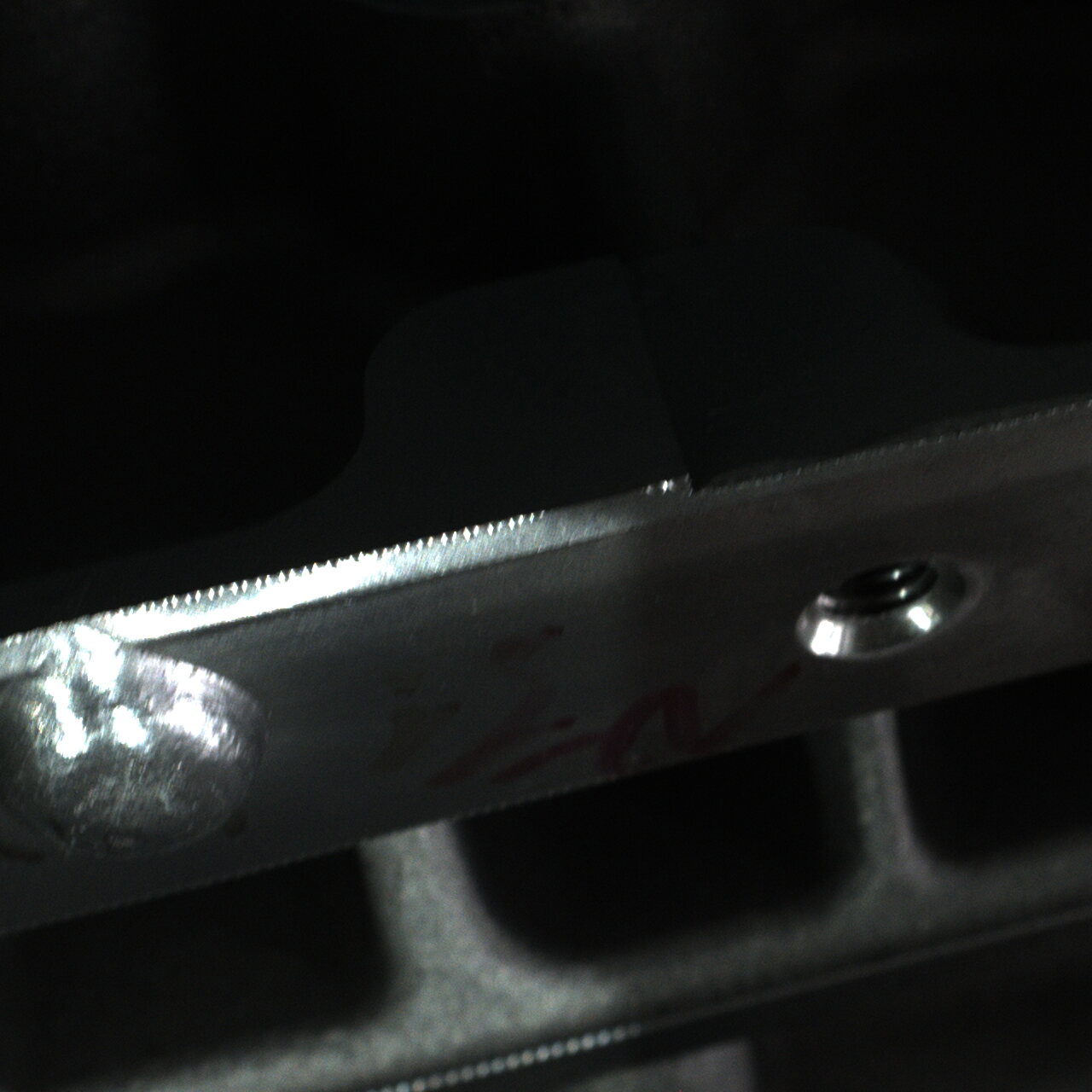

Supplement: Supplemental Information 1 — The CSD-DET dataset was collected from Guangde Hardware Casting Factory and Wuhu Automobile Casting Factory in May 2023. The CSD-DET dataset was used to train and measure the advantages of the DES-YOLO model. This is the filtered partial dataset. [file peerj-cs-10-2224-s001.zip › CastingDefectsDataSet/data/Ho_198.jpg]

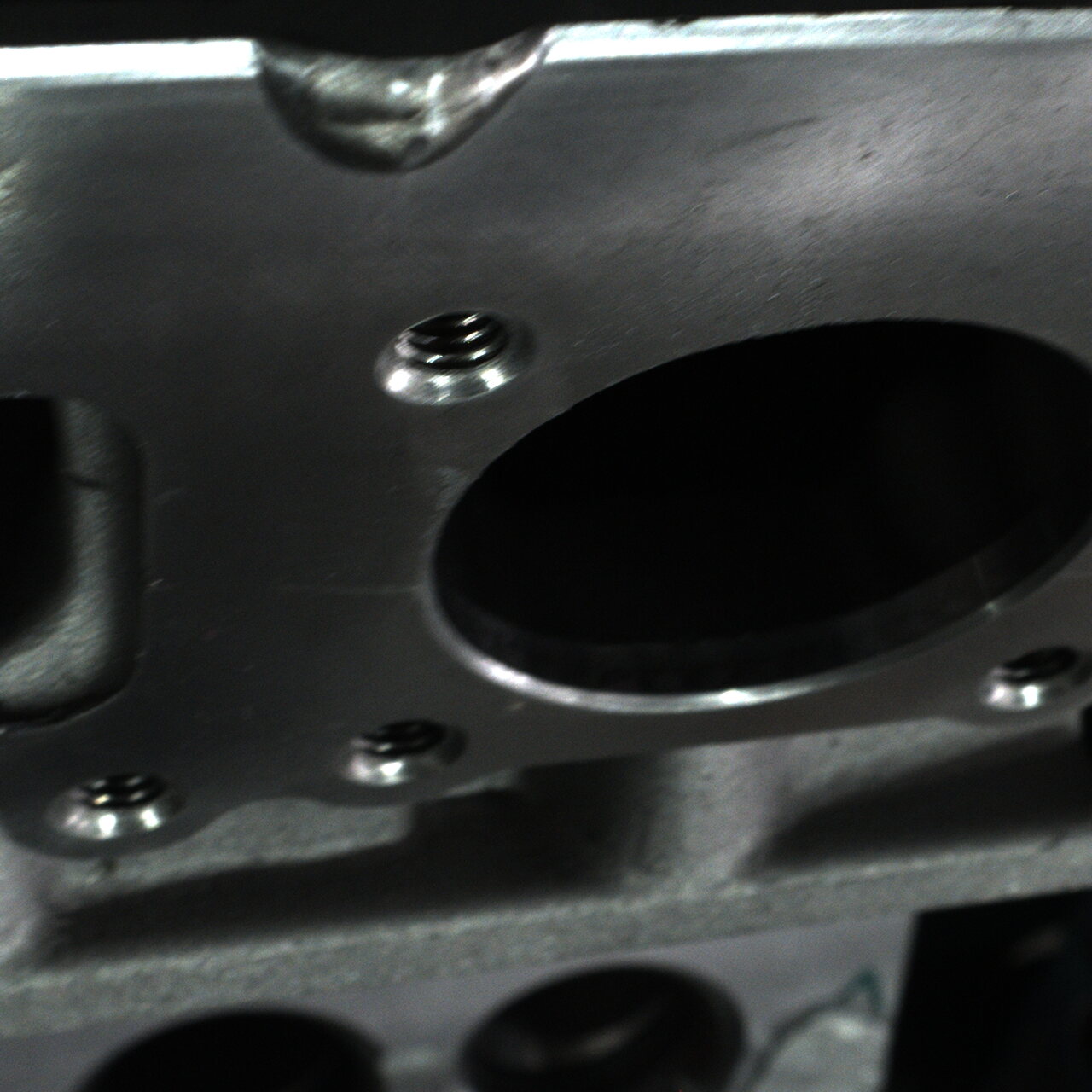

Supplement: Supplemental Information 1 — The CSD-DET dataset was collected from Guangde Hardware Casting Factory and Wuhu Automobile Casting Factory in May 2023. The CSD-DET dataset was used to train and measure the advantages of the DES-YOLO model. This is the filtered partial dataset. [file peerj-cs-10-2224-s001.zip › CastingDefectsDataSet/data/Ho_226.jpg]

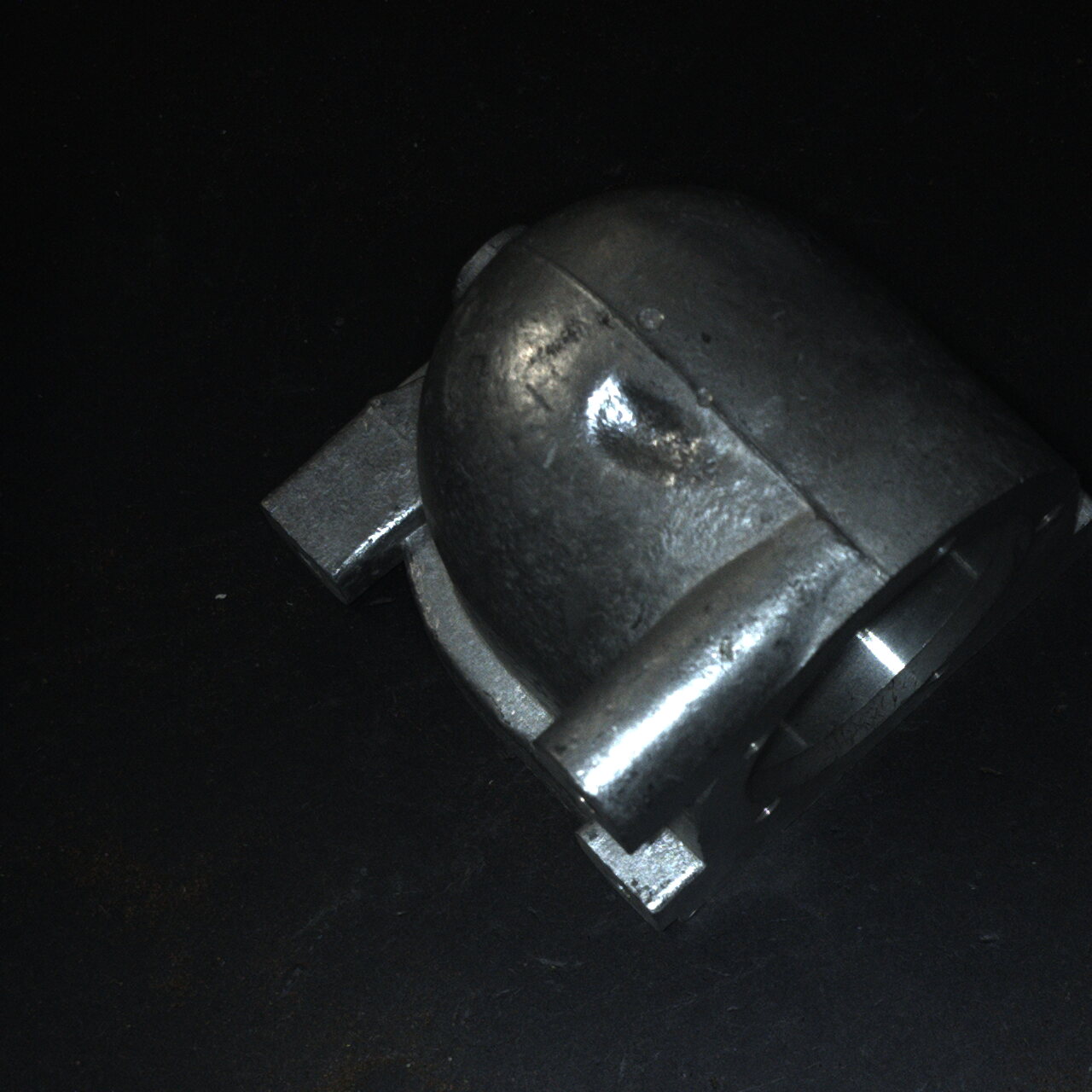

Supplement: Supplemental Information 1 — The CSD-DET dataset was collected from Guangde Hardware Casting Factory and Wuhu Automobile Casting Factory in May 2023. The CSD-DET dataset was used to train and measure the advantages of the DES-YOLO model. This is the filtered partial dataset. [file peerj-cs-10-2224-s001.zip › CastingDefectsDataSet/data/Ho_314.jpg]

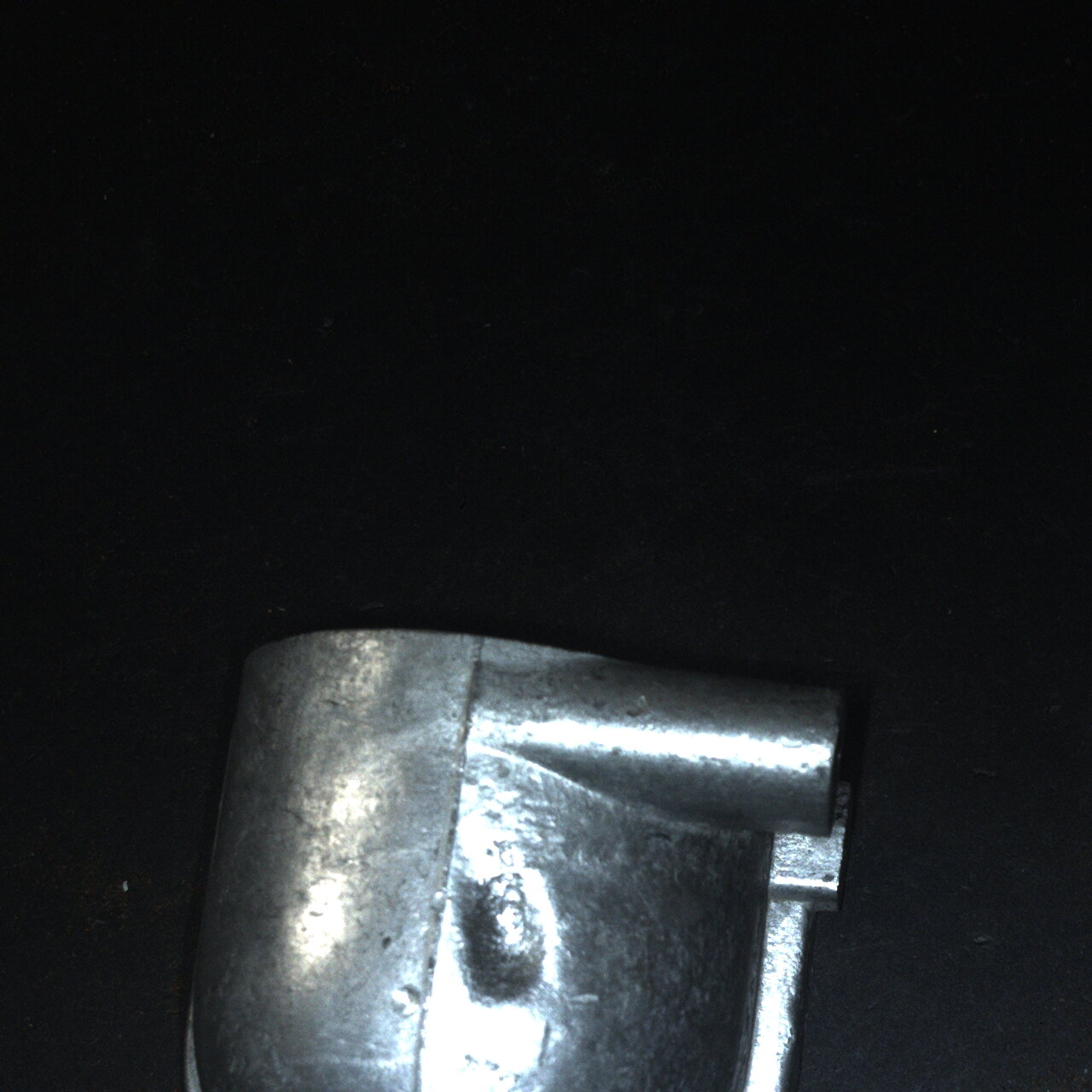

Supplement: Supplemental Information 1 — The CSD-DET dataset was collected from Guangde Hardware Casting Factory and Wuhu Automobile Casting Factory in May 2023. The CSD-DET dataset was used to train and measure the advantages of the DES-YOLO model. This is the filtered partial dataset. [file peerj-cs-10-2224-s001.zip › CastingDefectsDataSet/data/Ho_334.jpg]

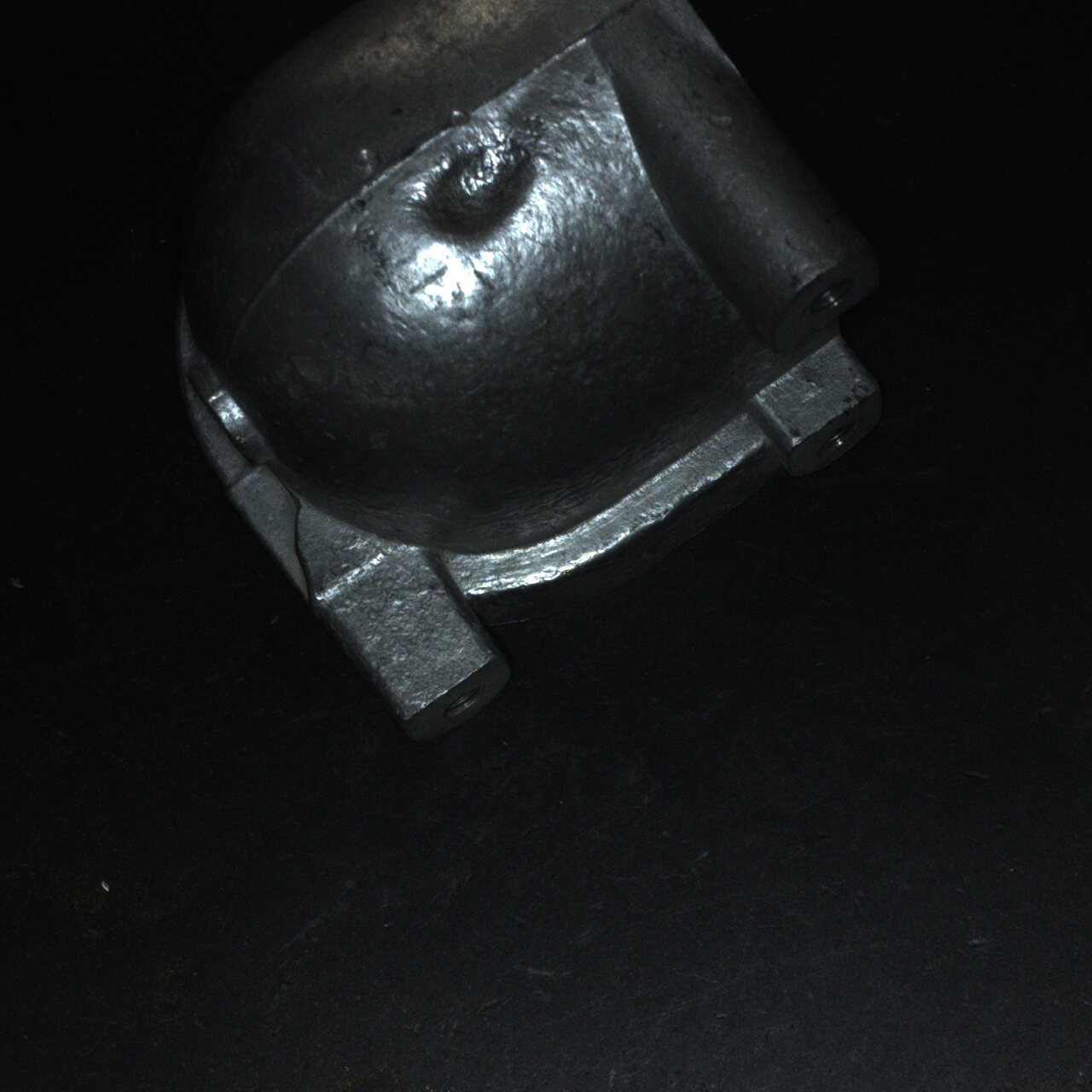

Supplement: Supplemental Information 1 — The CSD-DET dataset was collected from Guangde Hardware Casting Factory and Wuhu Automobile Casting Factory in May 2023. The CSD-DET dataset was used to train and measure the advantages of the DES-YOLO model. This is the filtered partial dataset. [file peerj-cs-10-2224-s001.zip › CastingDefectsDataSet/data/Ho_382.jpg]

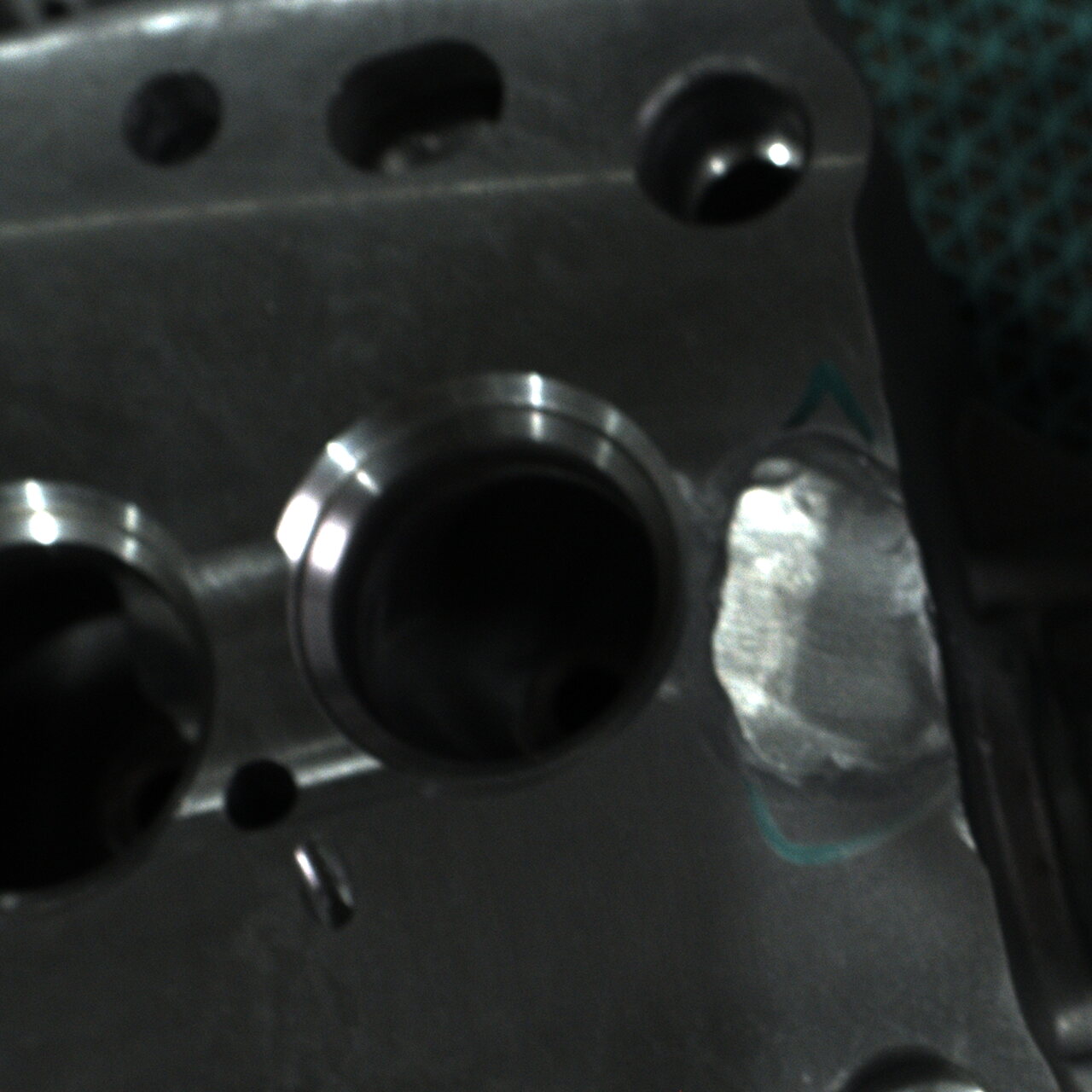

Supplement: Supplemental Information 1 — The CSD-DET dataset was collected from Guangde Hardware Casting Factory and Wuhu Automobile Casting Factory in May 2023. The CSD-DET dataset was used to train and measure the advantages of the DES-YOLO model. This is the filtered partial dataset. [file peerj-cs-10-2224-s001.zip › CastingDefectsDataSet/data/Ho_46.jpg]

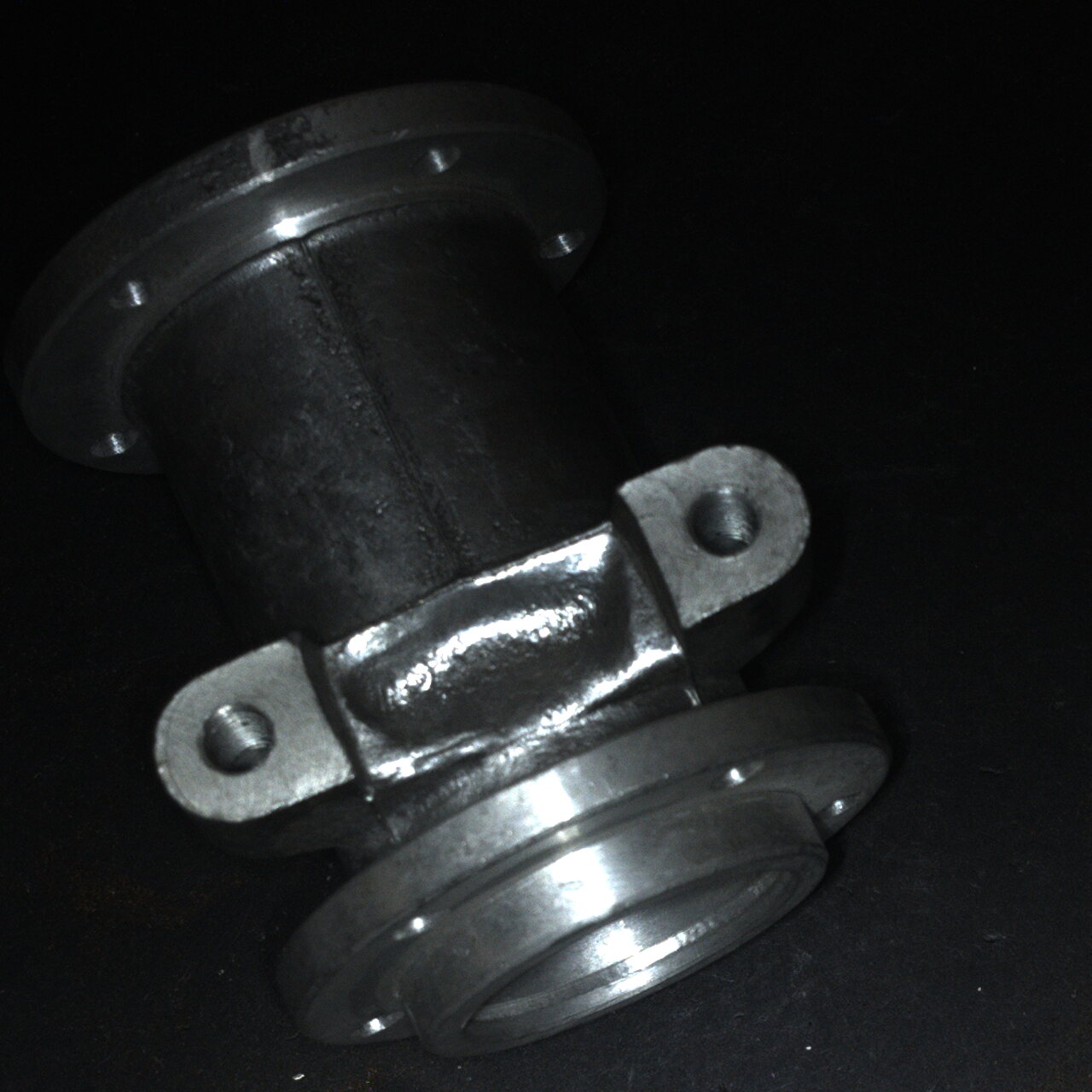

Supplement: Supplemental Information 1 — The CSD-DET dataset was collected from Guangde Hardware Casting Factory and Wuhu Automobile Casting Factory in May 2023. The CSD-DET dataset was used to train and measure the advantages of the DES-YOLO model. This is the filtered partial dataset. [file peerj-cs-10-2224-s001.zip › CastingDefectsDataSet/data/Ho_486.jpg]

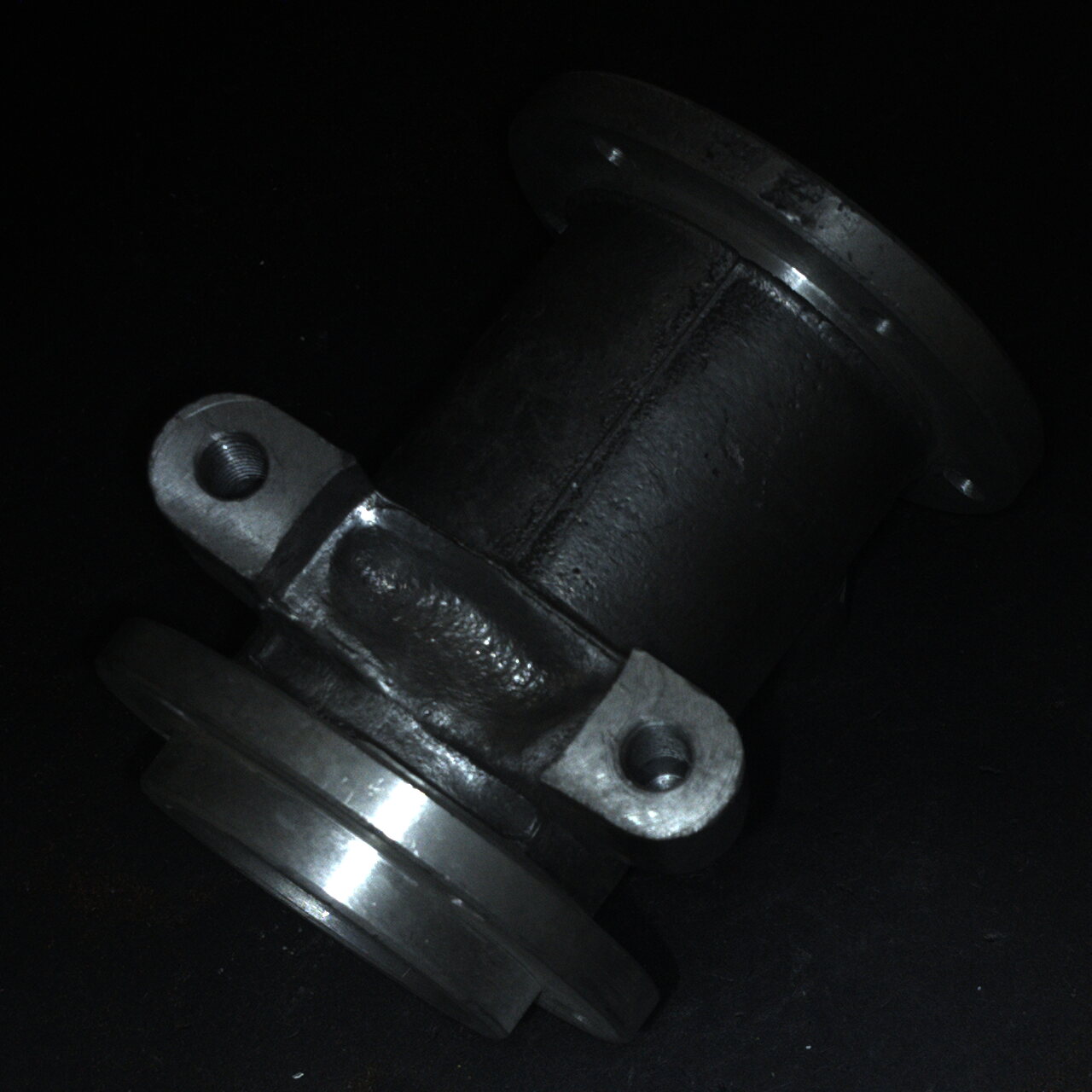

Supplement: Supplemental Information 1 — The CSD-DET dataset was collected from Guangde Hardware Casting Factory and Wuhu Automobile Casting Factory in May 2023. The CSD-DET dataset was used to train and measure the advantages of the DES-YOLO model. This is the filtered partial dataset. [file peerj-cs-10-2224-s001.zip › CastingDefectsDataSet/data/Ho_490.jpg]

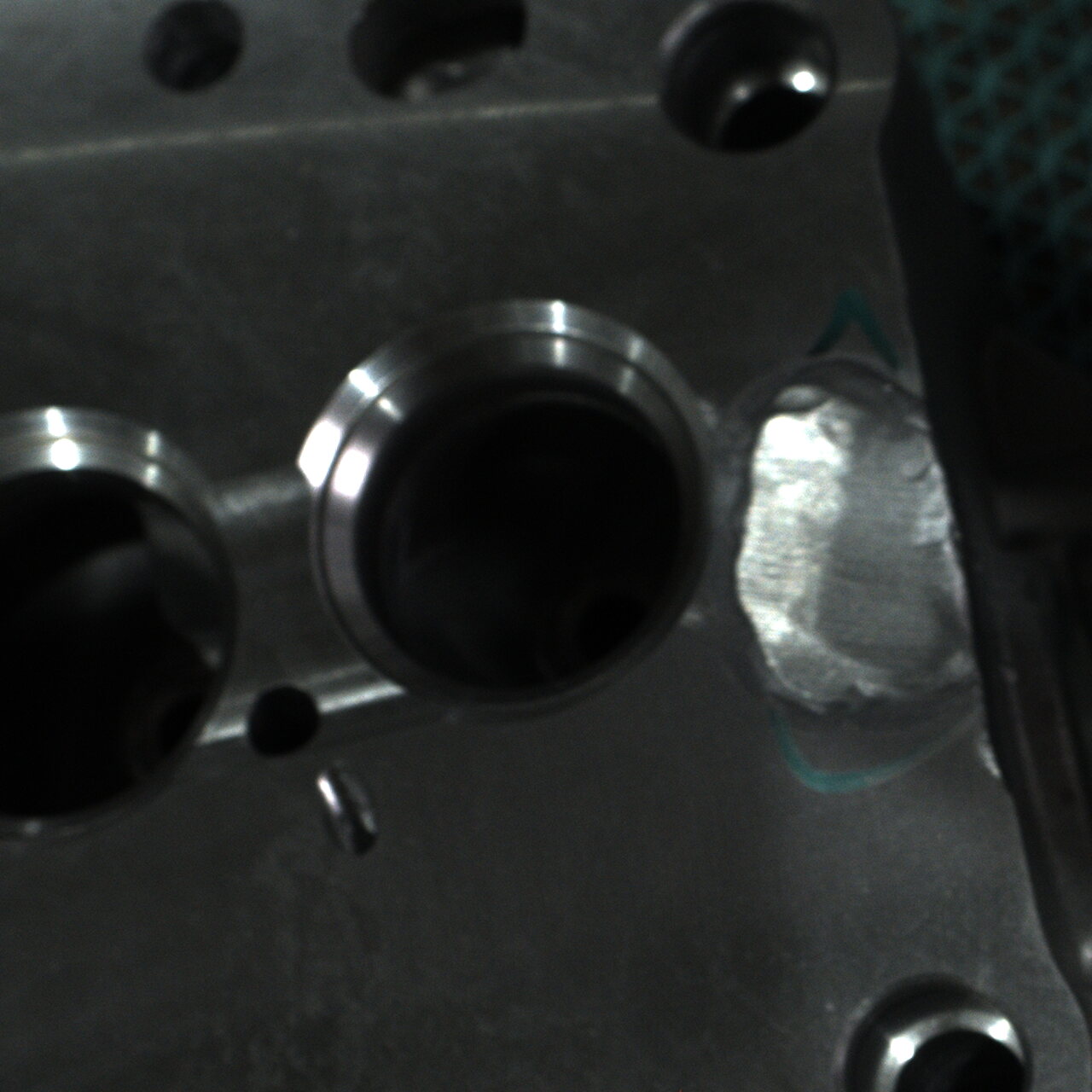

Supplement: Supplemental Information 1 — The CSD-DET dataset was collected from Guangde Hardware Casting Factory and Wuhu Automobile Casting Factory in May 2023. The CSD-DET dataset was used to train and measure the advantages of the DES-YOLO model. This is the filtered partial dataset. [file peerj-cs-10-2224-s001.zip › CastingDefectsDataSet/data/Ho_50.jpg]

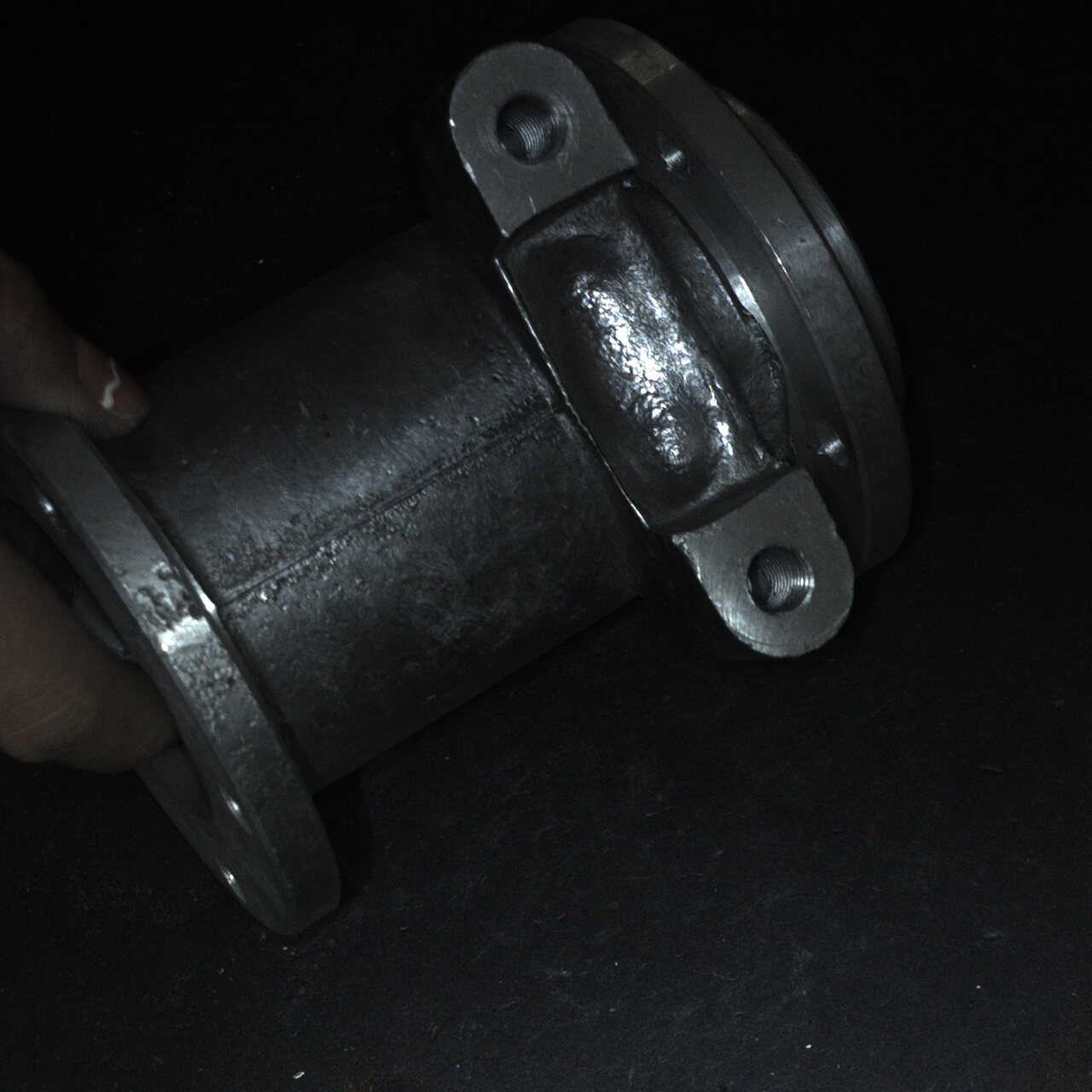

Supplement: Supplemental Information 1 — The CSD-DET dataset was collected from Guangde Hardware Casting Factory and Wuhu Automobile Casting Factory in May 2023. The CSD-DET dataset was used to train and measure the advantages of the DES-YOLO model. This is the filtered partial dataset. [file peerj-cs-10-2224-s001.zip › CastingDefectsDataSet/data/Ho_510.jpg]

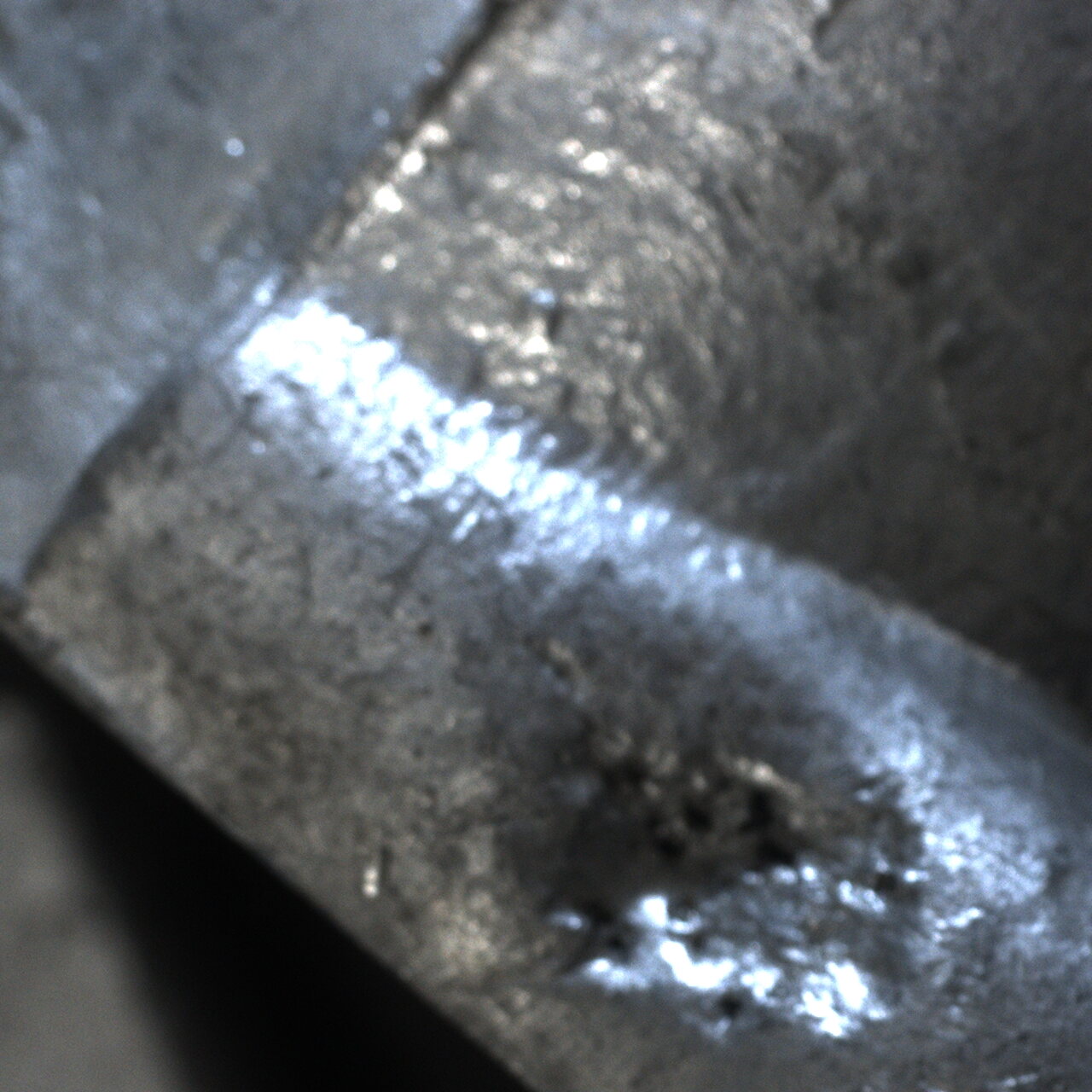

Supplement: Supplemental Information 1 — The CSD-DET dataset was collected from Guangde Hardware Casting Factory and Wuhu Automobile Casting Factory in May 2023. The CSD-DET dataset was used to train and measure the advantages of the DES-YOLO model. This is the filtered partial dataset. [file peerj-cs-10-2224-s001.zip › CastingDefectsDataSet/data/Ho_622.jpg]

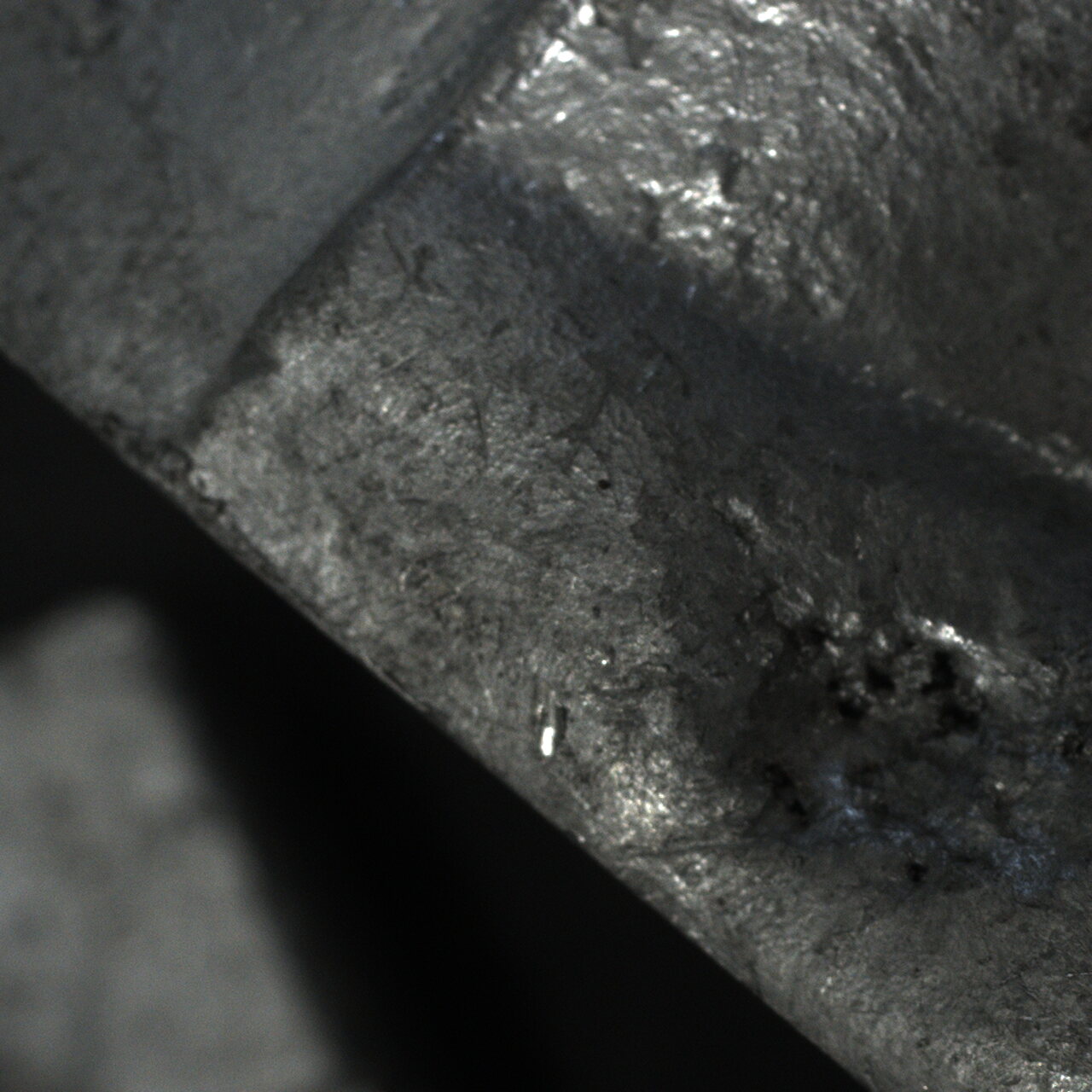

Supplement: Supplemental Information 1 — The CSD-DET dataset was collected from Guangde Hardware Casting Factory and Wuhu Automobile Casting Factory in May 2023. The CSD-DET dataset was used to train and measure the advantages of the DES-YOLO model. This is the filtered partial dataset. [file peerj-cs-10-2224-s001.zip › CastingDefectsDataSet/data/Ho_630.jpg]

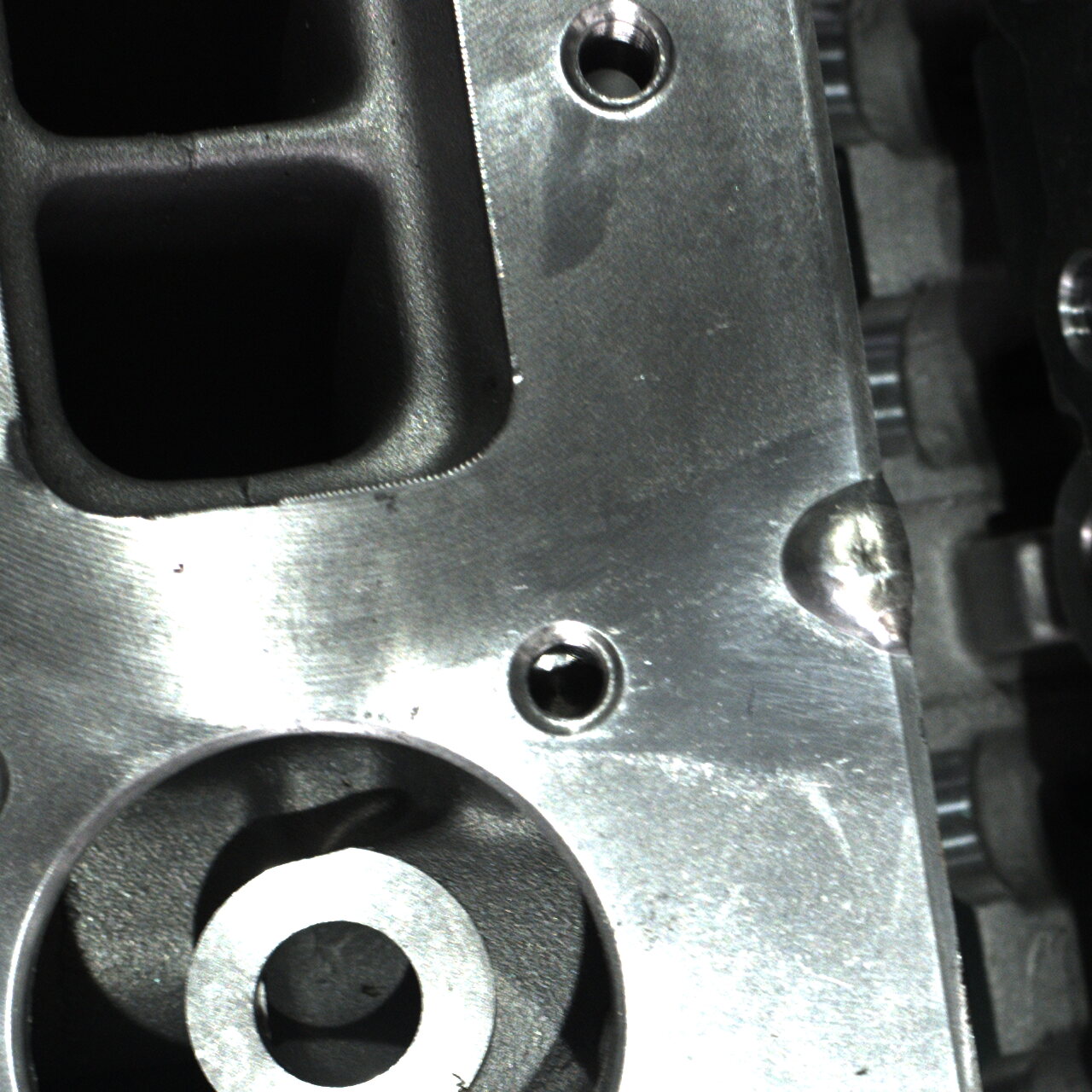

Supplement: Supplemental Information 1 — The CSD-DET dataset was collected from Guangde Hardware Casting Factory and Wuhu Automobile Casting Factory in May 2023. The CSD-DET dataset was used to train and measure the advantages of the DES-YOLO model. This is the filtered partial dataset. [file peerj-cs-10-2224-s001.zip › CastingDefectsDataSet/data/Ho_98.jpg]

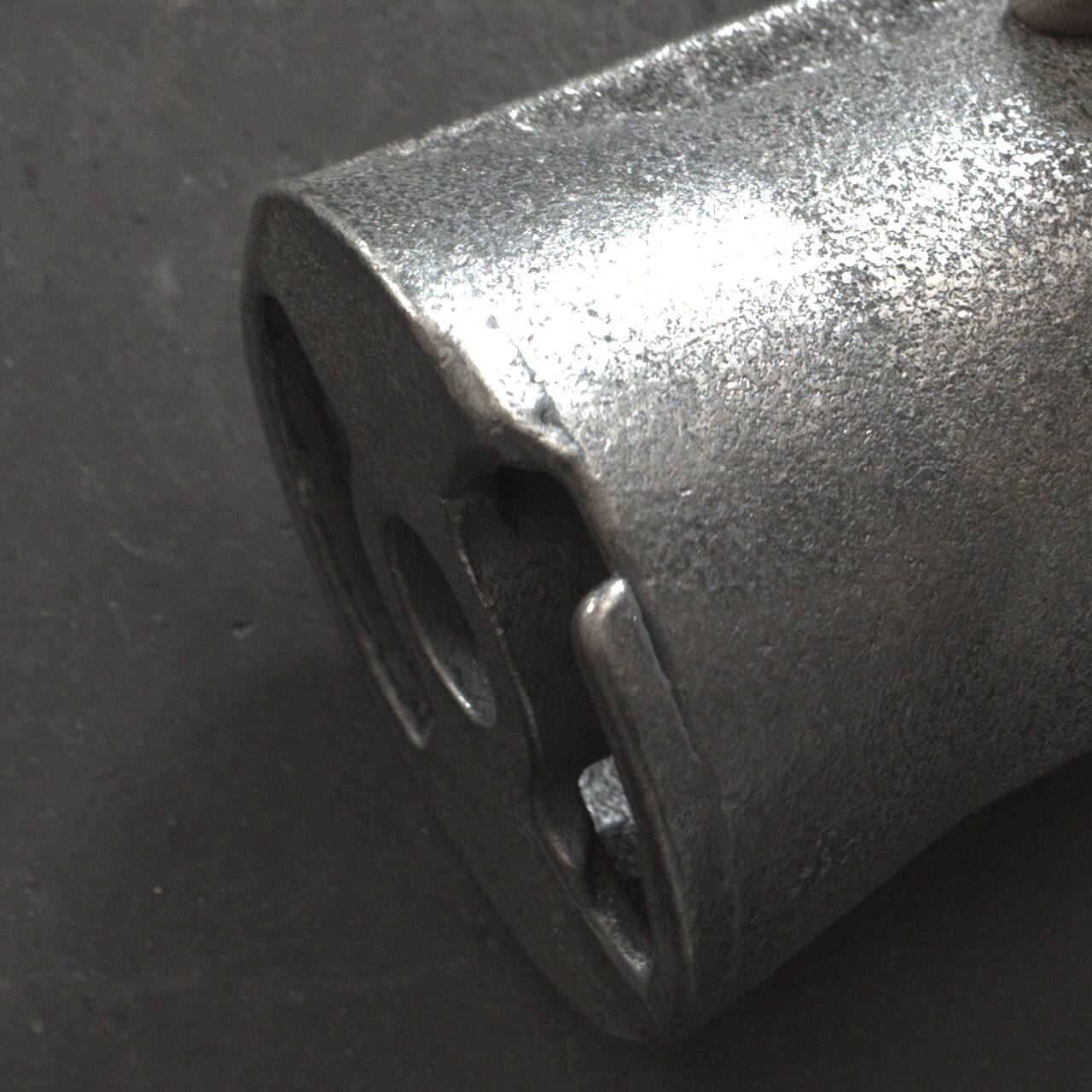

Supplement: Supplemental Information 1 — The CSD-DET dataset was collected from Guangde Hardware Casting Factory and Wuhu Automobile Casting Factory in May 2023. The CSD-DET dataset was used to train and measure the advantages of the DES-YOLO model. This is the filtered partial dataset. [file peerj-cs-10-2224-s001.zip › CastingDefectsDataSet/data/Mr_136.jpg]

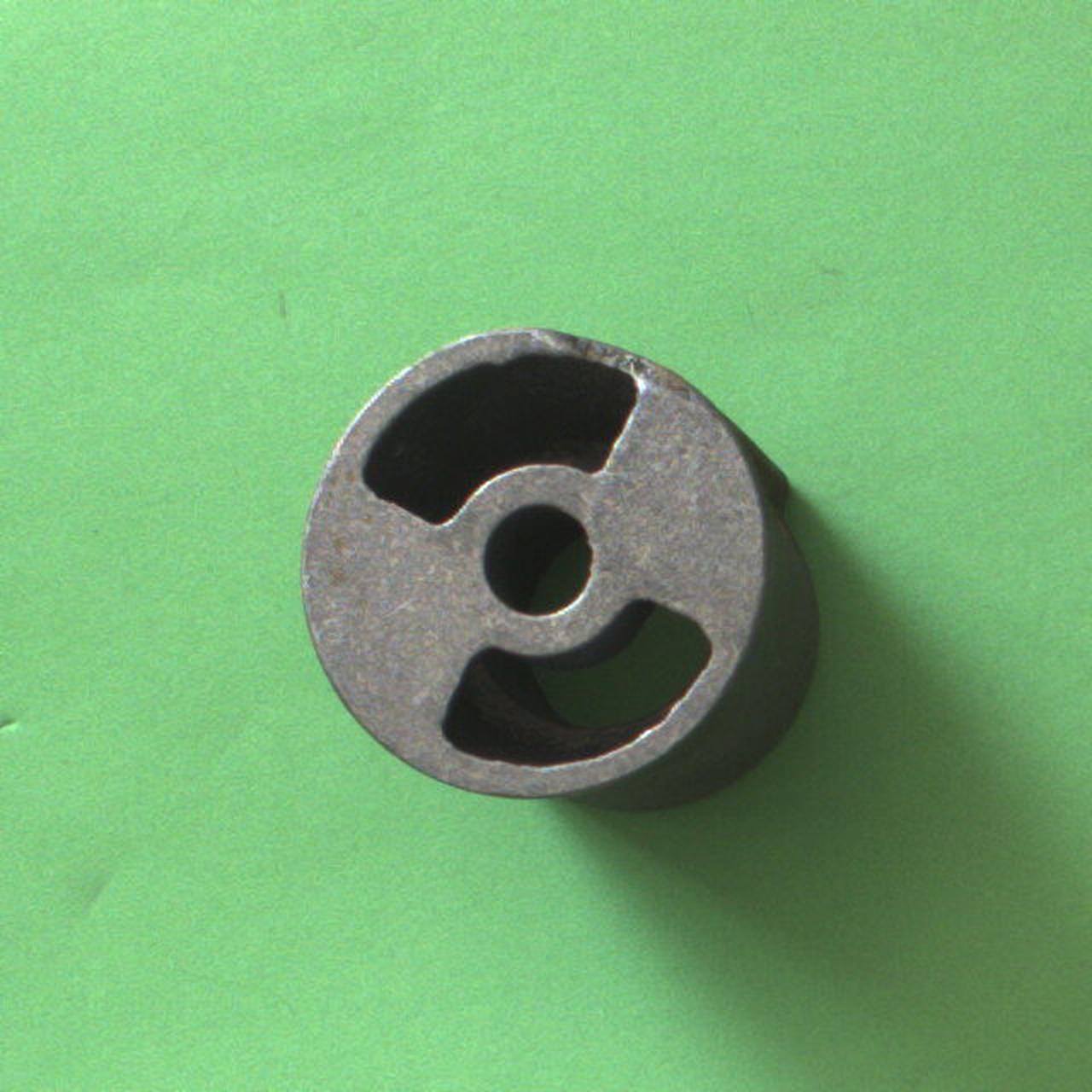

Supplement: Supplemental Information 1 — The CSD-DET dataset was collected from Guangde Hardware Casting Factory and Wuhu Automobile Casting Factory in May 2023. The CSD-DET dataset was used to train and measure the advantages of the DES-YOLO model. This is the filtered partial dataset. [file peerj-cs-10-2224-s001.zip › CastingDefectsDataSet/data/Mr_140.jpg]

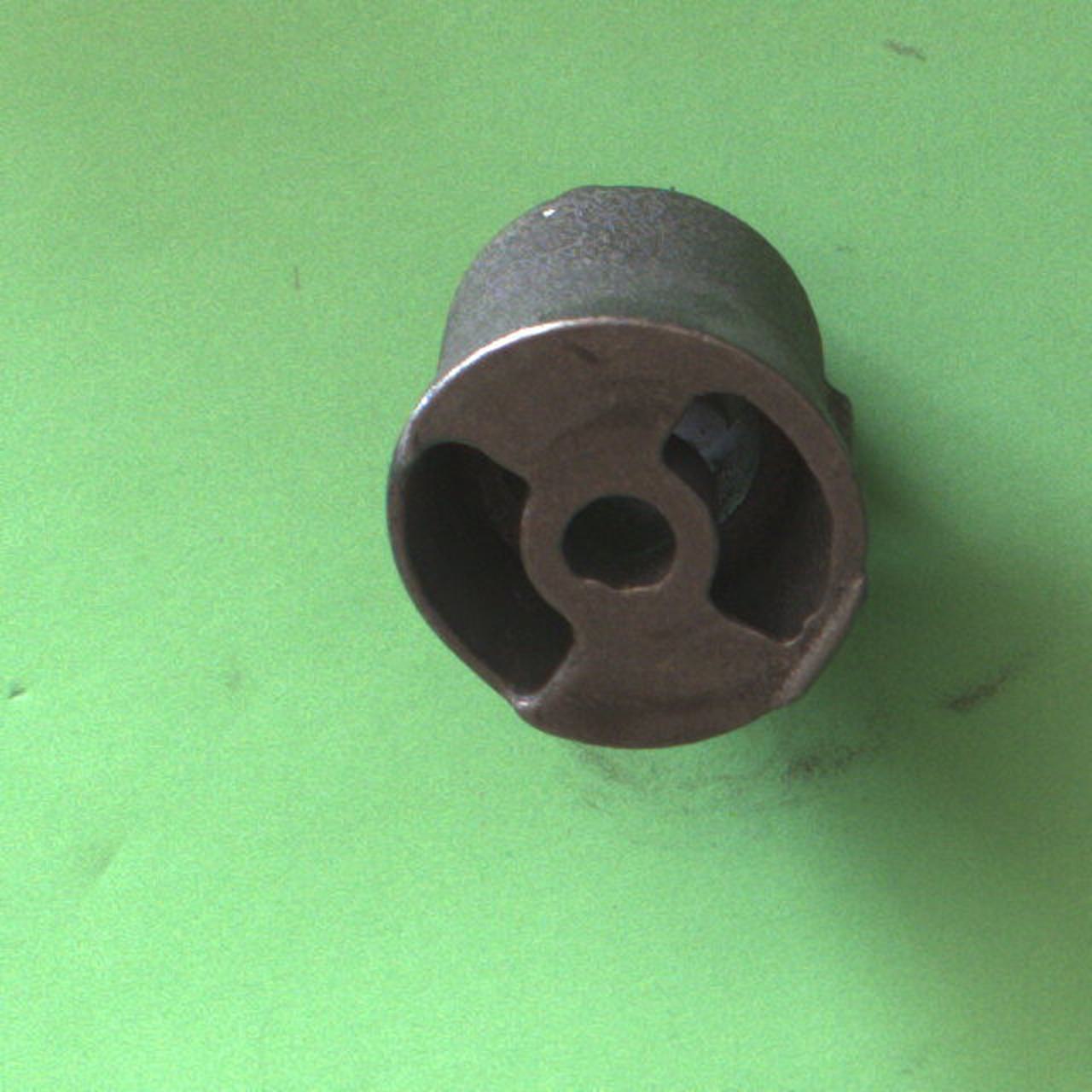

Supplement: Supplemental Information 1 — The CSD-DET dataset was collected from Guangde Hardware Casting Factory and Wuhu Automobile Casting Factory in May 2023. The CSD-DET dataset was used to train and measure the advantages of the DES-YOLO model. This is the filtered partial dataset. [file peerj-cs-10-2224-s001.zip › CastingDefectsDataSet/data/Mr_180.jpg]

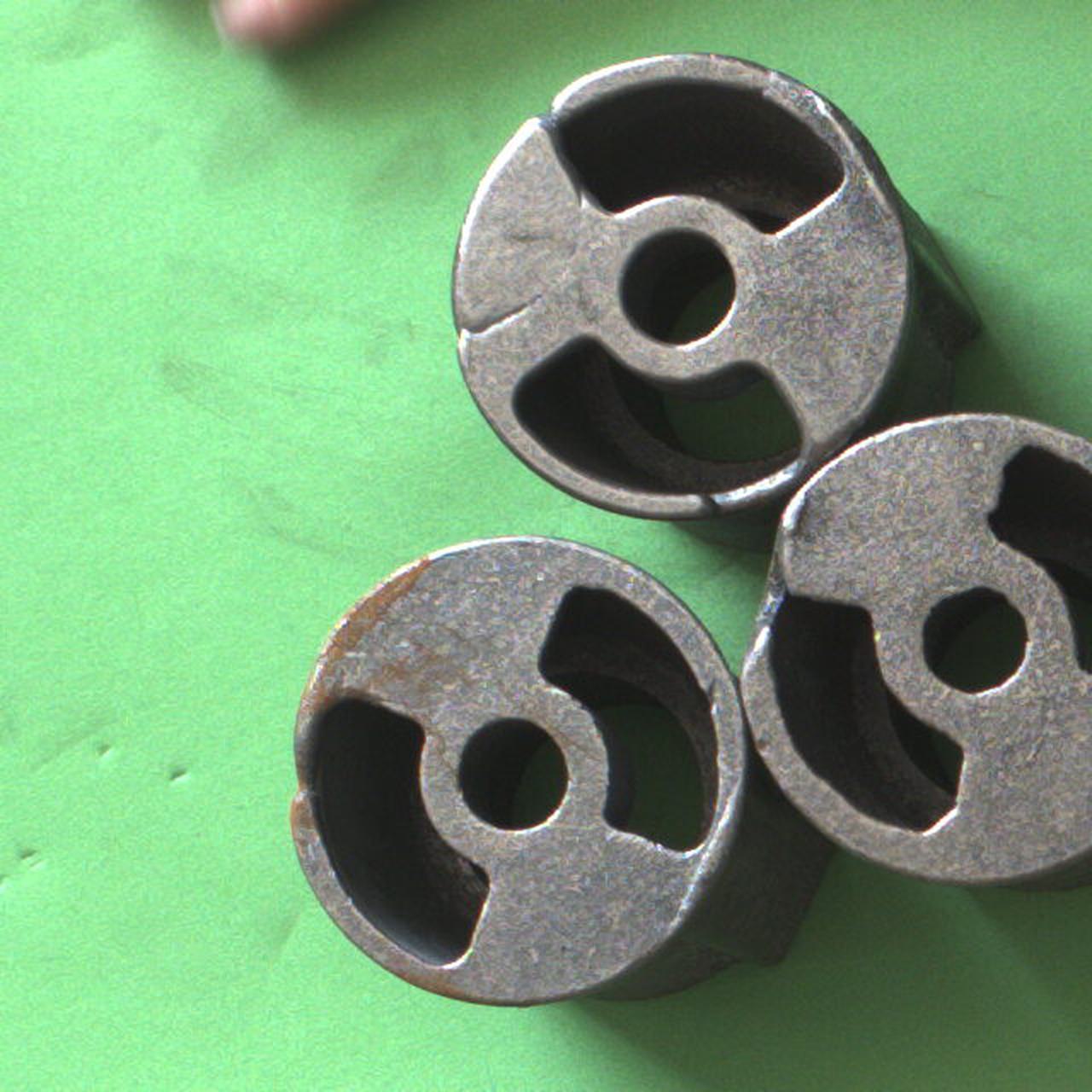

Supplement: Supplemental Information 1 — The CSD-DET dataset was collected from Guangde Hardware Casting Factory and Wuhu Automobile Casting Factory in May 2023. The CSD-DET dataset was used to train and measure the advantages of the DES-YOLO model. This is the filtered partial dataset. [file peerj-cs-10-2224-s001.zip › CastingDefectsDataSet/data/Mr_276.jpg]

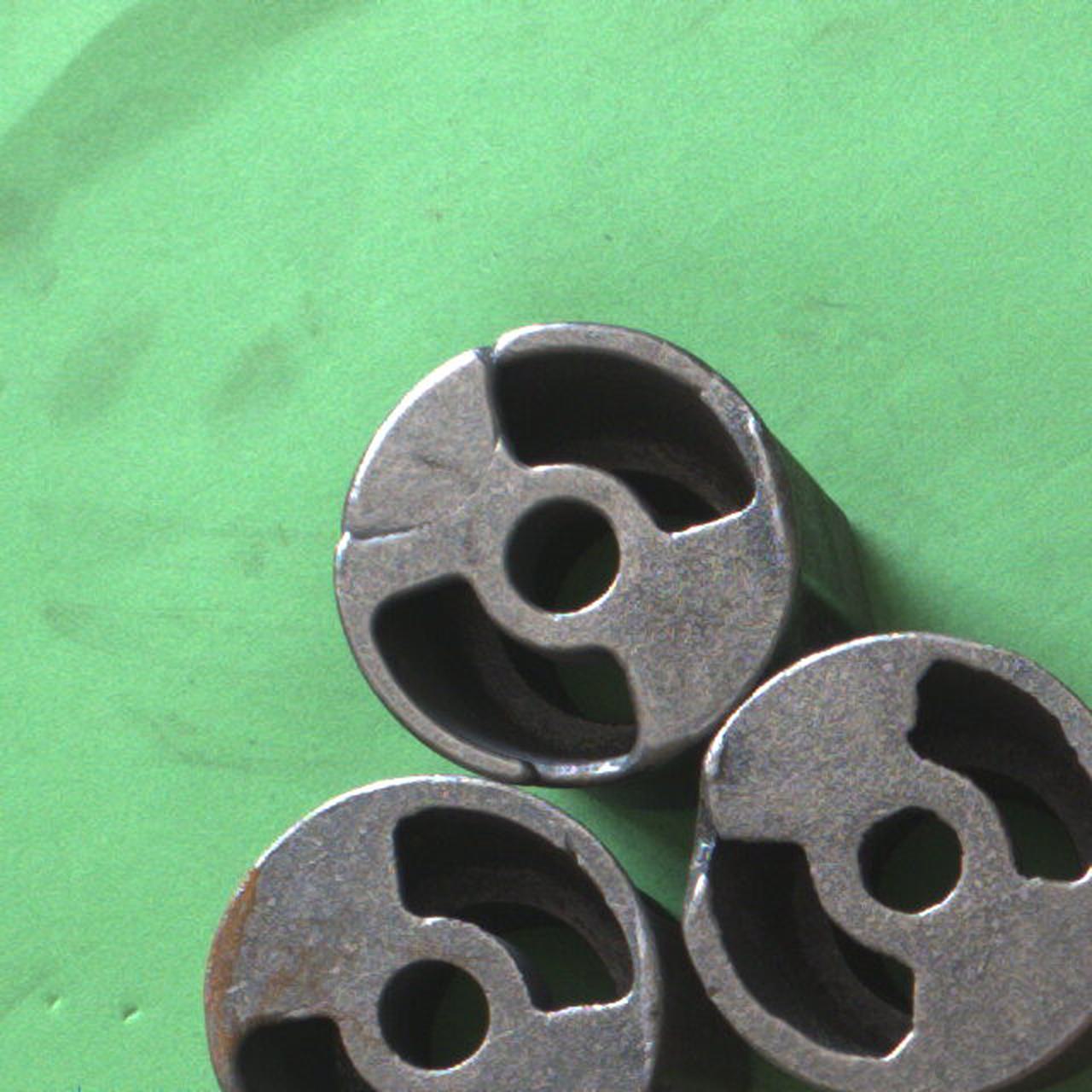

Supplement: Supplemental Information 1 — The CSD-DET dataset was collected from Guangde Hardware Casting Factory and Wuhu Automobile Casting Factory in May 2023. The CSD-DET dataset was used to train and measure the advantages of the DES-YOLO model. This is the filtered partial dataset. [file peerj-cs-10-2224-s001.zip › CastingDefectsDataSet/data/Mr_280.jpg]

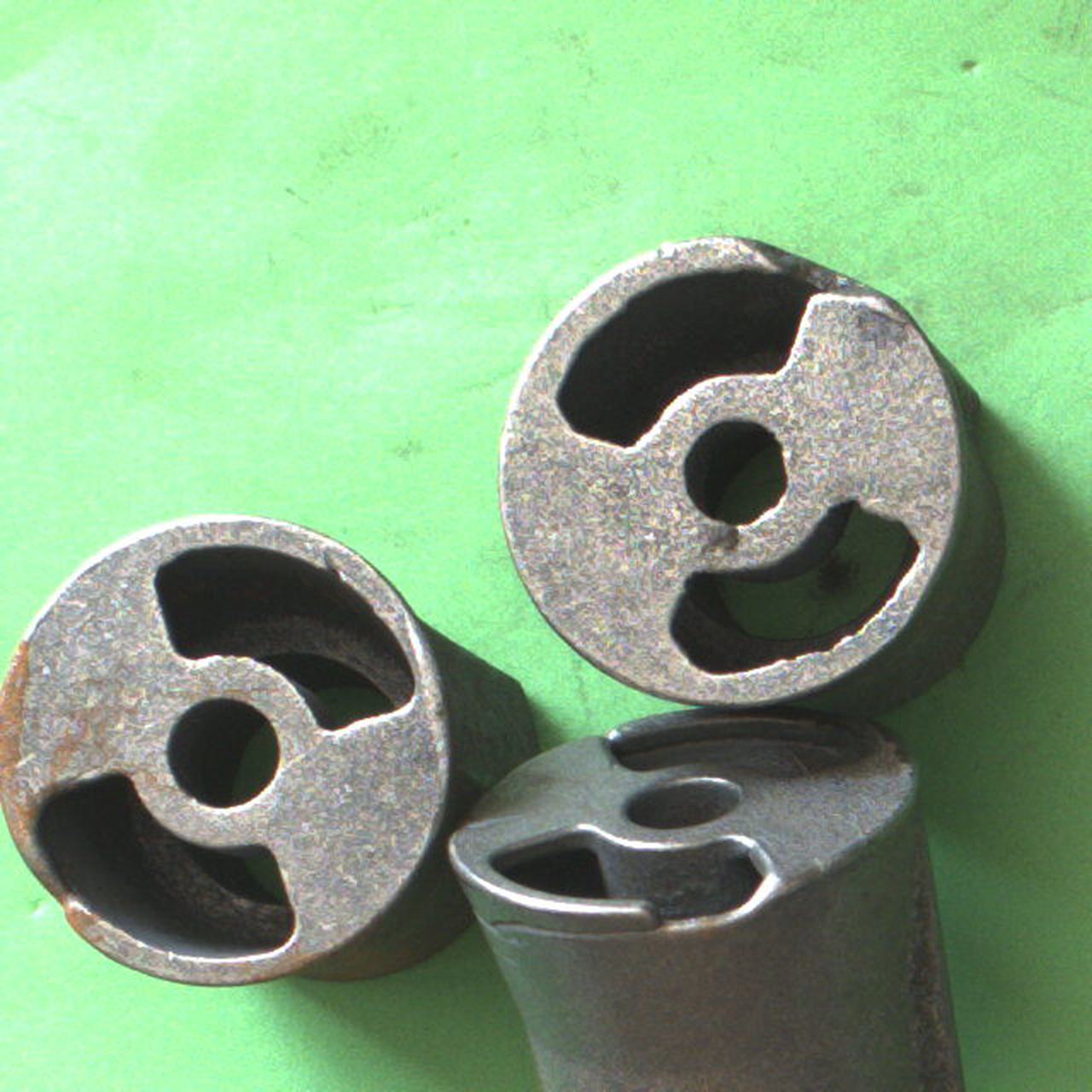

Supplement: Supplemental Information 1 — The CSD-DET dataset was collected from Guangde Hardware Casting Factory and Wuhu Automobile Casting Factory in May 2023. The CSD-DET dataset was used to train and measure the advantages of the DES-YOLO model. This is the filtered partial dataset. [file peerj-cs-10-2224-s001.zip › CastingDefectsDataSet/data/Mr_328.jpg]

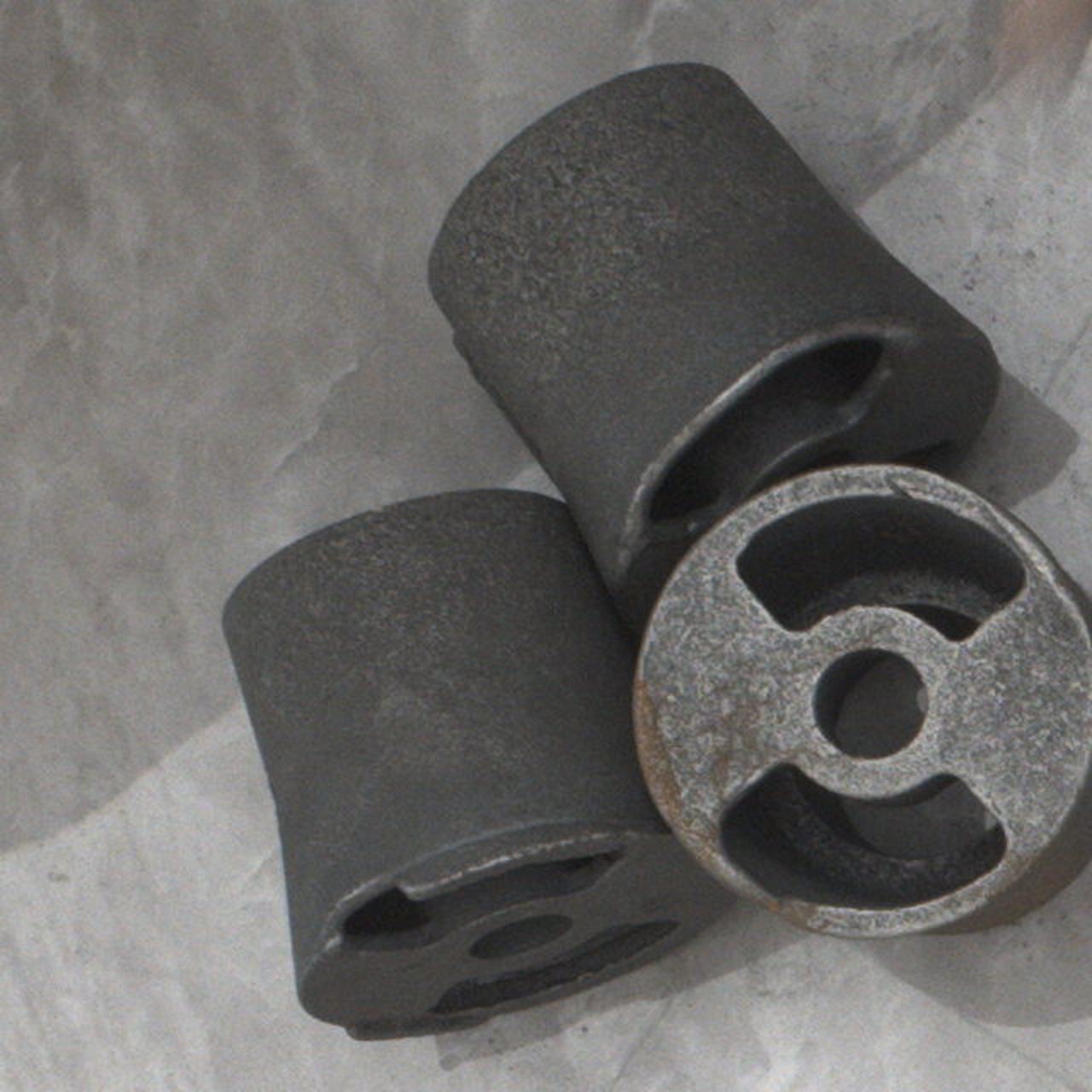

Supplement: Supplemental Information 1 — The CSD-DET dataset was collected from Guangde Hardware Casting Factory and Wuhu Automobile Casting Factory in May 2023. The CSD-DET dataset was used to train and measure the advantages of the DES-YOLO model. This is the filtered partial dataset. [file peerj-cs-10-2224-s001.zip › CastingDefectsDataSet/data/Mr_432.jpg]

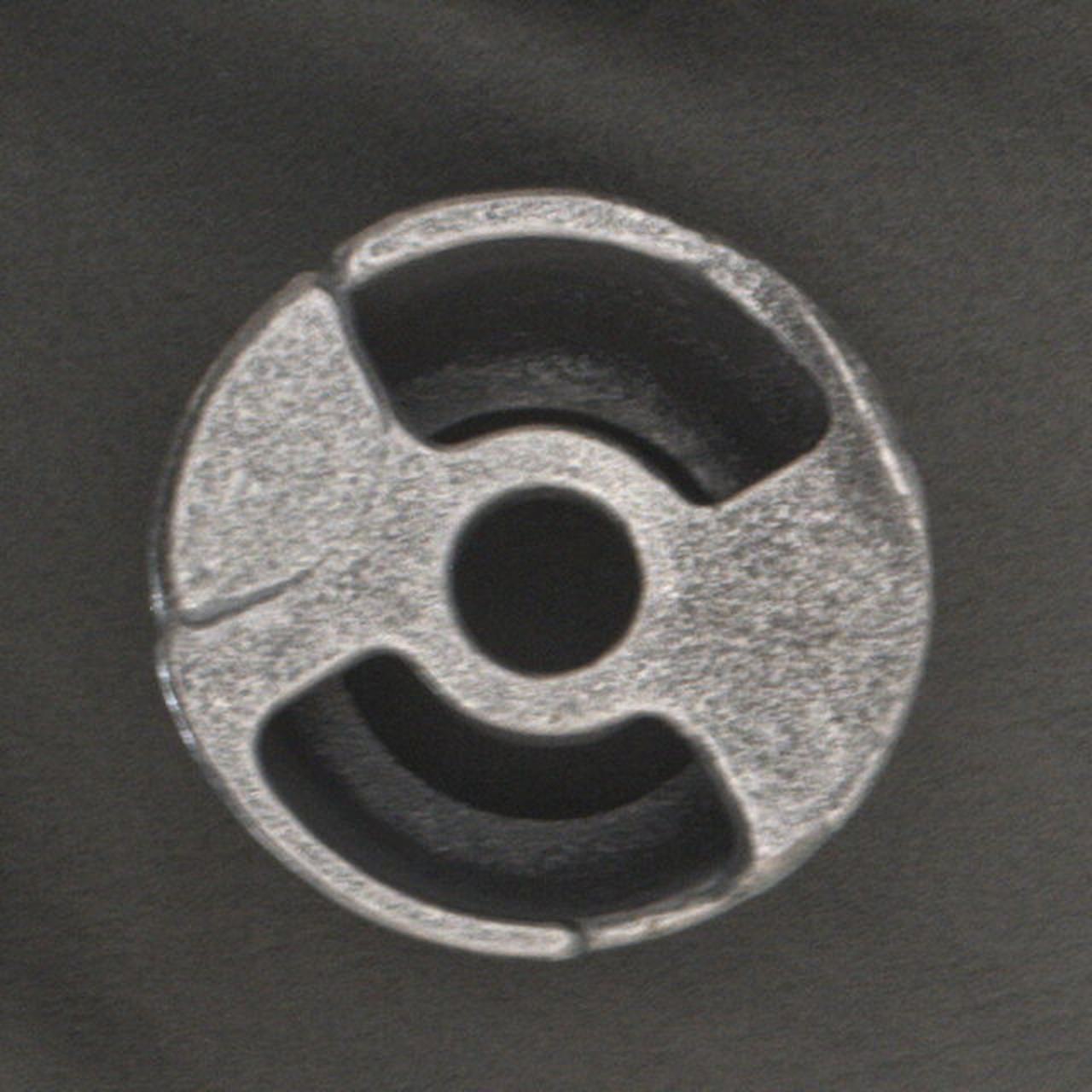

Supplement: Supplemental Information 1 — The CSD-DET dataset was collected from Guangde Hardware Casting Factory and Wuhu Automobile Casting Factory in May 2023. The CSD-DET dataset was used to train and measure the advantages of the DES-YOLO model. This is the filtered partial dataset. [file peerj-cs-10-2224-s001.zip › CastingDefectsDataSet/data/Mr_452.jpg]

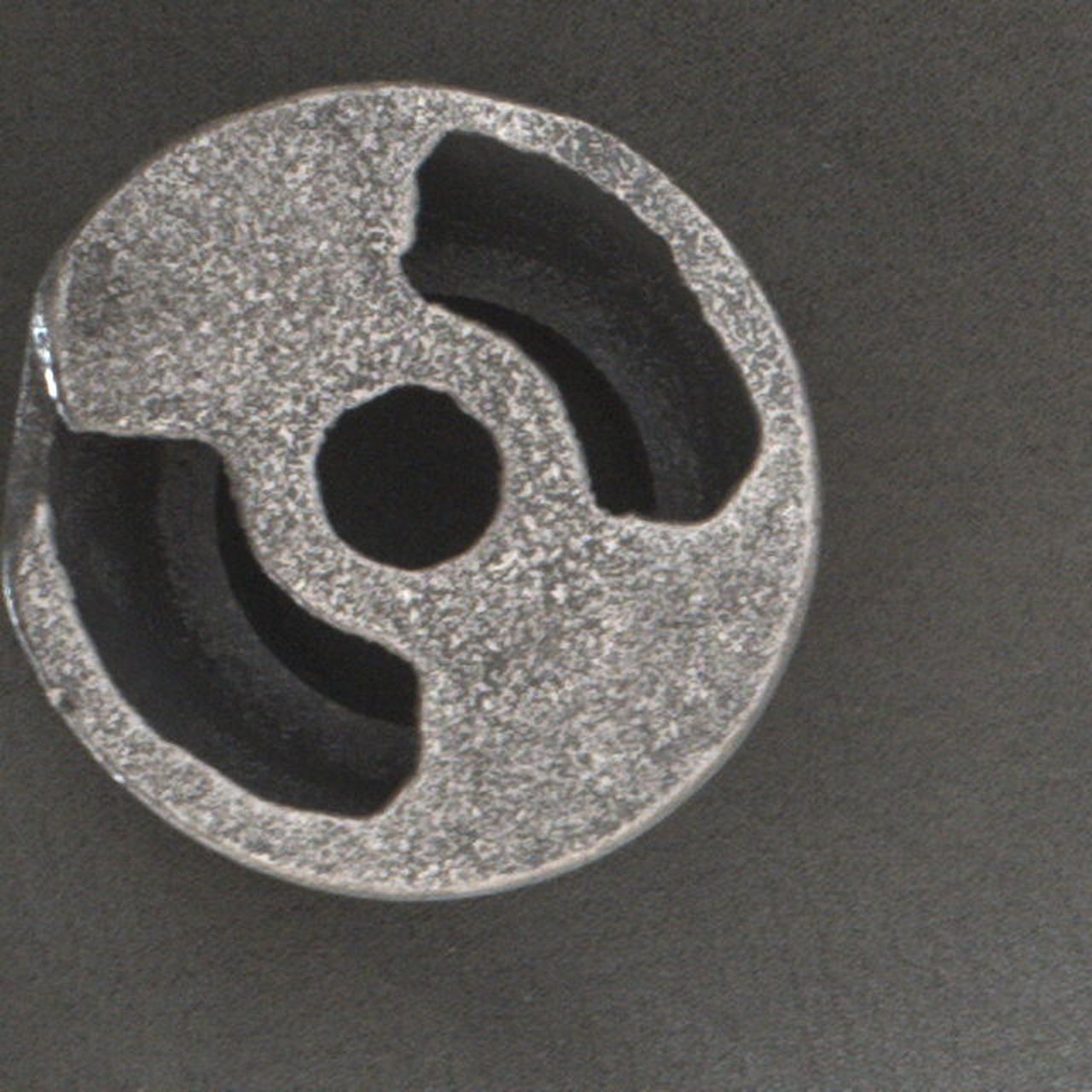

Supplement: Supplemental Information 1 — The CSD-DET dataset was collected from Guangde Hardware Casting Factory and Wuhu Automobile Casting Factory in May 2023. The CSD-DET dataset was used to train and measure the advantages of the DES-YOLO model. This is the filtered partial dataset. [file peerj-cs-10-2224-s001.zip › CastingDefectsDataSet/data/Mr_456.jpg]

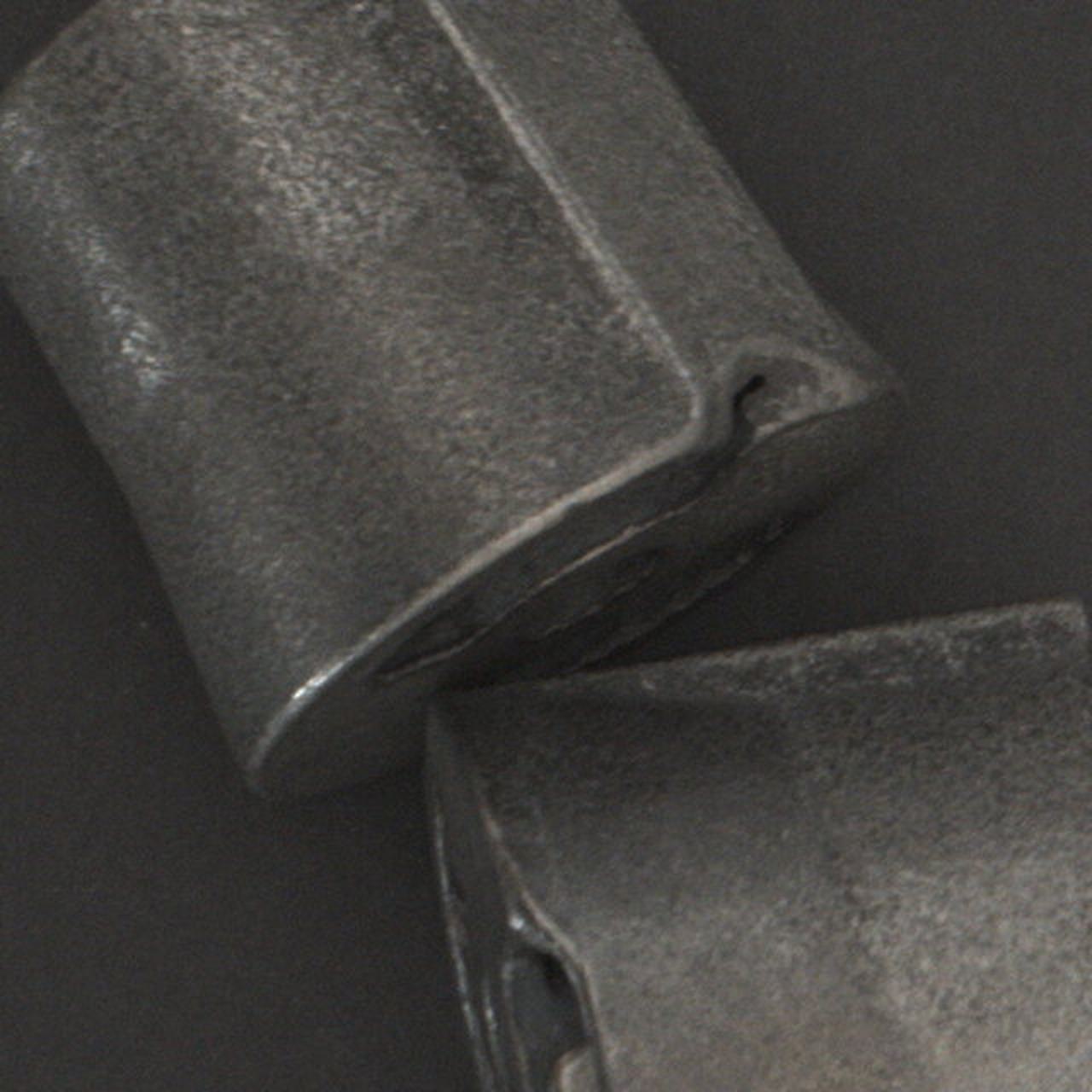

Supplement: Supplemental Information 1 — The CSD-DET dataset was collected from Guangde Hardware Casting Factory and Wuhu Automobile Casting Factory in May 2023. The CSD-DET dataset was used to train and measure the advantages of the DES-YOLO model. This is the filtered partial dataset. [file peerj-cs-10-2224-s001.zip › CastingDefectsDataSet/data/Mr_568.jpg]

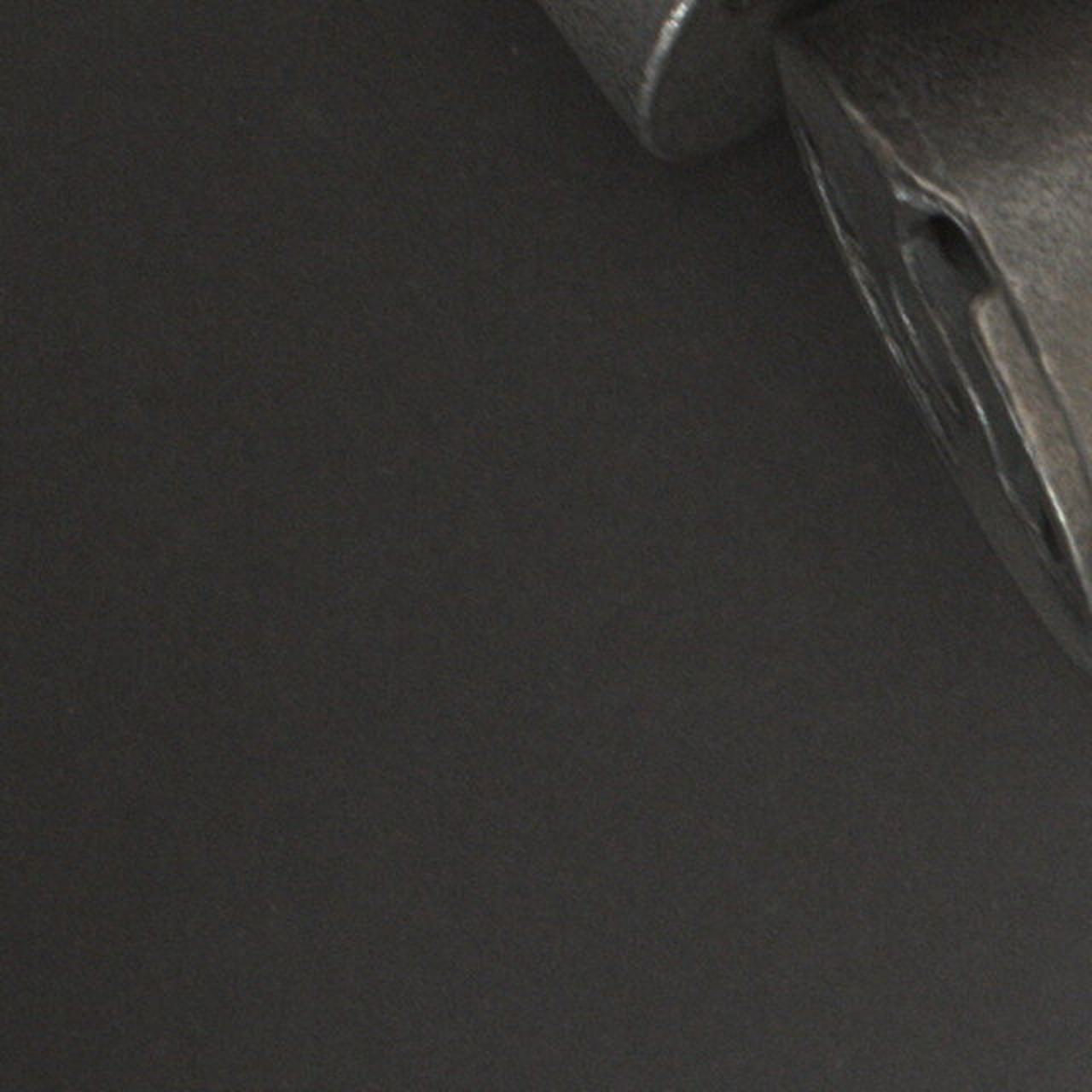

Supplement: Supplemental Information 1 — The CSD-DET dataset was collected from Guangde Hardware Casting Factory and Wuhu Automobile Casting Factory in May 2023. The CSD-DET dataset was used to train and measure the advantages of the DES-YOLO model. This is the filtered partial dataset. [file peerj-cs-10-2224-s001.zip › CastingDefectsDataSet/data/Mr_572.jpg]

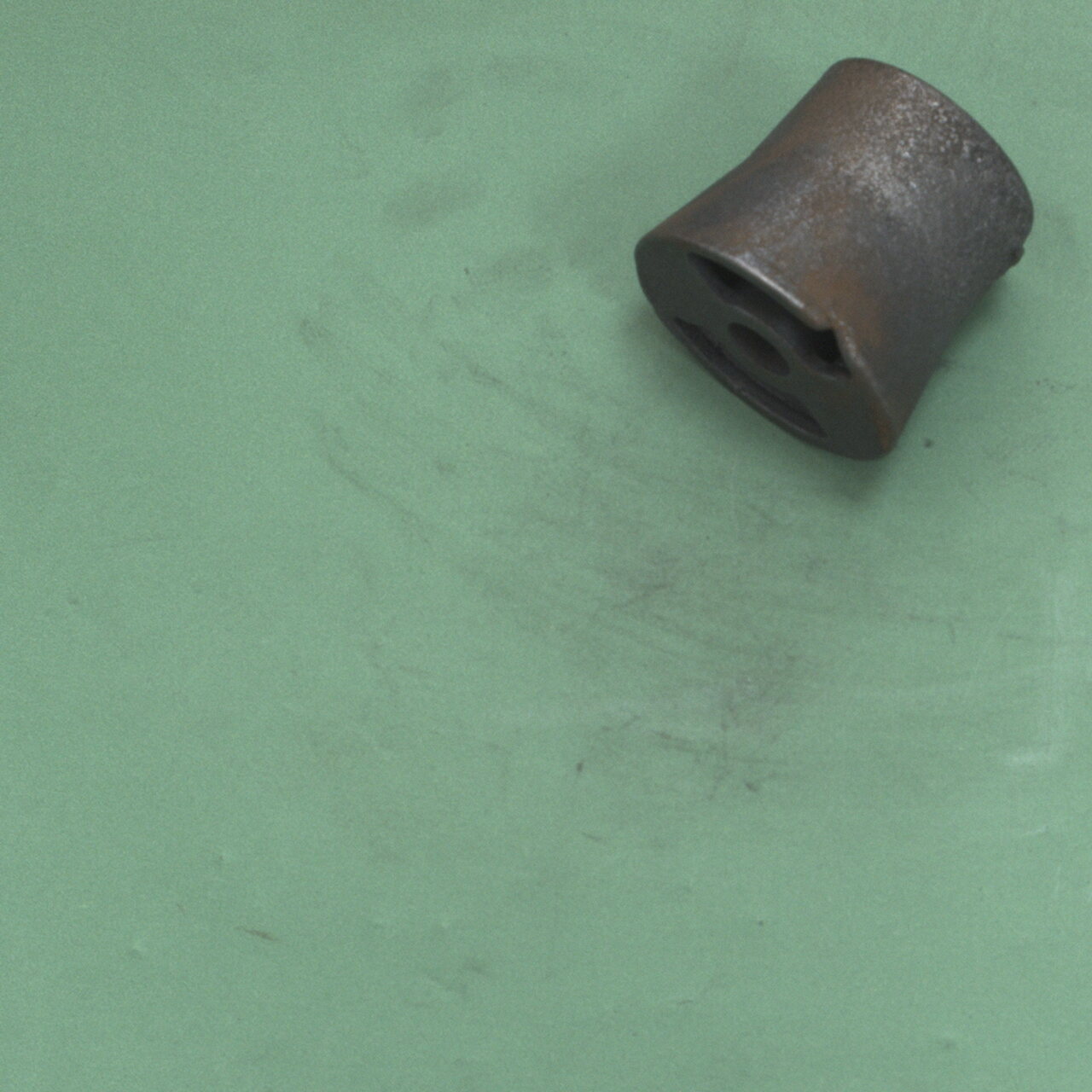

Supplement: Supplemental Information 1 — The CSD-DET dataset was collected from Guangde Hardware Casting Factory and Wuhu Automobile Casting Factory in May 2023. The CSD-DET dataset was used to train and measure the advantages of the DES-YOLO model. This is the filtered partial dataset. [file peerj-cs-10-2224-s001.zip › CastingDefectsDataSet/data/Mr_60.jpg]

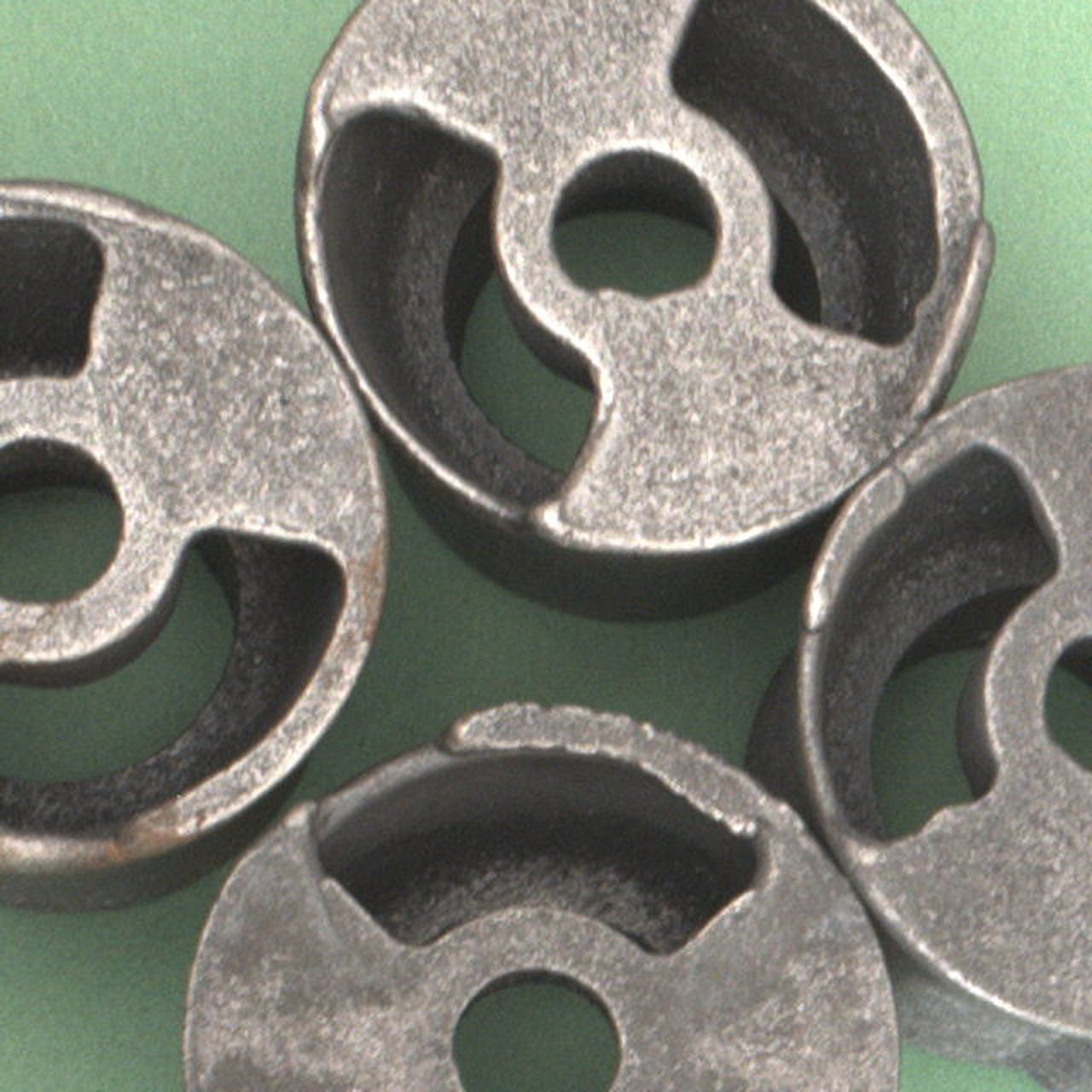

Supplement: Supplemental Information 1 — The CSD-DET dataset was collected from Guangde Hardware Casting Factory and Wuhu Automobile Casting Factory in May 2023. The CSD-DET dataset was used to train and measure the advantages of the DES-YOLO model. This is the filtered partial dataset. [file peerj-cs-10-2224-s001.zip › CastingDefectsDataSet/data/Mr_636.jpg]

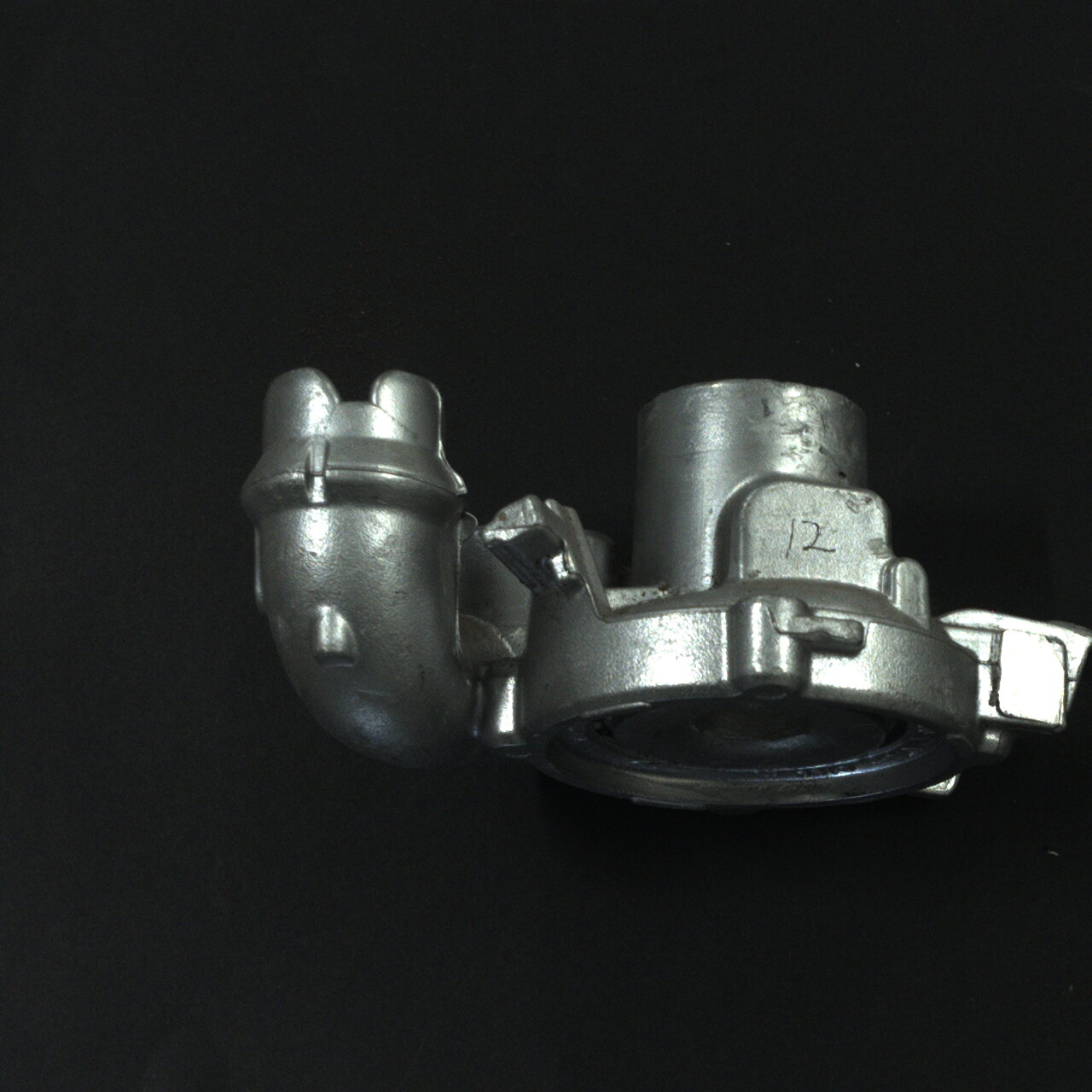

Supplement: Supplemental Information 1 — The CSD-DET dataset was collected from Guangde Hardware Casting Factory and Wuhu Automobile Casting Factory in May 2023. The CSD-DET dataset was used to train and measure the advantages of the DES-YOLO model. This is the filtered partial dataset. [file peerj-cs-10-2224-s001.zip › CastingDefectsDataSet/data/Mr_716.jpg]

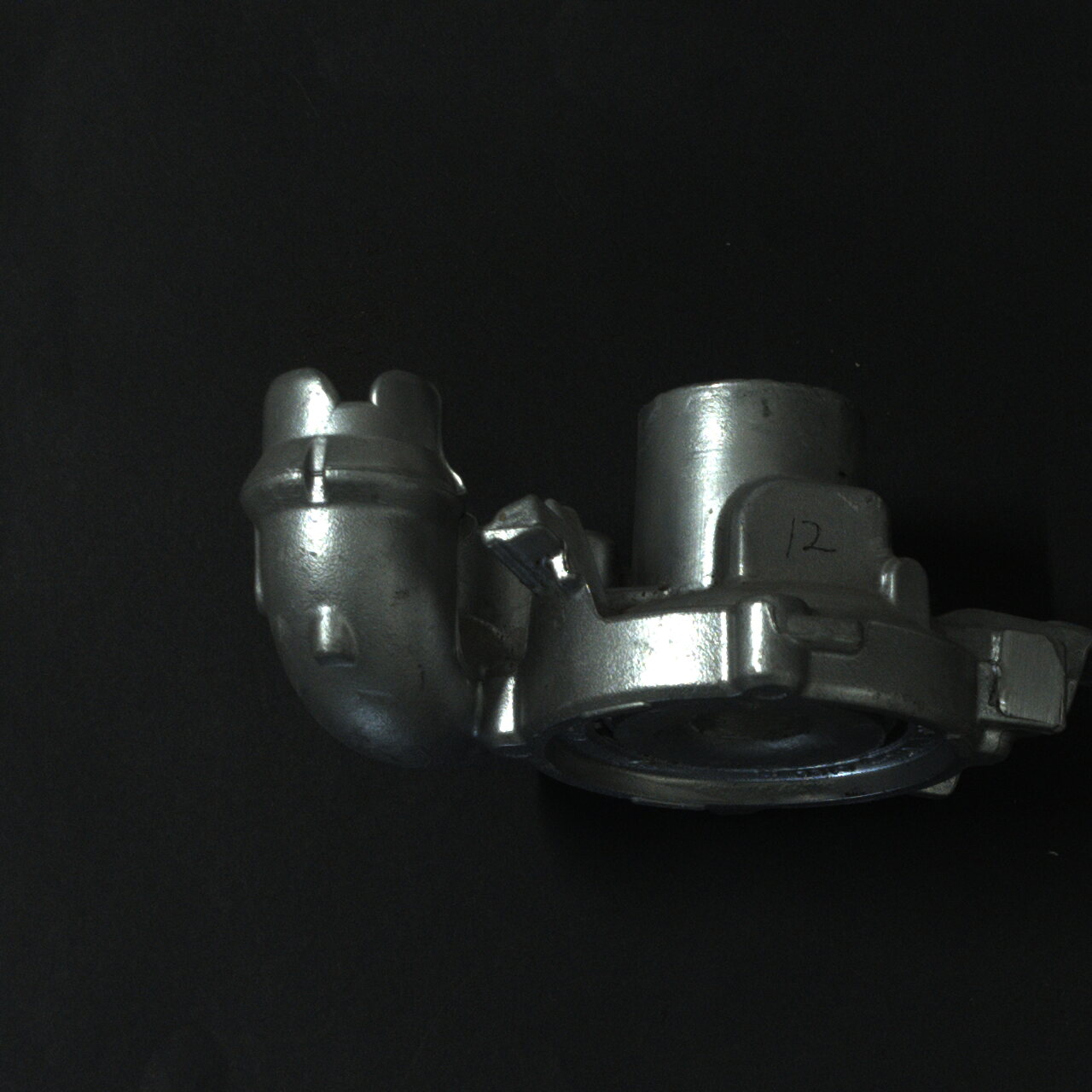

Supplement: Supplemental Information 1 — The CSD-DET dataset was collected from Guangde Hardware Casting Factory and Wuhu Automobile Casting Factory in May 2023. The CSD-DET dataset was used to train and measure the advantages of the DES-YOLO model. This is the filtered partial dataset. [file peerj-cs-10-2224-s001.zip › CastingDefectsDataSet/data/Mr_720.jpg]

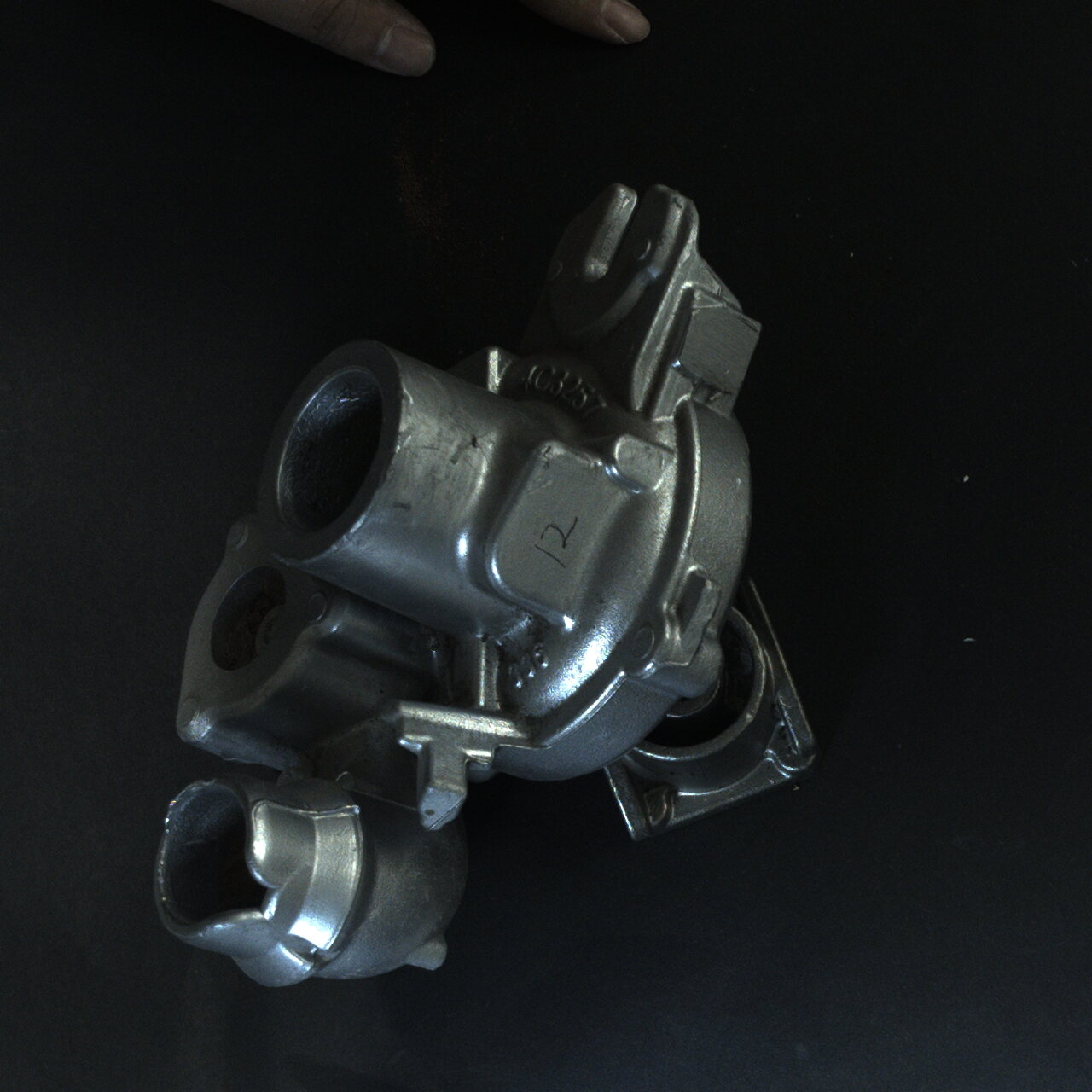

Supplement: Supplemental Information 1 — The CSD-DET dataset was collected from Guangde Hardware Casting Factory and Wuhu Automobile Casting Factory in May 2023. The CSD-DET dataset was used to train and measure the advantages of the DES-YOLO model. This is the filtered partial dataset. [file peerj-cs-10-2224-s001.zip › CastingDefectsDataSet/data/Mr_760.jpg]

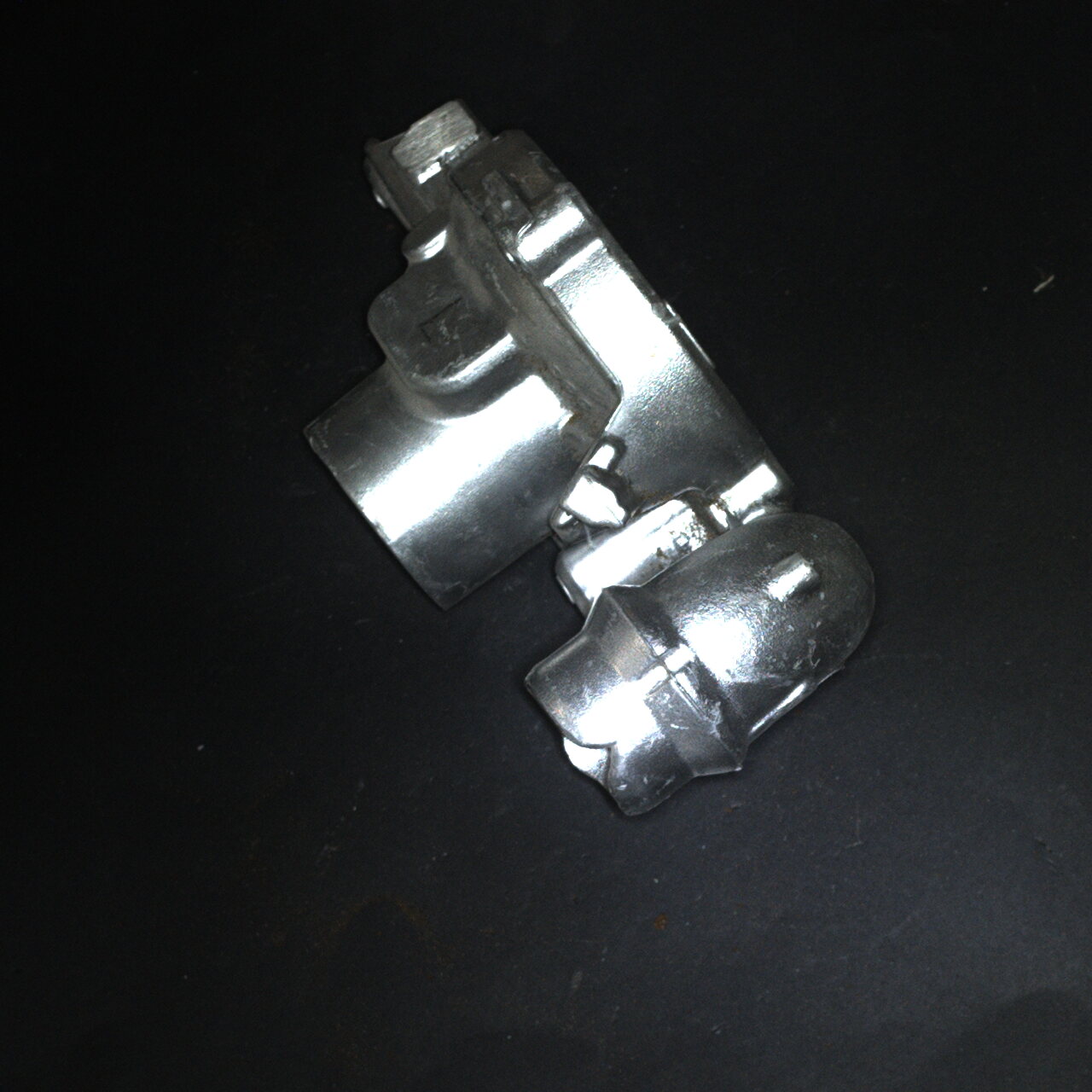

Supplement: Supplemental Information 1 — The CSD-DET dataset was collected from Guangde Hardware Casting Factory and Wuhu Automobile Casting Factory in May 2023. The CSD-DET dataset was used to train and measure the advantages of the DES-YOLO model. This is the filtered partial dataset. [file peerj-cs-10-2224-s001.zip › CastingDefectsDataSet/data/Mr_852.jpg]

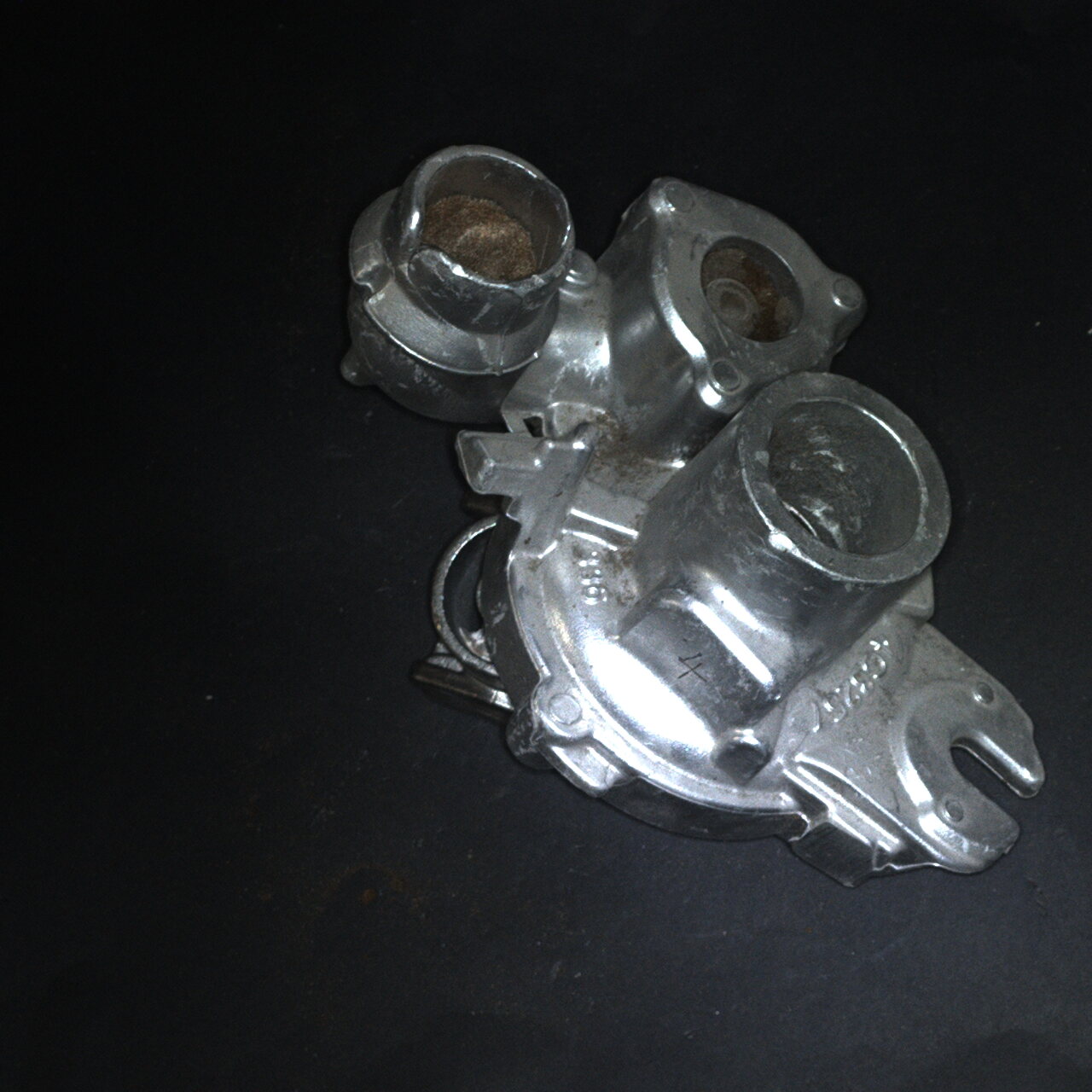

Supplement: Supplemental Information 1 — The CSD-DET dataset was collected from Guangde Hardware Casting Factory and Wuhu Automobile Casting Factory in May 2023. The CSD-DET dataset was used to train and measure the advantages of the DES-YOLO model. This is the filtered partial dataset. [file peerj-cs-10-2224-s001.zip › CastingDefectsDataSet/data/Mr_860.jpg]

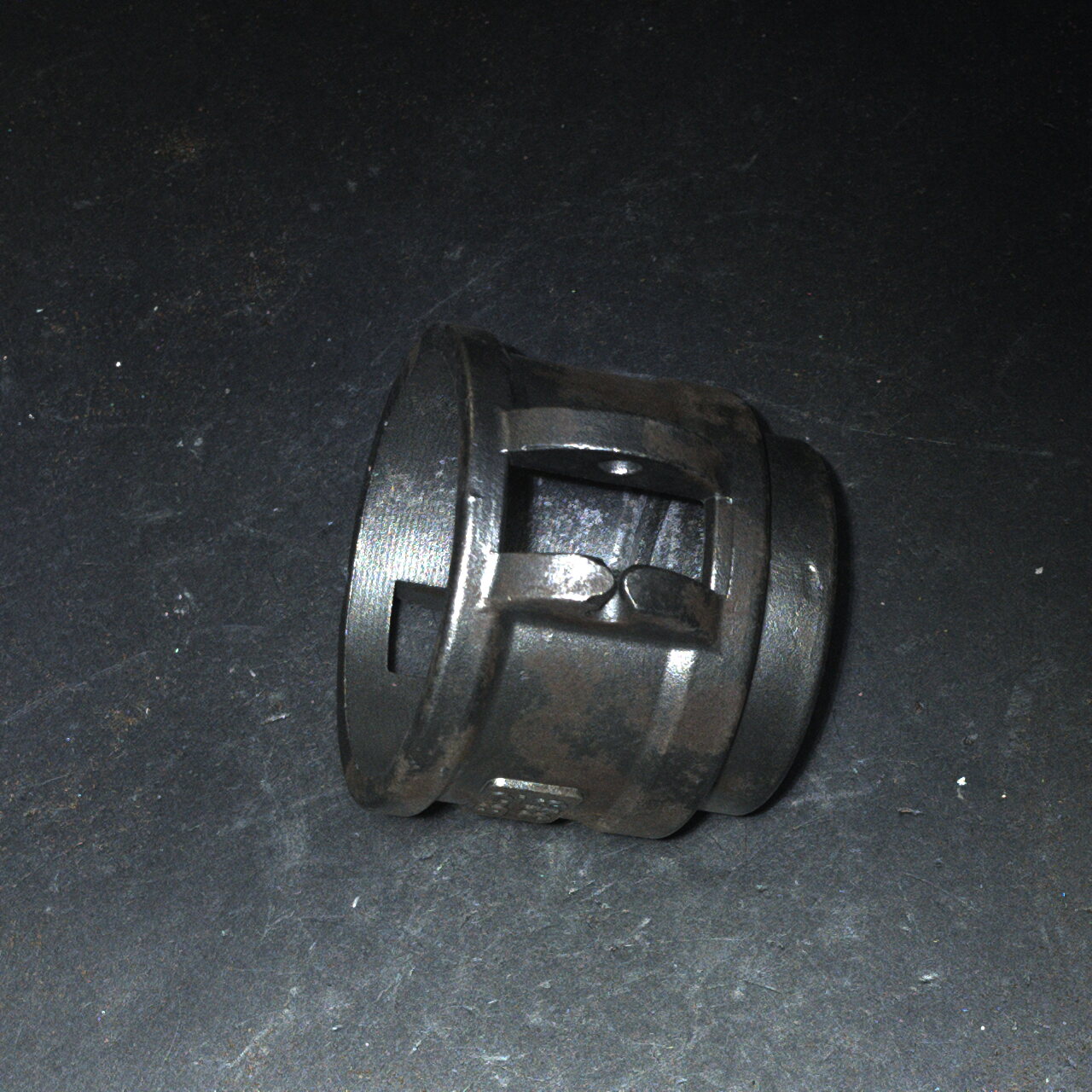

Supplement: Supplemental Information 1 — The CSD-DET dataset was collected from Guangde Hardware Casting Factory and Wuhu Automobile Casting Factory in May 2023. The CSD-DET dataset was used to train and measure the advantages of the DES-YOLO model. This is the filtered partial dataset. [file peerj-cs-10-2224-s001.zip › CastingDefectsDataSet/data/Mr_924.jpg]

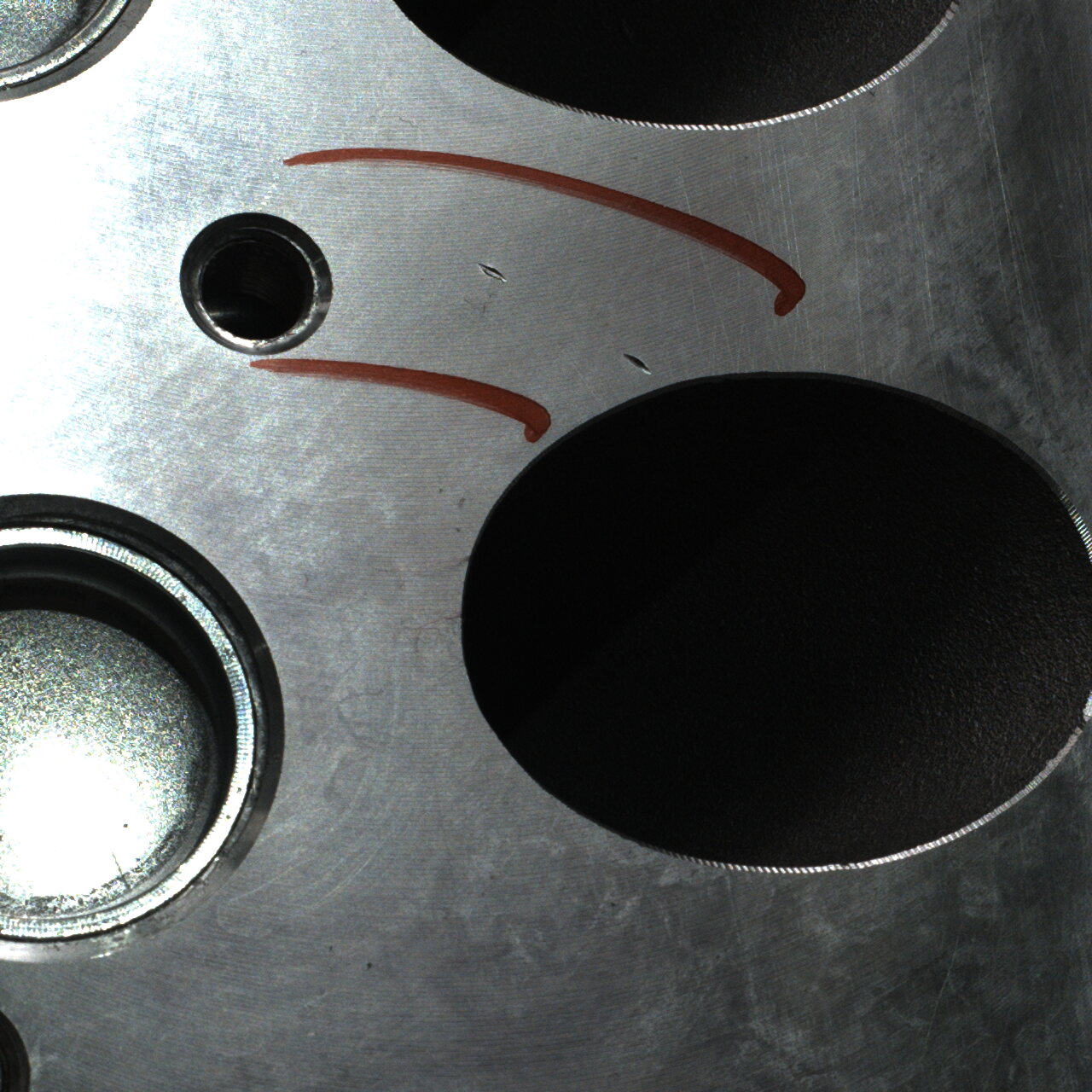

Supplement: Supplemental Information 1 — The CSD-DET dataset was collected from Guangde Hardware Casting Factory and Wuhu Automobile Casting Factory in May 2023. The CSD-DET dataset was used to train and measure the advantages of the DES-YOLO model. This is the filtered partial dataset. [file peerj-cs-10-2224-s001.zip › CastingDefectsDataSet/data/Sc_139.jpg]

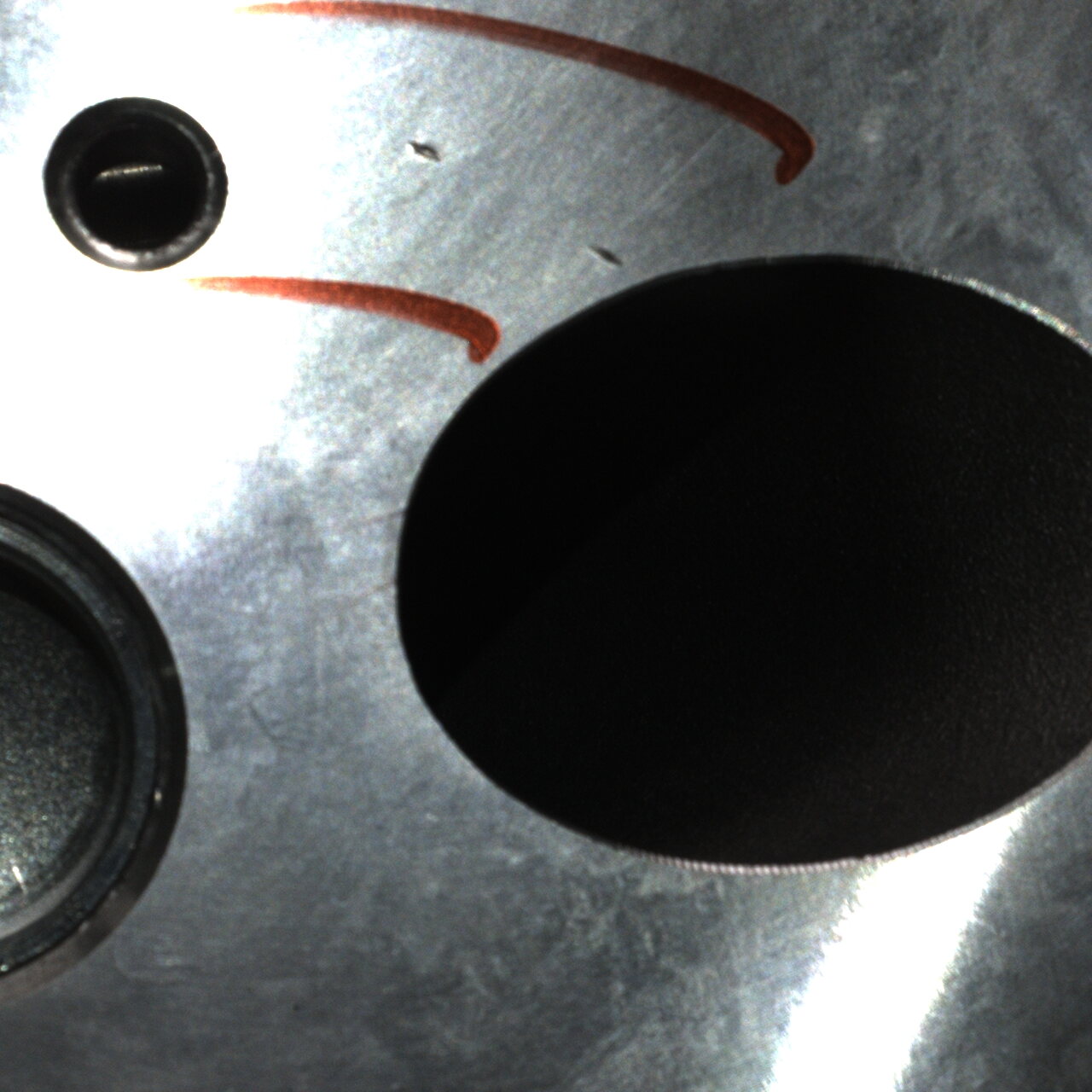

Supplement: Supplemental Information 1 — The CSD-DET dataset was collected from Guangde Hardware Casting Factory and Wuhu Automobile Casting Factory in May 2023. The CSD-DET dataset was used to train and measure the advantages of the DES-YOLO model. This is the filtered partial dataset. [file peerj-cs-10-2224-s001.zip › CastingDefectsDataSet/data/Sc_143.jpg]

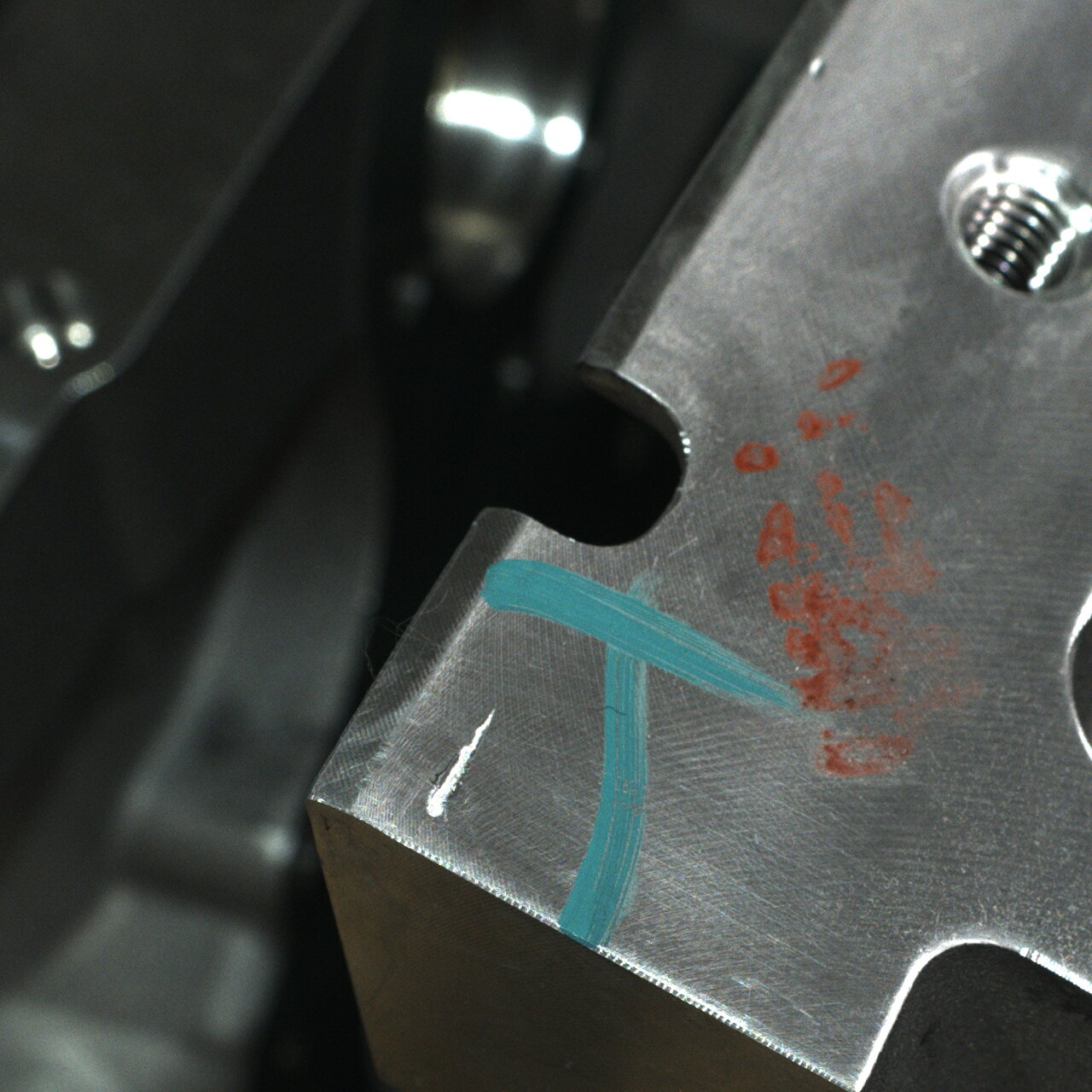

Supplement: Supplemental Information 1 — The CSD-DET dataset was collected from Guangde Hardware Casting Factory and Wuhu Automobile Casting Factory in May 2023. The CSD-DET dataset was used to train and measure the advantages of the DES-YOLO model. This is the filtered partial dataset. [file peerj-cs-10-2224-s001.zip › CastingDefectsDataSet/data/Sc_19.jpg]

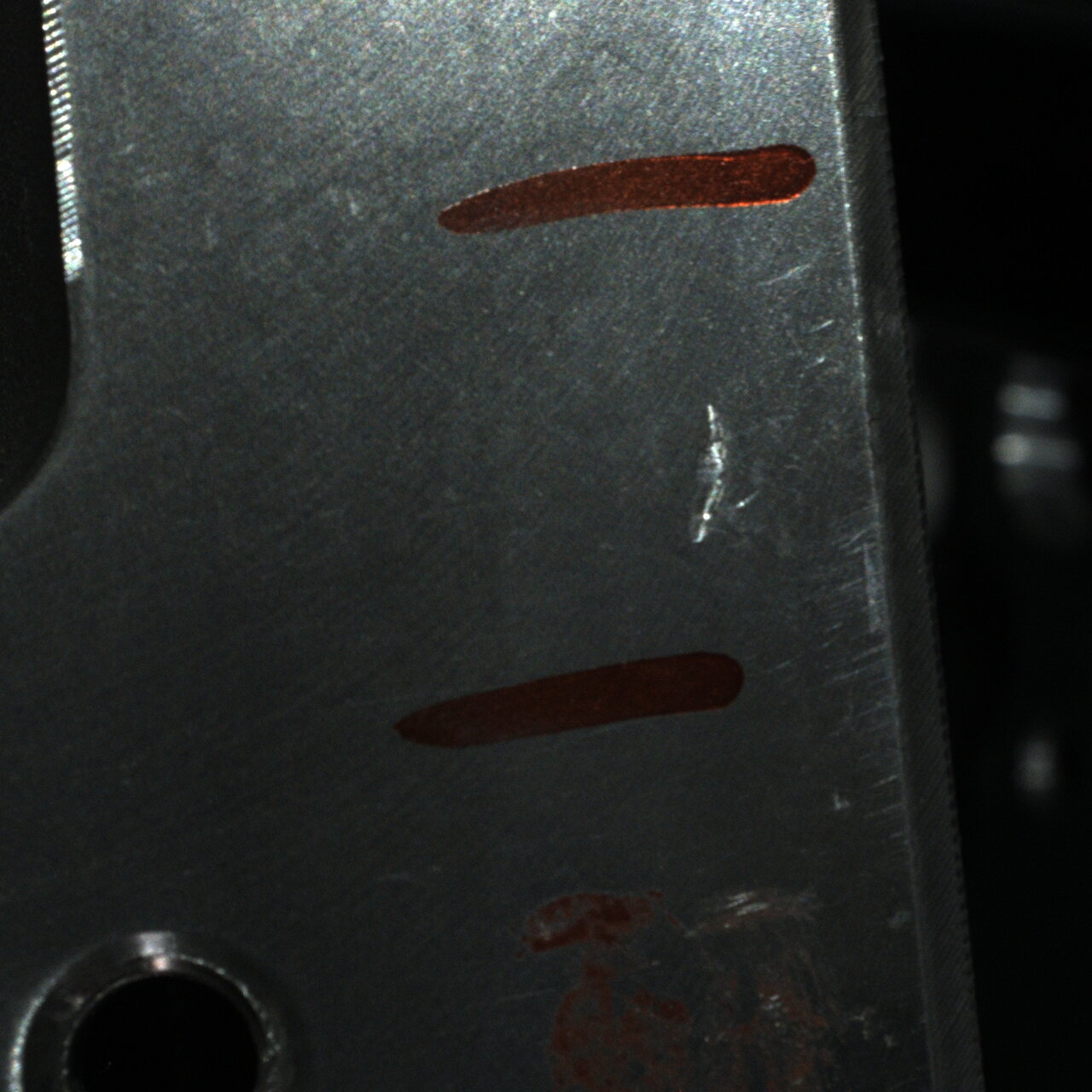

Supplement: Supplemental Information 1 — The CSD-DET dataset was collected from Guangde Hardware Casting Factory and Wuhu Automobile Casting Factory in May 2023. The CSD-DET dataset was used to train and measure the advantages of the DES-YOLO model. This is the filtered partial dataset. [file peerj-cs-10-2224-s001.zip › CastingDefectsDataSet/data/Sc_207.jpg]

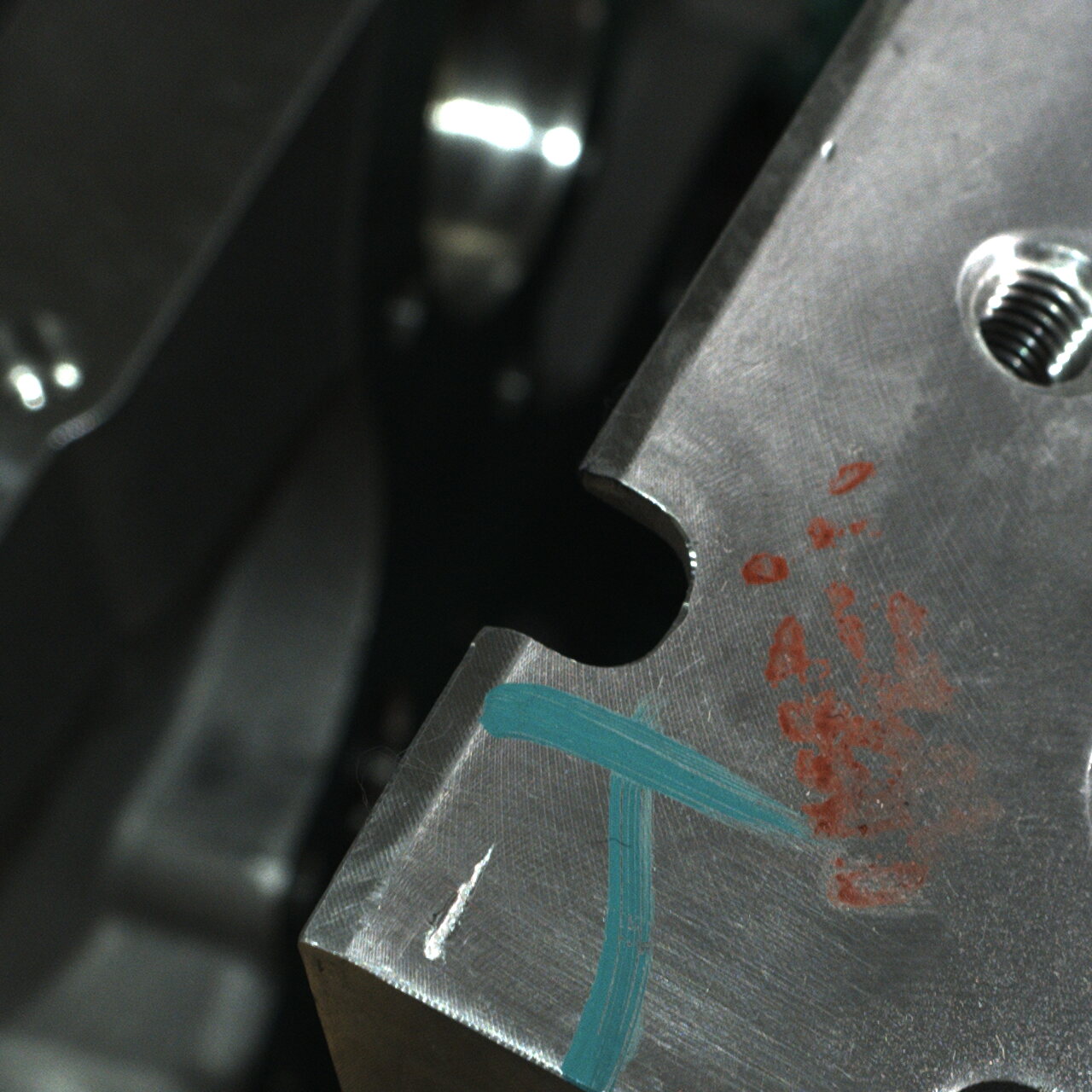

Supplement: Supplemental Information 1 — The CSD-DET dataset was collected from Guangde Hardware Casting Factory and Wuhu Automobile Casting Factory in May 2023. The CSD-DET dataset was used to train and measure the advantages of the DES-YOLO model. This is the filtered partial dataset. [file peerj-cs-10-2224-s001.zip › CastingDefectsDataSet/data/Sc_23.jpg]

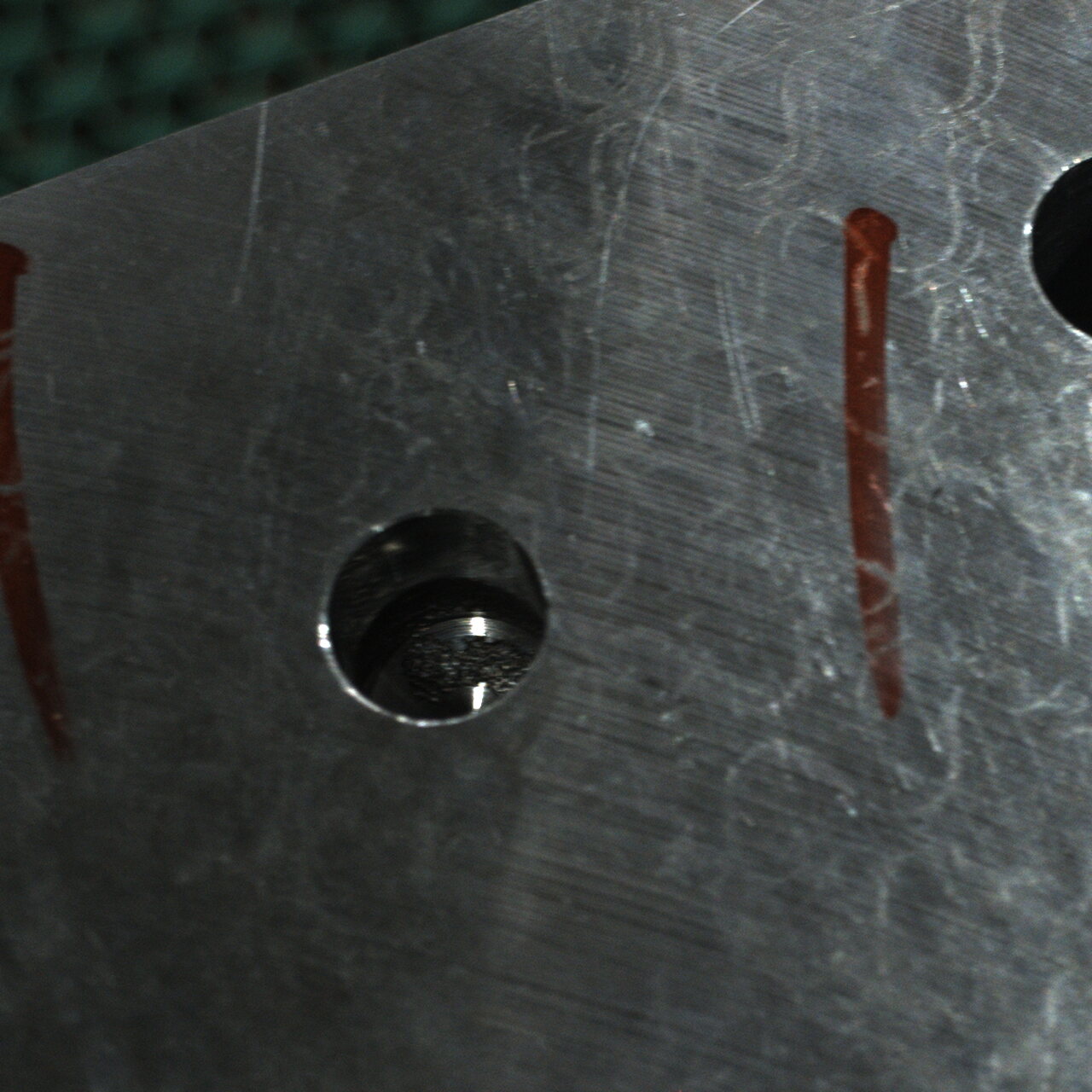

Supplement: Supplemental Information 1 — The CSD-DET dataset was collected from Guangde Hardware Casting Factory and Wuhu Automobile Casting Factory in May 2023. The CSD-DET dataset was used to train and measure the advantages of the DES-YOLO model. This is the filtered partial dataset. [file peerj-cs-10-2224-s001.zip › CastingDefectsDataSet/data/Sc_287.jpg]

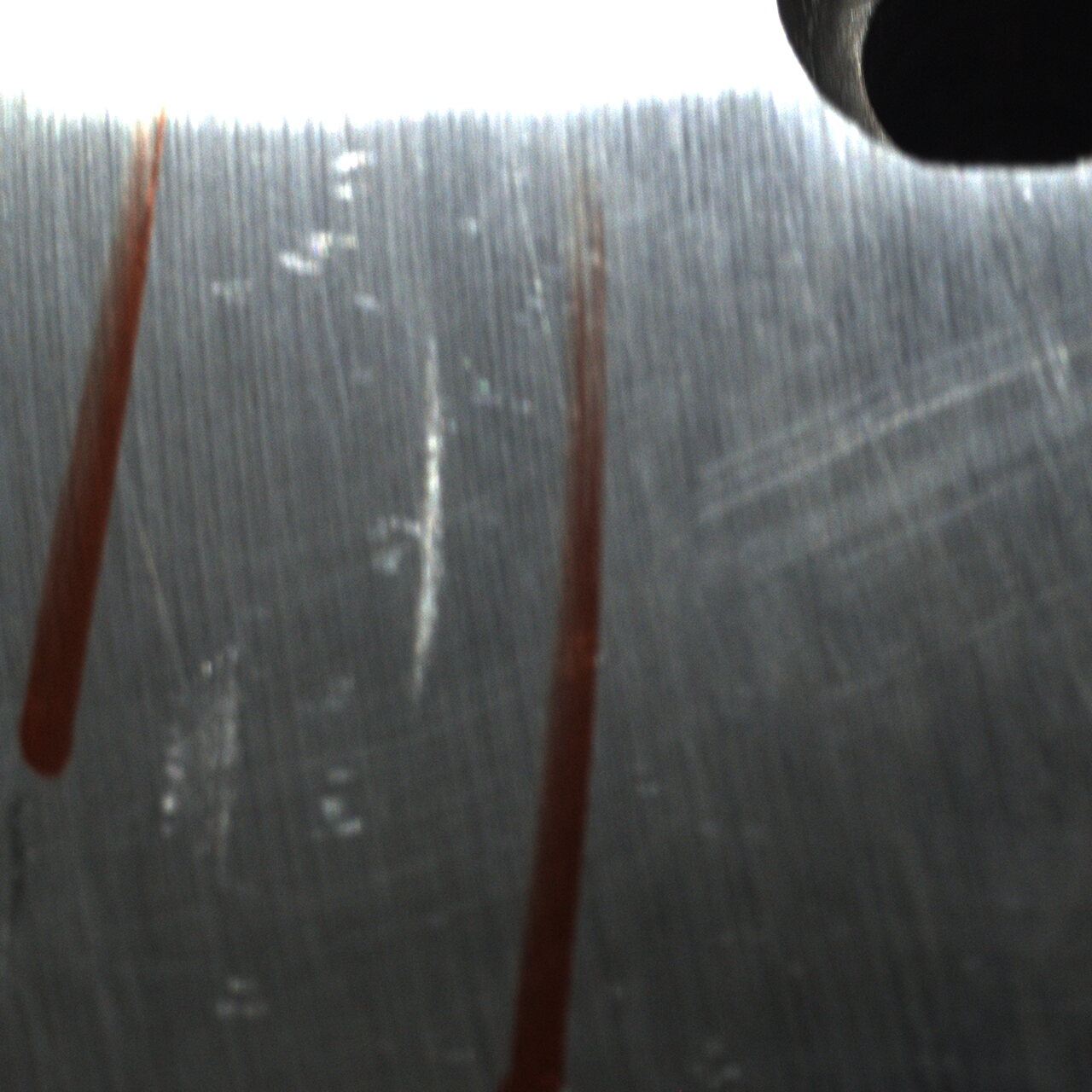

Supplement: Supplemental Information 1 — The CSD-DET dataset was collected from Guangde Hardware Casting Factory and Wuhu Automobile Casting Factory in May 2023. The CSD-DET dataset was used to train and measure the advantages of the DES-YOLO model. This is the filtered partial dataset. [file peerj-cs-10-2224-s001.zip › CastingDefectsDataSet/data/Sc_307.jpg]

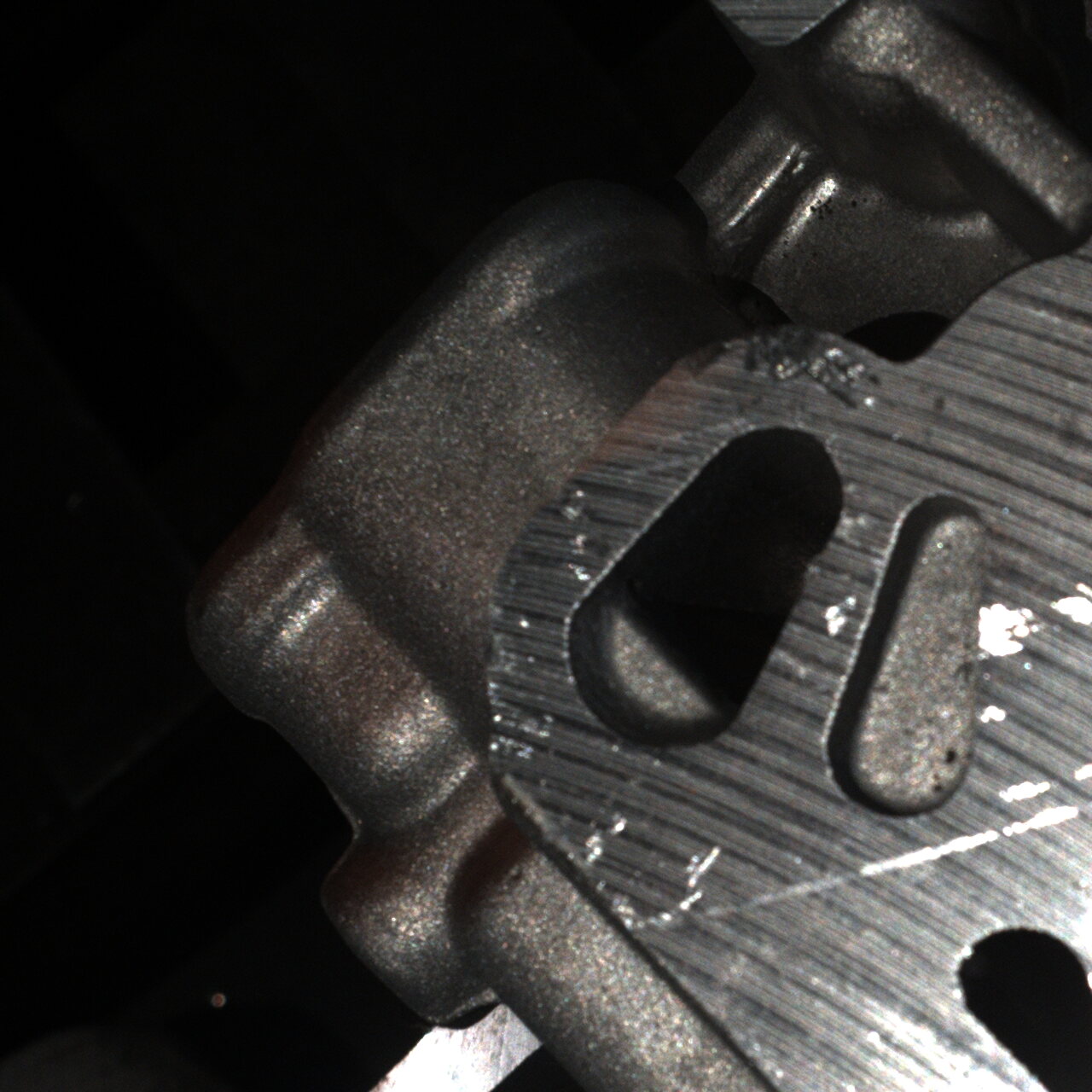

Supplement: Supplemental Information 1 — The CSD-DET dataset was collected from Guangde Hardware Casting Factory and Wuhu Automobile Casting Factory in May 2023. The CSD-DET dataset was used to train and measure the advantages of the DES-YOLO model. This is the filtered partial dataset. [file peerj-cs-10-2224-s001.zip › CastingDefectsDataSet/data/Sc_347.jpg]

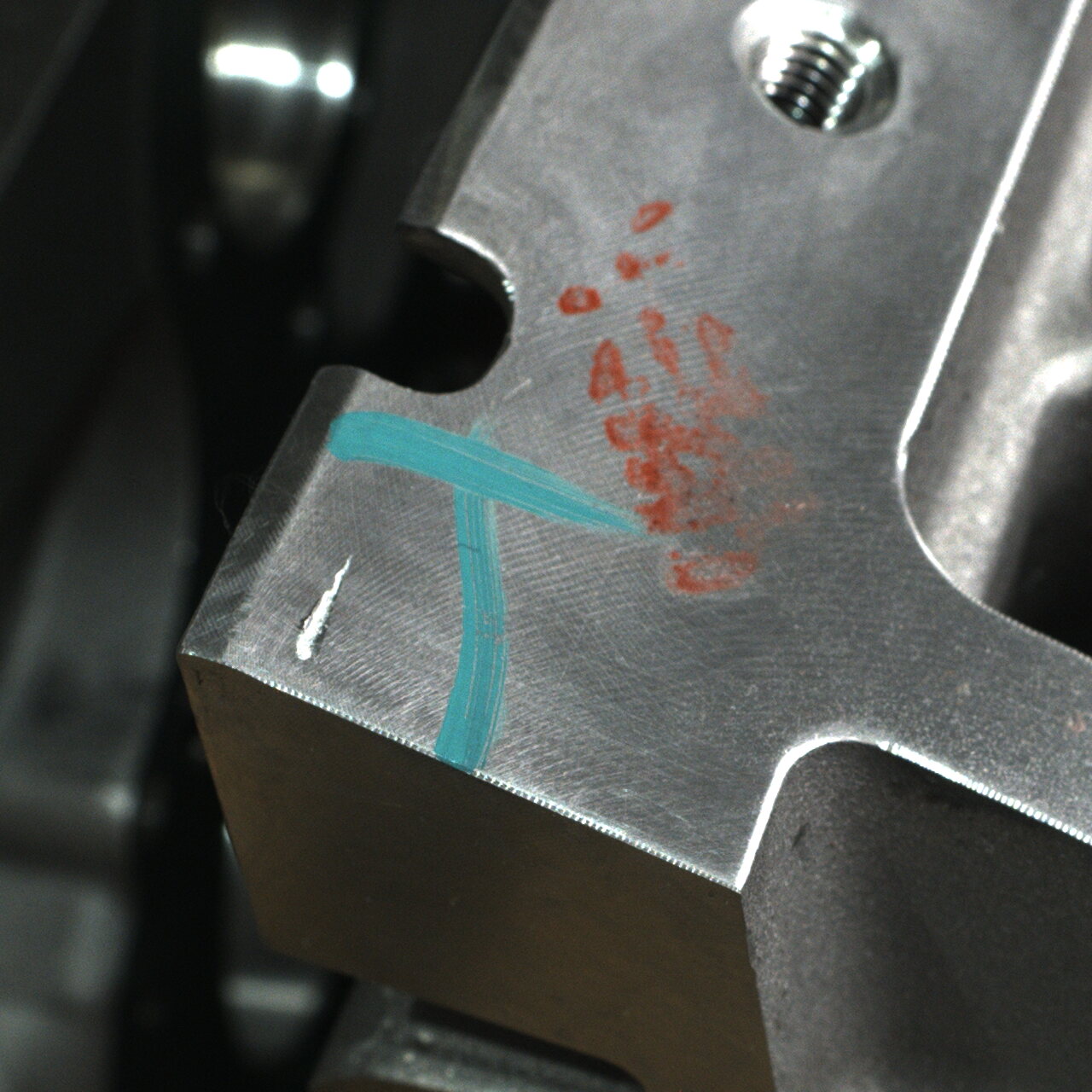

Supplement: Supplemental Information 1 — The CSD-DET dataset was collected from Guangde Hardware Casting Factory and Wuhu Automobile Casting Factory in May 2023. The CSD-DET dataset was used to train and measure the advantages of the DES-YOLO model. This is the filtered partial dataset. [file peerj-cs-10-2224-s001.zip › CastingDefectsDataSet/data/Sc_35.jpg]

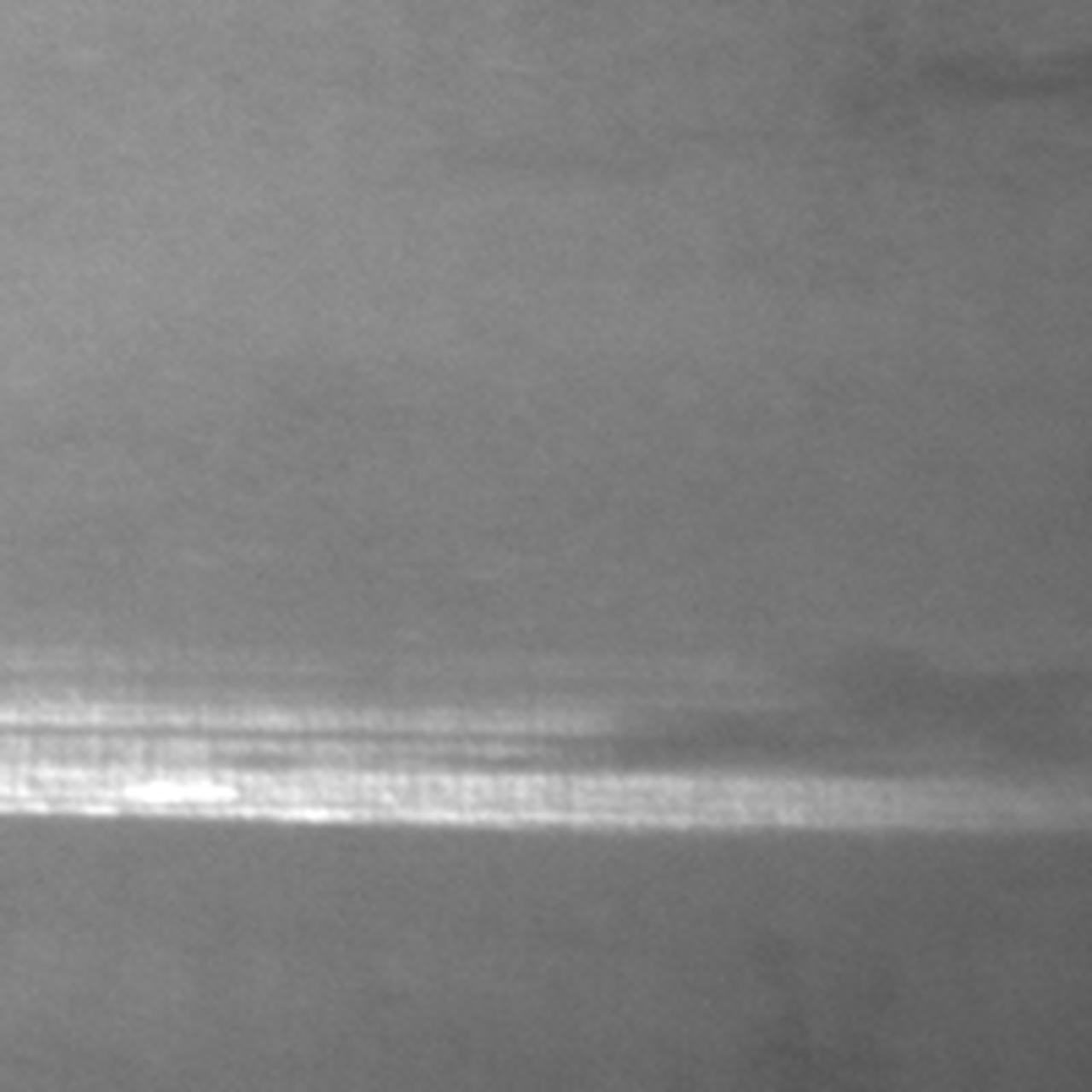

Supplement: Supplemental Information 1 — The CSD-DET dataset was collected from Guangde Hardware Casting Factory and Wuhu Automobile Casting Factory in May 2023. The CSD-DET dataset was used to train and measure the advantages of the DES-YOLO model. This is the filtered partial dataset. [file peerj-cs-10-2224-s001.zip › CastingDefectsDataSet/data/Sc_423.jpg]

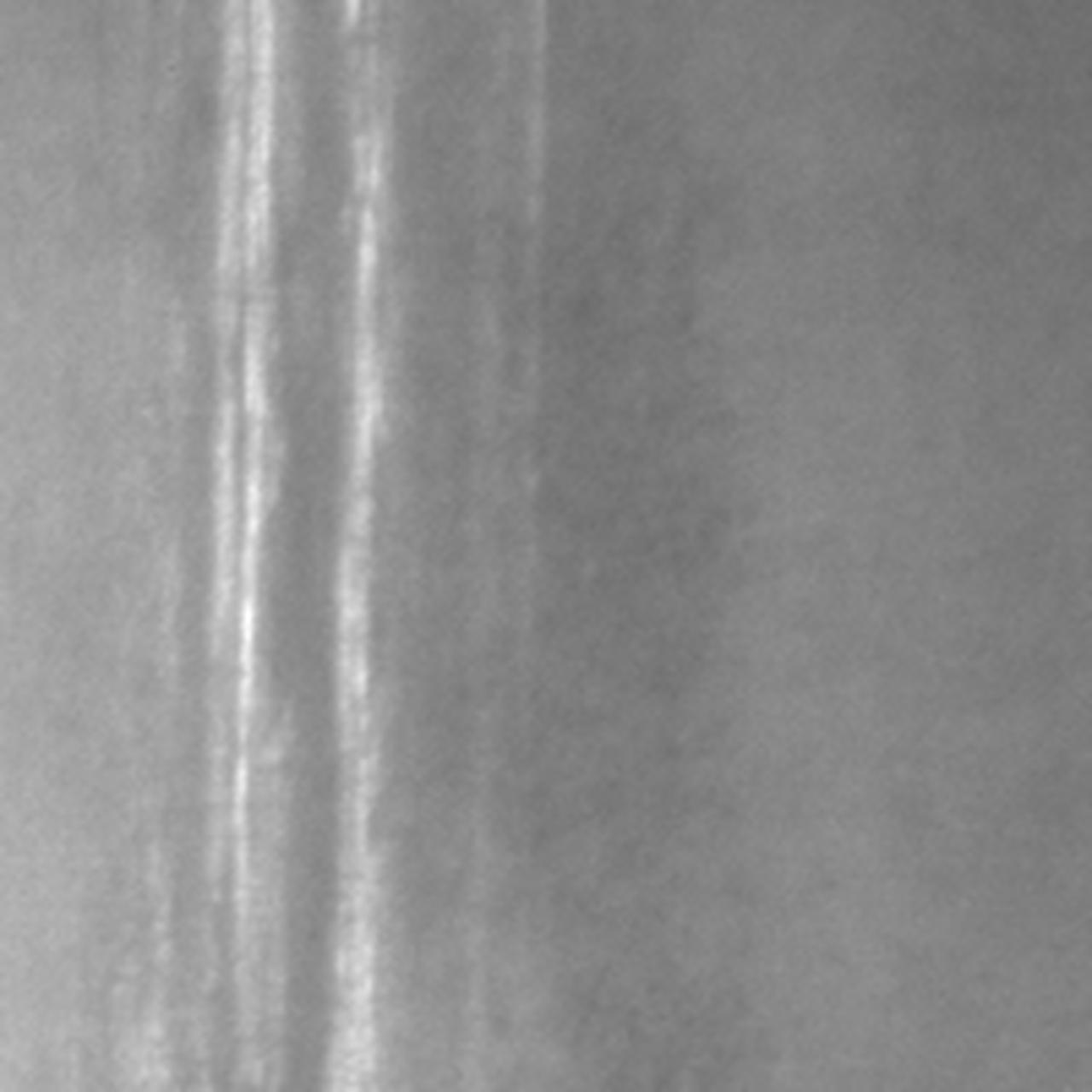

Supplement: Supplemental Information 1 — The CSD-DET dataset was collected from Guangde Hardware Casting Factory and Wuhu Automobile Casting Factory in May 2023. The CSD-DET dataset was used to train and measure the advantages of the DES-YOLO model. This is the filtered partial dataset. [file peerj-cs-10-2224-s001.zip › CastingDefectsDataSet/data/Sc_427.jpg]

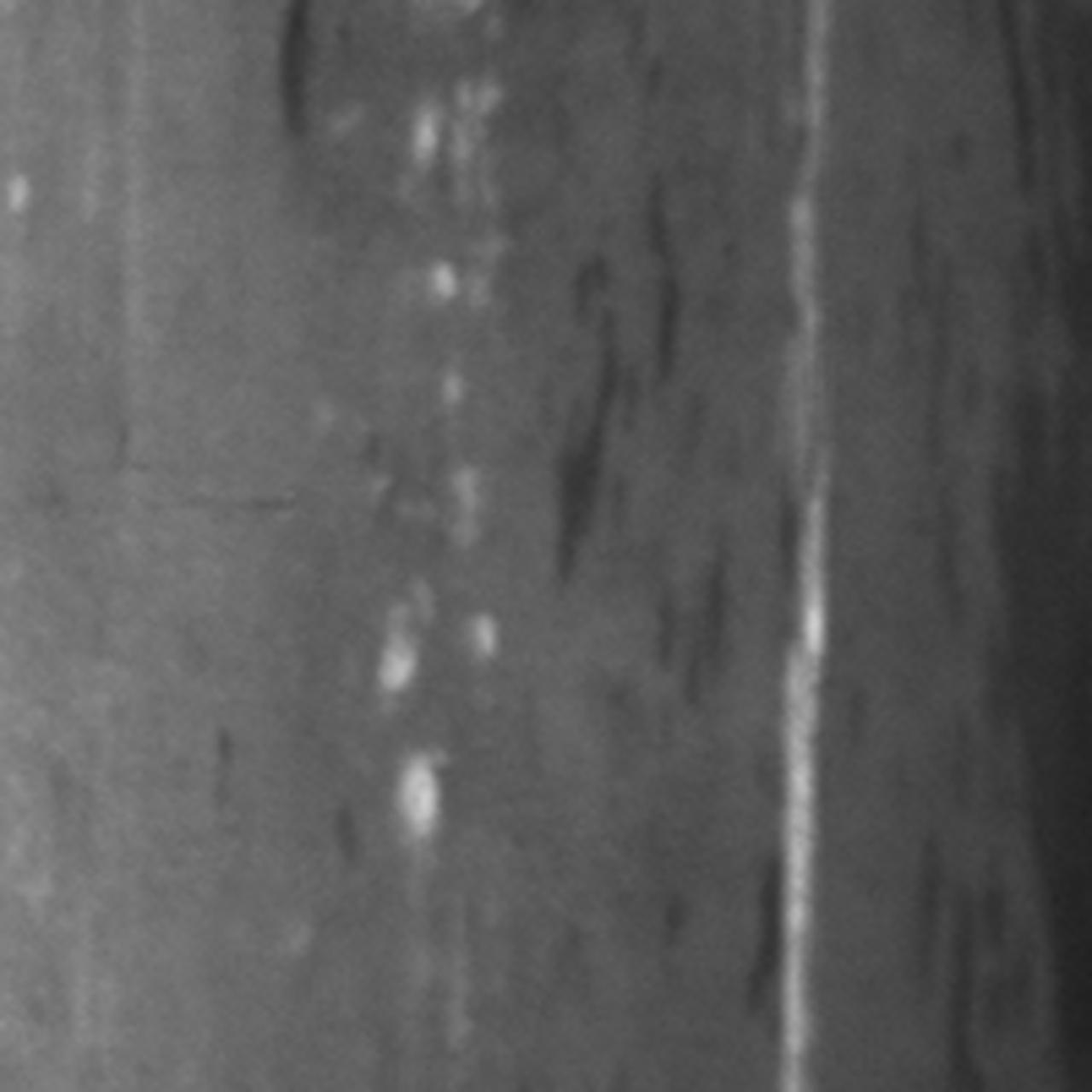

Supplement: Supplemental Information 1 — The CSD-DET dataset was collected from Guangde Hardware Casting Factory and Wuhu Automobile Casting Factory in May 2023. The CSD-DET dataset was used to train and measure the advantages of the DES-YOLO model. This is the filtered partial dataset. [file peerj-cs-10-2224-s001.zip › CastingDefectsDataSet/data/Sc_491.jpg]

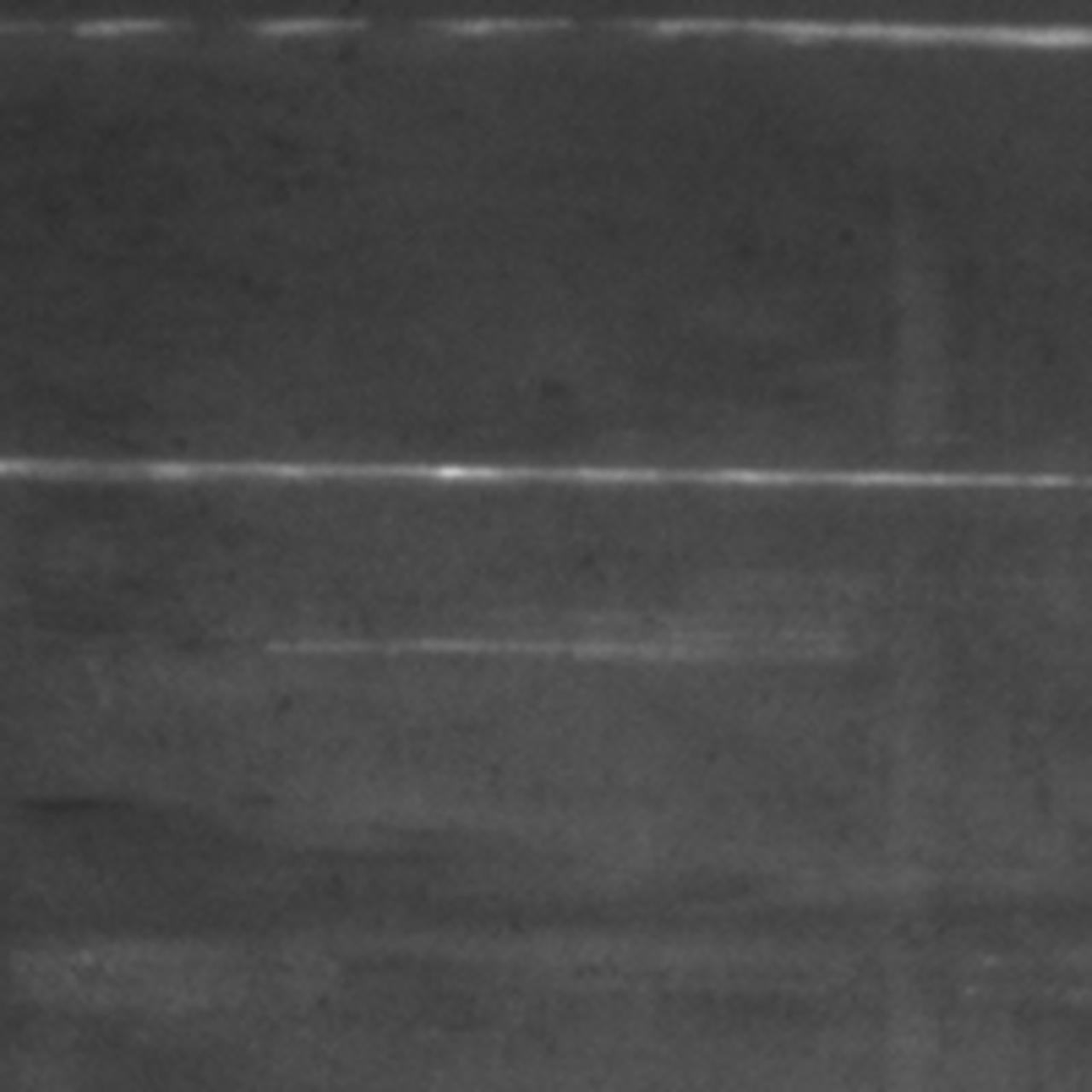

Supplement: Supplemental Information 1 — The CSD-DET dataset was collected from Guangde Hardware Casting Factory and Wuhu Automobile Casting Factory in May 2023. The CSD-DET dataset was used to train and measure the advantages of the DES-YOLO model. This is the filtered partial dataset. [file peerj-cs-10-2224-s001.zip › CastingDefectsDataSet/data/Sc_595.jpg]

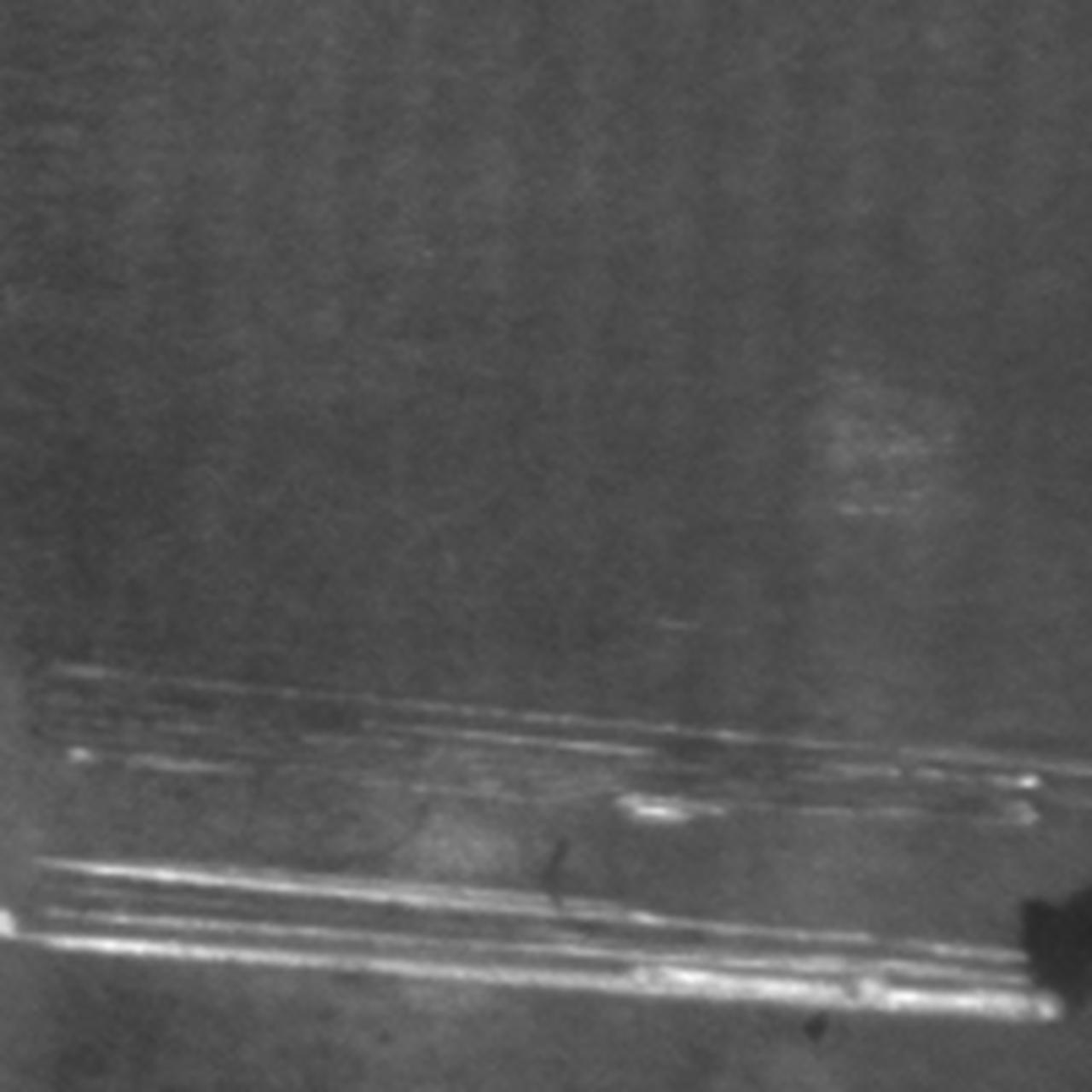

Supplement: Supplemental Information 1 — The CSD-DET dataset was collected from Guangde Hardware Casting Factory and Wuhu Automobile Casting Factory in May 2023. The CSD-DET dataset was used to train and measure the advantages of the DES-YOLO model. This is the filtered partial dataset. [file peerj-cs-10-2224-s001.zip › CastingDefectsDataSet/data/Sc_611.jpg]

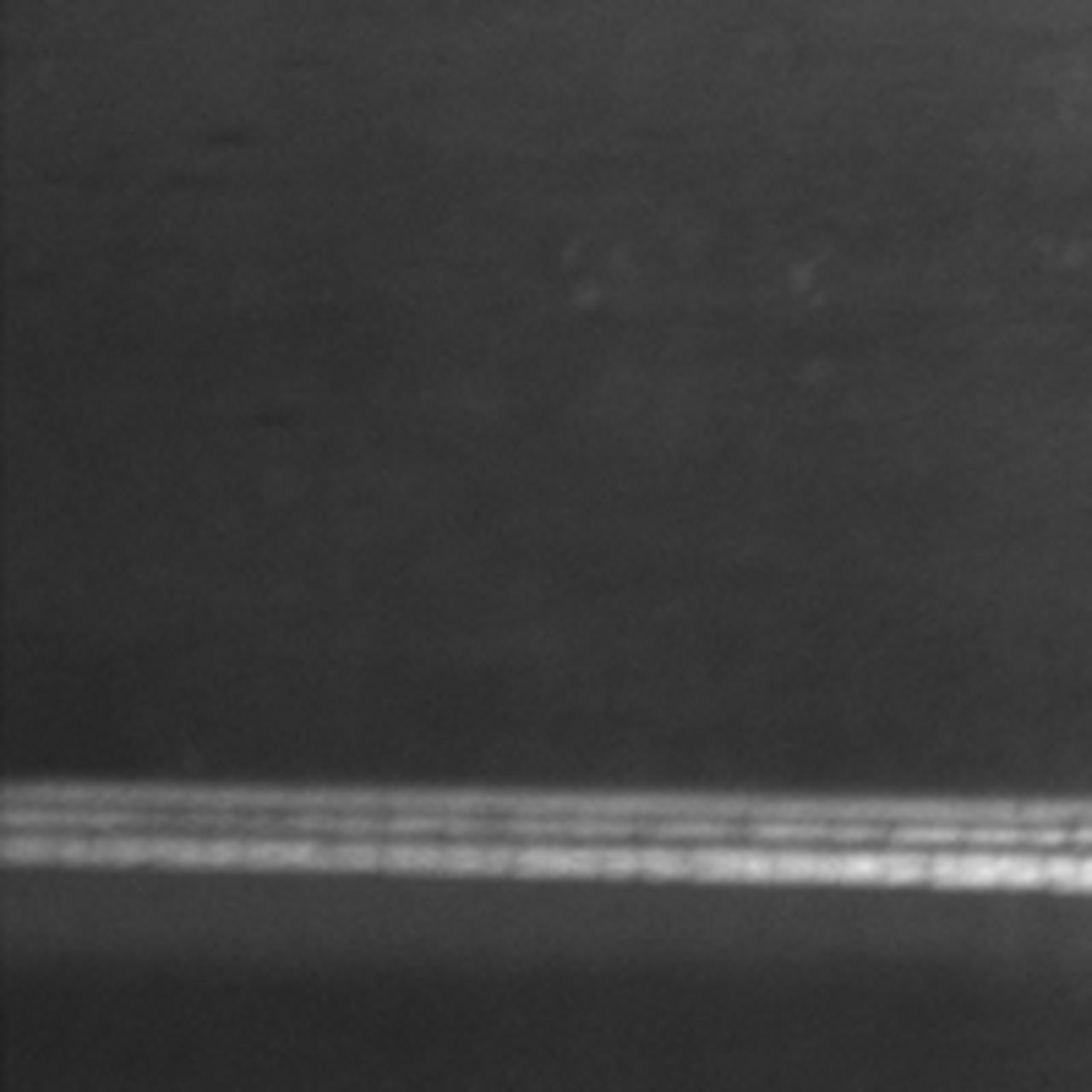

Supplement: Supplemental Information 1 — The CSD-DET dataset was collected from Guangde Hardware Casting Factory and Wuhu Automobile Casting Factory in May 2023. The CSD-DET dataset was used to train and measure the advantages of the DES-YOLO model. This is the filtered partial dataset. [file peerj-cs-10-2224-s001.zip › CastingDefectsDataSet/data/Sc_615.jpg]

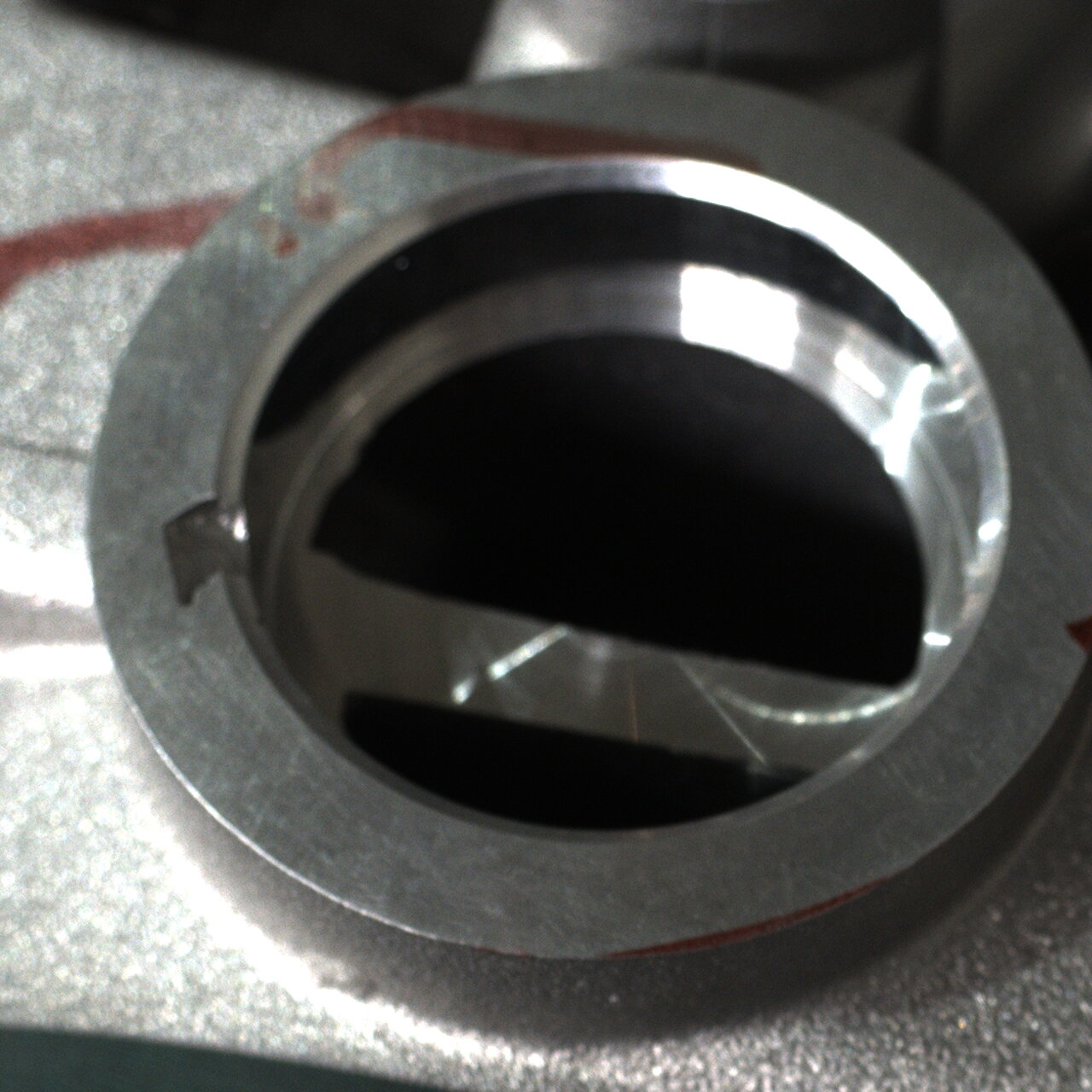

Supplement: Supplemental Information 1 — The CSD-DET dataset was collected from Guangde Hardware Casting Factory and Wuhu Automobile Casting Factory in May 2023. The CSD-DET dataset was used to train and measure the advantages of the DES-YOLO model. This is the filtered partial dataset. [file peerj-cs-10-2224-s001.zip › CastingDefectsDataSet/data/Sh_127.jpg]

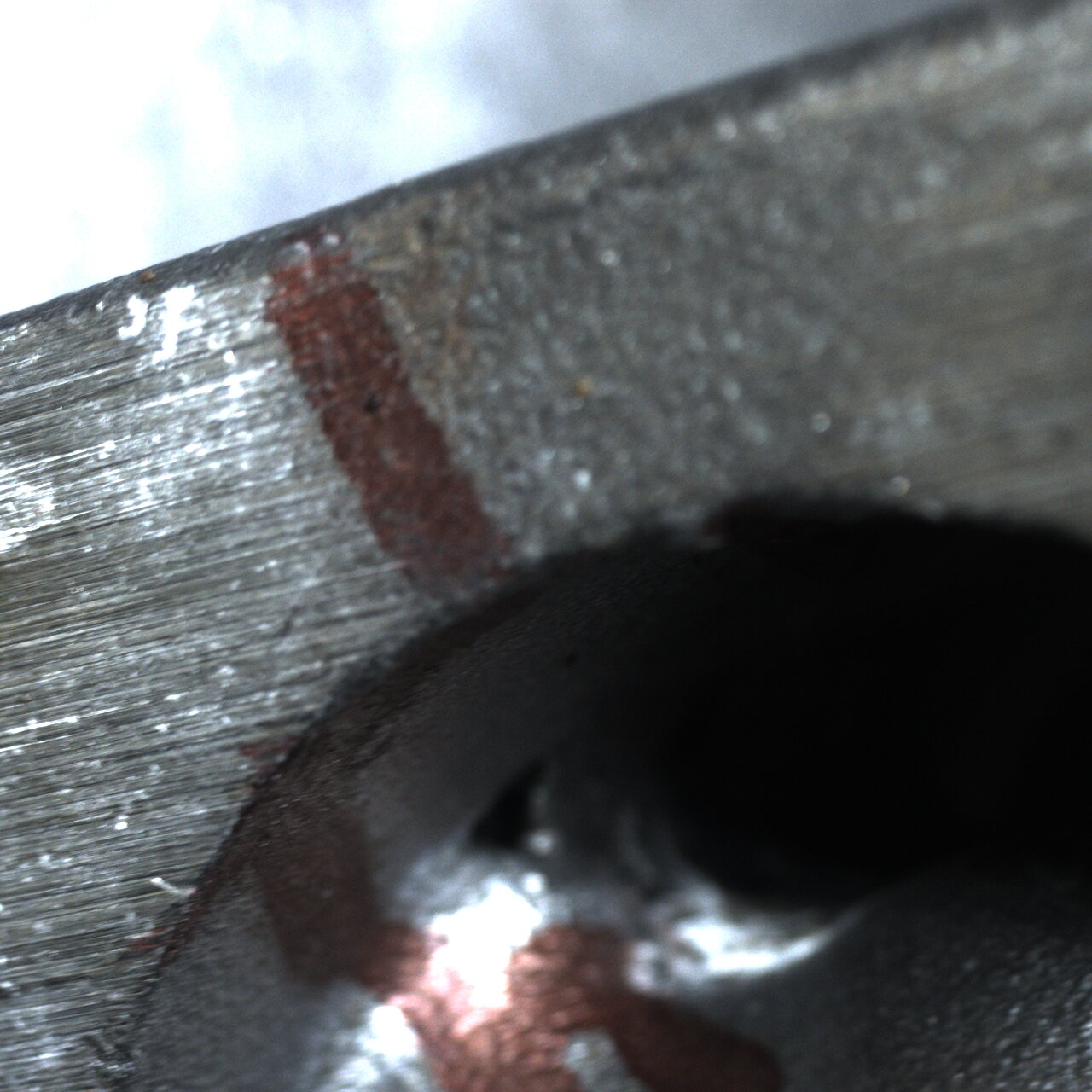

Supplement: Supplemental Information 1 — The CSD-DET dataset was collected from Guangde Hardware Casting Factory and Wuhu Automobile Casting Factory in May 2023. The CSD-DET dataset was used to train and measure the advantages of the DES-YOLO model. This is the filtered partial dataset. [file peerj-cs-10-2224-s001.zip › CastingDefectsDataSet/data/Sh_223.jpg]

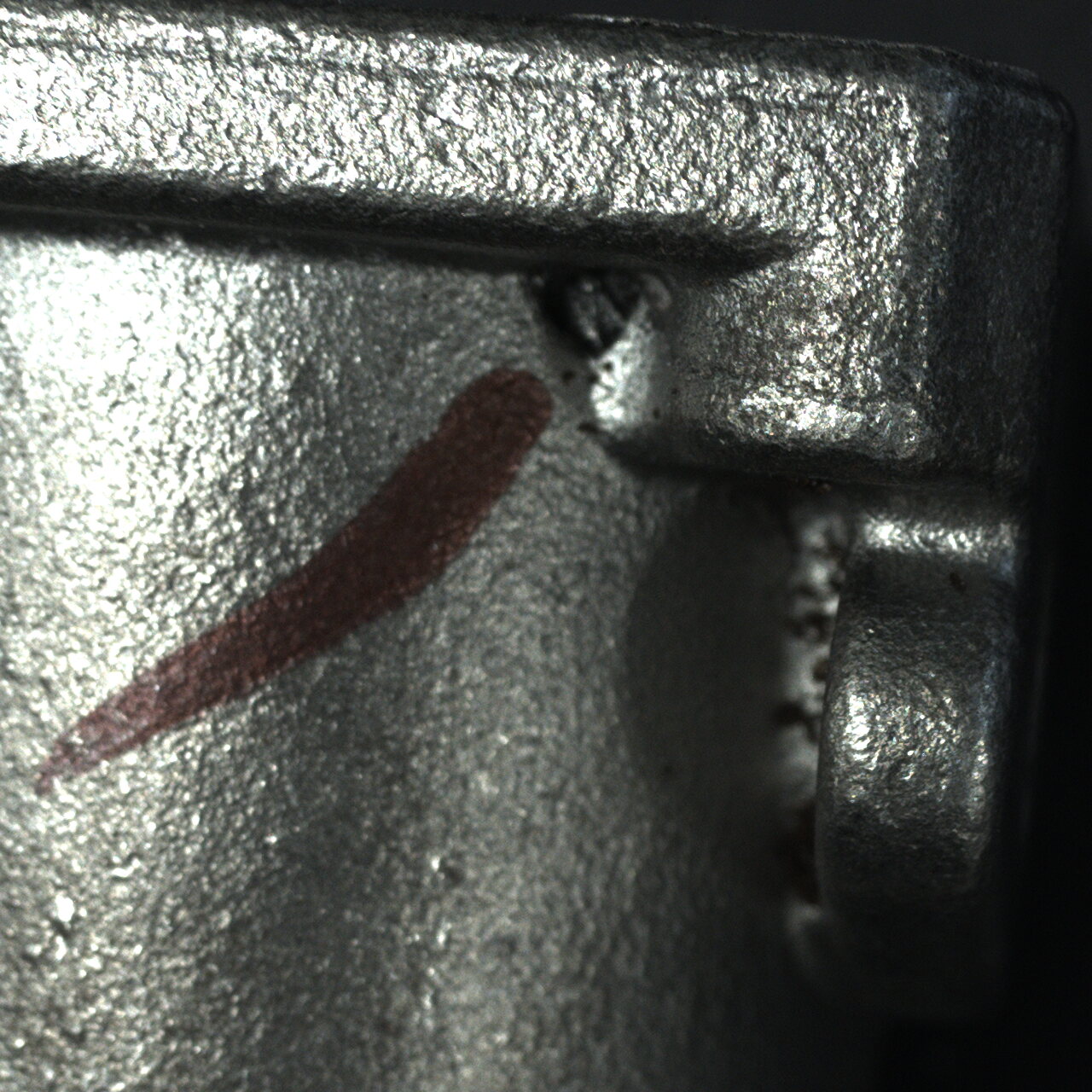

Supplement: Supplemental Information 1 — The CSD-DET dataset was collected from Guangde Hardware Casting Factory and Wuhu Automobile Casting Factory in May 2023. The CSD-DET dataset was used to train and measure the advantages of the DES-YOLO model. This is the filtered partial dataset. [file peerj-cs-10-2224-s001.zip › CastingDefectsDataSet/data/Sh_227.jpg]

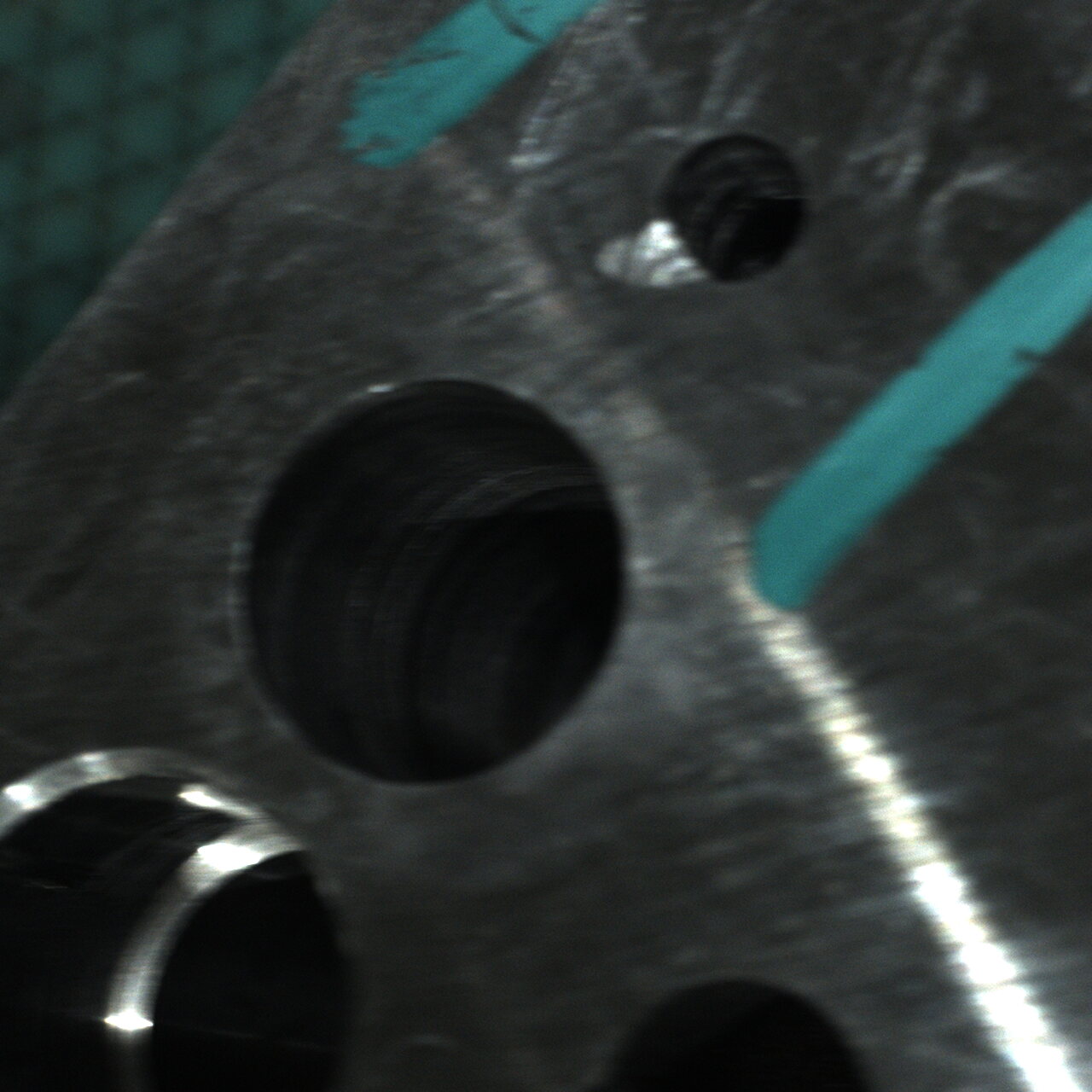

Supplement: Supplemental Information 1 — The CSD-DET dataset was collected from Guangde Hardware Casting Factory and Wuhu Automobile Casting Factory in May 2023. The CSD-DET dataset was used to train and measure the advantages of the DES-YOLO model. This is the filtered partial dataset. [file peerj-cs-10-2224-s001.zip › CastingDefectsDataSet/data/Sh_263.jpg]

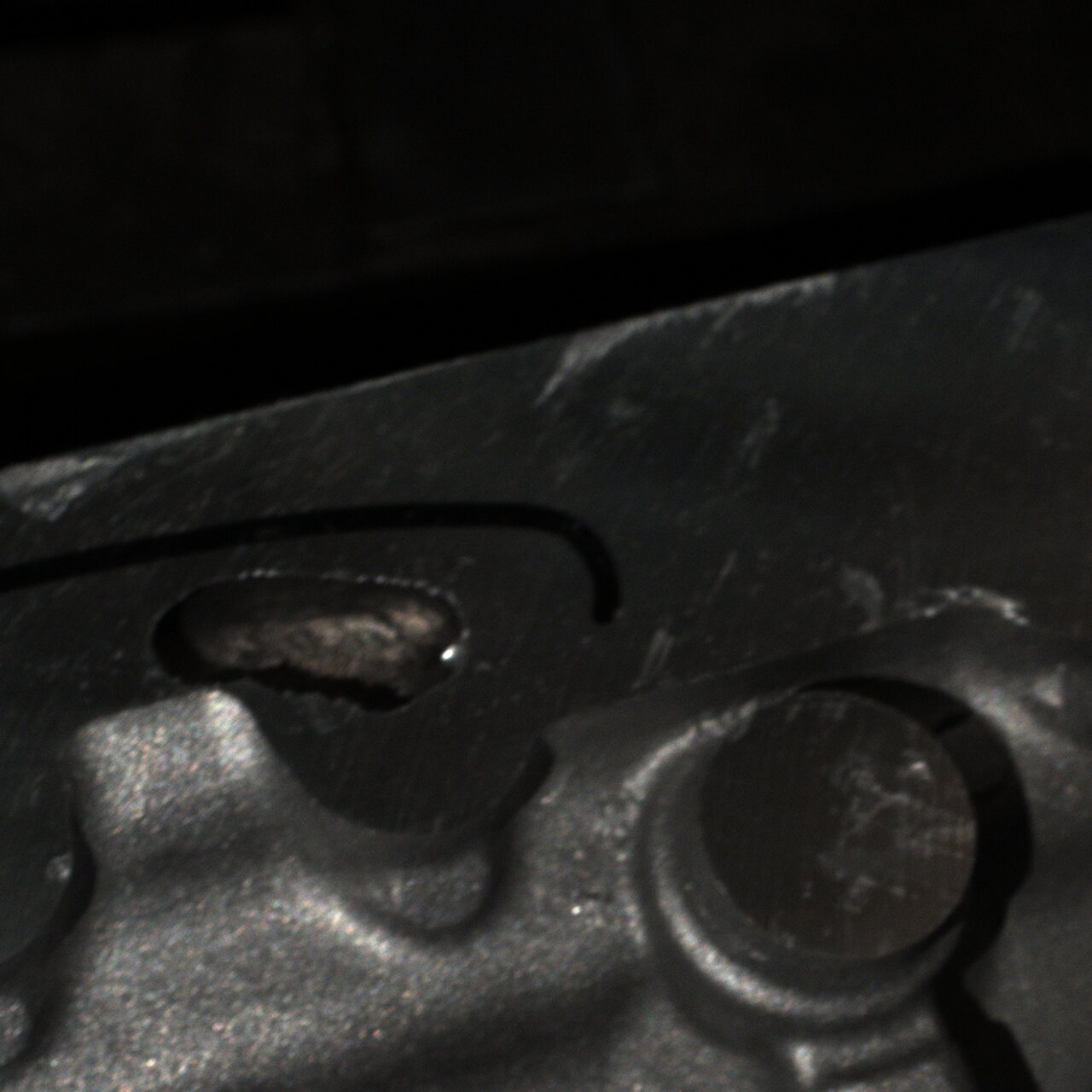

Supplement: Supplemental Information 1 — The CSD-DET dataset was collected from Guangde Hardware Casting Factory and Wuhu Automobile Casting Factory in May 2023. The CSD-DET dataset was used to train and measure the advantages of the DES-YOLO model. This is the filtered partial dataset. [file peerj-cs-10-2224-s001.zip › CastingDefectsDataSet/data/Sh_343.jpg]

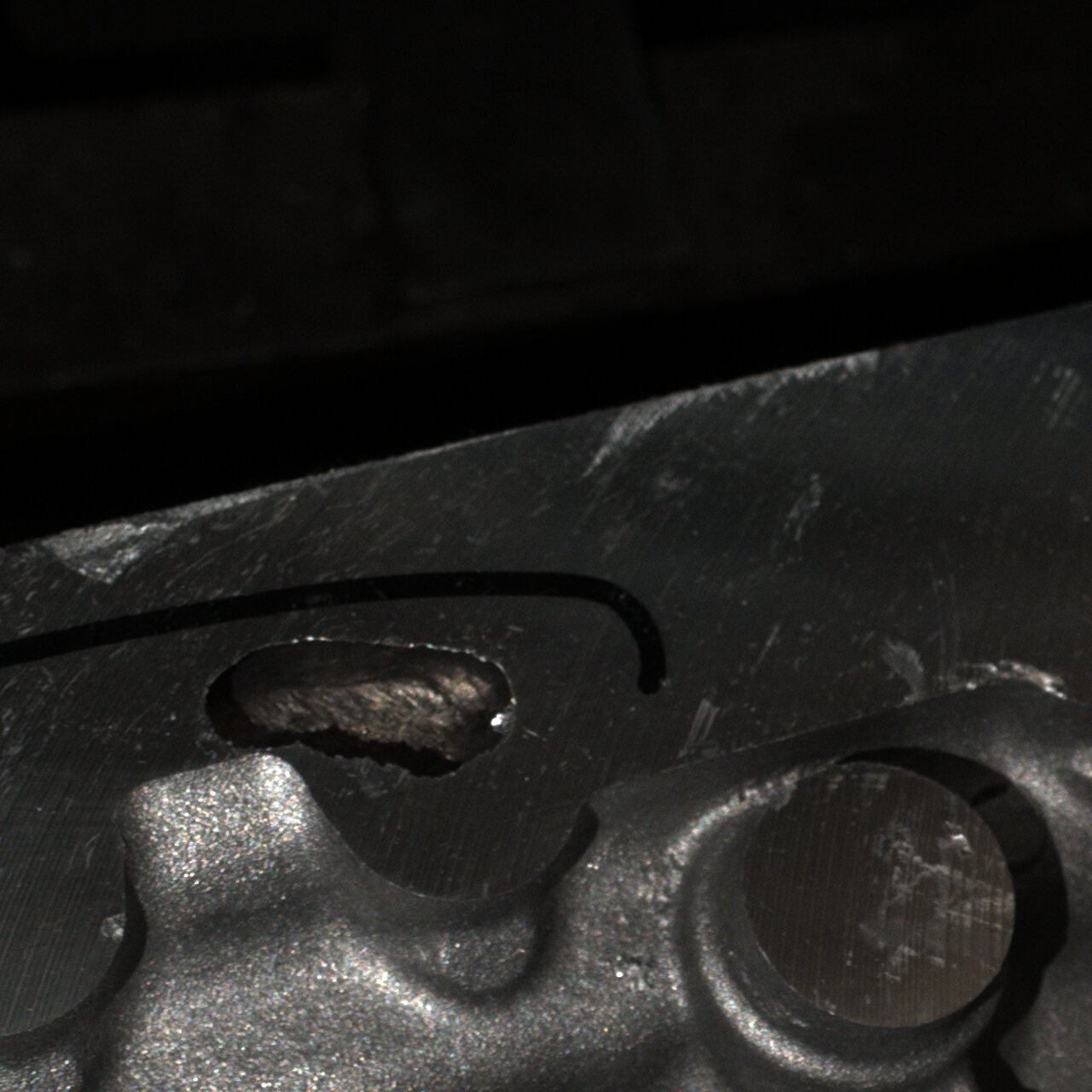

Supplement: Supplemental Information 1 — The CSD-DET dataset was collected from Guangde Hardware Casting Factory and Wuhu Automobile Casting Factory in May 2023. The CSD-DET dataset was used to train and measure the advantages of the DES-YOLO model. This is the filtered partial dataset. [file peerj-cs-10-2224-s001.zip › CastingDefectsDataSet/data/Sh_347.jpg]

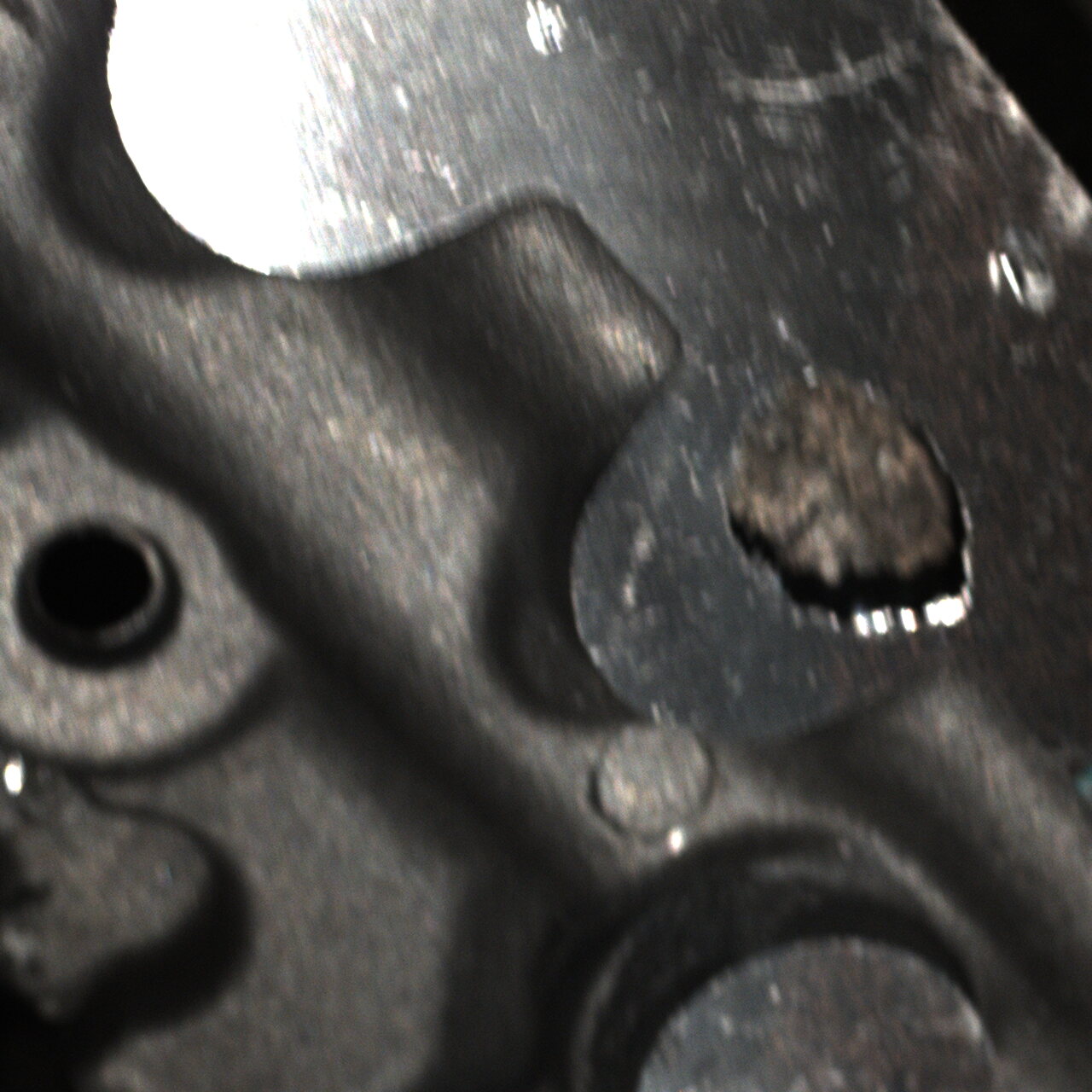

Supplement: Supplemental Information 1 — The CSD-DET dataset was collected from Guangde Hardware Casting Factory and Wuhu Automobile Casting Factory in May 2023. The CSD-DET dataset was used to train and measure the advantages of the DES-YOLO model. This is the filtered partial dataset. [file peerj-cs-10-2224-s001.zip › CastingDefectsDataSet/data/Sh_411.jpg]

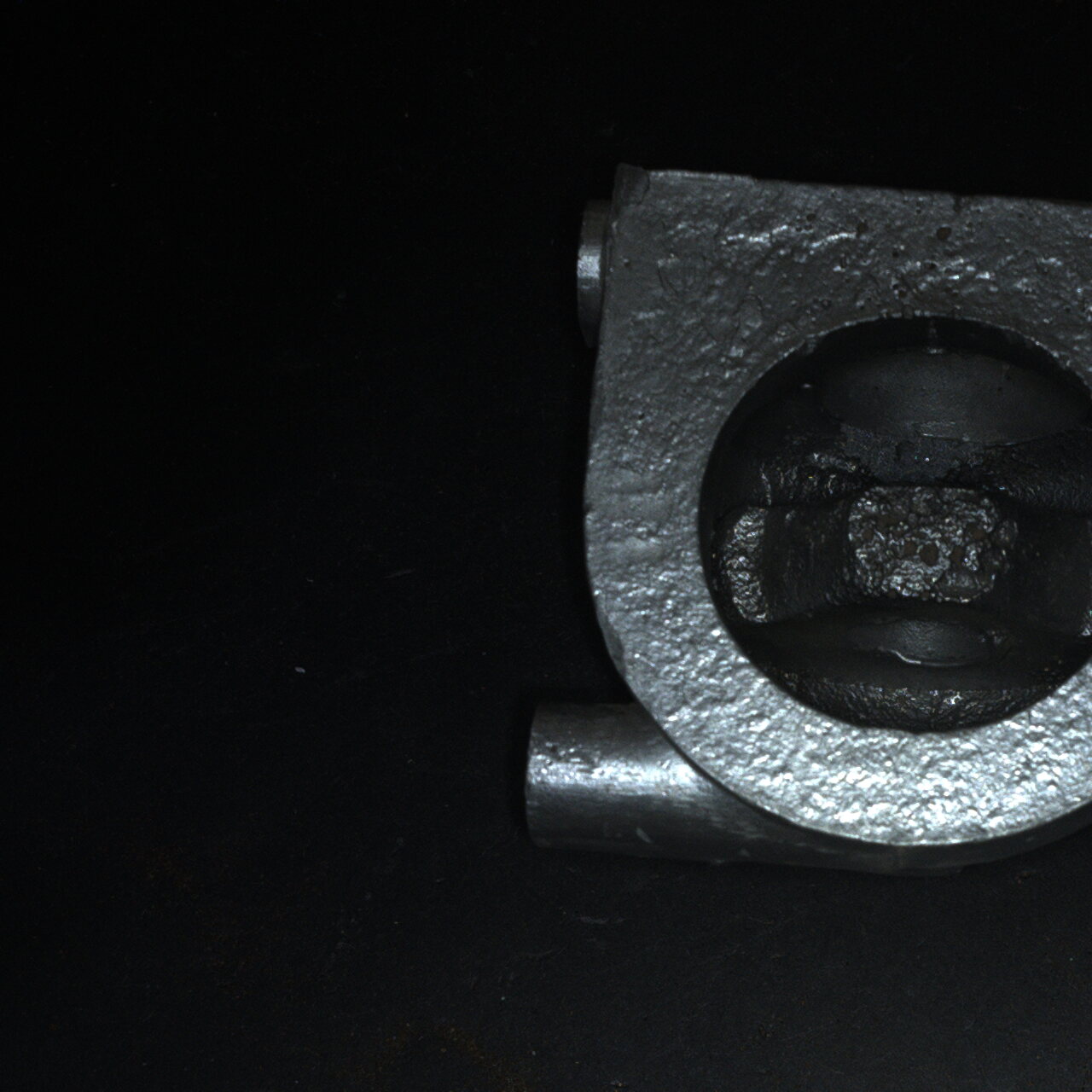

Supplement: Supplemental Information 1 — The CSD-DET dataset was collected from Guangde Hardware Casting Factory and Wuhu Automobile Casting Factory in May 2023. The CSD-DET dataset was used to train and measure the advantages of the DES-YOLO model. This is the filtered partial dataset. [file peerj-cs-10-2224-s001.zip › CastingDefectsDataSet/data/Sh_515.jpg]

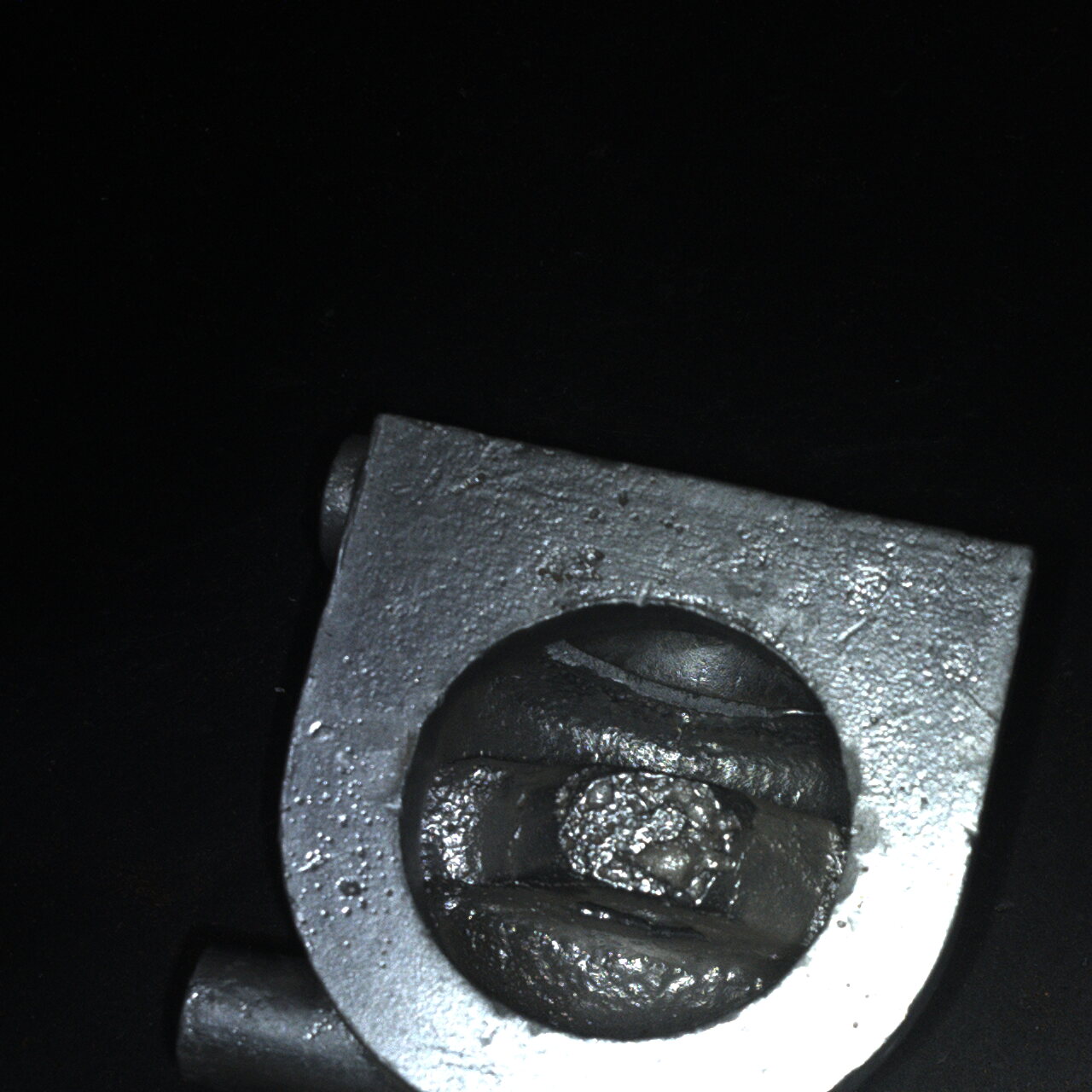

Supplement: Supplemental Information 1 — The CSD-DET dataset was collected from Guangde Hardware Casting Factory and Wuhu Automobile Casting Factory in May 2023. The CSD-DET dataset was used to train and measure the advantages of the DES-YOLO model. This is the filtered partial dataset. [file peerj-cs-10-2224-s001.zip › CastingDefectsDataSet/data/Sh_527.jpg]

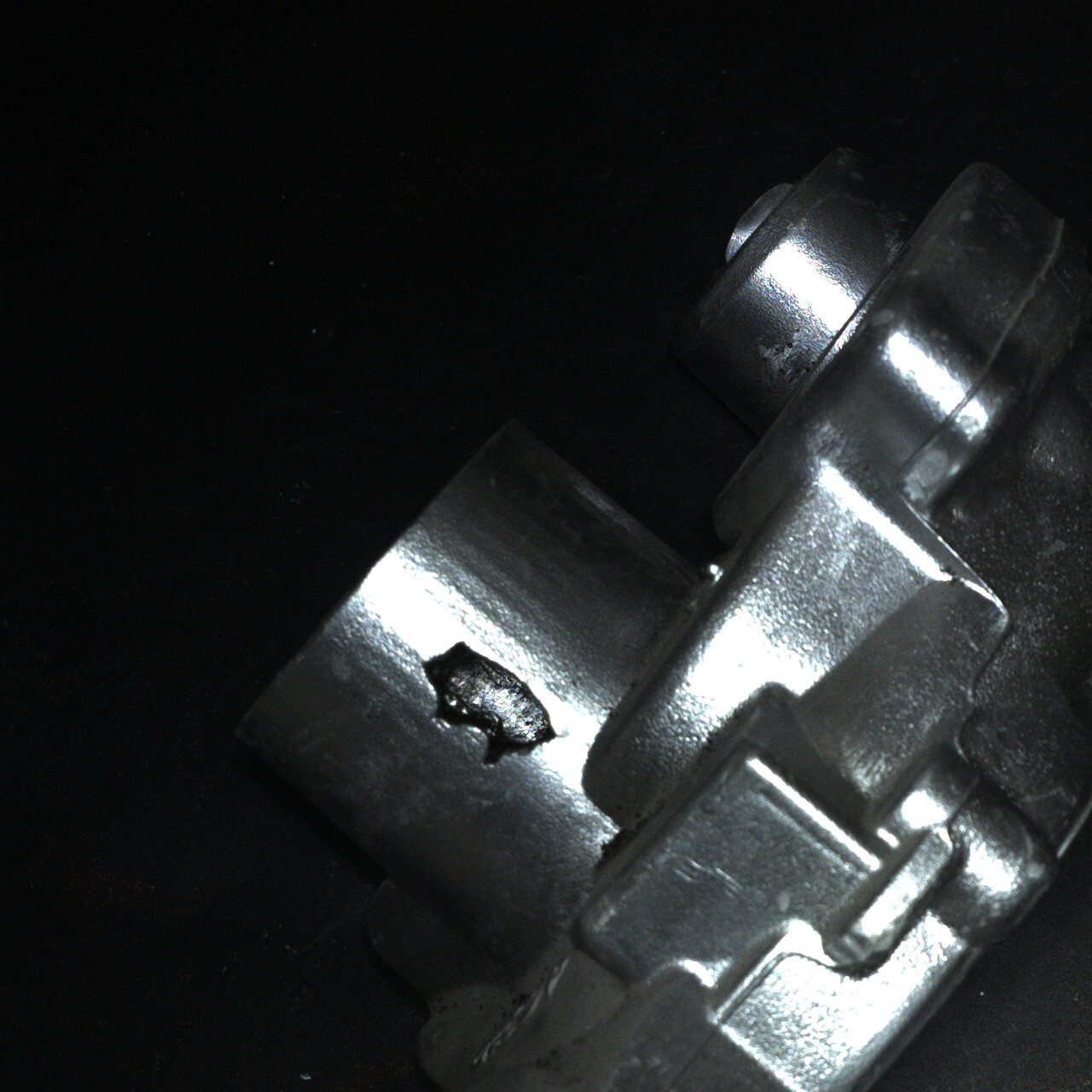

Supplement: Supplemental Information 1 — The CSD-DET dataset was collected from Guangde Hardware Casting Factory and Wuhu Automobile Casting Factory in May 2023. The CSD-DET dataset was used to train and measure the advantages of the DES-YOLO model. This is the filtered partial dataset. [file peerj-cs-10-2224-s001.zip › CastingDefectsDataSet/data/Sh_551.jpg]

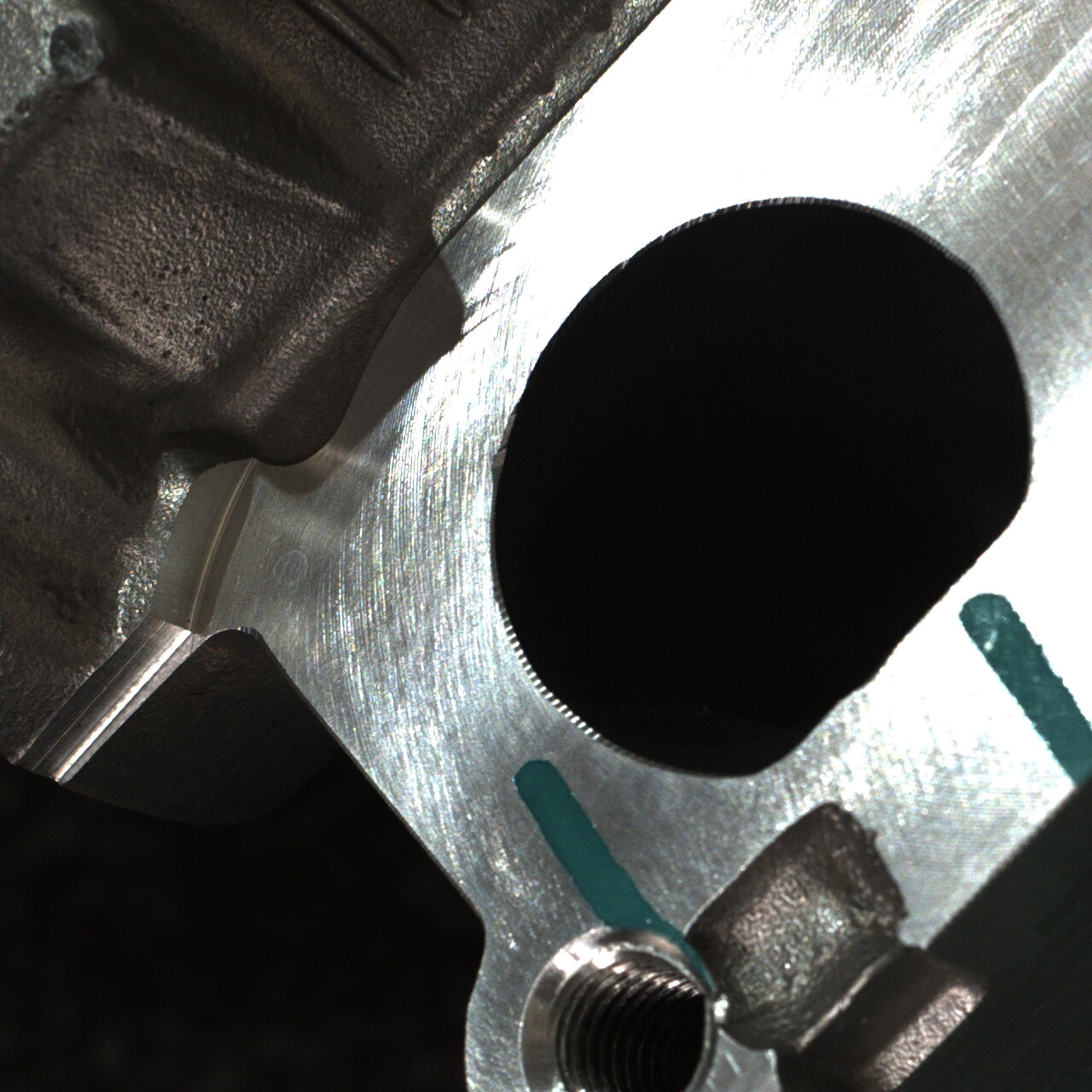

Supplement: Supplemental Information 1 — The CSD-DET dataset was collected from Guangde Hardware Casting Factory and Wuhu Automobile Casting Factory in May 2023. The CSD-DET dataset was used to train and measure the advantages of the DES-YOLO model. This is the filtered partial dataset. [file peerj-cs-10-2224-s001.zip › CastingDefectsDataSet/data/Sh_59.jpg]

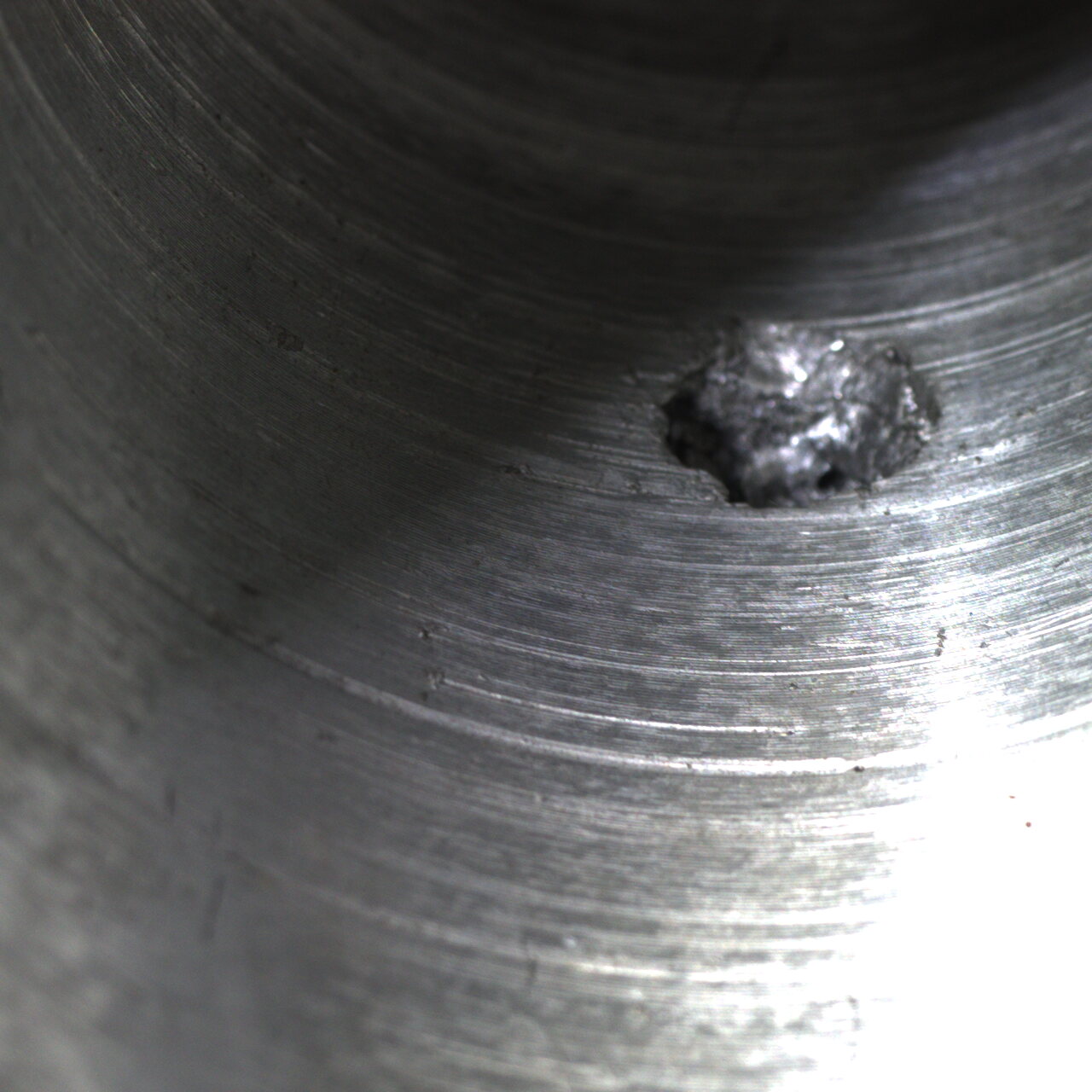

Supplement: Supplemental Information 1 — The CSD-DET dataset was collected from Guangde Hardware Casting Factory and Wuhu Automobile Casting Factory in May 2023. The CSD-DET dataset was used to train and measure the advantages of the DES-YOLO model. This is the filtered partial dataset. [file peerj-cs-10-2224-s001.zip › CastingDefectsDataSet/data/Sh_655.jpg]

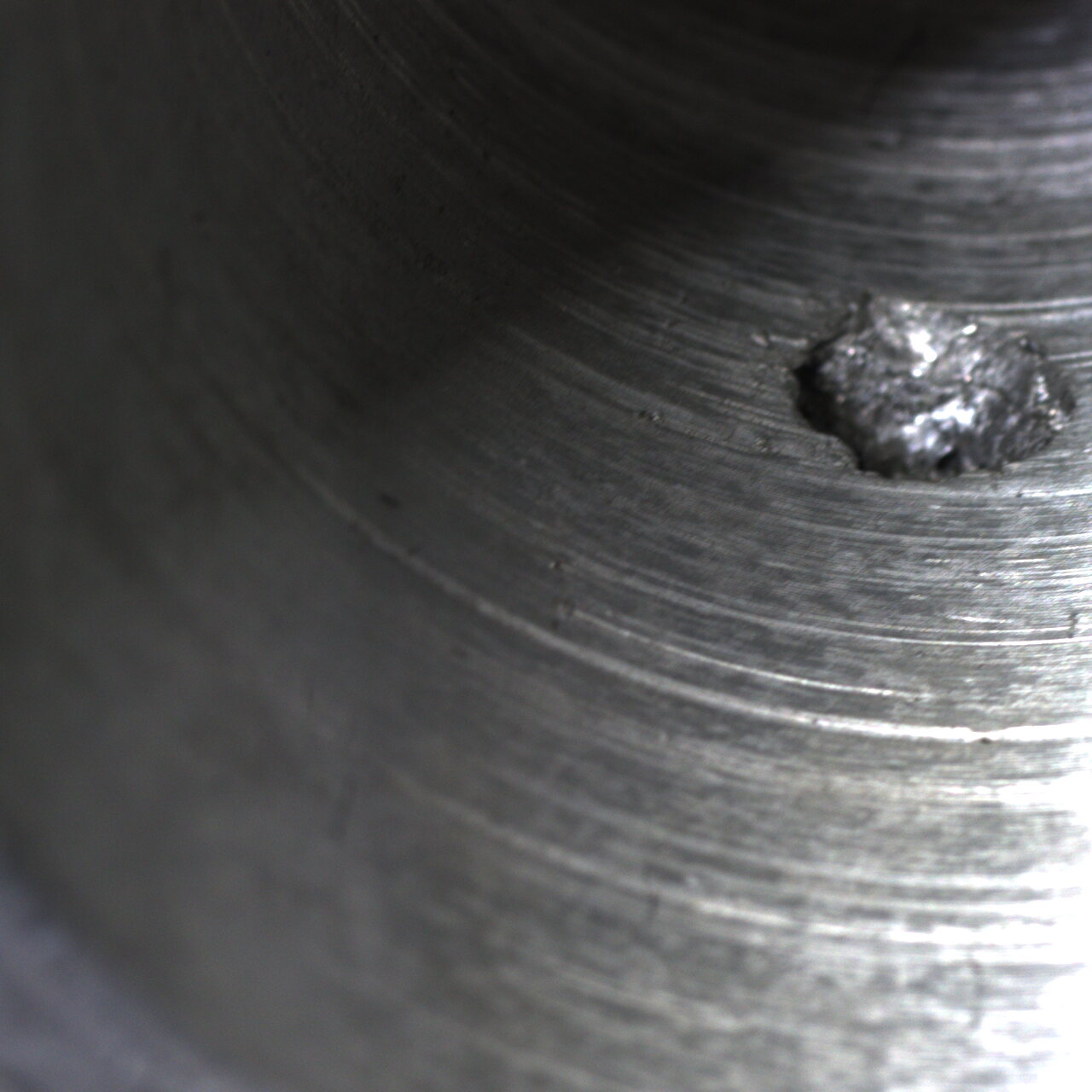

Supplement: Supplemental Information 1 — The CSD-DET dataset was collected from Guangde Hardware Casting Factory and Wuhu Automobile Casting Factory in May 2023. The CSD-DET dataset was used to train and measure the advantages of the DES-YOLO model. This is the filtered partial dataset. [file peerj-cs-10-2224-s001.zip › CastingDefectsDataSet/data/Sh_659.jpg]

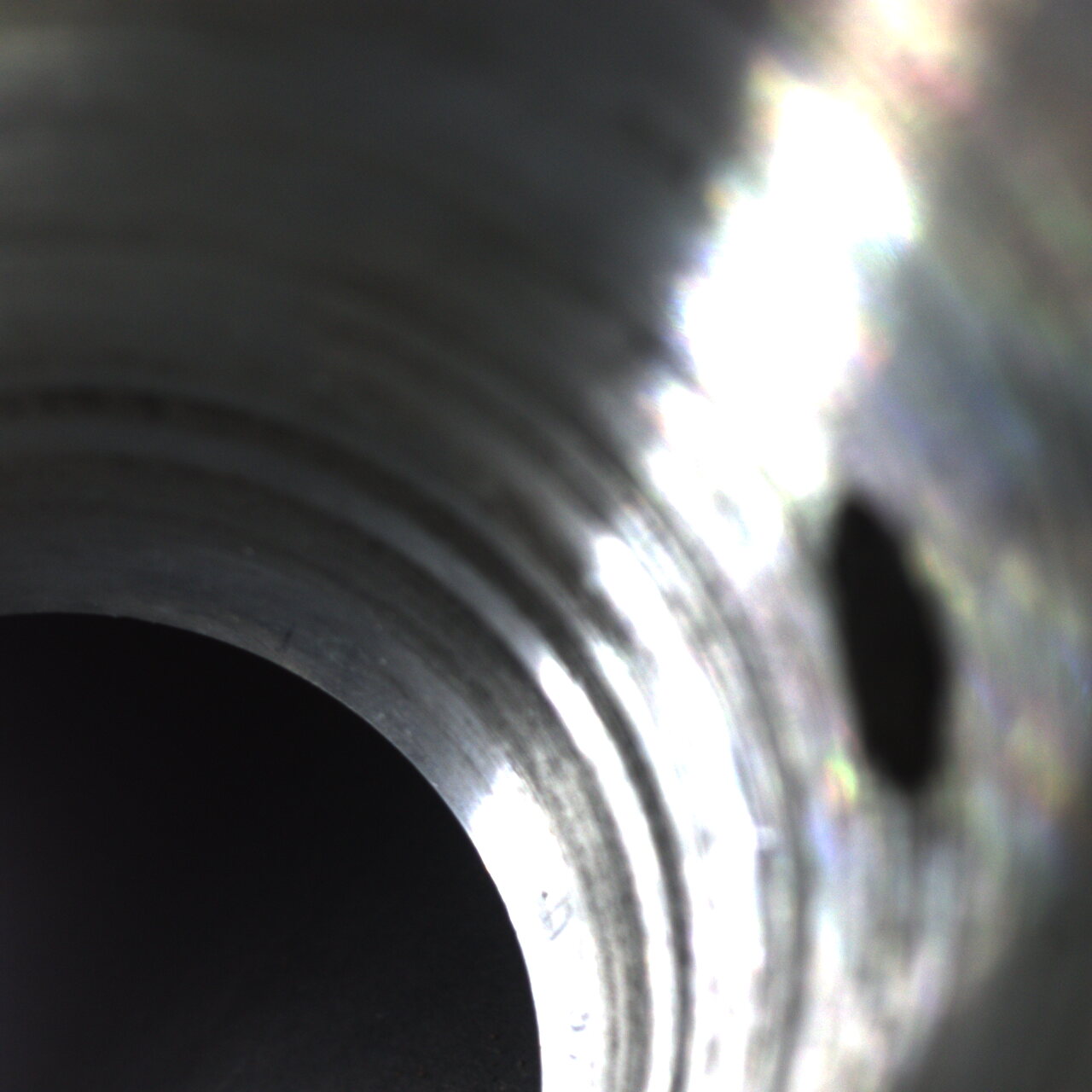

Supplement: Supplemental Information 1 — The CSD-DET dataset was collected from Guangde Hardware Casting Factory and Wuhu Automobile Casting Factory in May 2023. The CSD-DET dataset was used to train and measure the advantages of the DES-YOLO model. This is the filtered partial dataset. [file peerj-cs-10-2224-s001.zip › CastingDefectsDataSet/data/Sh_707.jpg]

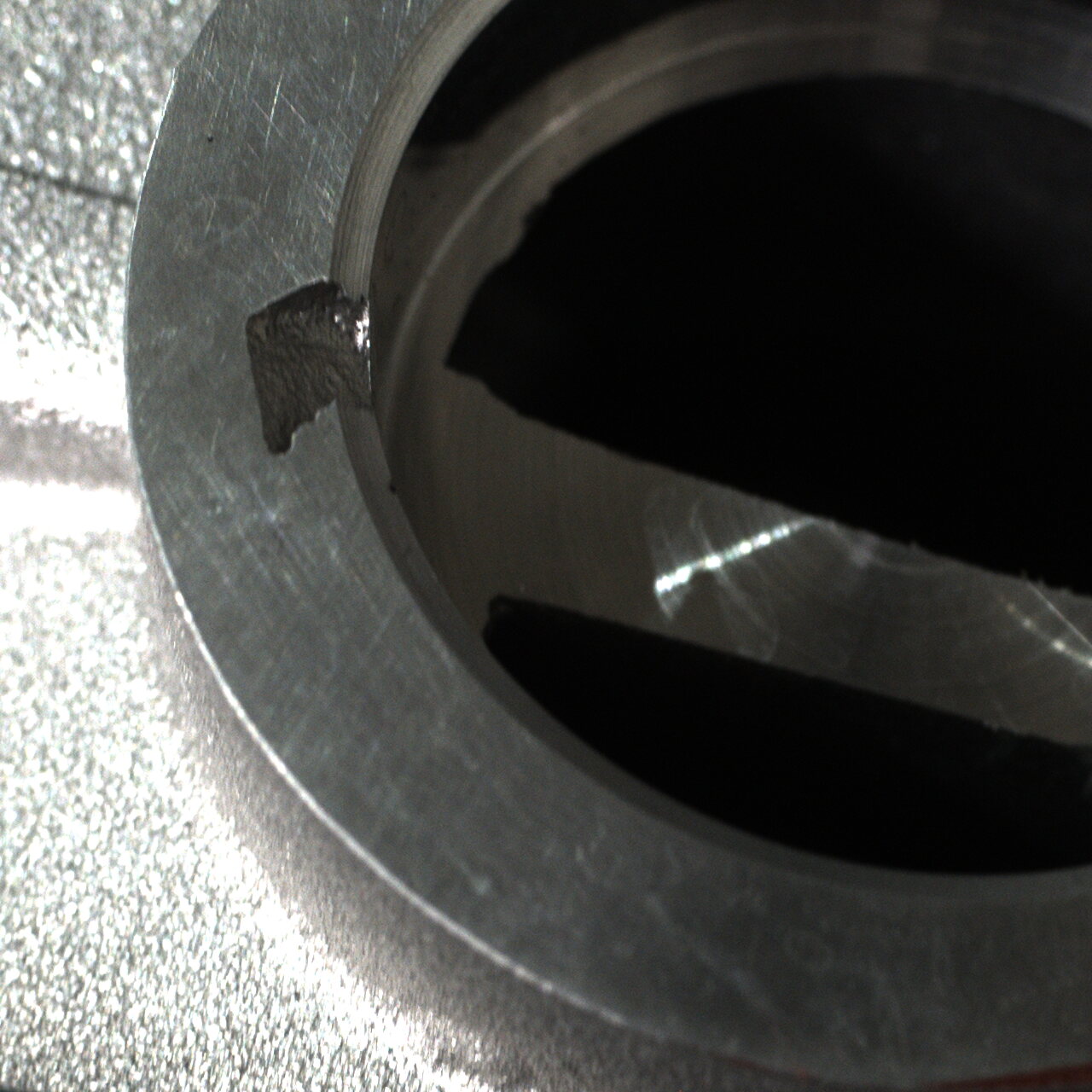

Supplement: Supplemental Information 1 — The CSD-DET dataset was collected from Guangde Hardware Casting Factory and Wuhu Automobile Casting Factory in May 2023. The CSD-DET dataset was used to train and measure the advantages of the DES-YOLO model. This is the filtered partial dataset. [file peerj-cs-10-2224-s001.zip › CastingDefectsDataSet/data/Sh_79.jpg]

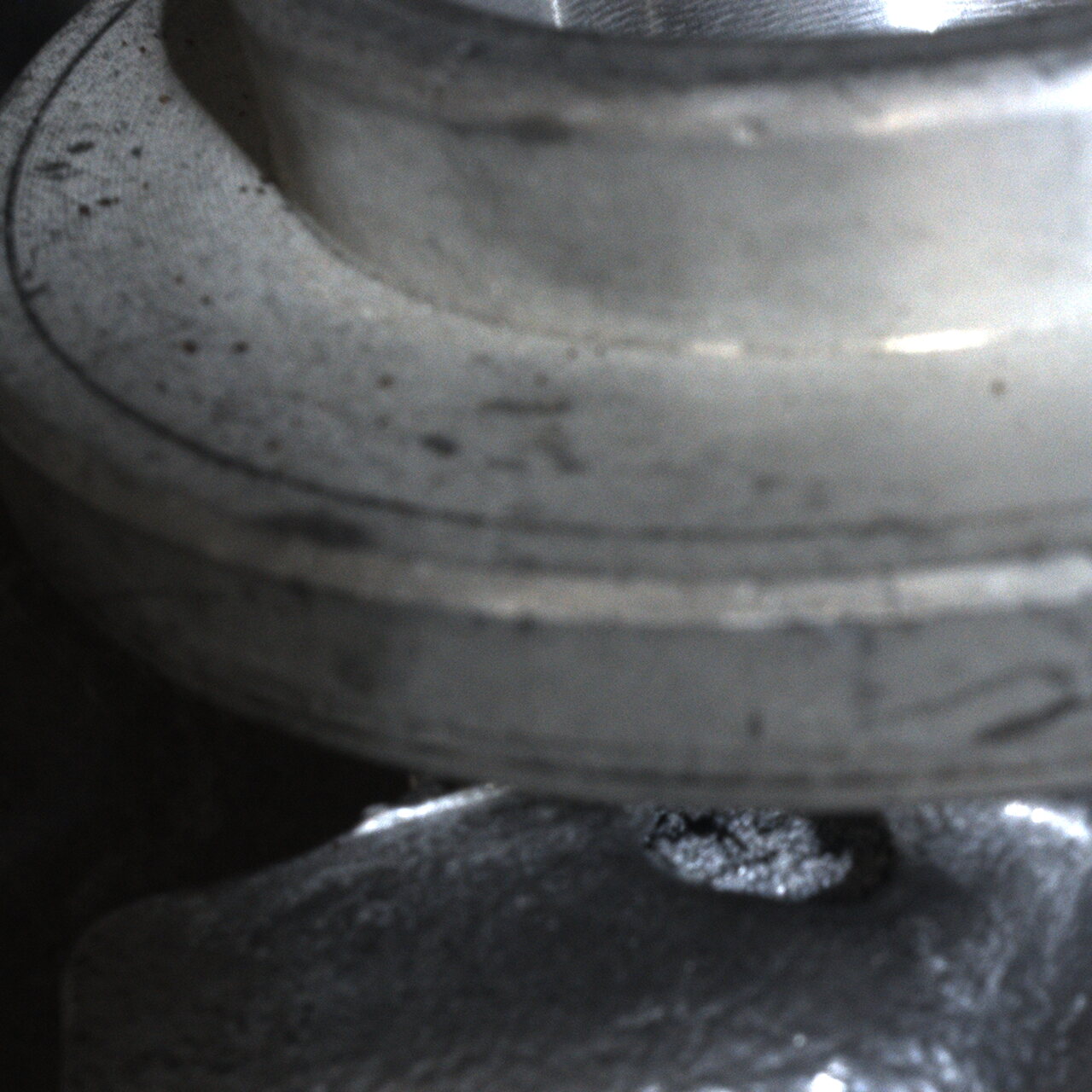

Supplement: Supplemental Information 1 — The CSD-DET dataset was collected from Guangde Hardware Casting Factory and Wuhu Automobile Casting Factory in May 2023. The CSD-DET dataset was used to train and measure the advantages of the DES-YOLO model. This is the filtered partial dataset. [file peerj-cs-10-2224-s001.zip › CastingDefectsDataSet/data/Sh_799.jpg]

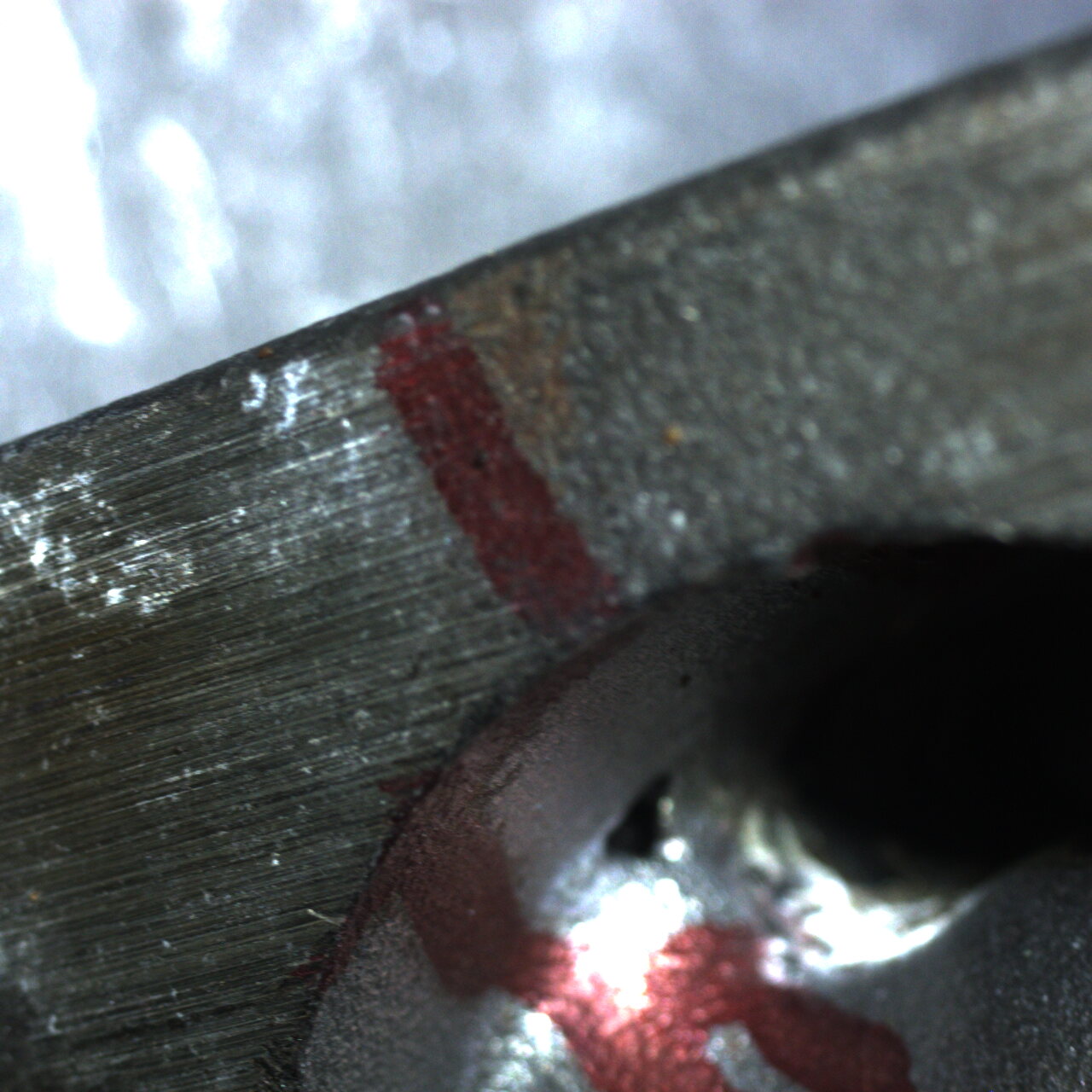

Supplement: Supplemental Information 1 — The CSD-DET dataset was collected from Guangde Hardware Casting Factory and Wuhu Automobile Casting Factory in May 2023. The CSD-DET dataset was used to train and measure the advantages of the DES-YOLO model. This is the filtered partial dataset. [file peerj-cs-10-2224-s001.zip › CastingDefectsDataSet/data/Sh_815.jpg]

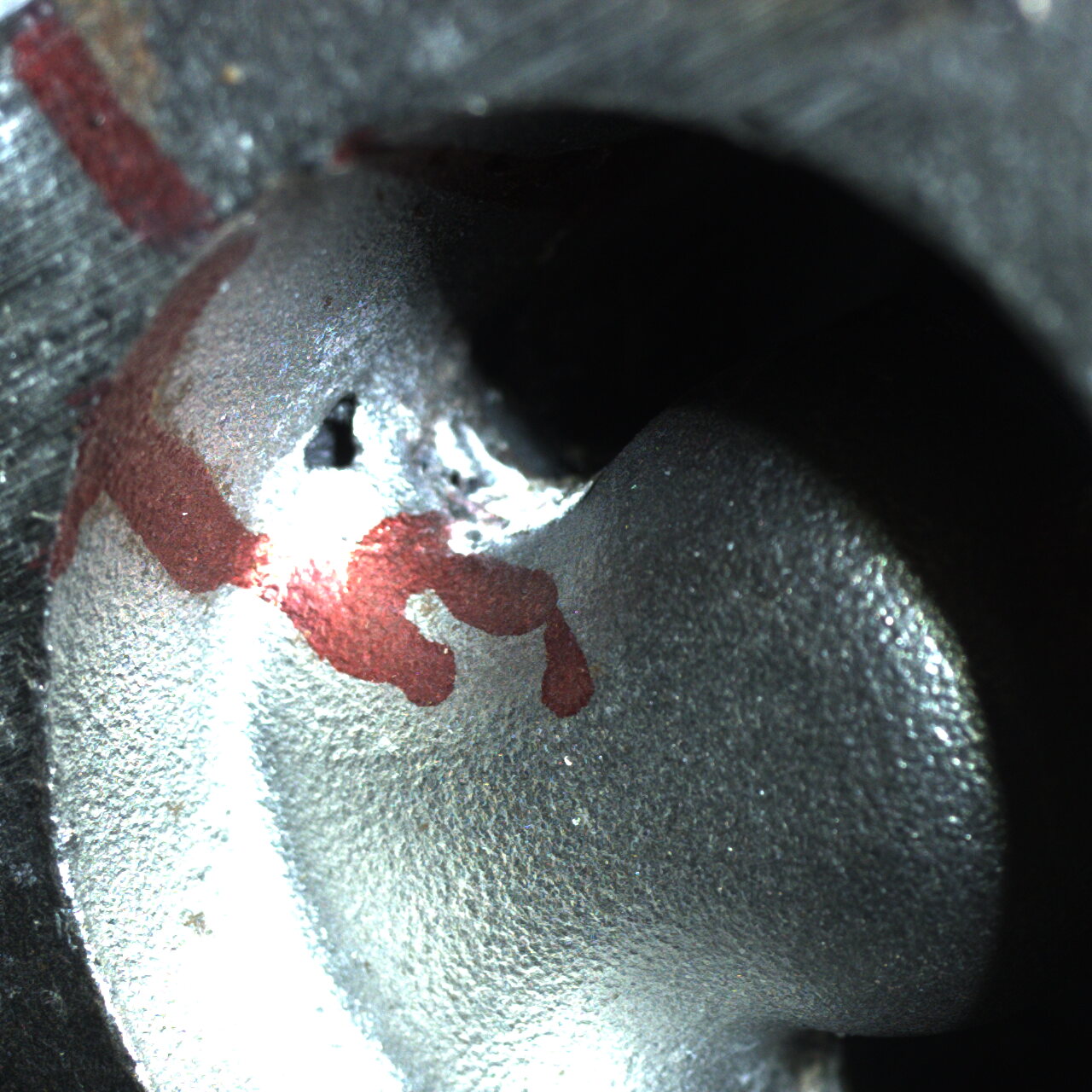

Supplement: Supplemental Information 1 — The CSD-DET dataset was collected from Guangde Hardware Casting Factory and Wuhu Automobile Casting Factory in May 2023. The CSD-DET dataset was used to train and measure the advantages of the DES-YOLO model. This is the filtered partial dataset. [file peerj-cs-10-2224-s001.zip › CastingDefectsDataSet/data/Sh_843.jpg]

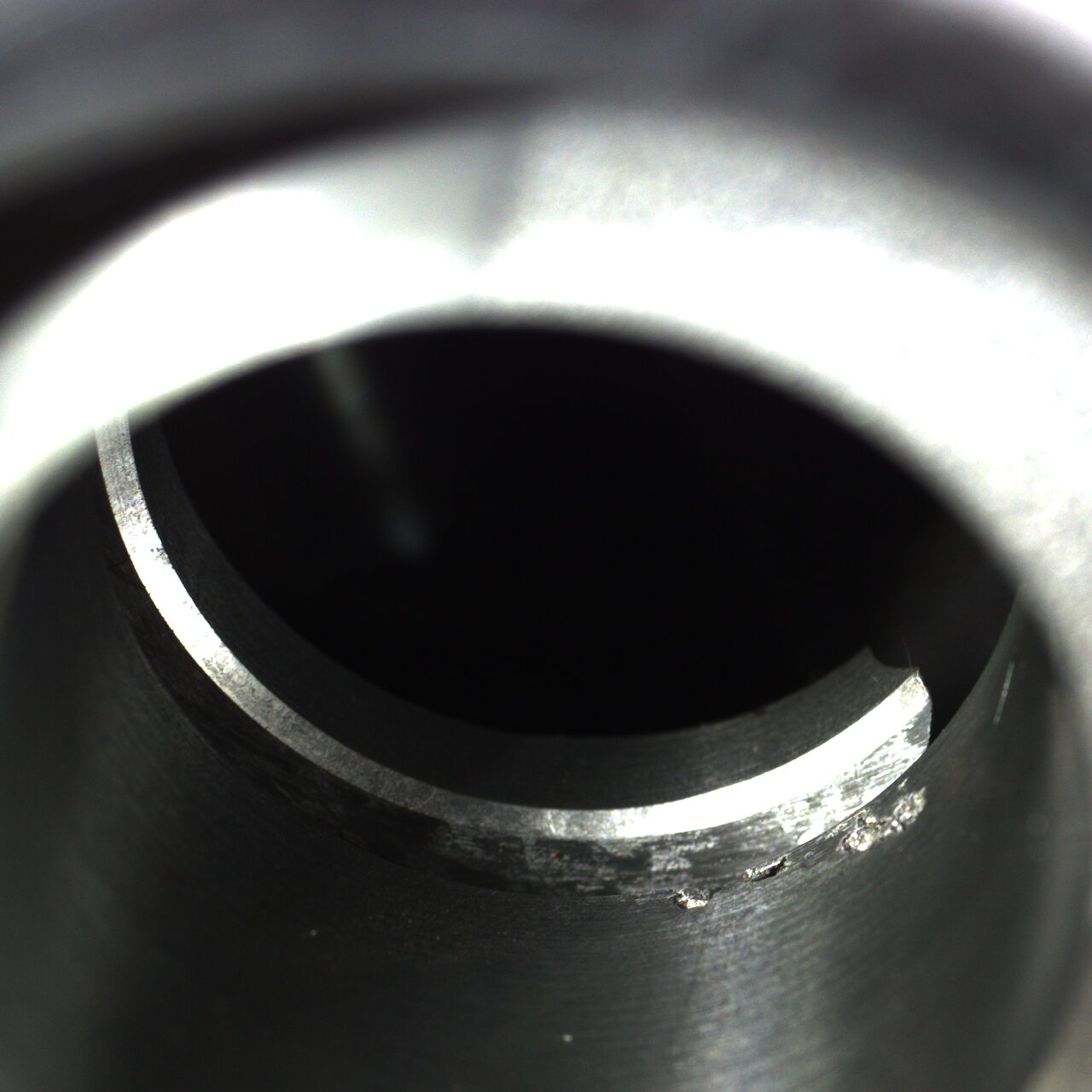

Supplement: Supplemental Information 1 — The CSD-DET dataset was collected from Guangde Hardware Casting Factory and Wuhu Automobile Casting Factory in May 2023. The CSD-DET dataset was used to train and measure the advantages of the DES-YOLO model. This is the filtered partial dataset. [file peerj-cs-10-2224-s001.zip › CastingDefectsDataSet/data/Sl_202.jpg]

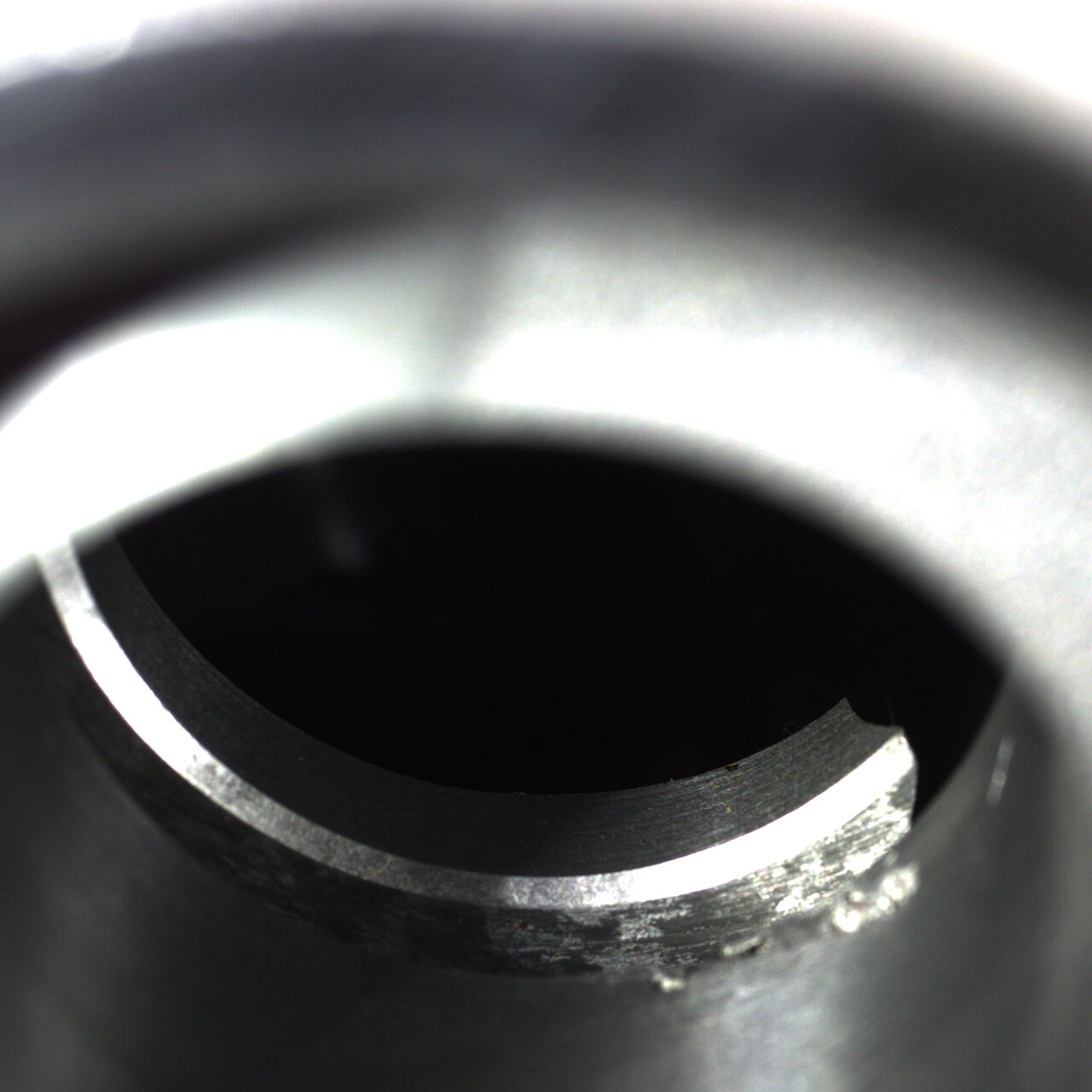

Supplement: Supplemental Information 1 — The CSD-DET dataset was collected from Guangde Hardware Casting Factory and Wuhu Automobile Casting Factory in May 2023. The CSD-DET dataset was used to train and measure the advantages of the DES-YOLO model. This is the filtered partial dataset. [file peerj-cs-10-2224-s001.zip › CastingDefectsDataSet/data/Sl_206.jpg]

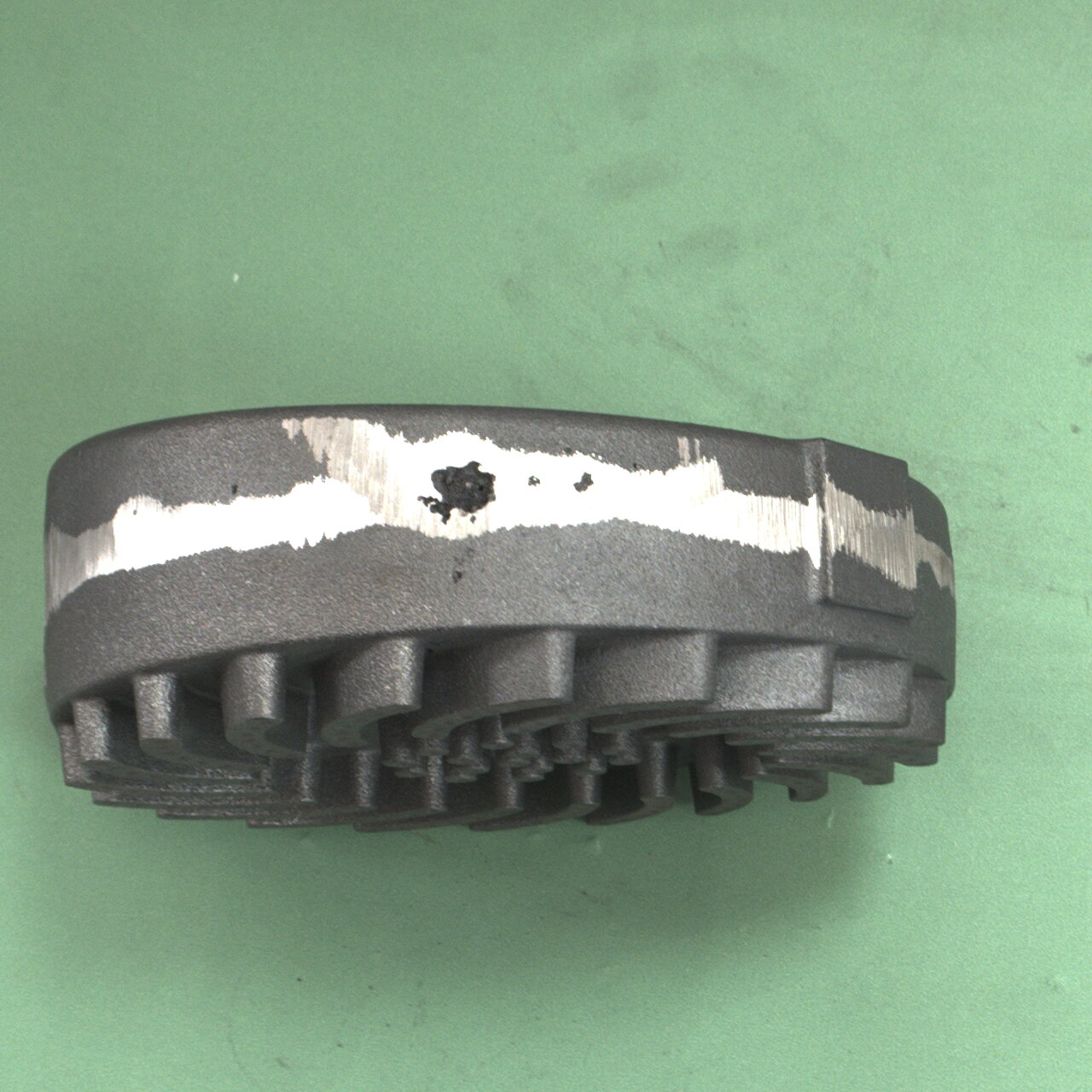

Supplement: Supplemental Information 1 — The CSD-DET dataset was collected from Guangde Hardware Casting Factory and Wuhu Automobile Casting Factory in May 2023. The CSD-DET dataset was used to train and measure the advantages of the DES-YOLO model. This is the filtered partial dataset. [file peerj-cs-10-2224-s001.zip › CastingDefectsDataSet/data/Sl_22.jpg]

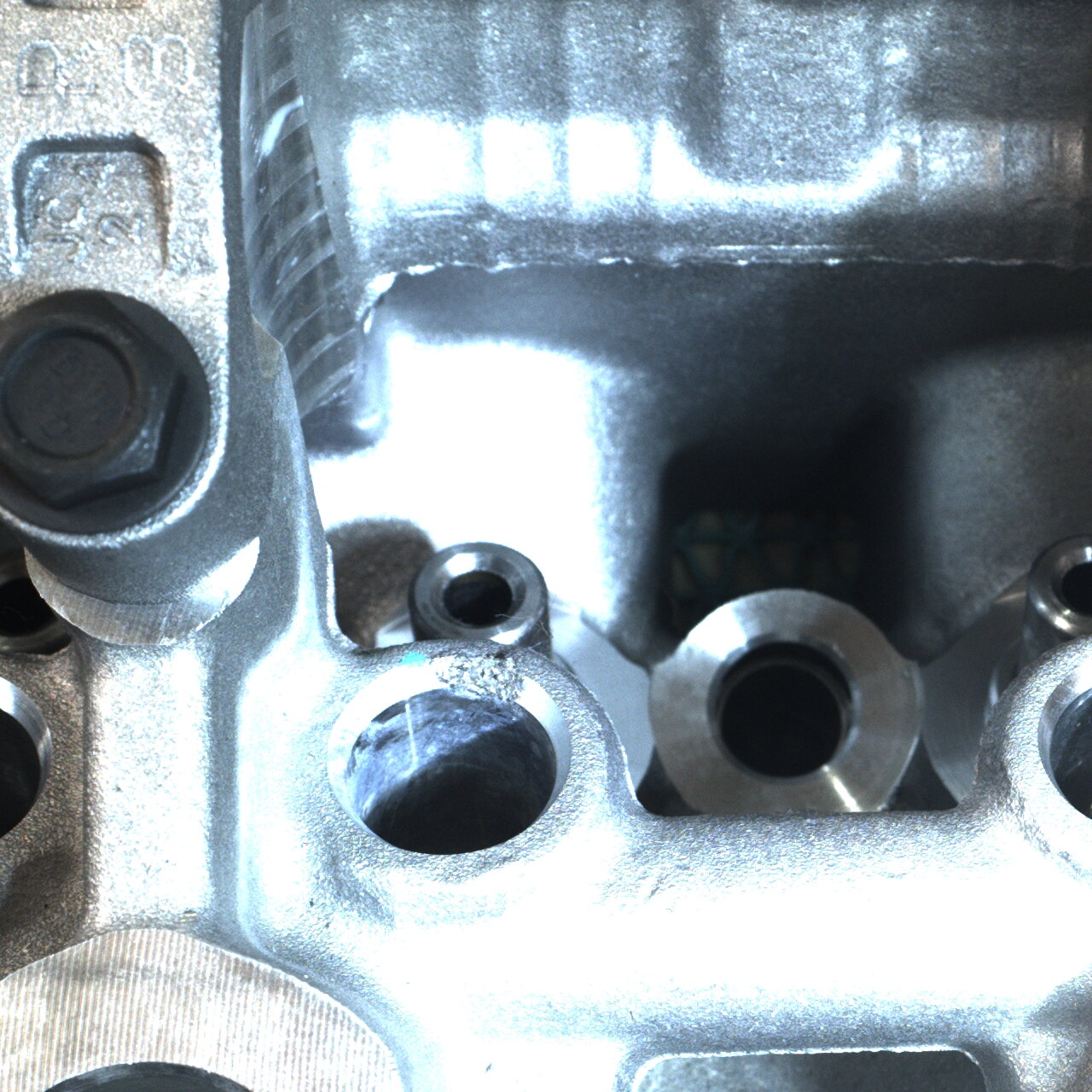

Supplement: Supplemental Information 1 — The CSD-DET dataset was collected from Guangde Hardware Casting Factory and Wuhu Automobile Casting Factory in May 2023. The CSD-DET dataset was used to train and measure the advantages of the DES-YOLO model. This is the filtered partial dataset. [file peerj-cs-10-2224-s001.zip › CastingDefectsDataSet/data/Sl_226.jpg]

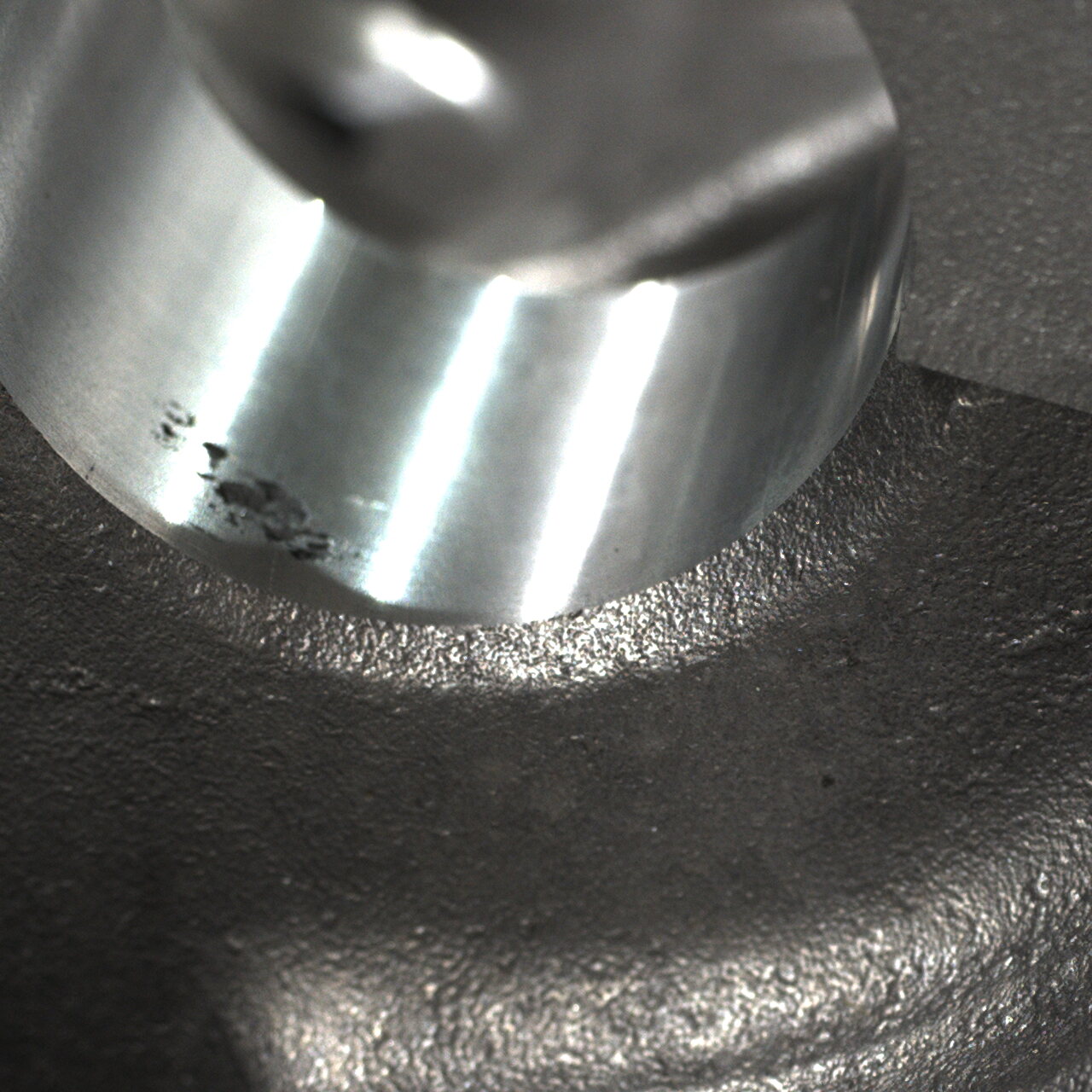

Supplement: Supplemental Information 1 — The CSD-DET dataset was collected from Guangde Hardware Casting Factory and Wuhu Automobile Casting Factory in May 2023. The CSD-DET dataset was used to train and measure the advantages of the DES-YOLO model. This is the filtered partial dataset. [file peerj-cs-10-2224-s001.zip › CastingDefectsDataSet/data/Sl_330.jpg]

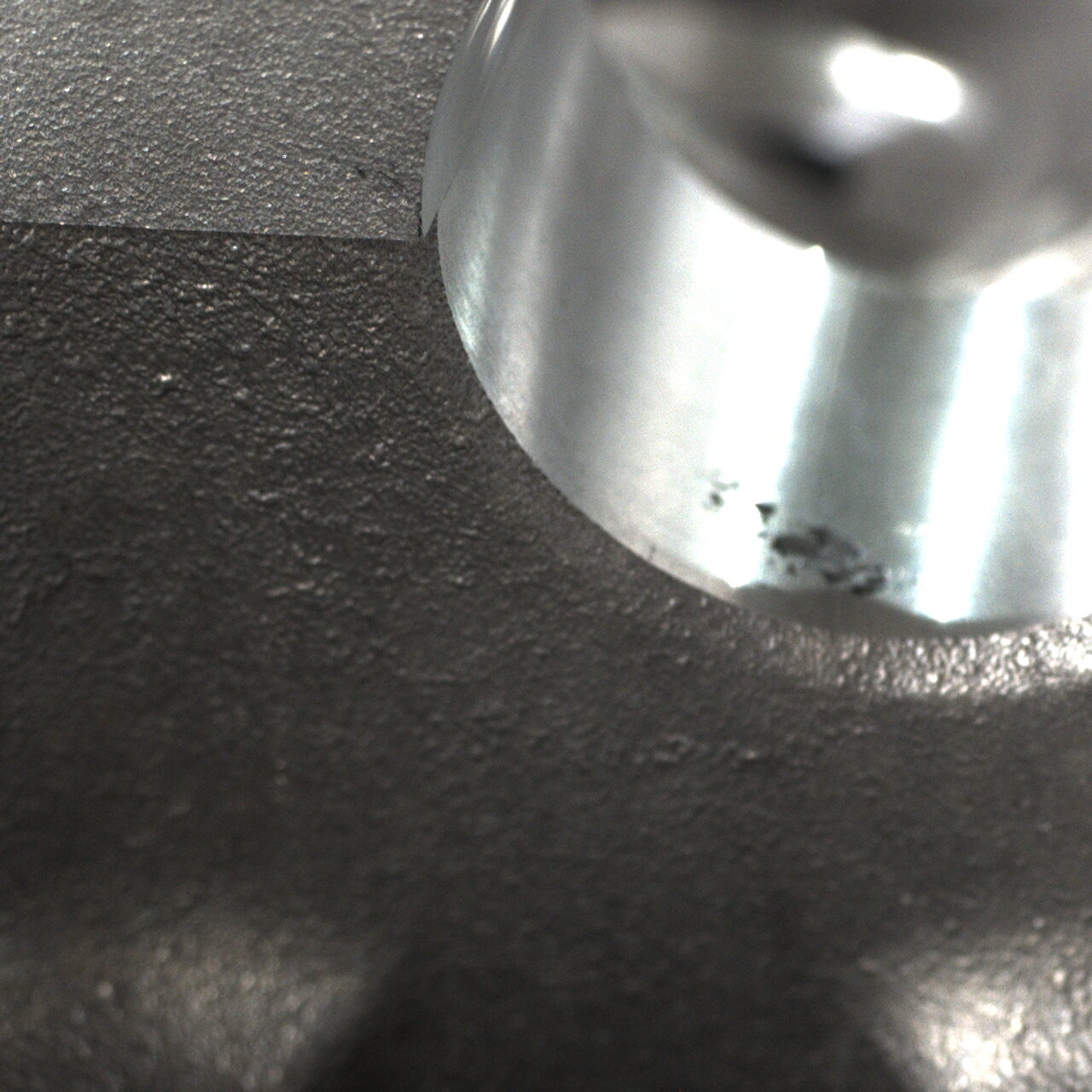

Supplement: Supplemental Information 1 — The CSD-DET dataset was collected from Guangde Hardware Casting Factory and Wuhu Automobile Casting Factory in May 2023. The CSD-DET dataset was used to train and measure the advantages of the DES-YOLO model. This is the filtered partial dataset. [file peerj-cs-10-2224-s001.zip › CastingDefectsDataSet/data/Sl_346.jpg]

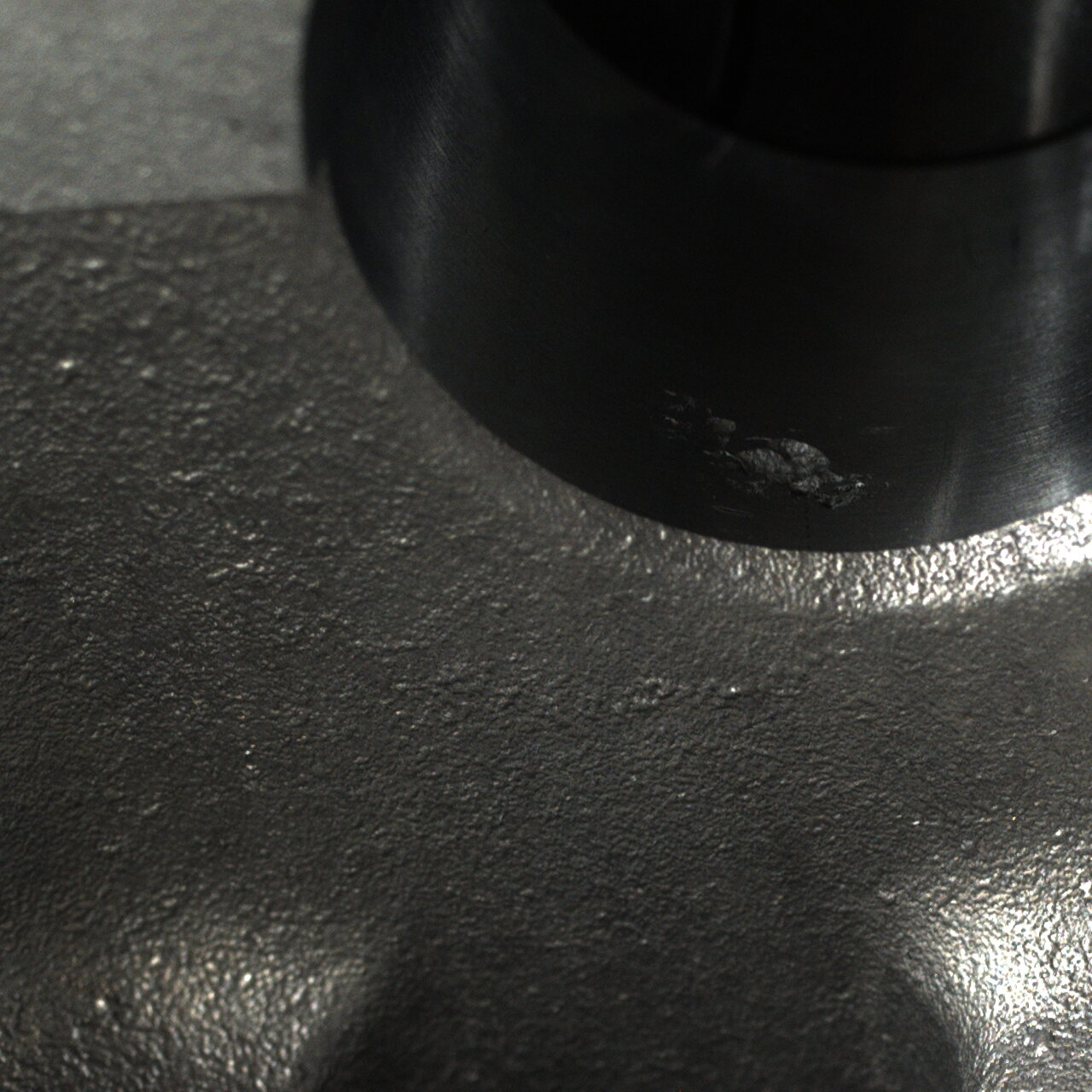

Supplement: Supplemental Information 1 — The CSD-DET dataset was collected from Guangde Hardware Casting Factory and Wuhu Automobile Casting Factory in May 2023. The CSD-DET dataset was used to train and measure the advantages of the DES-YOLO model. This is the filtered partial dataset. [file peerj-cs-10-2224-s001.zip › CastingDefectsDataSet/data/Sl_402.jpg]
